# An expanded library of orthogonal split inteins enables modular multi-peptide assemblies

**DOI:** 10.1038/s41467-020-15272-2

**Published:** 2020-03-23

**Authors:** Filipe Pinto, Ella Lucille Thornton, Baojun Wang

**Affiliations:** 1 School of Biological Sciences, University of Edinburgh, Edinburgh, EH9 3FF UK; 2 Centre for Synthetic and Systems Biology, University of Edinburgh, Edinburgh, EH9 3FF UK

**Keywords:** Proteins, Synthetic biology, Post-translational modifications, Synthetic biology

## Abstract

Inteins are protein segments capable of joining adjacent residues via a peptide bond. In this process known as protein splicing, the intein itself is not present in the final sequence, thus achieving scarless peptide ligation. Here, we assess the splicing activity of 34 inteins (both uncharacterized and known) using a rapid split fluorescent reporter characterization platform, and establish a library of 15 mutually orthogonal split inteins for in vivo applications, 10 of which can be simultaneously used in vitro. We show that orthogonal split inteins can be coupled to multiple split transcription factors to implement complex logic circuits in living organisms, and that they can also be used for the in vitro seamless assembly of large repetitive proteins with biotechnological relevance. Our work demonstrates the versatility and vast potential of an expanded library of orthogonal split inteins for their use in the fields of synthetic biology and protein engineering.

## Abstract (web summary)

Inteins allow the joining of protein segments through scarless ligation. Here the authors assess 34 inteins to establish a library of 15 mutually orthogonal split inteins for in vivo applications, 10 of which are also active in vitro.

## Introduction

Inteins (internal proteins) are auto-catalytic protein segments capable of excising themselves from a larger precursor protein, enabling the flanking extein (external protein) sequences to be ligated through the formation of a new peptide bond—a process known as protein splicing^1,2^. Inteins can be found in all domains of life and in viruses, and are usually embedded within essential proteins^3–5^. Inteins can be bifunctional, containing an endonuclease domain inserted within the splicing domain; mini-inteins, if they lack the endonuclease domain; or naturally split, existing as two fragments encoded by two independently transcribed and translated genes, each fused to one extein half^1^. Split inteins can subsequently self-associate non-covalently and catalyze protein splicing *in trans*^2^.

The ability of inteins to seamlessly ligate two peptides or to selectively release the N- or C-extein portion has been extensively exploited for protein modification and purification, to produce intein-based biosensor and reporter systems, and even in gene therapy^6–13^.

The feasibility of using inteins in combination with other recombinant proteins is dictated by the residues in the vicinity of the insertion site and deviation from these preferred junction sequence may lead to reduced splicing activity^2,6,14^. Although this issue has been partially addressed by engineering more permissive inteins^14–16^, there are still limitations to their use. A larger set of fully characterized inteins would increase the chance of finding an intein with a junction sequence preference compatible with the sequence of the target protein. Moreover, most inteins reported so far have been characterized independently under different conditions and only a very limited number of inteins have been shown to be orthogonal^17,18^. Identifying more inteins that can be used simultaneously, under the same experimental conditions and with no cross-reactivity, would significantly expand their applications.

Here, we develop and employ a characterization platform using split mCherry to provide a comparable framework to evaluate the functionality of 34 previously reported and uncharacterized inteins under similar conditions, and we establish an extended library of orthogonal split inteins that can be used together in vivo or in vitro. We demonstrate that these orthogonal split inteins can be coupled to split transcription factors to build logic gates^19^ that can be simultaneously integrated into more complex genetic circuits in vivo^20^. Further, we show their use for the modular in vitro seamless assembly of large repetitive proteins with biotechnological applicability. This study illustrates the vast potential and versatile applications of orthogonal split inteins, ranging from engineering complex cellular regulatory networks^21^ to designing synthetic proteins with bespoke traits^22^.

## Results

### Intein library candidates’ selection

We aim to establish an extended library of orthogonal split inteins and to demonstrate their simultaneous use in synthetic biology or protein engineering approaches. To this end, we selected 11 split inteins that were characterized in previous studies, showing high splicing reaction rates and efficiencies. We also selected three additional reported inteins: NrdA-2, previously identified but not yet characterized^23^; PfuRIR1-1 (PI-*Pfu*I), shown to work *in trans* when containing the endonuclease domain^24^ and MjaKlbA, shown to work *in cis*^25^ but not investigated *in trans*.

To expand this set we searched “The Intein Database and Registry”^5^ (InBase) for viral and viral-like inteins with the premise that, owing to the short duration of the viral life cycle, these inteins would display faster splicing rates. Following an in silico analysis to identify putative homing endonuclease domains, we performed a structural alignment and shortlisted the 50 inteins initially identified (Supplementary Table 1) down to 20 phylogenetically distant inteins (Supplementary Fig. 1), with the assumption that homology negatively correlates with orthogonality. Most of the inteins selected were hypothetical and never characterized before.

**Fig. 1:**
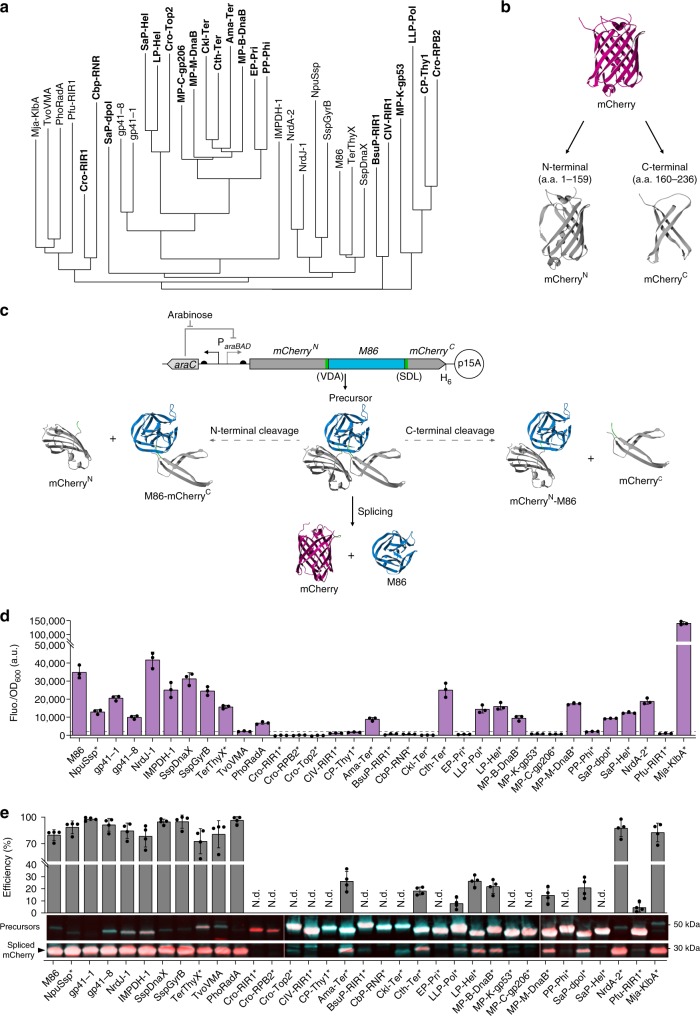
*Cis*-splicing characterization of 34 selected inteins. **a** Phylogram based on the structural alignment of the 34 inteins selected for characterization. The inteins in bold were retrieved from InBase. **b** Structure of the fluorescent reporter mCherry and representation of the split site used to construct the characterization platform. **c** Genetic circuit for the arabinose-induced expression of the mCherry precursor (top) and schematics of protein splicing and side reactions that mCherry-intein fusion proteins (precursors) can undergo, exemplified using M86 intein (bottom): splicing will reconstitute the mCherry reporter by the excision of the intein while in N- or C-termini cleavage the reporter is not reconstituted. Promoters are represented by straight angle arrows and RBS by black semi-circles. Interrupted mCherry halves are shown in gray, M86 intein in blue, added junction sequence residues (VDA and SDL) in green and the spliced mCherry in purple. **d** Background subtracted fluorescence (Fluo.) of *E. coli* cells expressing the precursor proteins, normalized to the cell density (OD_600_). Bars represent the mean of three biological replicates (*n* = 3) and error bars correspond to s.d. The dashed gray line corresponds to a threshold of 2000 a.u. **e** Intein *cis*-splicing efficiency (top) and Western blot analysis (bottom) of the cultures used in **d**. Efficiencies were calculated from the band intensities detected using antibodies against the N-terminal of mCherry (red signal) and against the hexahistidine tag (H_6_) at the C-terminus (turquoise signal). The overlap of both signals is white. Bars represent the mean of the splicing efficiency calculated using signal quantifications from both antibodies and from two distinct membranes (*n* = 4 band signal intensities). Error bars correspond to s.d. *N.d.* not detected; * inteins with a flexible linker at canonical split site. Source data are provided as a Source Data file.

In total, we selected 34 candidate inteins for characterization: 14 from the literature and 20 from InBase (Fig. 1a and Supplementary Tables 2, 3). These inteins have different native exteins and share low sequence homology, thus they are unlikely to descend from a common ancestor intein. Therefore, they should not interact with one another and likely be orthogonal. These inteins were synthesized as mini-inteins and, apart from the 11 inteins previously characterized *in trans*, for all the rest a flexible linker was added at the canonical endonuclease insertion site to allow for structural flexibility and proper protein folding.

### A split mCherry platform for rapid intein characterization

As most characterization of inteins to date relied on Western blots, which can be time consuming and impractical for large-scale assays, we developed a reporter system based on the fluorescent protein mCherry for the rapid assessment of intein functionality and orthogonality. We proposed that non-fluorescent mCherry-intein chimeras would have their fluorescence reconstituted after splicing, providing a fast indirect measure of *cis*- or *trans*-splicing activity.

Based on a previous study^26^, we identified mCherry residues 159–160 as a target site for the insertion of intein sequences and to produce a non-fluorescent split reporter (Fig. 1b). To confirm the loss of fluorescence of mCherry-split at this position, we measured the fluorescence of *Escherichia coli* cells simultaneously expressing the two protein halves. We observed that the expression of the C-terminal half was exceptionally low, but adding the three residues corresponding to the native gp41-1 intein junction sequence (SSS) to the N-terminus of the protein greatly improved expression levels. No fluorescence could be detected, even when the two reporter halves were co-expressed at high levels (Supplementary Fig. 2), thus confirming that the split mCherry halves do not self-associate.

In order to characterize inteins close to their native context, we constructed mCherry variants with the cognate native intein junction sequences inserted between positions 159 and 160, to mimic the final splicing products. The assay of these variants showed that the insertion of the extra residues did not abolish mCherry fluorescence but reduced the maximum output levels to different extents (Supplementary Fig. 3). We noted such reduction was owing to changes in protein expression levels instead of protein intrinsic fluorescence reduction (Supplementary Fig. 4). Nonetheless, the resulting signal intensities remained sufficiently high for the assessment of the mock spliced product.

Our results demonstrate the feasibility of using split mCherry as a robust reporter system to rapidly assess intein functionality and orthogonality.

### Intein *cis*-splicing activity

We first tested the *cis*-splicing activity of the 34 synthesized inteins, mainly to assess the performance of the uncharacterized proteins. For this purpose, we introduced their sequences between the native junction residues present in the respective mCherry variants. These insertions should abolish mCherry fluorescence unless the inteins excise themselves from the precursor protein (Fig. 1c). In all, 22 out of 34 *E. coli* cultures expressing the precursor proteins exhibited fluorescence levels above an arbitrarily defined threshold of 2000 a.u. (Fig. 1d) and, with the exception of the precursors containing PP-Phi and SaP-Hel inteins, the fluorescence detected was indeed due to protein splicing, as confirmed by Western blot (Fig. 1e). It is likely that the PP-Phi and SaP-Hel structures bring the reporter halves in such proximity that allows mCherry reconstitution without splicing. A flexible linker was also added at the canonical endonuclease insertion site of NpuSsp and TerThyX, which significantly improved TerThyX *cis*-splicing activity (Supplementary Fig. 5). Our results show that the inteins retrieved from InBase overall displayed lower efficiencies (8–26%) when compared with the inteins selected from previous studies (efficiencies > 70%) (Fig. 1e).

Using our split mCherry characterization platform we successfully detected the activity of all the 12 inteins previously reported in the literature and have identified eight additional functional inteins (Fig. 1d, e). In total, 20 inteins exhibited *cis*-splicing activity.

### Intein *trans*-splicing in vivo

**Fig. 2:**
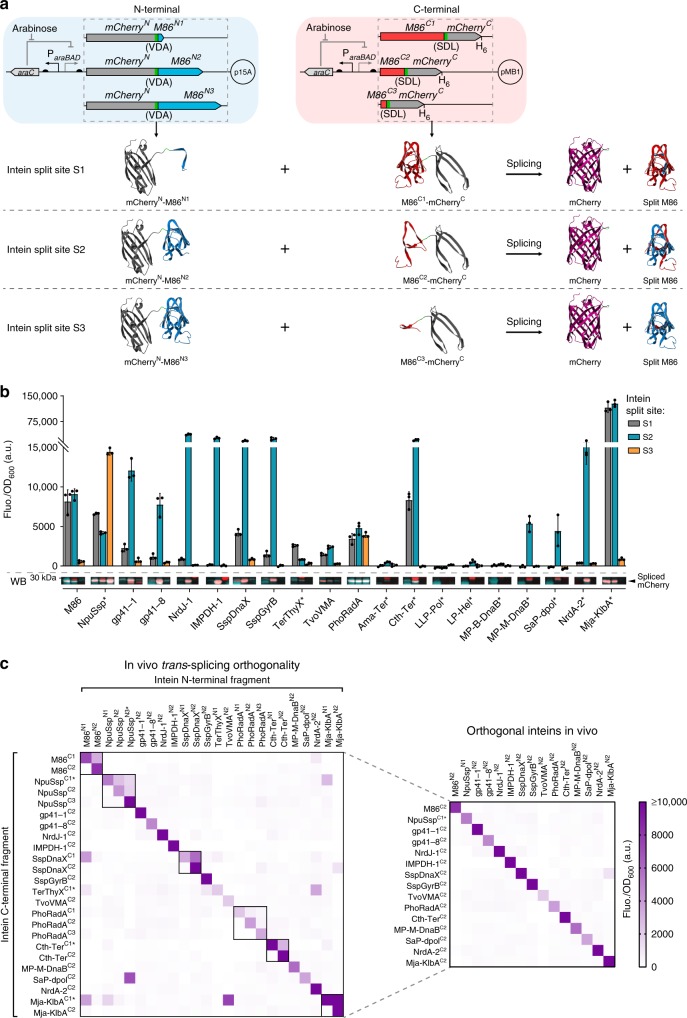
In vivo *trans*-splicing of inteins split at different sites and orthogonality characterization. **a** Genetic circuits for the arabinose-induced expression of split mCherry-split intein N- and C-terminal chimeric proteins from two different plasmids (top) and schematics of protein *trans*-splicing, exemplified using M86 intein (bottom). The three intein split sites are depicted: S1 at the N-terminus, S2 at the canonical endonuclease insertion site and S3 at the C-terminus. Promoters are represented by straight angle arrows and RBS by black semi-circles. Split mCherry halves are shown in gray, M86 intein N-terminal in blue, M86 intein C-terminal in red, added junction sequence residues (VDA and SDL) in green and the spliced mCherry in purple. **b** Background subtracted fluorescence (Fluo.) of *E. coli* cells expressing chimeric protein halves containing counterpart inteins split at the three different sites (S1, S2, and S3), normalized to the cell density (OD_600_). Bars represent the mean of three biological replicates (*n* = 3) and error bars correspond to s.d. The Western blot image (WB, bottom) shows the region corresponding to the spliced mCherry (Western blot full images can be found in Supplementary Fig. 7). The red signal corresponds to the antibody recognizing the N-terminal of mCherry and the turquoise signal corresponds to the antibody recognizing the hexahistidine tag (H_6_) at the C-terminus. The overlap of both signals (white) corresponds to the spliced reporter proteins. **c** Characterization of a 24 × 24 orthogonality matrix of the different split inteins (left). On the right, a pruned matrix showing the mutually orthogonal set of split inteins is depicted. Color gradient represents the background subtracted fluorescence (Fluo.) of *E. coli* cells expressing the split mCherry-split intein N- and C-terminal chimeric proteins, normalized to the cell density (OD_600_). Elements of the same intein split at different sites are boxed within the matrix. Fluo./OD_600_ values for each combination are shown in Supplementary Data 1. Data represents mean values of three biological replicates (*n* = 3). * Inteins with a flexible linker at the canonical split site. Source data are provided as a Source Data file.

Subsequently, we aimed to characterize all the functional inteins as split proteins, as *trans*-splicing applications are broader and transversal to different fields of research. Although naturally split inteins found in nature or inteins that are artificially split at the canonical endonuclease insertion site are predominantly used for this purpose, some inteins can be artificially split at atypical split sites, producing short N- or C-terminal peptides (<20 amino-acid residues in length). The latter are of particular interest for protein engineering since they can be fused to a protein of interest with no or minimal structural interference or can be obtained by peptide synthesis, thus allowing protein semisynthesis^27–29^. Therefore, to identify additional split sites, we performed a structural alignment of the 20 inteins exhibiting *cis*-splicing activity, and inferred additional atypical split sites from those previously reported^27^ (Supplementary Fig. 6). For each intein, three split sites were selected: split site S1, at the N-terminal end; split site S2, at the natural split site or the canonical endonuclease insertion site; and split site S3, at the C-terminal end (Fig. 2a and Supplementary Table 4).

We tested the *trans*-splicing activity of all the 60 intein pairs using the split mCherry platform in a two-plasmid system (Fig. 2a) and then optimized signal output for some inteins by tuning the C-termini RBS strength or by adding an expressivity tag (see Methods). Our results show that 24 split intein pairs produced substantial fluorescence levels and in all cases this was owing to mCherry *trans*-splicing, as confirmed by Western blot (Fig. 2b and Supplementary Fig. 7). Albeit all chimeric proteins were being expressed, for some split inteins one of the precursors was expressed at low levels, which may have limited the splicing reaction (Supplementary Fig. 7). Moreover, the *trans*-splicing efficiencies of NpuSsp split at sites S1 and S3, and of TerThyX split at site S1 could be significantly improved by adding a flexible linker at the canonical split site (Supplementary Fig. 8). We showed that 16 different inteins displayed *trans*-splicing activity and that six of them were active even when split at atypical sites (Fig. 2b).

To determine whether all split intein pairs could be used simultaneously in the same application we tested their orthogonality by measuring the fluorescence of *E. coli* cells co-expressing the 576 possible combinations of each N- and C-terminal chimeric protein halves (24 N × 24 C) (Fig. 2c). In this matrix, each split intein has 46 non-cognate combinations and their fluorescence values were then normalized to that of the cognate pair. We considered the non-cognate interactions negligible if all those relative values were below 0.2 and the median of those values was below 0.05. Out of the 16 inteins tested, 15 had negligible cross-interactions with other inteins and were thus considered orthogonal. When relative fluorescence above 0.2 was detected, in most cases, it was associated with combinations of inteins split at atypical sites, where one of the intein segments is a short peptide (Fig. 2c). Cross-reactivity within the same intein split at different sites was also observed in most cases, namely when there was sequence overlap between the N- and C-termini (Fig. 2c, boxed squares in left panel). Based on these results, we defined a set of 15 mutually orthogonal split inteins to be used for in vivo applications (Fig. 2c, right panel).

### Intein *trans*-splicing in vitro

We next sought to assess the in vitro performance of the 24 pairs of functional split inteins characterized in vivo. Inteins have been widely used in protein engineering, but most studies report the use of only a single intein per application and the reaction conditions vary among different reports^15,18,28,30–32^.

We first evaluated the expression of the 48 individual chimeric proteins and showed that their levels and solubility vary greatly depending on the intein portion fused to the split mCherry halves (Supplementary Fig. 10). We were able to improve the expression of the two chimeric proteins with the lowest expression levels in *E. coli* TOP10 (containing M86^C2^ and NpuSsp^C2^) by changing the expression plasmid and strain (Supplementary Fig. 10a). Mild denaturing conditions were used to extract and obtain all proteins in the soluble fraction (Supplementary Fig. 11).

**Fig. 3:**
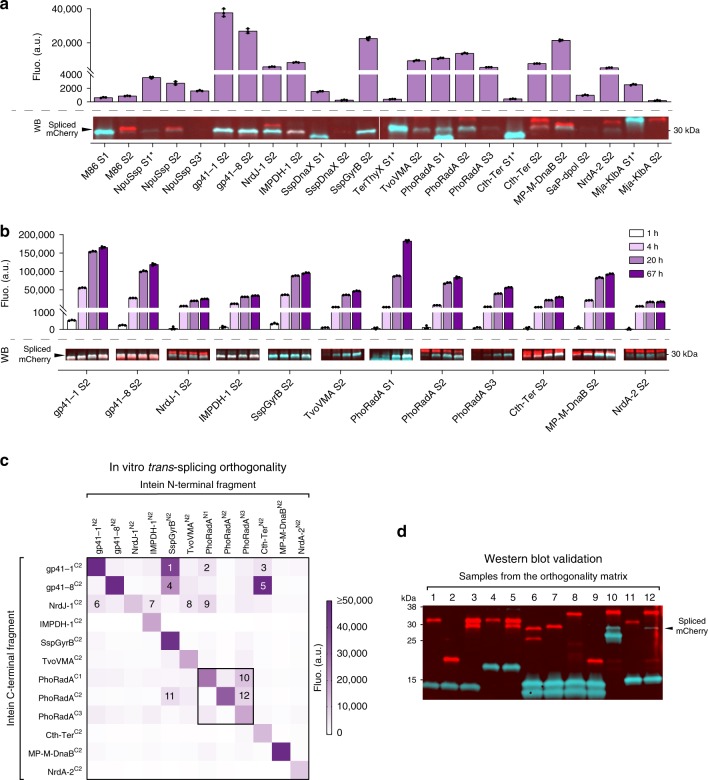
In vitro *trans*-splicing of split inteins and orthogonality characterization. **a** End-point fluorescence (Fluo., top) and Western blot analysis (WB, bottom) of the in vitro *trans*-splicing reactions carried out for 20 h, with mixed lysates of *E. coli* cells expressing complementary split mCherry-intein chimeric proteins (over time measurements can be found in Supplementary Fig. 13 and full WB image can be found in Supplementary Fig. 14). * Inteins with a flexible linker at the canonical split site. **b** Fluorescence (Fluo., top) and Western blot analysis (WB, bottom) of the in vitro *trans*-splicing reactions carried out with the most active split intein pairs as observed in **a**. Fluorescence was monitored for 67 h and measurements for 1, 4, 20, and 67 h of incubation are shown; samples for WBs were collected at the same time points (over time measurements can be found in Supplementary Fig. 15 and full WB images can be found in Supplementary Fig. 16). Bars in **a** and **b** represent the mean of three independent replicates (*n* = 3) and error bars correspond to s.d. **c** Characterization of a 12 × 12 orthogonality matrix of the most active split intein pairs. Color gradient represents the end-point fluorescence (Fluo.) of the in vitro *trans*-splicing reactions carried for 20 h. Elements of the same intein split at different sites are boxed within the matrix and the numbers correspond to samples with unexpectedly high fluorescence that were collected for further splicing evaluation. Fluo. values for each combination represent the mean values of three independent replicates (*n* = 3) and are shown in Supplementary Data 2. **d** Western blot validation of splicing for samples exhibiting unexpectedly high fluorescence. Lane numbers correspond to the elements numbered within the orthogonality matrix **c**. For all Western blots, the red signal corresponds to the antibody recognizing the N-terminal of mCherry and the turquoise signal corresponds to the antibody recognizing the hexahistidine tag (H_6_) at the C-terminus. The overlap of both signals (white) corresponds to the spliced reporter proteins. The proteins’ theoretical molecular weights can be found in Supplementary Table 9. Source data are provided as a Source Data file.

To enable using multiple inteins to achieve modular assembly of multiple peptides in one go, we used the split mCherry platform to determine the best common reaction conditions that would allow for the simultaneous use of any combination of inteins in vitro and to test their orthogonality. To this end, we performed a large-scale in vitro screening on *trans*-splicing reaction conditions and found that the conditions of room temperature (≈21 °C) and a reaction buffer comprising 100 mM Tris-HCl at pH 9.0, 100 mM NaCl, and 2 mM dithiothreitol (DTT), were optimal for the simultaneous use of intein combinations (Supplementary Fig. 12 and Supplementary Data 3). Using these reaction conditions we compared the in vitro *trans*-splicing activity of the 24 split intein pairs previously characterized in vivo. The results showed that 12 combinations, corresponding to 10 different inteins, exhibited fluorescence levels above 4000 a.u. and levels of spliced mCherry above 10% of the maximum achievable with the most efficient inteins (Fig. 3a and Supplementary Figs. 13, 14). We noted that 5 inteins (gp41-1, gp41-8, NrdJ-1, IMPDH-1, and SspGyrB, all split at site S2) were exceptionally fast, reaching over 75% of the maximum spliced product formation within 1 h of reaction, whereas for others this was attained by a longer incubation time (Fig. 3b and Supplementary Figs. 15, 16). The time lag observed between splicing and fluorescence detection is likely owing to delay caused by mCherry maturation.

Finally, we evaluated the in vitro orthogonality of this set of 12 split intein pairs and, using the same criteria from the in vivo orthogonality study, we showed that eight inteins exhibited low mutual cross-interaction in vitro (Fig. 3c and Supplementary Fig. 9b). Further analysis of the samples displaying unexpectedly high fluorescence levels showed that spliced mCherry could only be detected for N- and C-termini combinations of PhoRadA split at different sites (Fig. 3d). For the remaining, detected fluorescence is likely owing to non-specific electrostatic interactions between the split intein halves.

Altogether, the results obtained confirm that all the 10 split inteins tested are orthogonal in vitro and can be used simultaneously in protein engineering applications.

### Orthogonal split inteins enable complex biological circuits

**Fig. 4:**
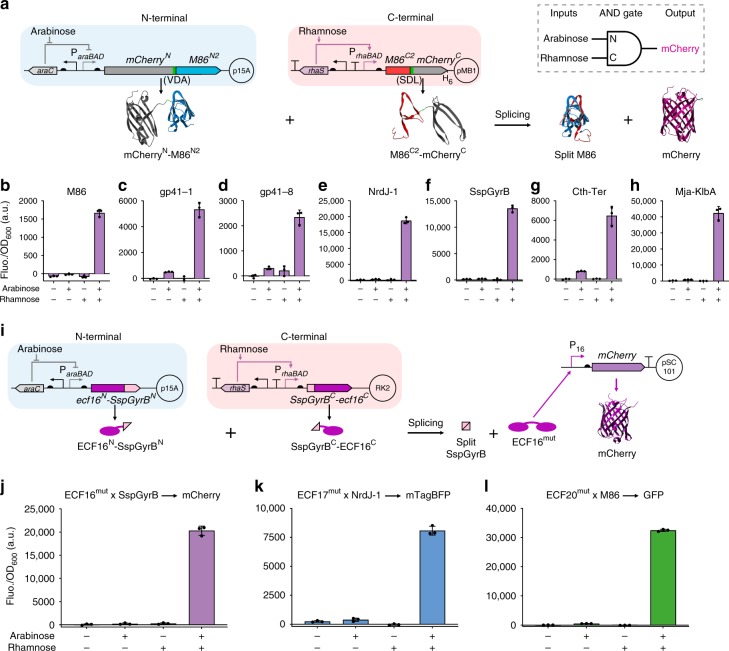
Design and characterization of split intein-enabled logic AND gates. **a** Genetic circuits for the arabinose- and rhamnose-induced expression of split mCherry-intein N- and C-terminal chimeric proteins, respectively, from two different plasmids (top, left), and schematics of protein *trans*-splicing, exemplified using M86 split at site S2 (bottom). The AND gate behavior (top, right) is achieved only when both inducers are present. Promoters are represented by straight angle arrows and RBS by black semi-circles. Split mCherry halves are shown in gray, M86 intein N-terminal in blue, M86 intein C-terminal in red, the added junction sequence residues (VDA and SDL) in green and the spliced mCherry in purple. **b**–**h** Background subtracted fluorescence (Fluo.) of *E. coli* cells harboring the genetic circuits for split mCherry-intein chimeric proteins, in the absence (−) or presence (+) of inducers. **i** Genetic circuits for the arabinose- and rhamnose-induced expression of split ECF^mut^-split intein N- and C-terminal chimeric proteins, respectively, from two different plasmids (top, left), and schematics of protein *trans*-splicing, exemplified using ECF16^mut^ and the SspGyrB intein (bottom). Split ECF16^mut^ halves are shown in dark pink and SspGyrB halves in light pink. After splicing, the reconstituted ECF16^mut^ can activate its cognate promoter (P_16_) and the fluorescent reporter is expressed. **j**–**l** Background subtracted fluorescence (Fluo.) of *E. coli* cells harboring the genetic circuits for the expression of the chimeric split ECF16^mut^ × SspGyrB halves and expression of the mCherry reporter from P_16_ (**j**), the chimeric split ECF17^mut^ × NrdJ-1 and expression of the mTagBFP reporter from P_17_ (**k**) or the chimeric split ECF20^mut^ × M86 and expression of the GFP reporter from P_20_ (**l**), in the absence (−) or presence (+) of inducers. In all the cases, fluorescence was measured 6 h after induction and it was normalized to the cell density (OD_600_). Bars in **b**–**h** and **j**–**l** represent the mean of three biological replicates (*n* = 3) and error bars correspond to s.d. Source data are provided as a Source Data file.

It has been shown that split inteins are useful tools to implement cellular logic operations^11,33–36^. We investigated whether alternative inteins could also be used for this purpose by building two-input logic AND gates where the two split mCherry-split intein halves were under the control of different inducible promoters (Fig. 4a). All seven intein-based gates tested exhibited the expected AND logic behavior, as high fluorescence levels could be detected only when expression of both chimeric protein halves was induced (Fig. 4b–h and Supplementary Fig. 17). The AND behavior of three gates was further improved by reducing the strength of the RBS controlling C-terminal translation, since leakiness was observed when using a stronger one (Supplementary Fig. 18). Thus, the seven split inteins tested were validated to be able to implement Boolean logic computation in vivo^37^.

We proceeded to couple orthogonal split inteins with orthogonal split extracytoplasmic function (ECF) sigma factors to build modular logic AND gates that can be wired to build complex logic circuits^38^. ECF sigma factors are the smallest and simplest alternative sigma factors, comprising only the two domains that bind the promoter −10 and −35 regions, and are separated by a disordered linker region. Thus, we split three orthogonal ECFs^39^ at the linker region and confirmed their loss of function (Supplementary Fig. 19). We then sought to incorporate native extein junction sequences into those split sites, which should maximize splicing efficiencies of inserted split inteins. To that end, we mutated the linker region of these three ECFs to accommodate the native junction sequences (6 AAs, −3 to +3) of the seven inteins previously tested for AND gate behavior (Fig. 4b–h). This mimicked the spliced protein products and allowed assessing the consequent ECF activity impairment. Although all mutated ECFs (ECF^mut^) had some decrease in transcriptional activation, they remained functional, with no clear correlation between the mutations and the extent of impairment (Supplementary Fig. 20). Subsequently, we investigated whether the activity of intein split ECF^mut^ proteins could be reconstituted by intein-mediated *trans*-splicing. We built three intein split ECF AND gates, namely ECF16^mut^ × SspGyrB, ECF17^mut^ × NrdJ-1 and ECF20^mut^ × M86 (Fig. 4i–l). We selected these three split inteins because they exhibited the lowest leakiness levels (Fig. 4b–h) and their native junction sequences have limited effects on the respective ECF activity (Supplementary Fig. 20). Furthermore, we show that the three ECF^mut^ corresponding to these three inteins are still orthogonal (Supplementary Fig. 21). The output fluorescence of these devices was detected only when both chimeric ECF halves were expressed (Fig. 4j–l and Supplementary Figs. 22–24), confirming their logic AND gate functionality. To rule out the possibility that split intein complex interaction without splicing sufficiently reconstitute ECF activities, we inactivated the inteins by mutating catalytic residues and showed that the ECF activities were abolished (Supplementary Fig. 25). Interestingly, we noted that the background leakiness of ECF16^mut^ and ECF20^mut^ was significantly reduced by splitting the sigma factors, resulting in higher output dynamic ranges despite reduction of the maximum output levels.

**Fig. 5:**
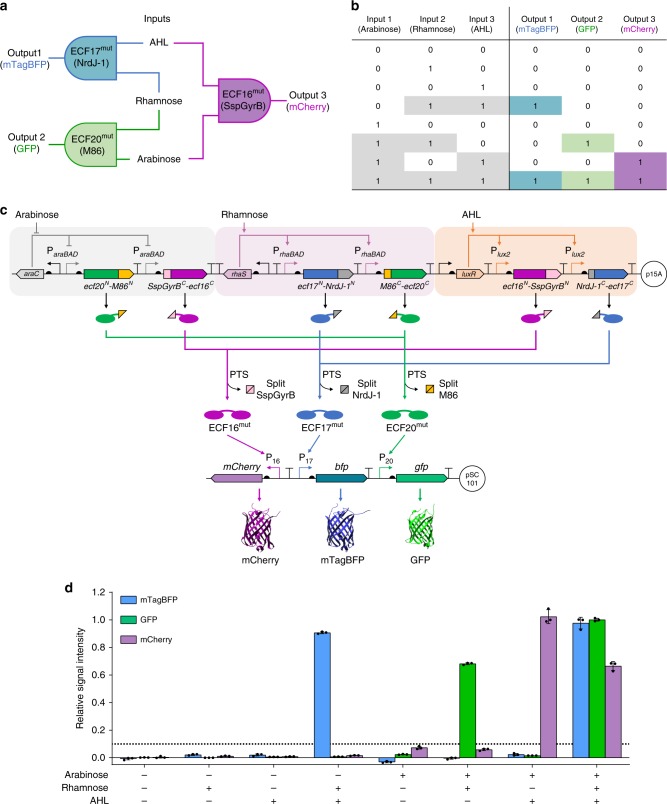
Integrated intein-based logic circuit for selective detection of multi-input combinations. **a**–**c** The schematic (**a**), truth table (**b**), and genetic design architecture (**c**) of the three-input three-output integrated logic circuit. Each input induces the expression of non-complementary split ECF-split intein N- and C-terminal chimeric protein proteins. An output is produced in the presence (+) of two inputs and in the absence (−) of the third input, via ECF *trans*-splicing and subsequent cognate promoter activation, but not when the system is exposed to only one input or in the absence of all inputs. The presence of the three inputs results in full activation of the system and the three outputs are expressed. **d** Relative fluorescence intensities of *E. coli* cells carrying the integrated logic circuit, measured 8 h after cultures exposed to the eight possible input combinations, normalized to maximum output signal of each reporter (see Supplementary Fig. 26). Bars represent the mean of three biological replicates (*n* = 3), error bars correspond to s.d. and the horizontal dotted line denotes 10% of the maximum signal output. Source data are provided as a Source Data file.

Finally, we connected the three AND gates to build a three-input three-output integrated logic circuit (Fig. 5a). The circuit produces fluorescent output under the conditions of having two activated inputs or above and discriminates between them by reporting a corresponding fluorescent protein (Fig. 5a, b). The circuit is designed by having each input promoter controlling the expression of a pair of unrelated intein split ECF halves (Fig. 5c). The characterization results showed that the circuit exhibited the expected logic behavior under the induction of eight logic combinations of the three inputs (arabinose, rhamnose, and AHL, Fig. 5b): high blue fluorescence output only when rhamnose and AHL were present; green, with arabinose and rhamnose; red, with arabinose and AHL; when all three inducers were present, all three-output reporters were high; and when only one or none of the inducers were present, the fluorescent levels of all output reporters were considerably low (Fig. 5d and Supplementary Fig. 26). This application exemplifies the great potential of integrating orthogonal split inteins and split transcription factors to design complex cellular logic circuits, while limiting the number of regulatory elements used.

### Orthogonal split inteins enable modular protein assembly

To further demonstrate the versatility of our expanded library of orthogonal split inteins, we proposed another in vitro application to achieve rapid modular assembly of highly repetitive and elongated proteins. Recombinant expression of this type of proteins is typically difficult owing to the long size and repetitive nature of their coding sequences, which often leads to genetic instability, host toxicity, and obstacles to molecular cloning^40–42^. Recently, a split intein has been employed in vitro to assemble repetitive proteins and yield mechanically strong biomaterials^41,43^, and another example of such a protein with biotechnological potential is the *Staphylococcus aureus* surface protein G (SasG). SasG comprises tandem repeats that fold into two structurally related domains (E and G5) and forms extended rod-shaped fibrils with remarkable mechanical strength on the surface of the cells, promoting host adherence and biofilm formation^44–46^.

We first sought to produce elongated SasG-based proteins by conventional assembly of gene blocks encoding SasG5^3^E- repeats (G5-E-G5-E-G5). This task proved to be challenging and while we could clone up to SasG5^12^E^8^ (a 4.1 kb gene and 150 kDa protein), attempts to produce larger assemblies always resulted in partial deletions of the repetitive sequences. We then analyzed protein production in two different *E. coli* strains suitable for protein expression (BL21-Gold and Origami B) and, as expected, we observed that as the protein size increases the expression levels decrease, which was more obvious in Origami cells (Supplementary Fig. 27).

**Fig. 6:**
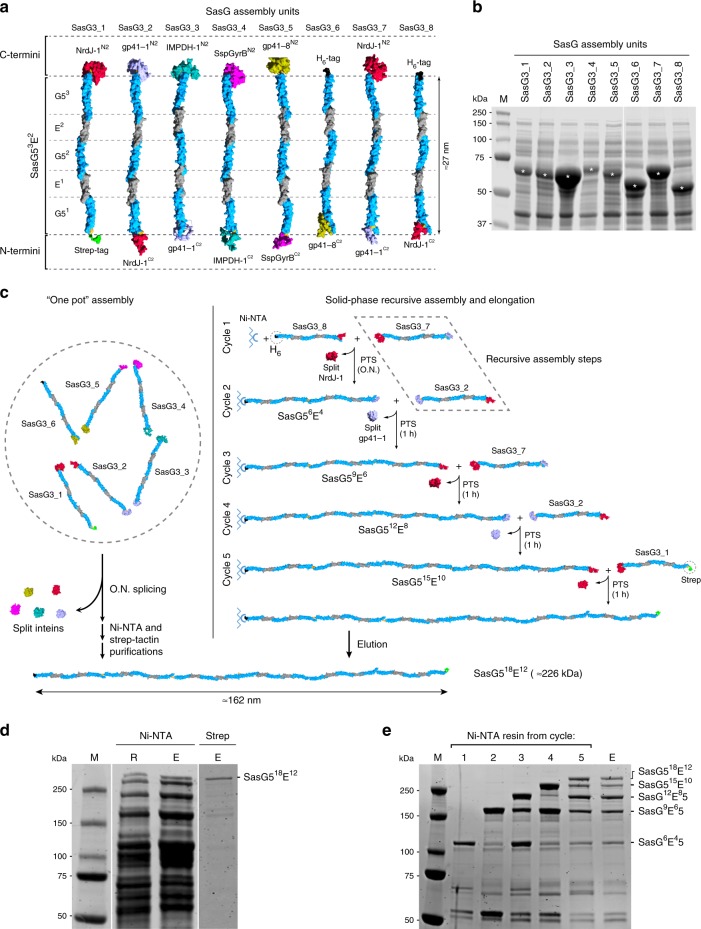
In vitro modular assembly of large and highly repetitive proteins based on SasG blocks. **a** Schematics of the protein assembly units showing SasG5^3^E^2^ fused to the split inteins or to purification tags (Strep-tag in green, hexahistidine (H_6_) tag in black). The approximate length of SasG5^3^E^2^ is shown for reference^47^. **b** SDS-PAGE analysis of *E. coli* BL21 clear lysates expressing the assembly units. The bands corresponding to each assembly unit are marked with “*” and the proteins’ theoretical molecular weights can be found in Supplementary Table 9. **c** Assembly approaches to produce SasG5^18^E^12^. In the “one pot” assembly (left), all the cell lysates containing the assembly units are mixed and reacted together overnight (O.N.) to allow for *trans*-splicing with consequent release of the split inteins. The assembled protein is then obtained after two purification steps using Ni-NTA resin followed by Strep-tactin resin. In the solid-phase recursive assembly, the lysates containing the H_6_-tagged unit and following assembly unit are incubated overnight with the resin to allow for protein binding to the Ni-NTA matrix and *trans*-splicing of the first two units. Subsequent washing steps are performed and the following assembly unit is mixed with the resin and incubated for 1 h to allow for *trans*-splicing. Cycles of washings and 1 h incubations with the following assembly unit are repeated until the desired product size is obtained and the final protein is eluted after the final washing step. **d** SDS-PAGE analysis of the resin (R) and eluate (E) from the Ni-NTA purification of the “one pot” assembly and eluate (E) after the Strep-tactin purification (Strep). The full-length final protein is indicated (SasG5^18^E^12^). **e** SDS-PAGE analysis of the Ni-NTA resin after each cycle of recursive solid-phase assembly (1–5) and final protein elution (E). The full-length final protein and other side products resulting from incomplete splicing reactions are indicated. *M* molecular weight marker. Protein gel images are representative of at least two independent experiments with similar results. Source data are provided as a Source Data file.

We next used our library of orthogonal split inteins to assemble elongated repetitive SasG proteins in vitro, using protein blocks of SasG5^3^E^2^ repeats fused to different split inteins. To this end, we followed two strategies to assemble up to six SasG5^3^E^2^ repetitive units (SasG5^18^E^12^, c.a. 225 kDa): (i) “one pot” assembly using five orthogonal inteins (gp41-1, gp41-8, NrdJ-1, IMPDH-1, and SspGyrB), requiring six different building units; and (ii) recursive solid-phase assembly using two orthogonal inteins (gp41-1 and NrdJ-1), requiring four different building units (Fig. 6). To achieve high splicing activity, we included the residues preferred for each intein at the SasG-intein interface. Purification tags were added to the terminal SasG units to facilitate product separation from the other *E. coli* proteins present in the lysates and to enable the recursive solid-phase assembly of protein units using nickel-nitrilotriacetic acid (Ni-NTA) resin. The results showed that these chimeric proteins can be readily produced in *E. coli* as soluble products (Fig. 6b) and that reacting the units with the respective split intein counterparts resulted in the formation of the desired spliced products, but not when units with different split inteins were reacted together, further attesting the orthogonality of the inteins used (Supplementary Fig. 28). Notably, SasG proteins run at an apparent molecular mass higher than its theoretical molecular mass, a feature characteristic of many cell wall-associated proteins. Our results show that reacting the six complementary SasG units altogether in “one pot” successfully produced a protein with the expected size (c.a. 225 kDa) that was obtained with relatively high purity after two affinity purification steps, using the different tags attached to each terminus (Fig. 6d). The recursive solid-phase approach required less assembly units, as the extracts used were the same every other cycle. By monitoring protein splicing at each elongation cycle on a protein gel, we could clearly spot a “ladder” pattern corresponding to the growing peptide, which resulted in the formation of a protein of the expected size following the fifth reaction cycle (Fig. 6e). As anticipated, smaller by-products resulting from incomplete splicing reactions can be seen and were shifted to higher molecular weights when they reacted with fresh extracts at each new cycle. Strikingly, by increasing the number of assembly cycles to nine, we were able to show the production of even much longer proteins (up to SasG5^30^E^20^, c.a. 377 kDa; Supplementary Fig. 29).

This work shows the vast potential of our expanded library of orthogonal split inteins for in vitro applications and demonstrates a rapid and simple way to seamlessly assemble long and repetitive proteins under mild conditions. It is worth noting that these proteins would otherwise be difficult to produce using heterologous expression systems.

## Discussion

Inteins have been referred to as “nature’s gift to protein chemists”^1^ and are remarkable tools for both protein engineering and synthetic biology applications. Indeed, the self-catalytic nature of the splicing reaction relying solely on protein folding and the fact that inteins are able to ligate two peptides without being part of the final product, are characteristics of exceptional value. However, the requirement for specific extein sequences at the site of insertion may constitute a limitation to the use of inteins in protein sequences that cannot tolerate amino-acid residue additions or substitutions. Considering the remaining limitations of using the few engineered promiscuous inteins that are able to tolerate non-native junction sequences^14–16^, we aimed to identify an expanded library of active and orthogonal split inteins, not only to increase the chance of compatibility with the proteins of interest but also to be used concurrently within the same application.

We developed a new split mCherry fluorescent reporter platform for the rapid assessment of inteins functionality and orthogonality, which allowed us to perform a comprehensive characterization of 34 selected inteins, including uncharacterized ones. We further demonstrated the power of our split reporter platform to screen optimal conditions for in vitro intein *trans*-splicing. By testing over 1000 samples in a single day, we were able to rapidly define a one-for-all inteins splicing condition. This would have been impractical to do using conventional methodologies, such as Western blot. Although existing characterization platforms, which are based on cell survival coupled to Western blotting analysis may allow for the directed evolution of inteins^15^, ours provide a faster and more direct method for intein performance comparison. Although with some possible limitations, such as different protein expression levels, the effect of mutations on reporter intrinsic fluorescence and the occasional need for confirmation by other molecular techniques, we envisage that this fluorescent reporter platform has a broad potential and will be useful for many other purposes, such as screening intein tolerance to non-native junction residues or further improving splicing reaction conditions.

We showed the in vivo *trans*-splicing activity of 24 pairs of inteins split at canonical and atypical sites and found 15 of them to be highly orthogonal. To our knowledge, this is the largest set of orthogonal split inteins reported so far. This expanded library increases the present options available for the simultaneous use of multiple split inteins within a single reaction system. To demonstrate potential applications enabled by this library, we coupled orthogonal split inteins with split orthogonal transcriptional regulators to implement complex logic in living cells. The nearly perfect behavior achieved in the engineered system shows the tremendous potential of using split inteins to build scalable logic circuits in synthetic biology applications^47^. Moreover, the use of split inteins in genetic logic systems reduces, at least by half, the number of controlling regulatory elements required and thus increases design space. We expect multiple orthogonal split inteins can also play important roles in other applications, such as for the ordered assembly of metabolic proteins to enhance pathways flux. Although other methodologies such as leucine zippers or SpyTag/SpyCatcher could be used for similar purposes, only a limited number of their orthogonal elements are available so far and their use is application dependent. For instance, the advantage of using split inteins over those systems was illustrated in a recent study of gene expression regulation using a bipartite transcription factor^36^. In particular, split inteins are powerful alternatives to other technologies where the assembling peptides become part of the final reconstituted product, reinforcing the value of the expanded library of orthogonal split inteins identified herein.

Out of the 16 split inteins that are functional in vivo, only 10 exhibit substantial *trans*-splicing activity in vitro while remaining catalytically orthogonal. This difference may be attributed to the change in the reaction environment or to the improper folding of individual chimeric proteins that were expressed and purified independently and subsequently combined. Similar to previously reported^27,48^, we found that chimeric proteins solubility is affected by the intein segment fused. However, we provided evidence that total protein extraction under mild denaturing conditions results in higher yields of soluble chimeric proteins, while retaining intein activity. The strategy could be useful to extract other difficult-to-express intein-fused proteins.

Finally, using multiple orthogonal split inteins fused to SasG-derived assembly units, we demonstrated in vitro multi-peptide assembly of large and highly repetitive proteins at a small scale. By increasing the size of the assembly units and scaling up the amounts of protein and resin used, it would allow the production of even larger proteins with higher yields. Notably, our work describes a simple and fast method for the modular assembly of multiple peptides with the advantage that no intermediate purification steps are required, nor do the protein assembly units have to be purified in advance. We foresee our expanded orthogonal intein library will be applied to produce other similar large repetitive proteins of biotechnological interest and to produce single-chain protein nanostructures^13^ with higher complexity.

In order to use engineered split inteins in particular applications, one must bear in mind that protein splicing efficiency is not only dependent on reaction conditions but also depends considerably on the junction sequences and on the exteins themselves^27^. In addition, protein expression levels and solubility of the chimeric (split) proteins of interest fused to split inteins also play a key role in the overall splicing efficiency^27^. Any of these factors can turn an otherwise highly efficient intein into a poor protein ligation tool and this makes activity prediction challenging. Our work addresses this issue by offering a catalog of well-characterized and benchmarked orthogonal split inteins with demonstrated versatility, which increases the design space and hence the chances of success in intein applications. These inteins have the potential to be scalable tools for genetic circuit design, protein engineering and biomaterial manufacturing.

## Methods

### Intein selection, design, and synthesis

The selection of split inteins previously described in the literature was performed by taking into account splicing reaction rates and efficiencies, demonstrated activity in *E. coli* and putative orthogonality. For these inteins, the sequences for the N- and C-termini were directly fused into a single gene. Three other inteins described in the literature were also considered for our analysis: NrdA-2, which was previously identified by metagenomics but not characterized^23,49^; PfuRIR1-1 that was shown to work *in trans* when containing the endonuclease domain^24,50^ and MjaKlbA, that was shown to work *in cis* but not tested *in trans*^25,51^. To select additional uncharacterized inteins, “The Intein Database and Registry”^5^ (accessed on 6th November 2017) was searched for the keywords “virus” and “phage”. Subsequently, the protein sequences retrieved were aligned to identify identical inteins (from different hosts), and a motif search was performed on the sequences putatively harboring a homing endonuclease domain to delimitate its sequences. The “MOTIF” resource from GenomeNet (https://www.genome.jp/) was used for this purpose, selecting all the motif libraries/databases available for the search. In addition, the presence of identical Intein Motif Consensus Sequences was assessed to avoid duplication and possible crosstalk between different split inteins. To design the synthetic genes, the identified homing endonuclease domains were replaced by a flexible linker^52^ (GSAGSAAGSG) to allow structural flexibility and proper folding of the proteins *in cis*. To determine the N- and C-terminal portions of the inteins without homing endonuclease domains, a predictive structure-based alignment was performed using the PROMALS3D tool^53^. Alignment to tree format conversions were performed using BioNJ and the phylogenetic trees were constructed using TreeDyn 198.3 (both available at the https://www.phylogeny.fr/ server^54^). The same flexible linker was added between the identified N- and C-terminal portions of these inteins. The atypical split sites were identified based on the structural alignment and the sites previously described in the literature. When intein DNA sequences were available, the restriction sites for *Eco*RI, *Pst*I, *Spe*I, and *Xba*I were removed by introducing silent mutations. When no DNA sequences were available, protein sequences for gp41-1, gp41-8, NrdJ-1, and IMPDH-1 were back-translated using GeneOptimizer (Thermo Fisher), and the Gene Designer v2.0 software (DNA2.0, Inc.) was used for the remaining sequences. In all cases, restriction sites for *Eco*RI, *Pst*I, *Spe*I, and *Xba*I were avoided. DNA fragments for all the inteins were synthesized as GeneArt Strings (Thermo Fisher). Supplementary Table 3 lists the protein and DNA sequences synthesized.

### Strains and standard growth conditions

Plasmid cloning work was all performed in *E. coli* TOP10 strain (Invitrogen). *E. coli* BL21-Gold(DE3) (Stratagene) and Origami B(DE3)pLysS (Merk) cells were also used as indicated. Cells were routinely cultivated in LB (lysogeny broth) medium (10 g L^−1^ tryptone, 5 g L^−1^ yeast extract, 5 g L^−1^ NaCl). Antibiotics were used when required at final concentrations of 10 μg mL^−1^ of tetracycline (T8032, Sigma-Aldrich), 50 or 100 μg mL^−1^ of ampicillin (A9518, Sigma-Aldrich) and 50 μg mL^−1^ of kanamycin (K4000, Sigma-Aldrich). Cells inoculated from single colonies were grown overnight in 5 mL of LB in sterile 30 mL universal tubes at 37 °C with shaking (160 rpm). When required, 0.83 mM of l-(+)-Arabinose (A3256, Sigma-Aldrich), 13.7 mM of l-(+)-Rhamnose (L5701, Promega), 10 μM of N-(3-Oxohexanoyl)-l-homoserine lactone (AHL) (K3007, Sigma-Aldrich) or 1 mM Isopropyl-β-d-thiogalactopyranoside (IPTG) (MB1008, Melford) were added to the growth medium.

### Preparation and assembly of DNA

DNA constructions were performed according to the principles of Gibson assembly^55^ or using the BioBrick RFC[10] standard^56^, following standard molecular biology techniques. For the BioBrick RFC[10] standard FastDigest *Eco*RI, *Bcu*I (*Spe*I), *Pst*I, and *Xba*I enzymes were used (FD0274, FD1253, FD0614, and FD0684, respectively; Thermo Fisher). DNA fragments were generated by polymerase chain reaction (PCR) amplifications using primers with ≥18 bp overlaps. All PCR amplifications were performed using Phusion high-fidelity DNA polymerase (F530, Thermo Fisher) and performed on a ProFlex 3 × 32-well PCR System (4484073, Thermo Fisher). The oligonucleotides used in this study were synthesized by Sigma-Aldrich or Integrated DNA Technologies and are listed in Supplementary Table 5. The regulatory parts used in this study are listed in Supplementary Table 6 and the plasmids used as templates are listed in Supplementary Table 7. Detailed methods for constructing the plasmids used, listed in Supplementary Table 8, are described in Supplementary Note 1 and their sequences can be found in Supplementary Data 4.

### Bacterial transformations for in vivo assays

The procedure for bacterial transformation with one, two or three plasmids was adapted from Rhodius et al.^39^. In brief, 5–10 ng of each plasmid was incubated in PCR tubes or PCR plates with 10 μL (for one or two plasmids) or 50 μL (for three plasmids) of competent cells and incubated on ice for 30 min. Heat shock was performed for 45 s at 42 °C in a PCR machine, followed by incubation on ice for 2 min. Cells were then diluted in fresh LB to a final volume of 200 μL, transferred to 96-well plate(s) (CC7672-7596, Starlab) and incubated on a plate shaker (AS-03020-00, Allsheng) at 37 °C and 1000 rpm, for 1–2 h. After this period, 10 μL (for one or two plasmids transformation) or 30 μL (for three plasmids transformation) of culture was transferred to three independent wells containing up to 190 μL LB supplemented with the appropriate antibiotics to generate the three biological repeats. Plates were covered with Rayon breathable films (Z380059, Sigma-Aldrich) and incubated overnight at 37 °C and 1000 rpm. As reported^39^, this liquid selection in the presence of antibiotics was sufficient to prevent growth of no plasmid controls. Cultures grown to saturation were used for downstream assays and for growing fresh cultures as required.

### In vivo fluorescence measurements

All in vivo assays were performed with three biological replicates and cells transformed with appropriate empty backbone plasmids were included as negative controls. Unless stated differently, overnight cultures were diluted 100-fold into 96-well microplates (655096, Greiner Bio-One) containing 198 μL of LB medium supplemented with the appropriate antibiotics and inducers. Microplates were sealed with breathable films and incubate for 6 h on plate shakers at 37 °C and 1000 rpm. End-point fluorescence for mCherry (excitation filter: 584 nm; emission filter: 620–10 nm; gain = 2000), GFP (excitation filter: 485 nm; emission filter: 520–10 nm; gain = 1000) and mTagBFP (excitation filter: 405–10 nm; emission filter: 460 nm; gain = 1000), and culture optical density at 600 nm (OD_600_) were measured using Omega Control v5.11 (BMG Labtech) in a FLUOstar Omega plate reader (BMG Labtech) and data was analyzed using Omega MARS Software v3.32 (BMG Labtech). The LB medium background fluorescence and absorbance were subtracted from the readings of sample wells and fluorescence/OD_600_ (Fluo./OD_600_) was calculated for all samples. Normalized data were produced by subtracting the negative control Fluo./OD_600_ from the other samples and exported to GraphPad Prism v8.1.2 or Microsoft Excel 2013 for further analysis.

For mCherry insertion variants characterization and inteins *cis*- and *trans*-splicing evaluation, overnight cultures were diluted 100-fold into 96-deepwell plates (E2896-2110, Starlab) containing 1 mL of fresh LB medium supplemented with 0.83 mM arabinose and incubated at 37 °C and 1000 rpm. Five hours after induction, chloramphenicol was added to a final concentration of 100 μg mL^−1^ to stop translation and the cultures were re-incubated under the same conditions for another 2 h, to allow further splicing and full mCherry maturation. Subsequently, 200 μL of each culture was transferred to a 96-well microplate and mCherry fluorescence and cell density were measured and analyzed as described above.

For the three-input/three-output circuit characterization, cells carrying only the three-reporter plasmid were used for data normalization as a negative control. The bacterial cultures were grown in a 96-well microplate covered with a lid (656171, Greiner Bio-One) to prevent evaporation and incubated inside the plate reader at 37 °C and 700 rpm. Fluorescence and OD_600_ were measured every 20 min, for a period of 20 h. Detector gains were adjusted to 1200 and 900 for mTagBFP and GFP, respectively, to provide output signals comparable to those of mCherry. Data was further analyzed as described above.

When required, 200 μL of the cultures from each corresponding biological replicate were mixed (600 μL), spun down for 1 min at 17,000 × *g* and resuspended in 50 μL of 1× Laemmli sample buffer (1610747, Bio-Rad) for Western blot analysis.

### SDS-PAGE and Western blots

For protein expression and inteins splicing evaluation, samples in 1× Laemmli buffer were boiled for 10 min and spun down for 10 min at 17,000 × *g*. Routinely, 10–15 μL of sample was separated in Any kD TGX Stain-Free protein gels (4568126, Bio-Rad) or 4–20% TGX stain-free protein gels (4568095, Bio-Rad). Proteins were stained with Bio-Safe Coomassie Stain (1610786, Bio-Rad) or transferred to nitrocellulose membranes (1704270, Bio-Rad) using the Trans-Blot Turbo Transfer System (1704150, Bio-Rad), according to the manufacturer’s instructions. Precision Plus Protein Dual Xtra Prestained Protein Standards (1610377, Bio-Rad) and Chameleon Duo Prestained Protein Ladder (928-60000, Li-cor) were used for Coomassie stained gels or Western blots, respectively. Western blots and near-infrared detection were performed as described in Li-cor “Near-Infrared Western Blot Detection document” (Doc. #988-13627), using 5% (w/v) skimmed milk in 1× tris-buffered saline (1706435, Bio-Rad) for blocking. Antibodies recognizing residues 27–41 of the mCherry (Anti RFP-tag, pAb, Rabbit; A00682, GenScript) or the hexahistidine tag (THE His Tag Antibody, mAb, Mouse; A00186, GenScript) were used as primary antibodies at dilutions of 1:3000 and 1:5000, respectively. IRDye 680RD Goat anti-Rabbit (925-68071, Li-cor) and IRDye 800CW Goat anti-Mouse (925-32210, Li-cor) were used as secondary antibodies at dilutions of 1:20,000. Stain-free images of TGX stain-free protein gels were acquired and analyzed using Image Lab v5.2 (Bio-Rad). Coomassie stained protein gels and blotted membranes were visualized using an Odyssey CLx Infrared Imaging System (Li-cor) and analyzed using the Image Studio Lite v5.2.5 software (Li-cor). The theoretical molecular weight for all proteins analyzed by SDS-PAGE or Western blot are described in Supplementary Table 9.

### mCherry standard expression and purification

For the purification of the His-tagged mCherry reporter, an overnight culture of *E. coli* TOP10 harboring the plasmid pFP.R193 was diluted 100-fold in 50 mL of Terrific Broth (TB) supplemented with tetracycline and arabinose. TB medium contained (per L): 12.0 g tryptone, 24.0 g yeast extract, 4 mL glycerol, 100 mL 0.17 M KH_2_PO_4_, and 100 mL 0.72 M K_2_HPO_4_. The culture was grown overnight at 37 °C with shaking (160 rpm) and cells were collected by centrifugation for 25 min at 4696 × *g* and resuspended in 3 mL of lysis buffer (50 mM NaH_2_PO_4_, 300 mM NaCl, pH 8.0, cOmplete Protease Inhibitor Cocktail (4693132001, Sigma-Aldrich)). Lysis was achieved by sonication at maximum output (Soniprep 150, MSE) for nine cycles of 10 s with a 10 s rest period between each cycle. Clarified cell lysates were obtained by centrifugation (20 min at 17,000 × *g*, 4 °C) and subsequently incubated overnight at 4 °C with 1 mL of pre-equilibrated Ni-NTA resin (30230, Qiagen), with gentle agitation to allow for binding. The resin was washed three times with 5 mL of washing buffer (50 mM NaH_2_PO_4_, 300 mM NaCl, 20 mM imidazole, pH 8.0) and mCherry was then eluted with four volumes of 1 mL of elution buffer (50 mM NaH_2_PO_4_, 300 mM NaCl, 250 mM imidazole). The eluted fractions were pooled and dialyzed against 1× PBS (k813, VWR) using centrifugal filter units with molecular weight cutoff of 3000 (UFC9003, Millipore). The concentrated sample was further purified by size exclusion chromatography using a Superdex 75 10/300 GL column (GE17-5174-01, GE). Eluted fractions were analyzed by SDS-PAGE and those containing mCherry were pooled and concentrated using a new centrifugal filter unit. Glycerol was added to the protein stock to a final concentration of 25% (v/v), for storage at −20 °C. Protein concentration was determined using the Pierce BCA Protein Assay kit (10678484, Thermo Fisher) and diluted to a working concentration of 25 μM, for comparative analysis in Western blots.

### In vitro characterization of mCherry insertion variants

For mCherry native protein and variants production, 5 mL of LB medium supplemented with tetracycline and arabinose were inoculated with *E. coli* cells carrying the appropriate plasmids and grown overnight at 37 °C (160 rpm). Cells were collected by centrifugation (5 min at 4696 × *g*) and resuspended in TE buffer (100 mM Tris-HCl, 1 mM EDTA), pH 8.0. Subsequently, 0.25 g of 425–600 µm glass beads (G8772, Sigma-Aldrich) were then added to the suspension and cells were disrupted by vortexing at maximum speed for 4 cycles of 5 min with 1 min rest period between each cycle. Clarified cell lysates were obtained by centrifugation (20 min at 17,000 × *g*, 4 °C) and target proteins concentrations were calculated by analyzing Western blot signal intensities and comparing them with known amounts of purified mCherry. Samples were diluted to 2.5 μM of target protein and a series of four twofold dilutions was prepared. In all, 20 μL of each sample was transferred in triplicate to a 384-well microplate (781906, Greiner Bio-One) and fluorescence (Fluo.) was measured on a plate reader (excitation filter: 584 nm; emission filter: 620–10 nm; gain = 2000). Fluorescence was blank corrected by subtracting the fluorescence measured for wells loaded with TE buffer. For each dilution, the fluorescence of each mCherry variant was normalized to that of the native protein and averaged for further analysis.

### Split mCherry-split intein chimeric protein extraction

For in vitro characterization, all chimeric proteins were expressed in 5 mL of TB medium. Protein expression in *E. coli* TOP10 was induced by supplementing the growth medium with 0.83 mM arabinose and growing the cultures overnight at 37 °C. Origami cells, were grown at 37 °C (160 rpm) for 3 h, subsequently induced with 1 mM IPTG and further grown overnight at 20 °C (200 rpm). For whole cell analysis, 500 μL of culture was spun down for 1 min at 17,000 × *g* and cells were resuspended in 50 μL of 1× Laemmli sample buffer and boiled for 10 min. In all, 10 μL of each sample was separated by SDS-PAGE to evaluate protein expression. To assess protein solubility, clear lysates were prepared as described for the mCherry variants and inclusion bodies were solubilized under mild denaturing conditions^57^. For this purpose, the cell debris was resuspended in 250 μL of extraction buffer (100 mM Tris-HCl, 2M urea, pH 12.5), incubated at room temperature for 30 min with gentle shaking and diluted with TE buffer (pH 8.0) to a final volume of 1 mL, correcting the pH to 8 with concentrated HCl. The tubes were incubated at room temperature for 10 min and centrifuged for 20 min at 4 °C (17,000 × *g*). Samples from clear lysates and solubilized inclusion bodies were separated by SDS-PAGE for further analysis. To prepare the extracts for in vitro reactions, whole cell proteins were extracted under mild denaturing conditions, by suspending the cell pellets in 250 μL of extraction buffer, adding 0.25 g of 425–600 µm glass beads and vortexing at maximum speed for four cycles of 5 min, with 1 min rest period between each cycle. The extracts were then incubated at room temperature for 30 min with gentle shaking and diluted to a final volume of 1 mL using dilution buffer (100 mM Tris-HCl, 1 mM EDTA, pH 9.0, cOmplete Protease Inhibitor Cocktail). The pH was corrected to pH 9.0 with concentrated HCl and the tubes were incubated at room temperature for 10 min. Clear lysates were obtained by centrifugation (20 min at 17,000 × *g*, 4 °C) and separated by SDS-PAGE for analysis and relative quantification. Western blots were further performed to identify and quantify the target proteins.

### Inteins *trans*-splicing assessment in vitro

To identify the best reaction conditions to allow for any intein combination, a large-scale in vitro screening was performed varying the pH (8 or 9) and NaCl concentration (100, 300, or 500 mM) of the buffer and incubating the reactions at different temperatures (4 °C, 21 °C (room temperature), 30 °C, 37 °C, and 42 °C). For this purpose, the amount of each chimeric protein in the clear lysates was estimated from Coomassie stained gels and equal amounts of matching N and C chimeric protein halves were mixed together in TE buffer (pH 9.0). In all, 20 μL of each mixture was consequently transferred to 96-well microplates containing 30 μL of TE buffer at pH 8.0 or pH 9.0 and the reactions were started by adding 20 μL of 2× reaction buffers containing DTT (443852A, VWR). In all, 2× reaction buffers were composed of 100 mM Tris-HCl, pH 8.0 or 9.0; 1 mM EDTA; 200, 600, or 1000 mM NaCl; and 4 mM DTT. Two replicate plates were prepared and sealed with breathable film, and the fluorescence was measured (*t* = 0). End-point mCherry fluorescence was measured after 13 h incubation at the different temperatures with gentle shaking. Normalized fluorescence was calculated by subtracting the values from *t* = 0 and averaging the values from two replicates.

Further in vitro characterizations were performed in triplicate using normalized amounts of all proteins, based on Western blot quantification using purified mCherry standards. To characterize the 24 split intein pairs, equal amounts of matching N and C chimeric protein halves were mixed together in TE buffer (pH 9.0). 100 μL of each mixture was consequently transferred to 96-well microplates and the reactions were started by adding 100 μL of 2× reaction buffer containing DTT (100 mM Tris-HCl pH 9.0, 1 mM EDTA, 200 mM NaCl, and 4 mM DTT). The plate was sealed with breathable film and incubated inside the plate reader at room temperature. mCherry fluorescence was measured every 5 min 24 s for 20 h and data were normalized by subtracting the lowest value measured for each well. Samples were taken at the end-point for Western blot analysis. The spliced products of 12 inteins exhibiting the highest fluorescence levels were further characterized by incubating the plates for 67 h and collecting samples for Western blot analysis at 1, 4, 20, and 67 h. In vitro orthogonality was assessed by mixing normalized amounts of each of the 144 combinations of N and C chimeric protein halves in TE buffer (pH 9.0). In all, 25 μL of each mixture was transferred to 96-well microplates in triplicate and the reactions were started by adding 25 μL of 2× reaction buffer containing DTT. The plate was sealed with breathable film and the fluorescence was measured (*t* = 0). End-point mCherry fluorescence was measured after 20 h of incubation at different temperatures with gentle shaking. Normalized fluorescence was calculated by subtracting the values from *t* = 0 and averaging the values from three replicates. Samples exhibiting unexpected fluorescence were collected for Western blot analysis.

### Extraction and analysis of SasG-based proteins

Plasmids encoding the SasG5^3^E^2^-split intein fusion proteins were transformed into *E. coli* BL21. Overnight cultures were diluted 100-fold in 50 mL of LB and incubated at 37 °C (160 rpm) until an OD_600_ of 0.6–0.8. Protein expression was induced with 1 mM IPTG and growth continued for a further 20 h at 20 °C with shaking (160 rpm). Cells were collected by centrifugation at 4696 × *g* for 25 minutes and pellets were stored at −20 °C until needed. Cells were then resuspended in 2 mL of splicing buffer (100 mM Tris-HCl pH 9.0, 100 mM NaCl, and cOmplete Protease Inhibitor Cocktail) and lysed by sonication at maximum output for six cycles of 10 s with a 10 s rest period between each cycle. Clarified cell lysates were prepared by centrifugation at 17,000 × *g* for 20 min at 4 °C, and stored for a maximum of 3 days at 4 °C. Samples from the lysates were separated by SDS-PAGE for analysis and comparative quantification. To confirm the functionality and orthogonality of the SasG5^3^E^2^-split intein fusion proteins, aliquots of the lysates were normalized to the lowest protein concentration and 7 μL of each lysate was mixed together in pairs in PCR tubes. Tris(2-carboxyethyl)phosphine hydrochloride (TCEP; 10252952, Thermo Fisher) was added to a final concentration of 5 mM and the tubes were incubated at room temperature for either 1 h or overnight, after which 5 μL of 4× Laemmli buffer was added to stop the reaction. For the negative controls, 7.5 μL of each corresponding lysate was mixed in a PCR tube already containing 5 μL 4× Laemmli buffer, to prevent the reaction from taking place. In all, 10 μL of each sample was analyzed by SDS-PAGE.

To assess SasG full-length controls expression (SasG5^3^E^2^, SasG5^6^E^4^, SasG5^9^E^6^, and SasG5^12^E^8^), the appropriate plasmids were transformed in *E. coli* BL21 and Origami. Overnight cultures were diluted 100-fold in 5 mL of LB and incubated at 37 °C (160 rpm) until an OD_600_ of 0.6–0.8. Protein expression was induced as above. After the 20 h growth at 20 °C, samples of each culture were collected and clear lysates were prepared as described. Whole cell proteins and clear lysates were analyzed by SDS-PAGE.

### In vitro assembly of elongated SasG proteins

An elongated SasG protein was assembled in “one pot” by mixing all necessary SasG3 assembly units in one reaction. For this purpose, cell lysates at equal concentrations were mixed to reach a final volume of 6 mL and TCEP was added to a final concentration of 5 mM. This mixture was incubated overnight at room temperature with gentle shaking. Subsequently, imidazole (56750, Sigma-Aldrich) was added to a final concentration of 10 mM and the mixture was incubated with 1 mL of equilibrated Ni-NTA resin (Qiagen). The mixture was left overnight at 4 °C and subsequently purified according to the manufacturer’s instructions; elutions were pooled and concentrated by ammonium precipitation. To concentrate the protein sample, ammonium sulfate was added gradually to the protein sample to reach 80% saturation (0.56 g mL^−1^ of protein solution) and after complete solubilization it was incubated at 4 °C overnight. The tube was then centrifuged at 4 °C for 10 min at 17,000 × *g* and the pellet was resuspended in 150 μL of Strep-Tactin buffer W (2-1002-001, IBA GmbH). The protein sample was then incubated with 50 μL Strep-Tactin Sepharose (2-1201-010, IBA GmbH) for 1 hour at 4 °C, in a 1 mL spin column (10351454, Thermo Fisher). Flow through was collected by centrifugation and the column was washed four times with 50 μL of buffer W. The protein was the eluted with six volumes of 12.5 μL of Strep-Tactin buffer E (2-1002-001, IBA GmbH). Samples were collected throughout the process and analyzed by SDS-PAGE.

For the recursive solid-phase assembly, 200 μL of SasG3_8 lysate and 400 μL of SasG3_7 lysate were mixed with 250 μL of equilibrated Ni-NTA resin in a 1 mL spin column and TCEP and imidazole were added to final concentrations of 5 mM and 10 mM, respectively. The column was incubated overnight at room temperature with gentle agitation and the flow through was collected by centrifugation (all centrifuge steps herein were performed at 900 × *g* for 10 s). The resin was then washed with four volumes of 500 μL of wash buffer (50 mM Tris-HCl pH 8.0, 300 mM NaCl and 10 mM imidazole). 400 μL of the next protein lysate was subsequently added to the resin, together with 200 μL of splicing buffer supplemented with TCEP, and the mixture was incubated for 1 h at room temperature with gentle agitation. These cycles of protein lysates addition were repeated every hour, until the desired protein size has been reached. At the penultimate washing step of each cycle, 20 μL of the resin in washing buffer was collected for SDS/PAGE analysis. After the very last washing step, the final protein was eluted with six volumes of 250 μL of elution buffer (50 mM Tris-HCl pH 8.0, 300 mM NaCl, and 200 mM imidazole) and analyzed by SDS-PAGE.

### Protein models

Protein models were built to better illustrate the phenomena depicted in the figures. For this purpose, SWISS-MODEL^58^ was used and the models retrieved were juxtaposed using the Swiss-PdbViewer v4.1 software^59^. Protein structure images were then rendered with the POV-Ray v3.7 software.

### Statistical analysis

The GraphPad Prism v8.1.2 software was used to produce all charts and analyze data. Statistical tests were performed as described in the figure legends and *P* values of ≤0.05 are labeled with a single asterisk (*), in contrast to *P* values of ≤0.01 (**), ≤0.001 (***), or ≤0.0001 (****); non-significant *P* values are labeled as “n.s.”. In all relevant figure panels, values of mean±s.d. are reported, and the exact *n* value is described in each figure legend.

### Reporting summary

Further information on research design is available in the Nature Research Reporting Summary linked to this article.

## Supplementary information

Supplementary Information

Description of Additional Supplementary Files

Supplementary Data 1

Supplementary Data 2

Supplementary Data 3

Supplementary Data 4

Reporting Summary

## Data Availability

All data and plasmids supporting the findings are available from the corresponding author upon reasonable request. Representative plasmids of the 34 selected inteins, as well as the plasmid for the three-input/three-output logic circuit and the corresponding circuit output reporter plasmid are available from Addgene (ID in brackets): pFP.R265 (138167), pFP.R400 (138175), pFP.R267 (138179), pFP.R268 (138180), pFP.R269 (138181), pFP.R270 (138219), pFP.R271 (138220), pFP.R272 (138221), pFP.R401 (138222), pFP.R274 (138223), pFP.R275 (138224), pFP.R355 (138225), pFP.R356 (138226), pFP.R357 (138227), pFP.R358 (138228), pFP.R359 (138229), pFP.R360 (138230), pFP.R361 (138231), pFP.R362 (138232), pFP.R363 (138233), pFP.R364 (138234), pFP.R365 (138235), pFP.R366 (138236), pFP.R367 (138237), pFP.R368 (138238), pFP.R369 (138239), pFP.R370 (138240), pFP.R371 (138241), pFP.R372 (138242), pFP.R373 (138243), pFP.R374 (138244), pFP.R375 (138245), pFP.R376 (138246), pFP.R377 (138247), pFP.E222 (138248), pFP.E227 (138249). The source data and full scan figures for the protein gels and Western blots underlying Figs. 1d–e, 2b, 3a, b, and d, 4b–h and j–l, 5d and 6b, d–e and Supplementary Figs. 2d, 3b, 4a–c, 5a, b, 7, 8a, b, 9a, b, 10a, b, 11a, b, 13a–x, 14a, b, 15a–l, 16a, b, 17a–g, 18a–c, 19c and f, 20b, c, 21d–f and h–j, 22b, c, 23b–c, 24b–c, 25e–g, 26a–d, 27a, b, 28a, b, and 29 are provided as a Source Data file.

## Source data

Source Data
