# Supplementary material for: An expanded library of orthogonal split inteins enables modular multi-peptide assemblies
Source: Nat Commun. 2020 Mar 23;11:1529. doi: 10.1038/s41467-020-15272-2 (PMC7090010; doi:10.1038/s41467-020-15272-2)
Supplement: Supplementary file 1 — Supplementary Information [file 41467_2020_15272_MOESM1_ESM.pdf]

Supplementary Information

An expanded library of orthogonal split inteins enables modular multi-peptide assemblies

Pinto *et al.*

Supplementary Figures

Supplementary Figure 1 | Phylogram of the inteins retrieved from InBase 3

Supplementary Figure 2 | Split mCherry fluorescence assessment........ 4

Supplementary Figure 3 | Effect of the insertion of the junction sequence amino acid residues into mCherry ... 5

Supplementary Figure 4 | *In vitro* analysis of mCherry insertion variants 6

Supplementary Figure 5 | Effect of linker addition in NpuSsp and TerThyX *cis*-splicing activity 7

Supplementary Figure 6 | Structural similarity of the inteins characterized 8

Supplementary Figure 7 | Western blot analysis of *trans*-splicing of inteins split at different sites 9

Supplementary Figure 8 | Effect of linker addition in NpuSsp and TerThyX *trans*-splicing activity........ 10

Supplementary Figure 9 | Highly orthogonal split inteins. 11

Supplementary Figure 10 | Expression and solubility analysis of split mCherry-split intein chimeric proteins 12

Supplementary Figure 11 | Analysis of split mCherry-split intein chimeric proteins extracted under mild denaturing conditions 13

Supplementary Figure 12 | Analysis of *in vitro trans*-splicing conditions 14

Supplementary Figure 13 | Time course analysis of the *in vitro trans*-splicing of 24 split intein pairs 15

Supplementary Figure 14 | End-point analysis of *in vitro trans*-splicing of 24 split intein pairs........ 16

Supplementary Figure 15 | Time course analysis of the *in vitro trans*-splicing of the 12 most active inteins 17

Supplementary Figure 16 | End-point analysis of *in vitro trans*-splicing of the 12 most active inteins........ 18

Supplementary Figure 17 | Split mCherry-split intein-based logic AND gates 19

Supplementary Figure 18 | Split mCherry-split intein-based AND gates exhibiting C-terminal leakiness 20

Supplementary Figure 19 | Characterization of split extracytoplasmic function (ECF) sigma factors........ 21

Supplementary Figure 20 | Effect of linker mutation on ECFs activity 22

Supplementary Figure 21 | Orthogonality analysis of the mutated ECF sigma factors........ 23

Supplementary Figure 22 | ECF16mut x SspGyrB AND gate characterization 24

Supplementary Figure 23 | ECF17mut x NrdJ-1 AND gate characterization 25

Supplementary Figure 24 | ECF20mut x M86 AND gate characterization 26

Supplementary Figure 25 | ECF-based AND gates depend on inteins' splicing activity 27

Supplementary Figure 26 | 3-input/3-output integrated circuit characterization 28

Supplementary Figure 27 | Full-length SasG-based proteins expression analysis........ 29

Supplementary Figure 28 | SasG3 assembly units trans-splicing and cross-reactivity assessment 30

Supplementary Figure 29 | *In vitro* production of up to SasG5^{30}E^{20} by recursive solid-phase assembly 31

Supplementary Tables

Supplementary Table 1 | List of viral and viral-like inteins retrieved from InBase 32

Supplementary Table 2 | Characteristics of the inteins analyzed in this study 36

Supplementary Table 3 | Mini-intein protein sequences and synthesized DNA sequences 38

Supplementary Table 4 | Sequences for the N- and C-termini of the split inteins 42

Supplementary Table 5 | List of primers used in this study 45

Supplementary Table 6 | Regulatory parts and reporters used in this study 50

Supplementary Table 7 | List of the plasmids used in this study 52

Supplementary Table 8 | List and structure of the plasmids constructed in this study 53

Supplementary Table 9 | Theoretical molecular weights of proteins analyzed by SDS-PAGE and Western blots 58

Supplementary Note

Supplementary Note 1 | Methods for constructing the plasmids used in this work 60

Supplementary Data (Provided as individual supplementary files) 63

Supplementary Data 1 | *In vivo* orthogonality data

Supplementary Data 2 | *In vitro* orthogonality data

Supplementary Data 3 | *In vitro* splicing conditions screening data

Supplementary Data 4 | Sequences of the plasmids constructed in this work

Source Data | Source Data file

References 64

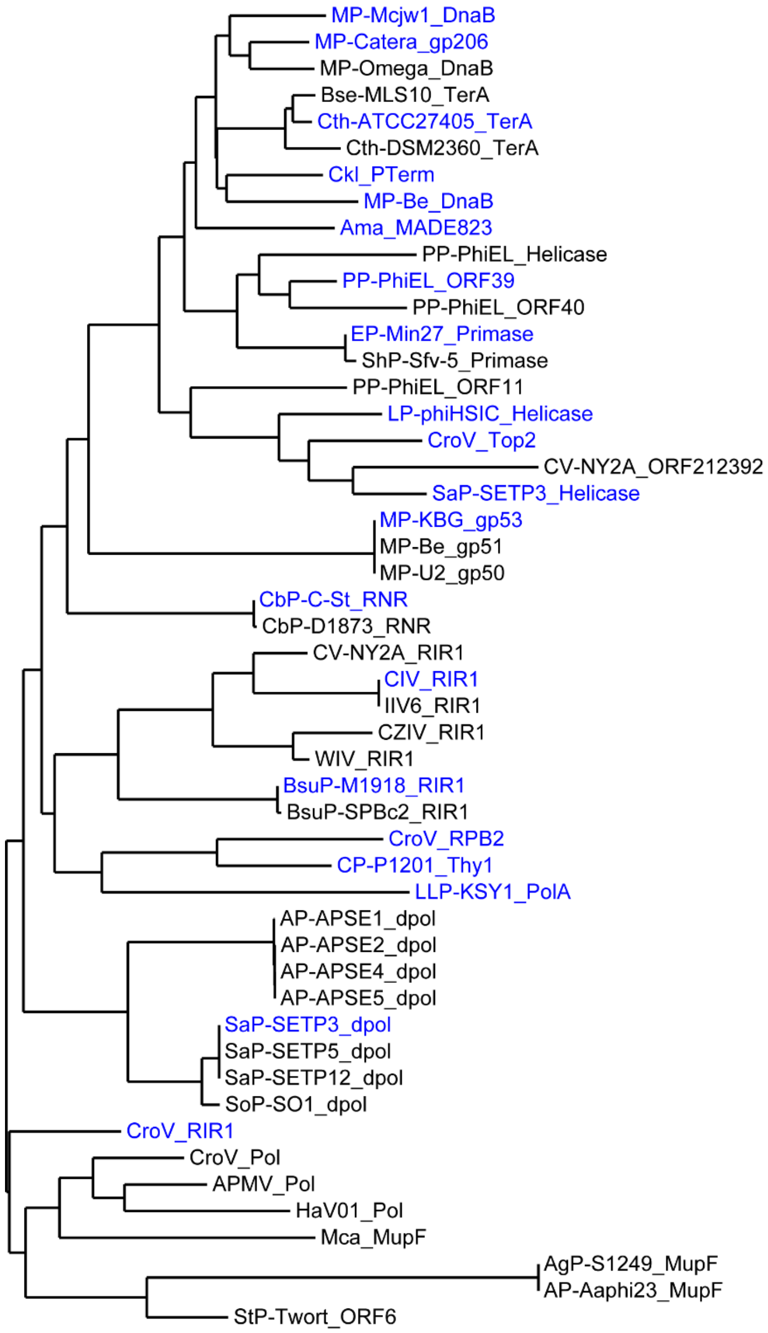

Supplementary Figure 1 | Phylogram of the inteins retrieved from InBase. The phylogenetic tree was constructed based on the structural alignment of the protein sequences of the inteins without endonuclease domains (mini-inteins). The alignment was performed using PROMALS3D, the alignment to tree format conversion was performed using BioNJ and the phylogenetic tree was constructed using TreeDyn 198.3 (both available at the http://www.phylogeny.fr/index.cgi). The inteins selected for further characterization are highlighted in blue.

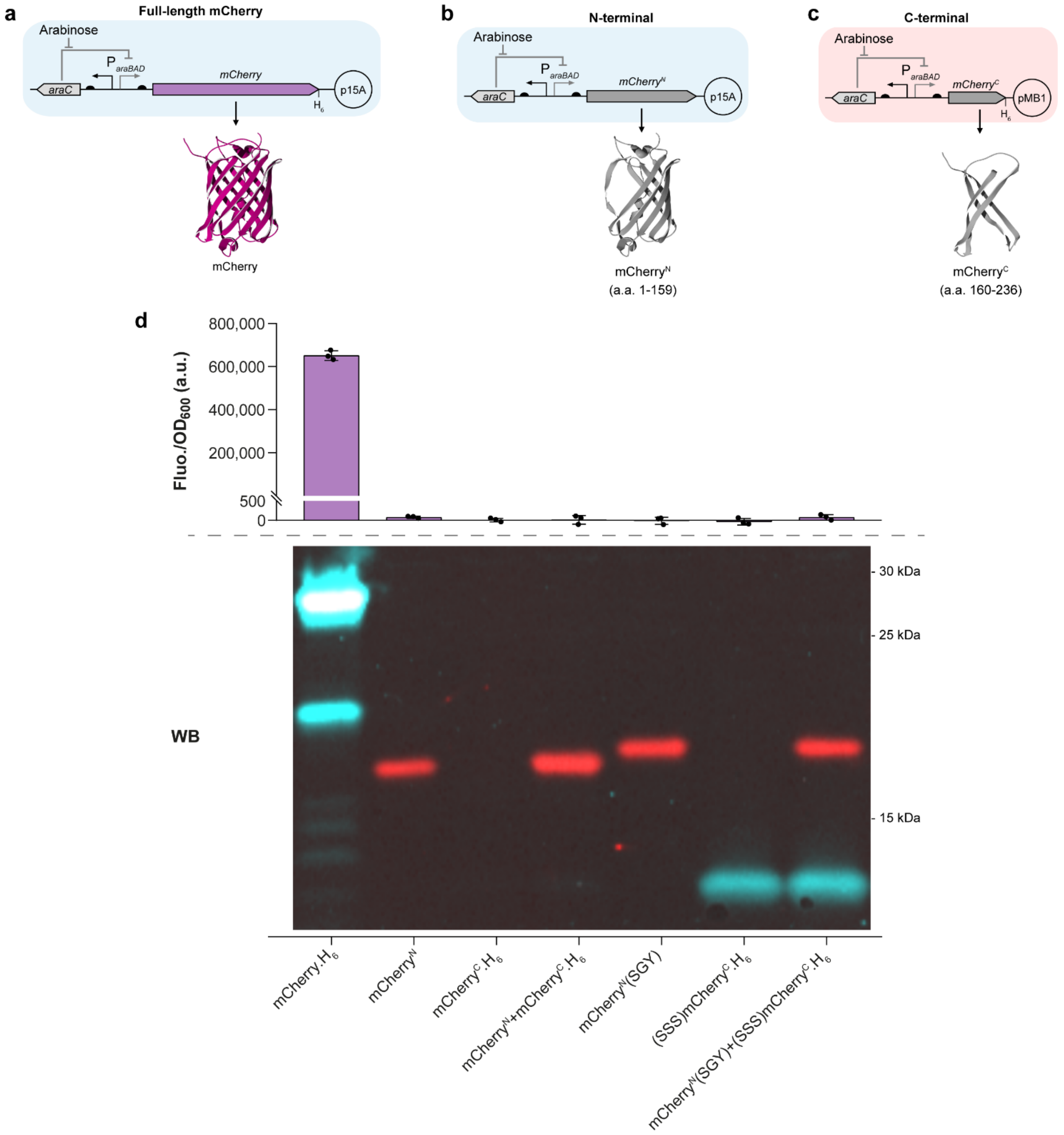

Supplementary Figure 2 | Split mCherry fluorescence assessment. a-c, Genetic circuits for the arabinose-induced expression of full-length mCherry (**a**), mCherry N-terminal (**b**) or mCherry C-terminal (**c**). Split mCherry is expressed from plasmids with different origins of replication. Promoters are represented by straight angle arrows and RBS by black semi-circles. mCherry is shown in purple and split mCherry halves are shown in gray; H_6 - hexahistidine tag. **d**, Background subtracted fluorescence (Fluo., top) and Western blot analysis (bottom) of *E. coli* cells harboring the genetic circuits for the expression of full-length mCherry (mCherry.H_6), mCherry N-terminal (mCherry^N), mCherry C-terminal (mCherry^C.H_6), mCherry N-terminal with extra SGY residues at the C-terminus (mCherry^N(SGY)), mCherry C-terminal with extra SSS residues at the N-terminus ((SSS)mCherry^C.H_6) or combinations of the correlated halves. Fluorescence was measured 6 h after induction and normalized to the cell density (OD_{600}). Bars represent the mean of three biological replicates (n = 3) and error bars correspond to s.d. Samples from the three biological replicates were mixed and used for Western blot analysis. The red signal corresponds to the antibody recognizing the N-terminal of mCherry and the turquoise signal corresponds to the antibody recognizing the hexahistidine-tag (H_6) at the C-terminus. The overlap of both signals (white) corresponds to full-length mCherry. Molecular weights corresponding to the protein standards are shown. Source data are provided as a Source Data file.

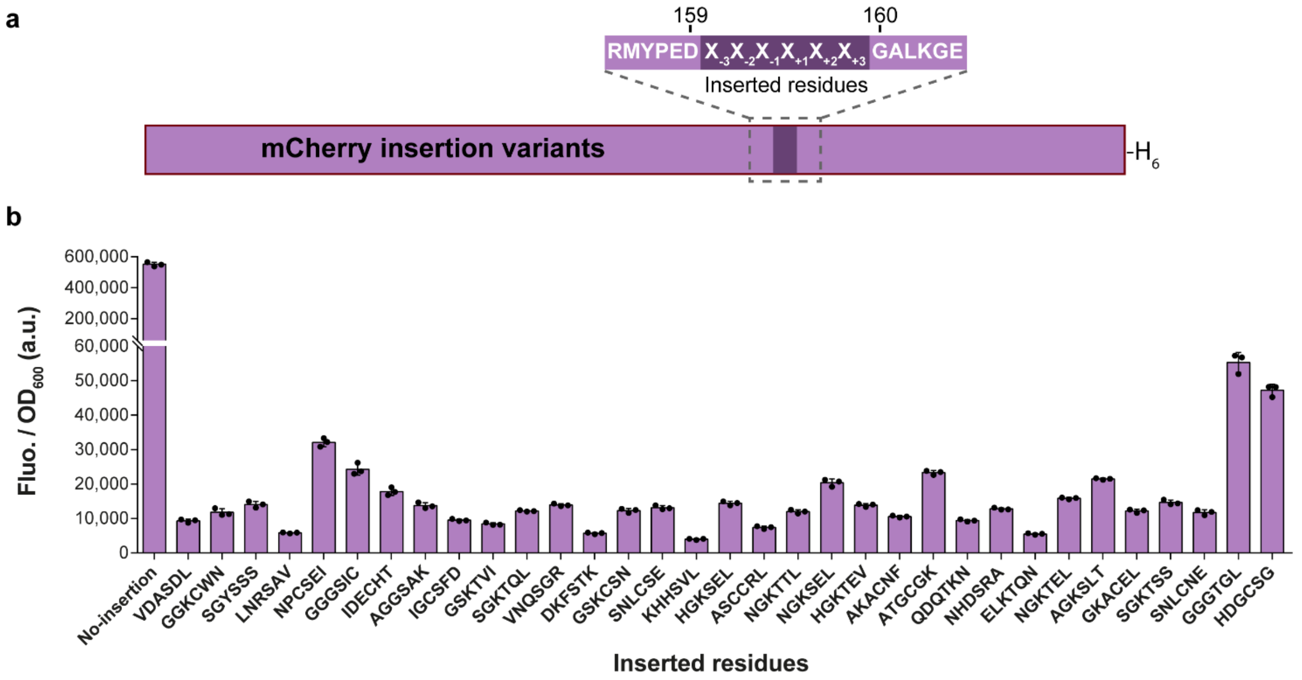

Supplementary Figure 3 | Effect of the insertion of the junction sequence amino acid residues into mCherry. (**a**) Schematics of the mCherry variants produced, showing the insertion of the junction sequence residues (X_{-3}, X_{-2}, X_{-1}, X_{+1}, X_{+2} and X_{+3}) between mCherry residues 159 and 160. (**b**) Background subtracted fluorescence (Fluo.) of *E. coli* cells expressing the native mCherry (no-insertion) or variants with the corresponding inserted amino acid residues. Fluorescence was measured 7 h after induction (5 h of growth followed by 2 h incubation after chloramphenicol addition) and normalized to the cell density (OD_{600}). Bars represent the mean of three biological replicates (*n* = 3) and error bars correspond to s.d. Source data are provided as a Source Data file.

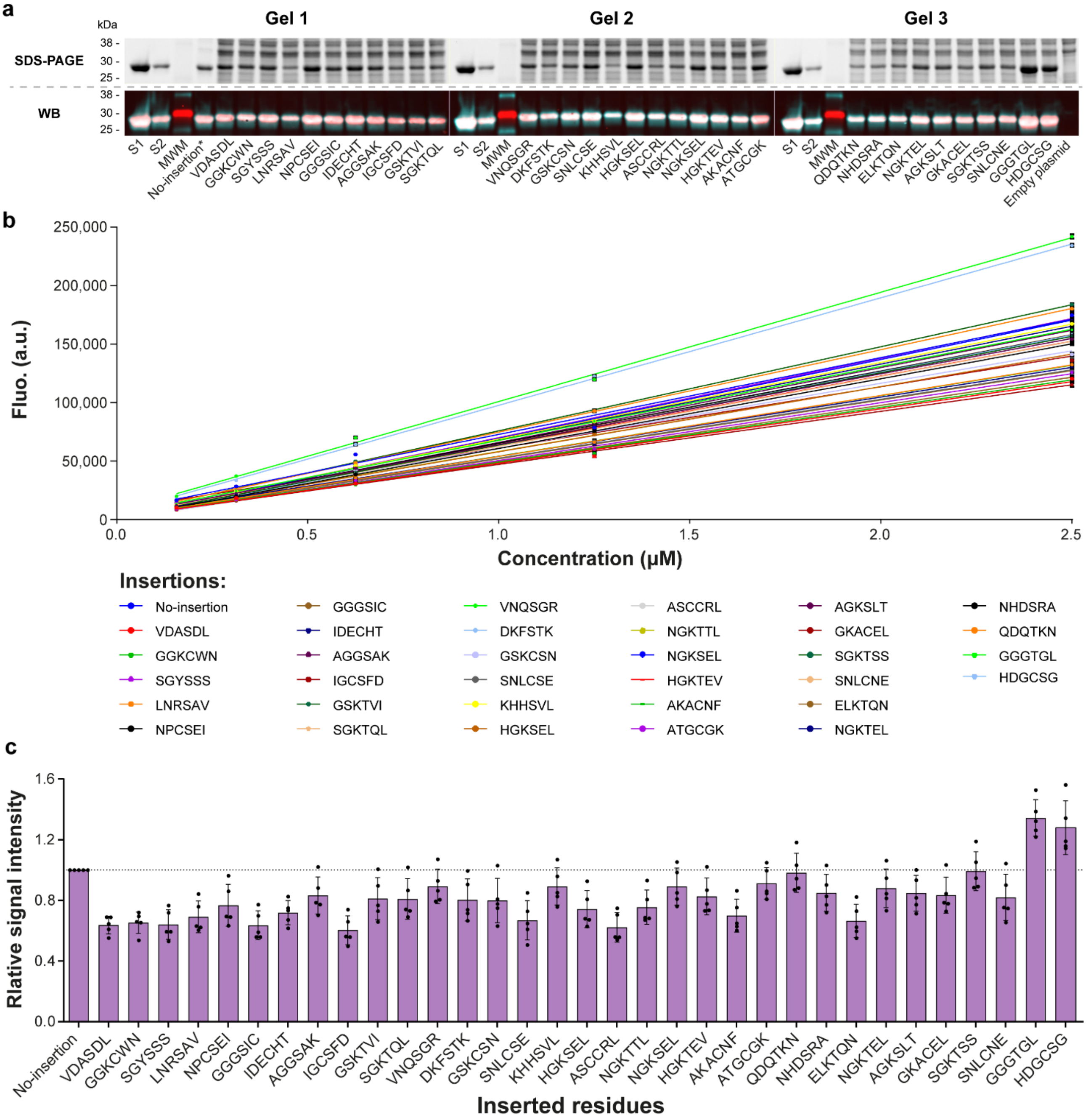

Supplementary Figure 4 | *In vitro* analysis of mCherry insertion variants. a, SDS-PAGE (top) and Western blot (bottom) analyses of lysates of *E. coli* cells expressing the native mCherry (no-insertion) or variants with the corresponding inserted amino acid residues between positions 159 and 160. Cells transformed with the corresponding empty plasmid were included as a control to validate the antibodies. Stain-free SDS-PAGE image was acquired prior to protein transfer. The red signal in the Western blots corresponds to the antibody recognizing the N-terminal of mCherry and the turquoise signal corresponds to the antibody recognizing the hexahistidine-tag (H_6) at the C-terminus. The overlap of both signals (white) corresponds to native mCherry or variants. * - the no-insertion sample was diluted 10-fold before loading the gel; S1 and S2 – standards of 5 pmol and 2.5 pmol of purified mCherry, respectively; MWM – molecular weight marker. **b**, Blank subtracted fluorescence (Fluo.) of 2-fold dilution series of cell lysates normalized the same concentration. Symbols represent the mean of three independent repeats (n = 3), error bars correspond to s.d. and the solid lines represent linear regressions (all R^2 values are above 0.988). **c**, Relative fluorescence intensities of mCherry insertion variants. For each dilution in **b**, Fluo. values were normalized to the no-insertion control and averaged. Bars represent the mean of the calculated relative signal intensities (n = 5) and error bars correspond to s.d. Source data are provided as a Source Data file.

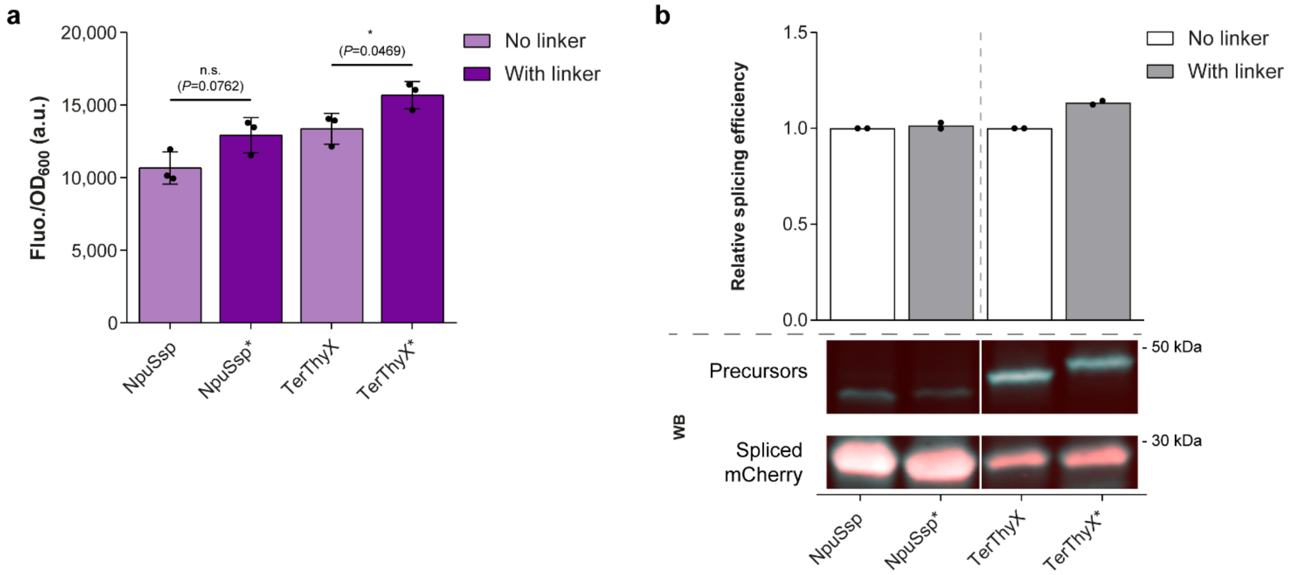

Supplementary Figure 5 | Effect of linker addition in NpuSsp and TerThyX *cis*-splicing activity. a, Background subtracted fluorescence (Fluo.) of *E. coli* cells expressing the mCherry-intein fusion proteins with (*) or without a flexible linker inserted at the canonical endonuclease insertion site. Fluorescence was measured 7 h after induction (5 h of growth followed by 2 h incubation after chloramphenicol addition) and normalized to the cell density (OD_{600}). Bars represent the mean of three biological replicates (*n* = 3) and error bars correspond to s.d. **b**, The relative splicing efficiency (top) as calculated from Western blot analysis (bottom). For each sample in **a**, the three biological replicates were mixed and analyzed by Western blot. The red signal corresponds to the antibody recognizing the N-terminal of mCherry and the turquoise signal corresponds to the antibody recognizing the hexahistidine-tag (H_6) at the C-terminus. The overlap of both signals (white) corresponds to the precursor proteins or to the spliced proteins. The relative splicing efficiency (Eff) was calculated for each antibody signal by dividing the signal intensity (SI) corresponding to the spliced products (Spl) by the sum of the signal from the precursors (Pre) and spliced products ($Eff=SI_{Spl}/(SI_{Spl}+SI_{Pre})$). Bars represent the mean of the calculated relative efficiencies (*n* = 2). Statistical differences in **a**, were determined for each intein, between the two groups (with linker/no linker) by means of two-tailed Student's *t* tests (*P=0.0469; n.s. non-significant, P=0.0762). Source data and detailed statistical analyses are provided as a Source Data file.

a

```
Conservation:      5    5 7                               5                                          5
M86              1 CISGDSLISLA--------------------------STG----KRVPIKDLLGE-----------------------KDFEIWAINEQTMKLESAKVSRVFCTG  52
NpuSsp           1 CLSYETEILTV--------------------------EYG-----SLPIGKIVEK----------------------RIECTVYSVDNNG-NIYTQPVAQWHDRG  51
gp41-1           1 CLDLKTQVQTPQ---------------------------G-----MKEISNIQ-------------------------VGDLVLSNTGY------NEVLNVFPKS  42
gp41-8           1 CLSLDTMVVTNG---------------------------K-----AIEIRDVK-------------------------VGDWLESECG------PVQVTEVLPII  42
NrdJ-1           1 CLVGSSEIITR--------------------------NYG-----KTTIKEVVEIF---------------DNDKN----IQVLAFNTHTDNIEWAPIKAAQLTR  55
IMPDH-1          1 CFVPGTLVNTEN---------------------------G-----LKKIEEIK-------------------------VGDKVFSHTGK-----LQEVVDTLIFD  43
SspDnaX          1 CLTGDSQVLTRN---------------------------G-----LMSIDNPQI------------------------KGREVLSYNETLQQWEYKKVLRWLDRG  49
SspGyrB          1 CFSGDTLVALTD---------------------------G----RSVSFEQLV-------------------EEEKQGKQNFCYTIRHDGSIGVEKIINARKTKT  55
TerThyX          1 CLSGNTKVRFRYSSSSQEAKYYEETIEKLANLWHYGSKNQYTSKDAKCMQENI-------------------------SSRNIFTLDTQTNQIVSSKITNIYING  80
TvoVMA           1 CVSGETPVYLAD---------------------------G----KTIKIKDLYSSERKKEDN--IVEAGSGEEIIHLKDPIQIYSYVDG--TIVRSRSRLLYKGK  70
PhoRadA          1 CFARDTEVYYE--------------------------NDT-----VPHMESIEEMYSKYASM--NGELPFDNGYAVPLDNVFVYTLDIASGEIKKTRASYIYREK  72
Ama-Ter          1 NIAHSTPVLTA--------------------------NRG-----WVTHGDLV-------------------------PGDQVFHPSGK-----PVDVLALSDEA  44
Cth-Ter          1 QLALDTPIPTPD---------------------------G-----WTTMGEIK-------------------------AGDKVIDEKGR-----PCNVVAISEID  43
LLP-Pol          1 CFSGDTEILTPY---------------------------G-----WVKFEDYD-------------------------ESMMCAQYDEVTGKISFTYPNETIHLK  48
LP-Hel           1 CHAYGHDIMMSD---------------------------G----TKKQVQDIA-------------------------VGDKVMGPDGN-----PRKVIRLVKG-  43
MP-B-DnaB        1 PLALNTEVPTPS---------------------------G-----WTTVGDLS-------------------------VGDYVLGSDGQ-----PHRVQRETPVL  43
MP-M-DnaB        1 ALDVETPILTGN---------------------------G-----WKKMGDIQ-------------------------VGDYVHAADGT-----LARVSYVSERH  43
SaP-dpol         1 CLHRHTQVLTDG---------------------------G-----FKDIMAVT-------------------------STDKVWSGEKW------VNTKGAHLMG  42
NrdA-2           1 CLTGDAKIDVLI-----------------------DNIPI----SQISLEEVV-------------------NLFNEGKEIYVLSYNIDTKEVEYKEISDAGLIS  59
Mja-KlbA         1 ALAYDEPIYLS--------------------------DGN-----IINIGEFVDKFFKKYKNSIKKEDNGFGWIDIGNENIYIKSFNKLSLIIEDKRILRVWRKK  74
Consensus aa:      ths.po.l.h.............................s.......ph.ph...........................s.blhs.s..........h..h....
Consensus ss:            eee                                    eeehhh                              eeeee      eeeeeeeeeee

Conservation:             5     6     5  5  9
M86             53 -KKLVYTLK-TRLG---RTIKATANHRFLTIDG----------------WKRLDE-------LSL------------KEHIA-----------------------  94
NpuSsp          52 -EQEVFEYC-LEDG---SLIRATKDHKFMTVDGQ---------------MLPIDE-------IFE------------RELDL-----------------------  94
gp41-1          43 -KKKSYKIT-LEDG---KEIICSEEHLFPTQTGE---------------MNISGG-------LKE------------GMCLY-----------------------  85
gp41-8          43 -KQPVFEIV-LKSG---KKIRVSANHKFPTKDGL---------------KTINSG-------LKV------------GDFLR-----------------------  85
NrdJ-1          56 PNAELVELE-IDTLHGVKTIRCTPDHPVYTKNRG---------------YVRADE-------LTD------------DDELV----------------------- 102
IMPDH-1         44 RDEEIISI---------NGIDCTKNHEFYVIDKENANRVNEDNIHLFARWVHAEE-------LDM------------KKHLL-----------------------  97
SspDnaX         50 -EKQTLSIK--TKN---STVRCTANHLIRTEQG----------------WTRAEN-------ITP------------GMKIL-----------------------  90
SspGyrB         56 -NAKVIKVT-LDNG---ESIICTPDHKFMLRDGS---------------YKCAMD-------LTL------------DDSLM-----------------------  98
TerThyX         81 -EKETYTIK-TVSG---KEIRATLEHQFWTNQG----------------WKRLKD-------FNN------------STQLC----------------------- 122
TvoVMA          71 -SSYLVRIE-TIGG---RSVSVTPVHKLFVLTEKGI------------EEVMASN-------LKV------------GDMIA----------------------- 116
PhoRadA         73 -VEKLIEIK-LSSG---YSLKVTPSHPVLLFRDGL-------------QWVPAAE-------VKP------------GDVVV----------------------- 117
Ama-Ter         45 --VDDYVVT-FTNG---EKIRCHANHEWTVYSRADKQ----------EKTVETKW-------FLENTNRGTPRSLTAGNRFQ----------------------- 103
Cth-Ter         44 DTEQAYKIN-FRDG---TSIVAGERHLWKVQVTNNGR---------REKLLTTGE-------MYQKQFK----TKSKENRAL----------------------- 101
LLP-Pol         49 --DQDIWVY-EDTN---TSIHATGNHDILIQKPN---------------GDIAKEKFSNLQLLQKGD----------KHRFI-----------------------  99
LP-Hel          44 -QDEMFRVT-PTKG---ESFVVNGGHILSLYQTPRRAGQ-----TPGYTEISVNE-------YIR------------SSSTF-----------------------  96
MP-B-DnaB       44 EGLATYVVR-FDDG---TEITASASHGWTTQRLTGHGD------SYETVTVTTEE-------LAQTVTN-----SKGRKRHR----------------------- 103
MP-M-DnaB       44 -WRDCFSVQ-FADG---AELVASDHHLWAVNDRLKG-----------ERVIDTAE-------LYRTQTY----GARGDRRYT-----------------------  98
SaP-dpol        43 -WKPVINVD---------GVLMTEDHKILTHS-----------------WKQAKQ-------LVS------------NKYMMDRALEIGMDAWLSCASYQNDKAK 101
NrdA-2          60 ESAEVLEIIDEETG---QKIVCTPDHKVYTLNRG---------------YVSAKD-------LKE------------DDELV----------------------- 104
Mja-KlbA        75 YSGKLIKIT-TKNR---REITLTHDHPVYISKTGEV------------LEINAEM-------VKV------------GDYIY----------------------- 121
Consensus aa:      ....hhpl..hpss...ppl.ho.sH.h.h.p...................h.p.......h...............p.hh.......................
Consensus ss:         eeeeee e       eeee     eeee                  eeehhh                        eee

Conservation:                                                                        65  5    5 5            69
M86             95 --------LPRK---LE-------------SSSLQLAPEIEKLPQSDIYWDPIVSITETG-VEEVFDL-TVPG-LRNF-VAN--D--IIVHN 154
NpuSsp          95 --------MRVD---NLPN------------------------------MVKVIGRRSLG-VQRIFDI-GLRQ-DHNF-LLA---NGAIAAN 147
gp41-1          86 --------VKE--------------------------------------LKKILKIEELD-ERELIDI-EVSG-NHLF-YAN--D--ILTHN 125
gp41-8          86 --------SRAK---------------------------MCEIFENEIDWDEIASIEYVG-VEETIDI-NVTN-DRLF-FAN--G--ILTHN 134
NrdJ-1         103 --------VAI--------------------------------MEAKTYIGKLKSRKIVS-NEDTYDI-QTS--THNF-FAN--D--ILVHN 145
IMPDH-1         98 --------IELE---------------------------------MKFKLKEITSIETKHYKGKVHDL-TVNQ-DHSY-NVR--G--TVVHN 141
SspDnaX         91 --------SPA--------------------------------PQWHTNFEEVESVTKGQ-VEKVYDL-EVED-NHNF-VAN--G--LLVHN 134
SspGyrB         99 --------PLHR---KISTTEDSGHA-------------MEAVL---NYNHRIVNIEAVSETIDVYDI-EVPH-THNFALAS--G--VFVHN 157
TerThyX        123 --------EVQL---A-------------------------------GVFVEIESIEKFG-KEITYDL-EVEHPEHNF-IAN--G--LVVHN 175
TvoVMA         117 --------AVAE---SESEARDCGMSEECV-------MEAEVYTSLEATFDRVKSIAYEKGDFDVYDL-SVPEYGRNF-IGGEGL--LVLHN 186
PhoRadA        118 --------GVRE---EVLRR--------------------RIISKGELEFHEVSSVRIIDYNNWVYDL-VIPE-THNF-IAPN-G--LVLHN 172
Ama-Ter        104 --------FQVP---KTNALE--------------------------ERRVSIEKVEYLPNGEKGHCI-QVDSPDGLY-LVG--KKLVATHN 164
Cth-Ter        102 --------FRIP---IADAFI--------------------------SHFHYIKSIEKTG-KTKMRCI-QVDSPSRLY-LAG--KSMIPTHN 161
LLP-Pol        100 --------NAGY---VDSAEEVDTLMQRLV-----------------SFPGKAFPTGPSY-KGDVYCV-NVPT--HNIVIRHN-DKVSIQGN 168
LP-Hel          97 --------KHRSKLQRFGFD---------------------------RVDVTGFKVEPIG-VGDYYGF-TVDG-DHLY-LDG--D-FVRHHN 156
MP-B-DnaB      104 --------IPVV---GME-----------------------------SQHRWVESVTPVE-SVPVKCI-GIDTEDHLF-QVSRSR--ILTHN 160
MP-M-DnaB       99 --------VTVP---EALD----------------------------ARTNTITSVTPVP-TVETVCI-QIDHPSHVF-LAG--KSLTPTHN 156
SaP-dpol       102 DNYSSNVIVERC---LG------------------------------NLNGKLTYCE------PVYDLIDVEDGNRFL-IAS-DSGFLVAHN 162
NrdA-2         105 --------FS-----------------------------------------GLKIIKRES-KEPVFDI-TVKD-NSNF-FAN--N--ILVHN 149
Mja-KlbA       122 --------IPKN---NT------------------------------INLDEVIKVETVDYNGHIYDL-TVED-NHTY-IAGKNEG-FAVSN 178
Consensus aa:      ...................................................pl..hp.......hhsl.pl.p.s+.@.hhs.....hhhHN
Consensus ss:              ee                                        eeeeeeee     eeeee ee      e ee      eee
```

b

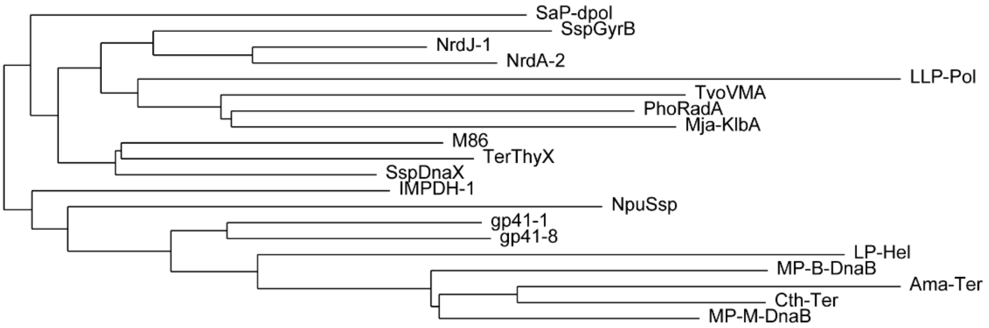

Supplementary Figure 6 | Structural similarity of the inteins characterized. a, Structural alignment of the multiple sequences of inteins without the endonuclease domains (mini-inteins), performed using PROMALS3D (see http://prodata.swmed.edu/promals3d/promals3d.php for output style). **b**, Phylogram of the inteins based on the alignment shown in **a**. The alignment to tree format conversion was performed using BioNJ and the phylogenetic tree was constructed using TreeDyn 198.3 (both available at the http://www.phylogeny.fr/index.cgi).

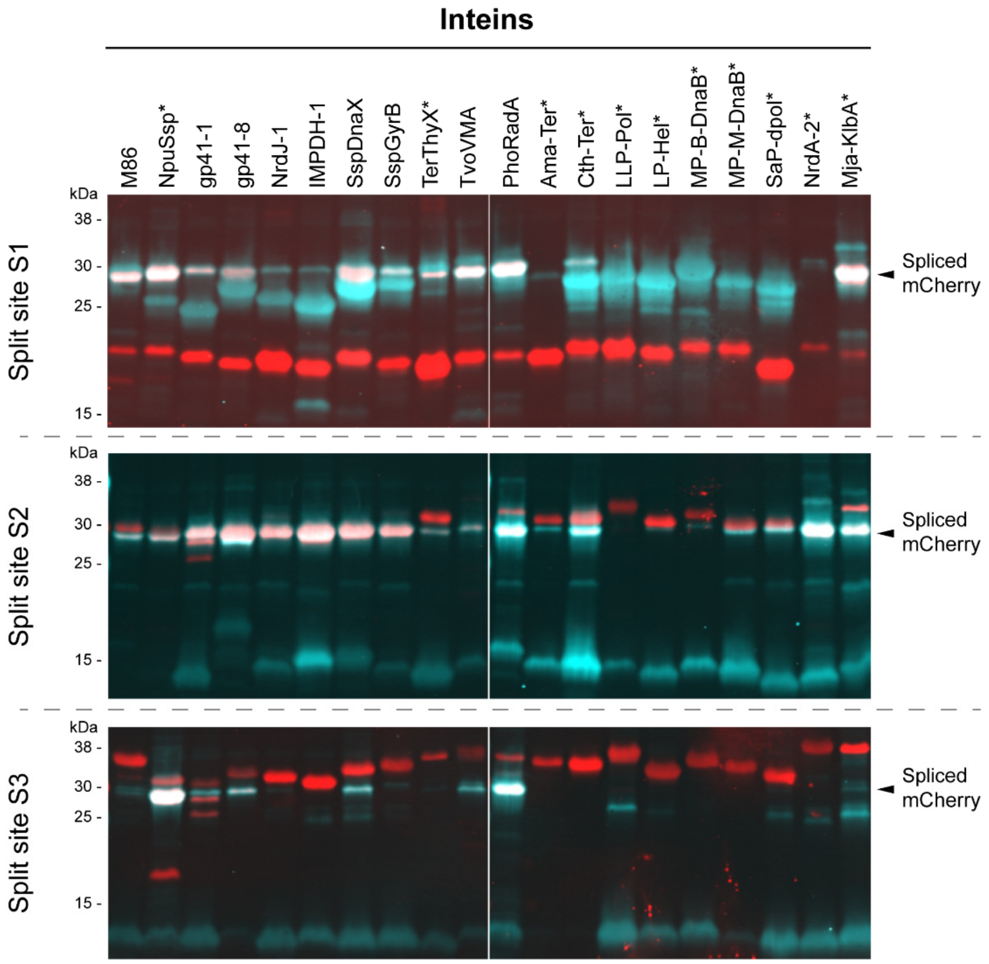

Supplementary Figure 7 | Western blot analysis of *trans*-splicing of inteins split at different sites. Samples from the three biological replicates of *E. coli* cells expressing chimeric mCherry halves fused to counterpart inteins split at the three different sites (S1, S2 and S3, shown in **Fig. 2b**) were mixed together and analyzed. The red signal corresponds to the antibody recognizing the N-terminal of mCherry and the turquoise signal corresponds to the antibody recognizing the hexahistidine-tag (H_6) at the C-terminus. The overlap of both signals (white) corresponds to the spliced mCherry. Source data are provided as a Source Data file.

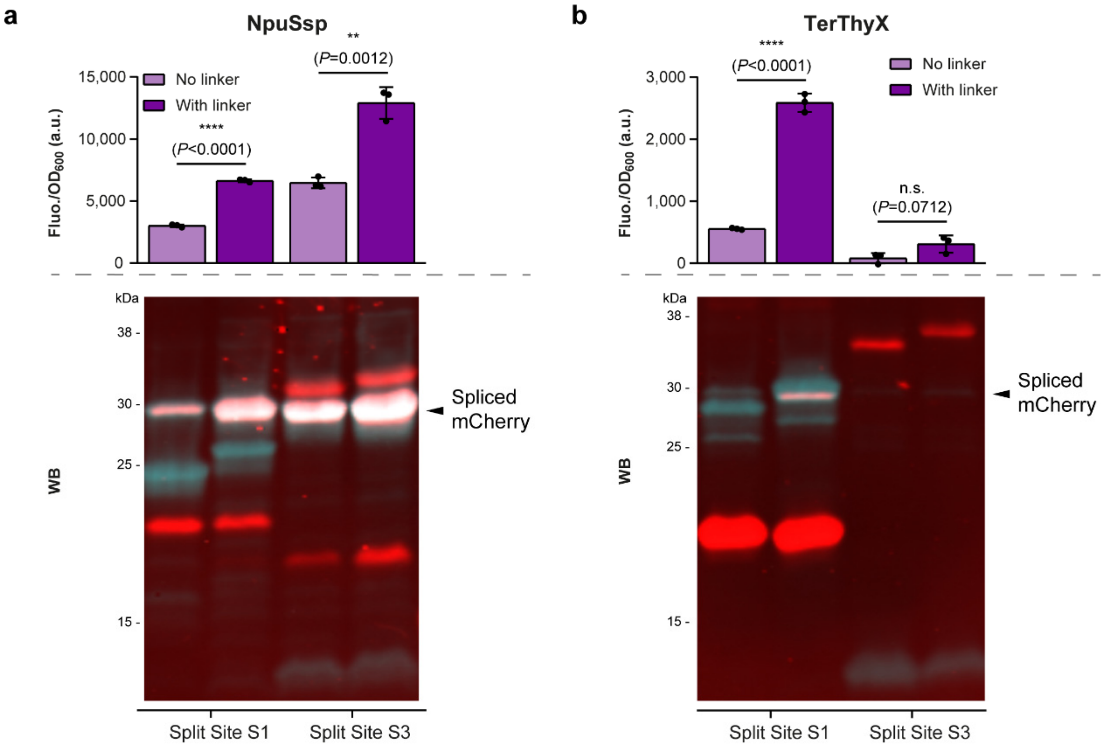

Supplementary Figure 8 | Effect of linker addition in NpuSsp and TerThyX *trans*-splicing activity. a-b, Background subtracted fluorescence (Fluo. top) and Western blot analysis (bottom) of *E. coli* cells expressing split mCherry proteins fused to NpuSsp (**a**) or TerThyX (**b**) split at the atypical sites S1 and S3, with or without a flexible linker inserted at the canonical endonuclease insertion site of the inteins. Fluorescence was measured 7 h after induction (5 h of growth followed by 2 h incubation after chloramphenicol addition) and normalized to the cell density (OD_{600}). Bars in **a** and **b** represent the mean of three biological replicates (*n* = 3) and error bars correspond to s.d. For each sample, the three biological replicates were mixed and analyzed by Western blot. The red signal in Western blots corresponds to the antibody recognizing the N-terminal of mCherry and the turquoise signal corresponds to the antibody recognizing the hexahistidine-tag (H_6) at the C-terminus. The overlap of both signals (white) corresponds to the spliced mCherry. Statistical differences were determined for each intein, between the two groups (with linker/no linker) by means of two-tailed Student's *t* tests (**P=0.0012; ****$P \leq 0.0001$; n.s. non-significant, P=0.0712). Source data and detailed statistical analyses are provided as a Source Data file.

a

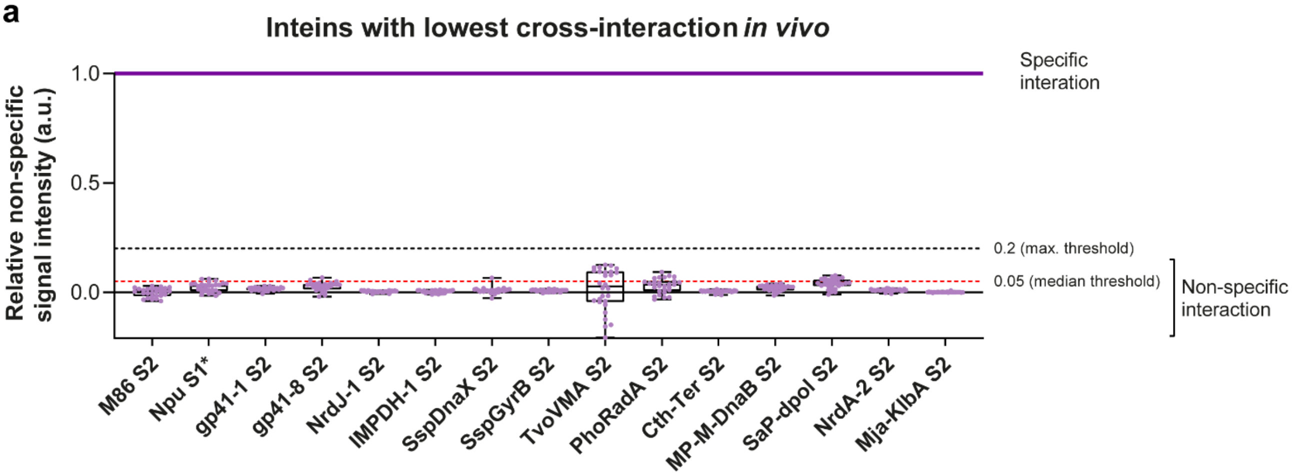

b

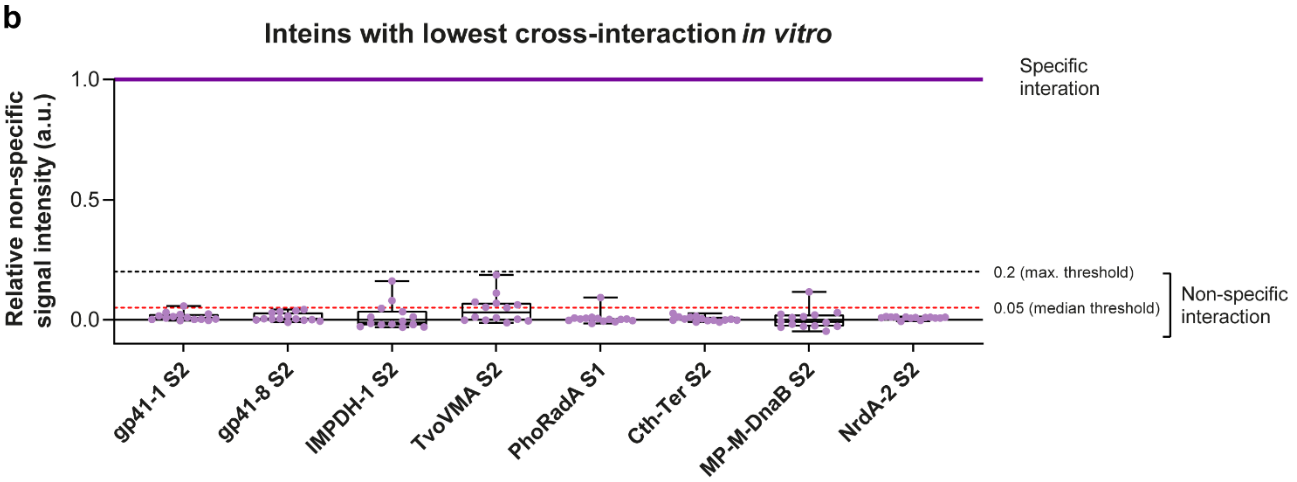

Supplementary Figure 9 | Highly orthogonal split inteins. Relative non-specific signal intensities for the split inteins exhibiting the lowest mutual cross-interaction *in vivo* (**a**) and *in vitro* (**b**). The relative fluorescence values of non-specific intein halves combinations were calculated for each intein using the specific fluorescence as reference. The average Fluo./OD_{600} (**a**) or Fluo. (**b**) values from Supplementary Data 1 and 2 were respectively used for calculations. For *in vivo* conditions n = 28 non-specific interactions and for *in vitro* conditions n = 14 non-specific interactions. Two thresholds were set to define the low degree of mutual cross-interaction: 0.05 (dashed red line) as the maximum for the median value and 0.2 (dashed black line) as the maximum for any individual value. The purple line corresponds to the specific interaction value of 1.0. For box-and-whisker plots, the center, boxes and whiskers represent the median, interquartile range, and the maximum to minimum range, respectively. Source data are provided as a Source Data file.

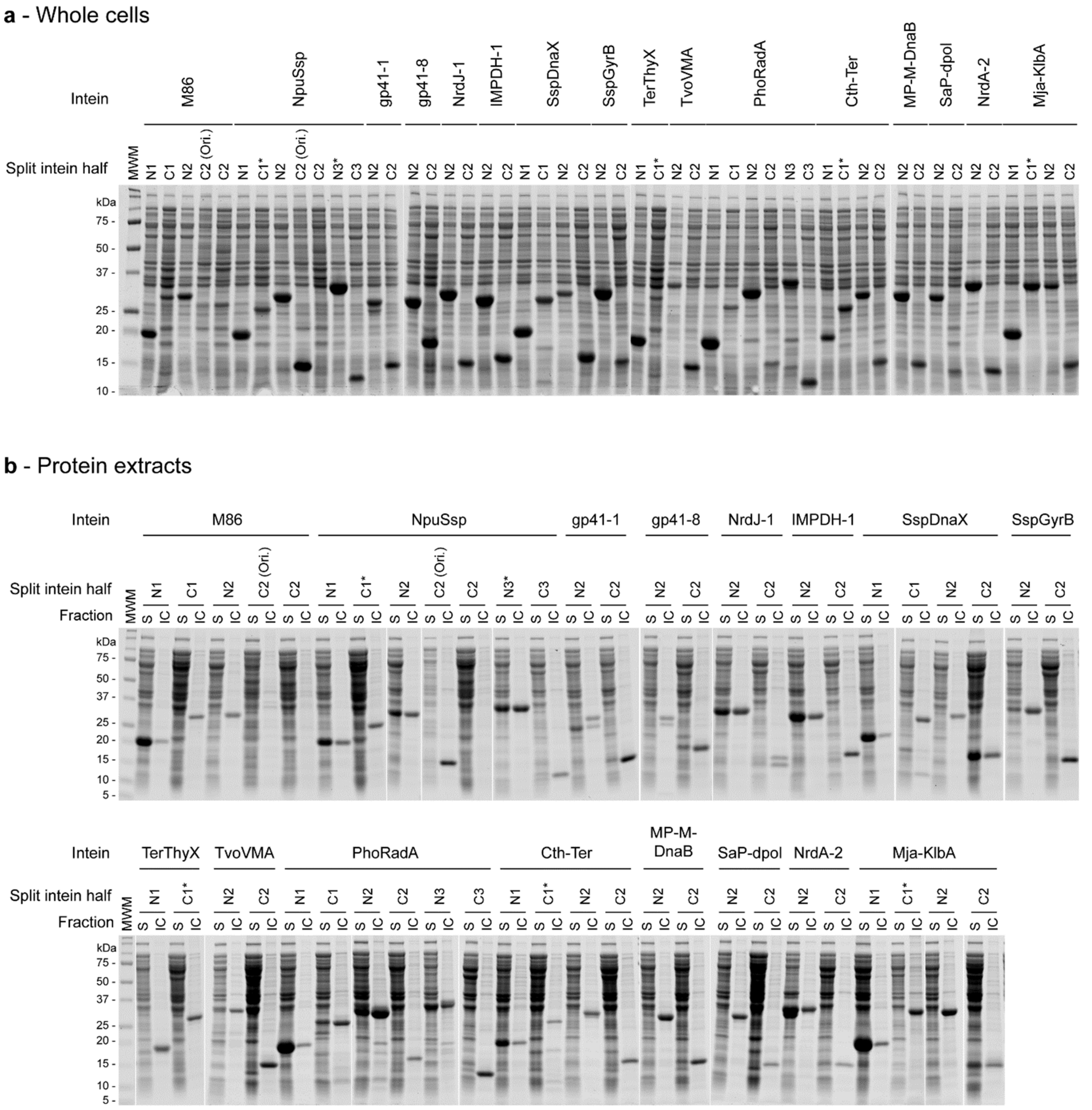

Supplementary Figure 10 | Expression and solubility analysis of split mCherry-split intein chimeric proteins. a, SDS-PAGE analysis of whole *E. coli* TOP10 or Origami cells expressing N- or C-terminal mCherry chimeric proteins fused to inteins split at sites S1 (N1 and C1), S2 (N2 and C2) or S3 (N3 and C3). **b**, SDS-PAGE analysis of soluble fractions (S) or solubilized inclusion bodies (IC) prepared from *E. coli* cultures as described in **a**. MWM - Molecular weight marker. Ori. - *E. coli* Origami; * - Inteins with a flexible linker at the canonical split site. The proteins' theoretical molecular weights can be found in **Supplementary Table 9**. Protein gel images are representative of at least two independent experiments with similar results. Source data are provided as a Source Data file.

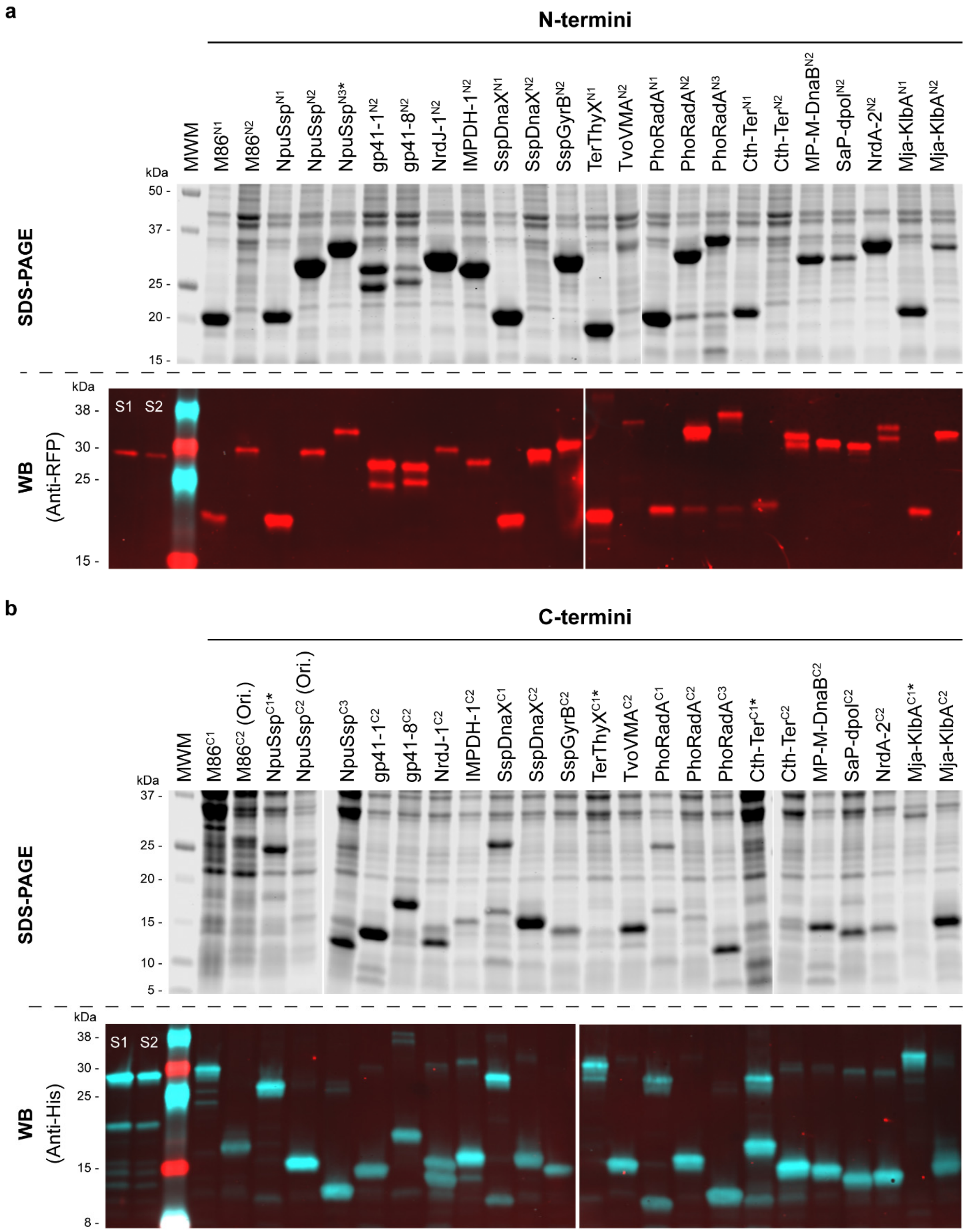

Supplementary Figure 11 | Analysis of split mCherry-split intein chimeric proteins extracted under mild denaturing conditions. a-b, SDS-PAGE (top) and Western blot (bottom) analyses of cell lysates of *E. coli* TOP10 or Origami cells expressing N-terminal (**a**) or C-terminal (**b**) mCherry chimeric proteins fused to inteins split at sites S1 (N1 and C1), S2 (N2 and C2) and S3 (N3 and C3), prepared under mild denaturing conditions. In the Western blots, the red signal corresponds to the antibody recognizing the N-terminal of mCherry (**a**) and the turquoise signal corresponds to the antibody recognizing the hexahistidine-tag (H_6) at the C-terminus (**b**). S1 and S2 – standards of 2.5 pmol and 1.25 pmol of purified mCherry, respectively; MWM - molecular weight marker. Ori. - *E. coli* Origami; * - Inteins with a flexible linker at the canonical split site. The proteins' theoretical molecular weights can be found in **Supplementary Table 9**. Protein gel images and blots are representative of at least two independent experiments with similar results. Source data are provided as a Source Data file.

M86 S1

pH	8			9		
NaCl (mM)	100	300	500	100	300	500
4 °C	5	5	9	2	0	-2
21 °C (RT)	70	90	79	72	100	88
30 °C	31	55	52	27	54	48
37 °C	5	26	32	4	21	22
42 °C	1	2	4	-2	-1	4

M86 S2

pH	8			9		
NaCl (mM)	100	300	500	100	300	500
4 °C	17	26	0	15	-16	-3
21 °C (RT)	26	26	31	25	49	50
30 °C	61	74	84	48	96	100
37 °C	-18	-18	-17	-7	16	11
42 °C	-25	-17	-22	-24	-22	-32

NpuSsp S1*

pH	8			9		
NaCl (mM)	100	300	500	100	300	500
4 °C	1	1	0	1	-1	-1
21 °C (RT)	77	56	42	70	30	21
30 °C	100	73	59	78	35	23
37 °C	100	86	78	56	34	25
42 °C	16	23	14	10	2	7

NpuSsp S2

pH	8			9		
NaCl (mM)	100	300	500	100	300	500
4 °C	8	12	6	8	3	-1
21 °C (RT)	71	82	74	74	75	64
30 °C	91	85	84	75	64	61
37 °C	80	82	100	73	62	50
42 °C	27	24	20	29	17	8

NpuSsp S3*

pH	8			9		
NaCl (mM)	100	300	500	100	300	500
4 °C	3	-1	6	-2	-7	-10
21 °C (RT)	61	36	35	100	36	33
30 °C	37	20	16	62	22	14
37 °C	9	2	-3	5	-3	3
42 °C	-6	-1	-8	-10	-5	-13

gp41-1 S2

pH	8			9		
NaCl (mM)	100	300	500	100	300	500
4 °C	7	5	6	11	7	5
21 °C (RT)	83	79	75	100	83	74
30 °C	91	80	75	100	86	77
37 °C	85	79	72	91	81	65
42 °C	26	31	22	44	24	25

gp41-8 S2

pH	8			9		
NaCl (mM)	100	300	500	100	300	500
4 °C	9	7	4	10	4	1
21 °C (RT)	78	70	60	87	74	60
30 °C	87	80	71	100	85	68
37 °C	82	72	60	95	74	56
42 °C	29	26	18	41	23	18

NrdJ-1 S2

pH	8			9		
NaCl (mM)	100	300	500	100	300	500
4 °C	14	12	16	13	6	6
21 °C (RT)	100	82	84	90	64	67
30 °C	67	59	59	70	58	54
37 °C	52	46	42	55	51	46
42 °C	12	23	15	30	18	16

IMPDH-1 S2

pH	8			9		
NaCl (mM)	100	300	500	100	300	500
4 °C	10	10	11	14	11	8
21 °C (RT)	71	75	75	100	96	94
30 °C	75	71	64	93	86	79
37 °C	70	66	61	80	77	64
42 °C	17	27	17	37	18	25

SspDnaX S1

pH	8			9		
NaCl (mM)	100	300	500	100	300	500
4 °C	1	2	3	2	-1	-1
21 °C (RT)	75	27	21	100	24	19
30 °C	50	24	15	64	21	14
37 °C	25	13	9	22	7	7
42 °C	2	2	1	6	-3	0

SspDnaX S2

pH						
NaCl (mM)	100	300	500	100	300	500
44C	7	10	9	13	-10	-6
21 °C (RT)	89	95	93	98	100	94
30 °C	87	90	85	87	90	87
37 °C	75	82	79	75	74	66
42 °C	13	17	13	33	9	9

SspGyrB S2

pH						
NaCl (mM)	100	300	500	100	300	500
4 °C	8	5	5	10	6	4
21 °C (RT)	72	70	66	84	77	73
30 °C	90	81	72	92	87	76
37 °C	92	86	77	100	97	74
42 °C	34	35	25	58	31	29

TerThyX S1*

pH	8			9		
NaCl (mM)	100	300	500	100	300	500
4 °C	7	-1	-25	-6	-5	-25
21 °C (RT)	94	65	68	100	86	74
30 °C	45	37	36	63	41	37
37 °C	16	17	4	33	25	14
42 °C	5	1	0	-2	3	-6

TvoVMA S2

pH	8			9		
NaCl (mM)	100	300	500	100	300	500
4 °C	-1	3	-25	0	-5	-10
21 °C (RT)	61	61	49	69	56	58
30 °C	93	75	72	96	77	64
37 °C	83	76	66	100	71	65
42 °C	13	17	9	26	16	16

PhoRadA S1

pH	8			9		
NaCl (mM)	100	300	500	100	300	500
4 °C	2	0	-4	0	-1	-1
21 °C (RT)	50	27	19	34	15	9
30 °C	86	30	26	47	18	10
37 °C	100	51	27	52	18	12
42 °C	27	9	8	16	3	1

PhoRadA S2

pH	8			9		
NaCl (mM)	100	300	500	100	300	500
4 °C	-1	1	0	2	0	0
21 °C (RT)	67	64	51	80	69	58
30 °C	98	78	69	100	88	70
37 °C	83	82	71	98	85	70
42 °C	30	23	20	40	22	19

PhoRadA S3

pH	8			9		
NaCl (mM)	100	300	500	100	300	500
4 °C	0	1	0	2	1	1
21 °C (RT)	84	47	43	65	26	21
30 °C	100	51	52	72	27	21
37 °C	68	35	27	47	14	11
42 °C	15	6	5	9	4	2

Cth-Ter S1*

pH	8			9		
NaCl (mM)	100	300	500	100	300	500
4 °C	7	2	-6	-5	5	-9
21 °C (RT)	100	76	75	94	41	39
30 °C	30	21	21	29	6	4
37 °C	-10	-12	-23	-6	-21	-3
42 °C	-2	1	-18	-8	-7	3

Cth-Ter S2

pH	8			9		
NaCl (mM)	100	300	500	100	300	500
4 °C	3	2	2	5	2	0
21 °C (RT)	85	85	67	100	94	64
30 °C	41	82	70	54	86	69
37 °C	5	22	29	6	26	29
42 °C	0	0	0	1	0	1

MP-M-DnaB S2

pH	8			9		
NaCl (mM)	100	300	500	100	300	500
4 °C	3	2	2	4	0	-1
21 °C (RT)	73	54	46	100	65	50
30 °C	82	48	43	96	49	34
37 °C	9	2	1	10	-2	-1
42 °C	0	-1	-2	1	2	1

SaP-dpol S2

pH	8			9		
NaCl (mM)	100	300	500	100	300	500
4 °C	4	4	3	3	0	-1
21 °C (RT)	84	100	97	72	73	80
30 °C	43	42	70	19	19	23
37 °C	2	6	10	1	3	1
42 °C	-1	-1	-1	1	1	0

NrdA-2 S2

pH	8			9		
NaCl (mM)	100	300	500	100	300	500
4 °C	9	6	5	9	7	4
21 °C (RT)	75	74	69	100	90	86
30 °C	76	58	61	87	70	61
37 °C	57	46	37	58	32	26
42 °C	14	9	8	11	4	4

Mja-KlbA S1*

pH	8			9		
NaCl (mM)	100	300	500	100	300	500
4 °C	3	9	10	7	3	-1
21 °C (RT)	51	86	94	46	85	92
30 °C	63	99	100	40	98	94
37 °C	36	86	84	16	61	68
42 °C	9	23	30	7	13	19

Mja-KlbA S2

pH	8			9		
NaCl (mM)	100	300	500	100	300	500
4 °C	-12	-18	-22	-10	-14	-29
21 °C (RT)	87	67	70	100	54	60
30 °C	70	56	42	57	31	37
37 °C	63	81	67	73	57	49
42 °C	32	40	48	49	40	39

Supplementary Figure 12 | Analysis of *in vitro trans*-splicing conditions. Cell extracts of *E. coli* cells expressing matching N- or C-terminal mCherry chimeric proteins fused to inteins split at different sites (S1, S2 and S3) were mixed together and the effect of pH (8 or 9) and salt (100, 300 or 500 mM NaCl) on splicing was evaluated after 13 h incubation at 4 °C, room temperature (RT, 21 °C), 30 °C, 37 °C or 42 °C. Color gradient represents relative fluorescence intensities normalized to the maximum signal within each split intein matrix and is represented as percentage. Data represents mean values of two independent replicates (*n* = 2) and the normalized fluorescence values can be found in **Supplementary Data 3**. * - Inteins with a flexible linker at the canonical endonuclease insertion site.

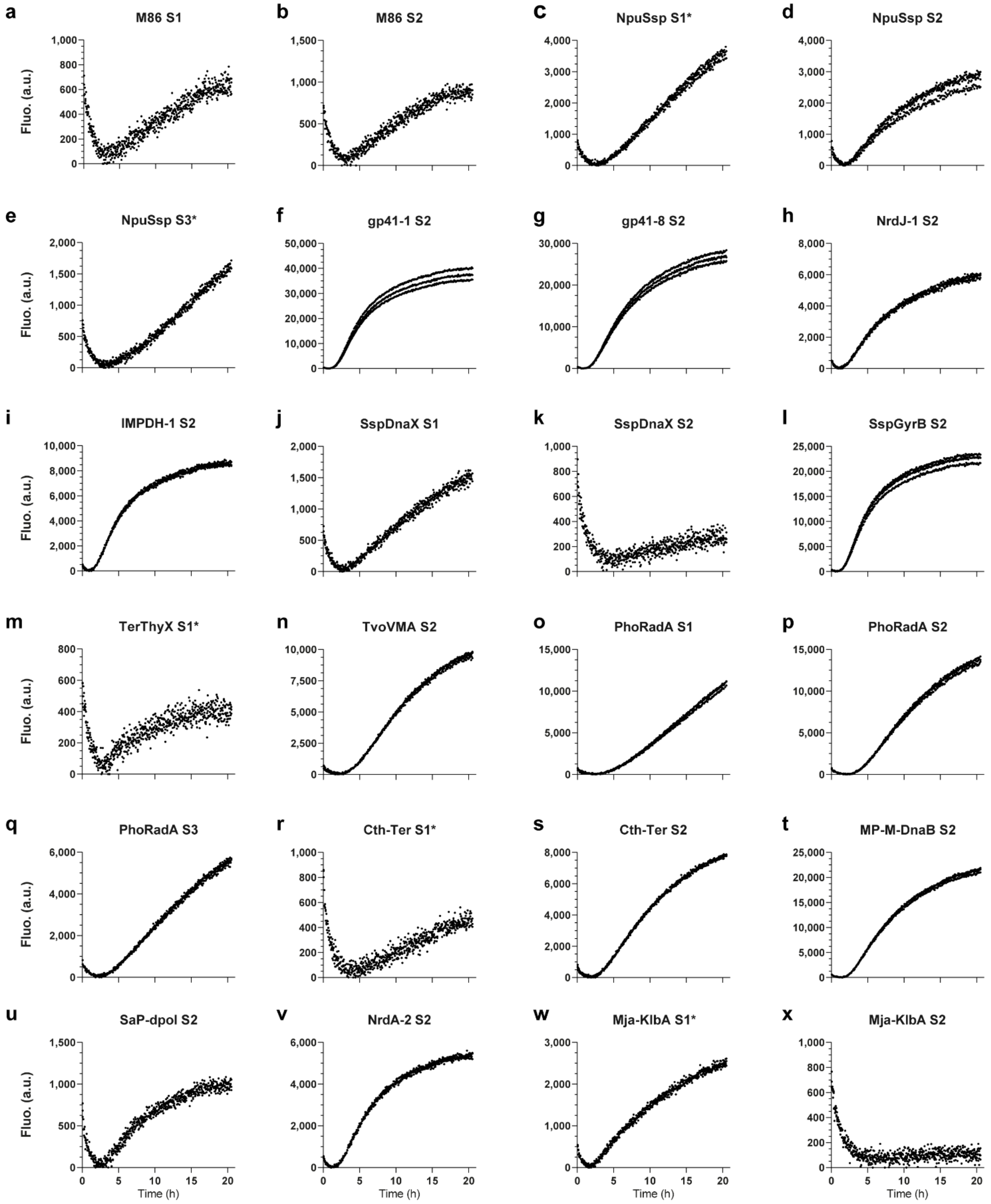

Supplementary Figure 13 | Time course analysis of the *in vitro trans*-splicing of 24 split intein pairs. Cell extracts of *E. coli* cells expressing matching N- or C-terminal mCherry chimeric proteins fused to inteins split at different sites (S1, S2 and S3) were mixed together in the same reaction buffer (100 mM Tris-HCL pH 9.0, 100 mM NaCl, 2 mM DTT) and incubated in a plate reader at room temperature for 20 h 30 min. Fluorescence (Fluo.) was measured every 5 min 24 s and data from three independent replicates are shown. Data was normalized by subtracting the lowest value measured for each well. * - Inteins with a flexible linker at the canonical split site. Source data are provided as a Source Data file.

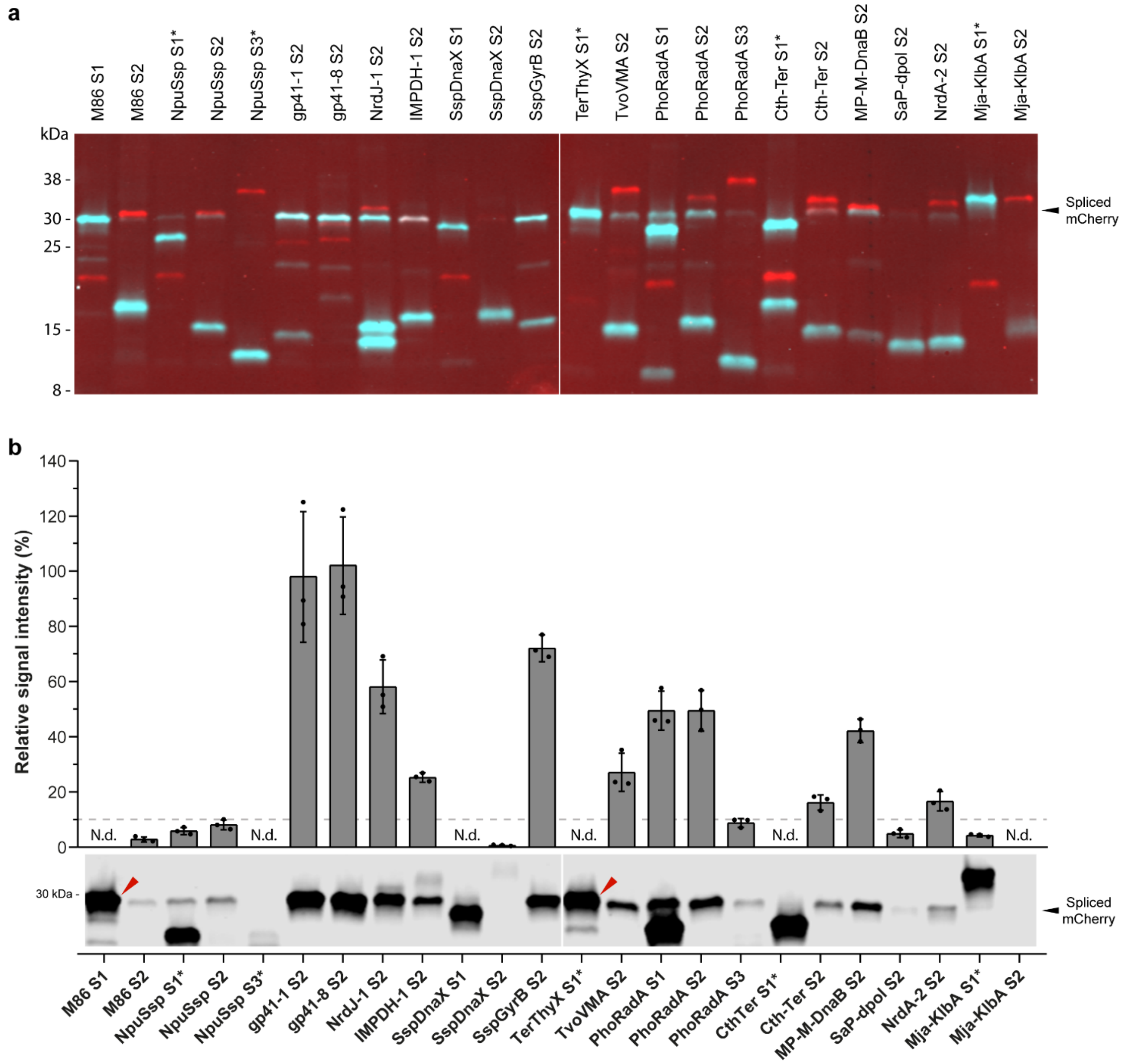

Supplementary Figure 14 | End-point analysis of *in vitro trans*-splicing of 24 split intein pairs. a, Western blot analysis of samples from **Supplementary Figure 13**, collected after 20 h 30 min of incubation. The red signal corresponds to the antibody recognizing the N-terminal of mCherry and the turquoise signal corresponds to the antibody recognizing the hexahistidine-tag (H_6) at the C-terminus. The overlap of both signals (white) corresponds to the spliced mCherry. **b**, Relative spliced mCherry signal intensities (top) as detected by Western blot (bottom). The signal intensities of bands detected with the anti-H_6 antibody were quantified and normalized to the highest signal for each replicate series. Bars represent the mean of the calculated relative signal intensities of the three independent replicates (n = 3) and error bars correspond to s.d. The gray dashed line shows a relative signal intensity of 10%. Red arrowheads indicate bands at the same position as the spliced product but that correspond only to the C-terminal chimeric protein since no signal from the N-terminal of mCherry could be detected. Source data are provided as a Source Data file.

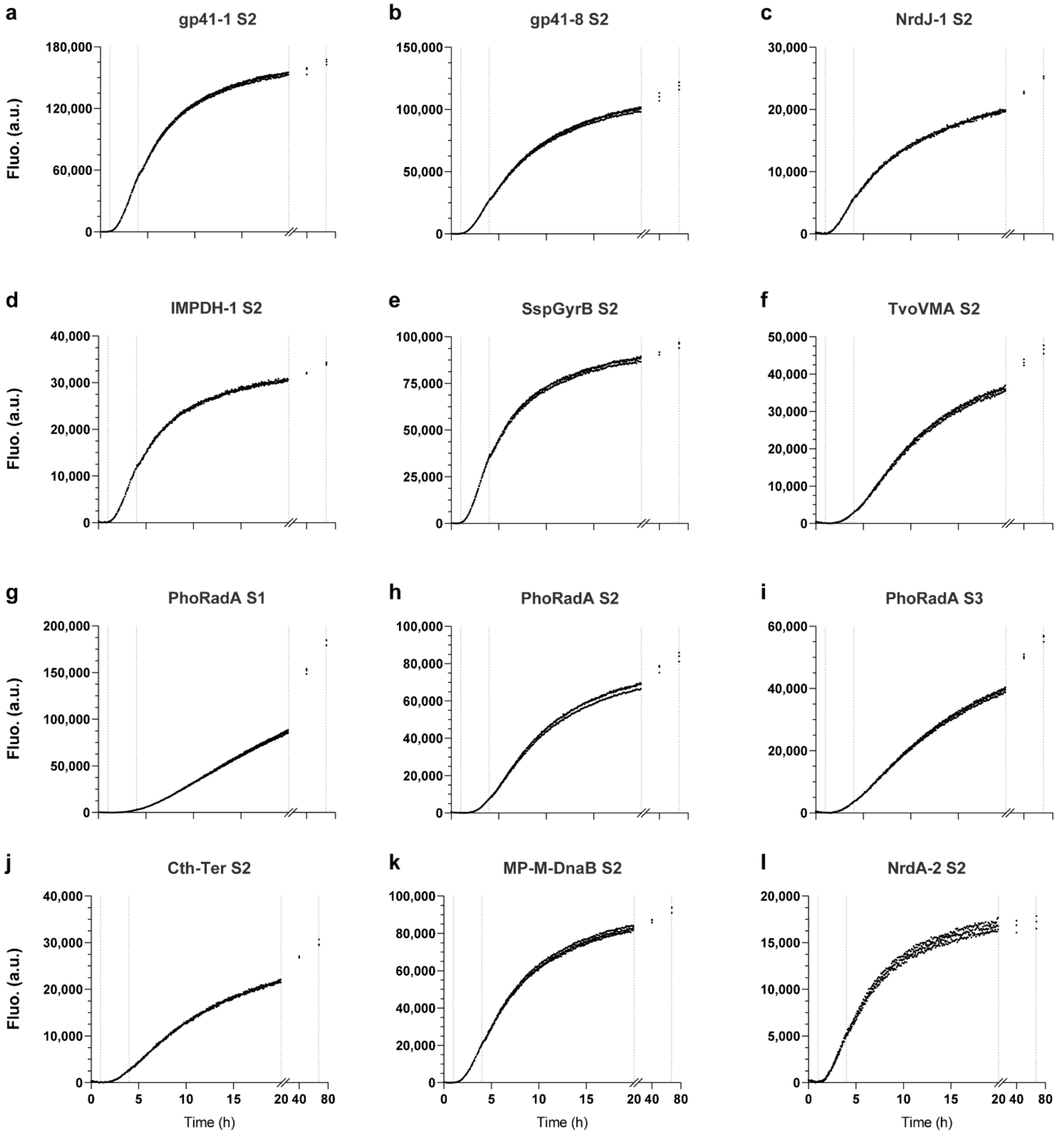

Supplementary Figure 15 | Time course analysis of the *in vitro trans*-splicing of the 12 most active inteins. Cell extracts of *E. coli* cells expressing matching N- or C-terminal mCherry chimeric proteins fused to inteins split at site S2 were mixed together in the same reaction buffer (100 mM Tris-HCL pH 9.0, 100 mM NaCl, 2 mM DTT) and incubated in a plate reader at room temperature for 67 h. Fluorescence (Fluo.) was measured every 5 min 24 s and data from three independent replicates are shown. Data was normalized by subtracting the lowest value measured for each well. Samples for Western blot analysis were collected after 1, 4, 20 and 67 h of incubation (dashed lines). Source data are provided as a Source Data file.

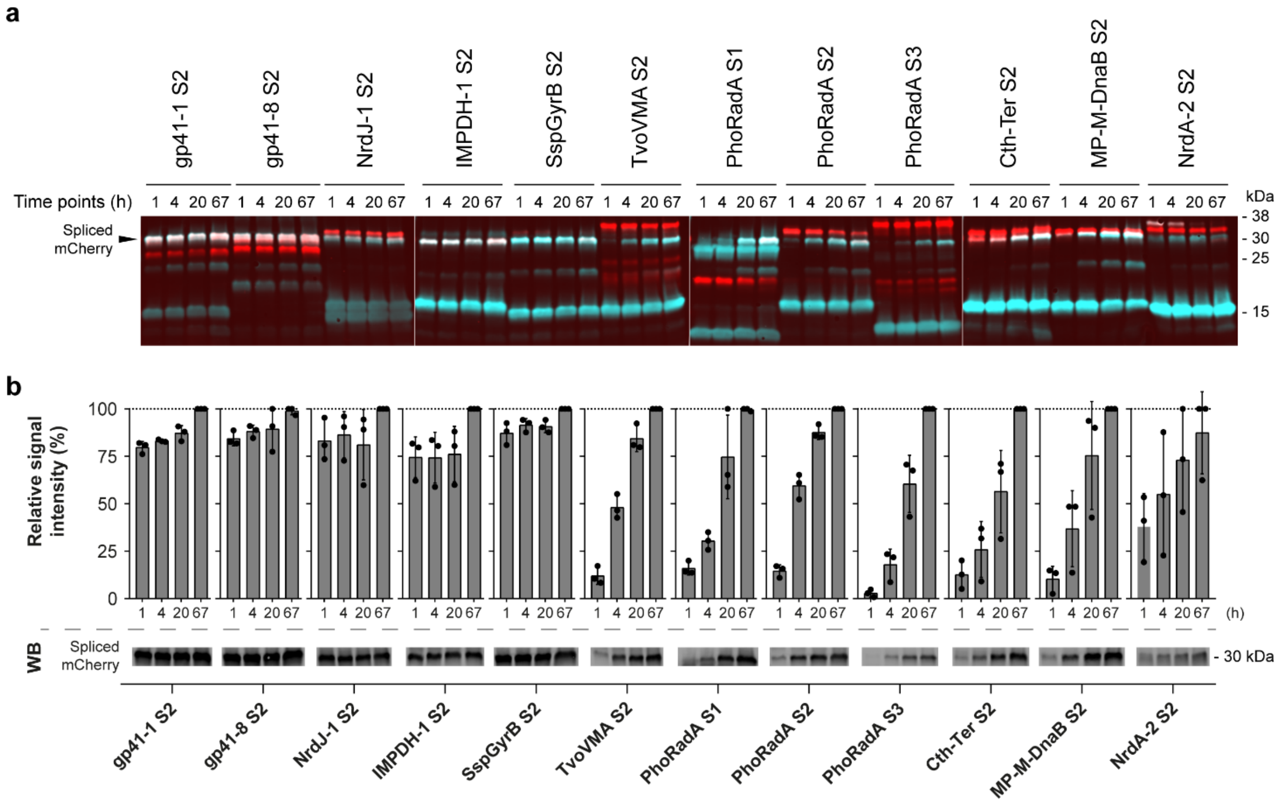

Supplementary Figure 16 | End-point analysis of *in vitro trans*-splicing of the 12 most active inteins. a, Western blot analysis of samples from **Supplementary Figure 15**, collected after 1, 4, 20 and 67 h of incubation. The red signal corresponds to the antibody recognizing the N-terminal of mCherry and the turquoise signal corresponds to the antibody recognizing the hexahistidine-tag (H_6) at the C-terminus. The overlap of both signals (white) corresponds to the spliced mCherry. **b**, Relative spliced mCherry signal intensities (top) as detected by Western blot (bottom). The signal intensities of bands detected with the anti-H_6 antibody were quantified and normalized to the highest signal within the samples from the same replicate and for each intein. Bars represent the mean of the calculated relative signal intensities of the three independent replicates (n = 3) and error bars correspond to s.d. Source data are provided as a Source Data file.

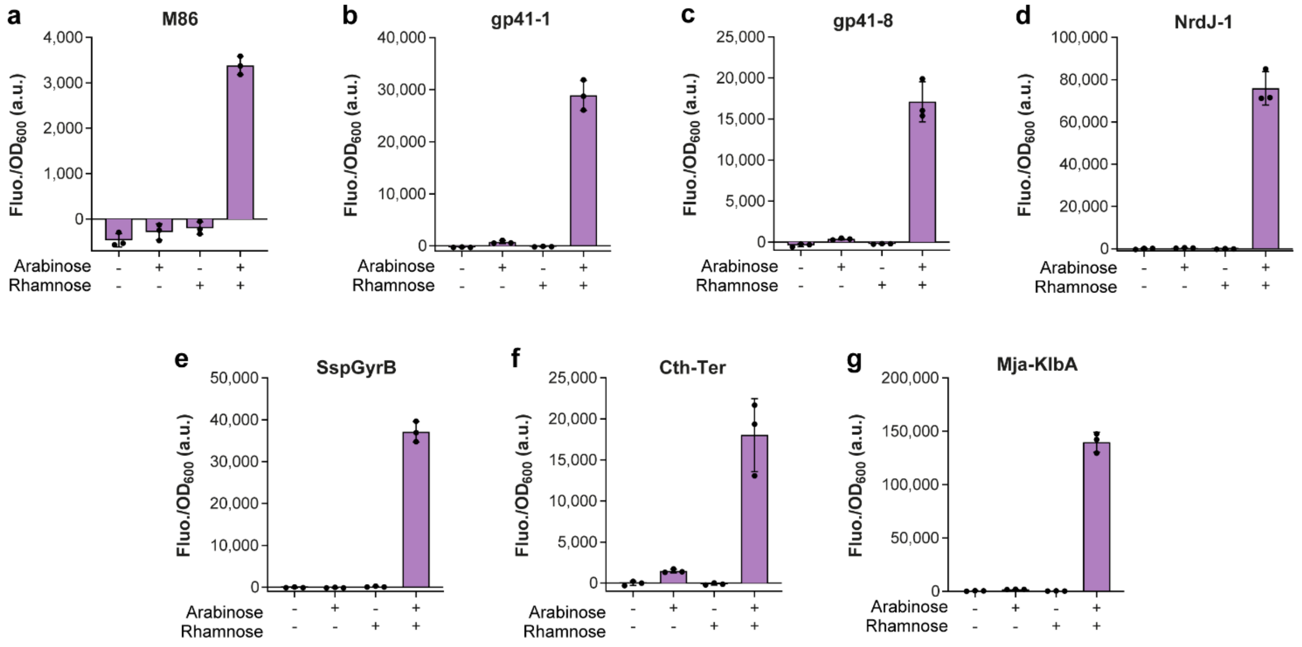

Supplementary Figure 17 | Split mCherry-split intein-based logic AND gates. a-g, Background subtracted fluorescence (Fluo.) of *E. coli* cells harboring the genetic circuits for split mCherry-split intein chimeric proteins, in the absence (-) or presence (+) of inducers (same cultures as in **Fig. 4**). Fluorescence was measured after overnight growth and it was normalized to the cell density (OD_{600}). Bars represent the mean of three biological replicates (*n* = 3) and error bars correspond to s.d. Source data are provided as a Source Data file.

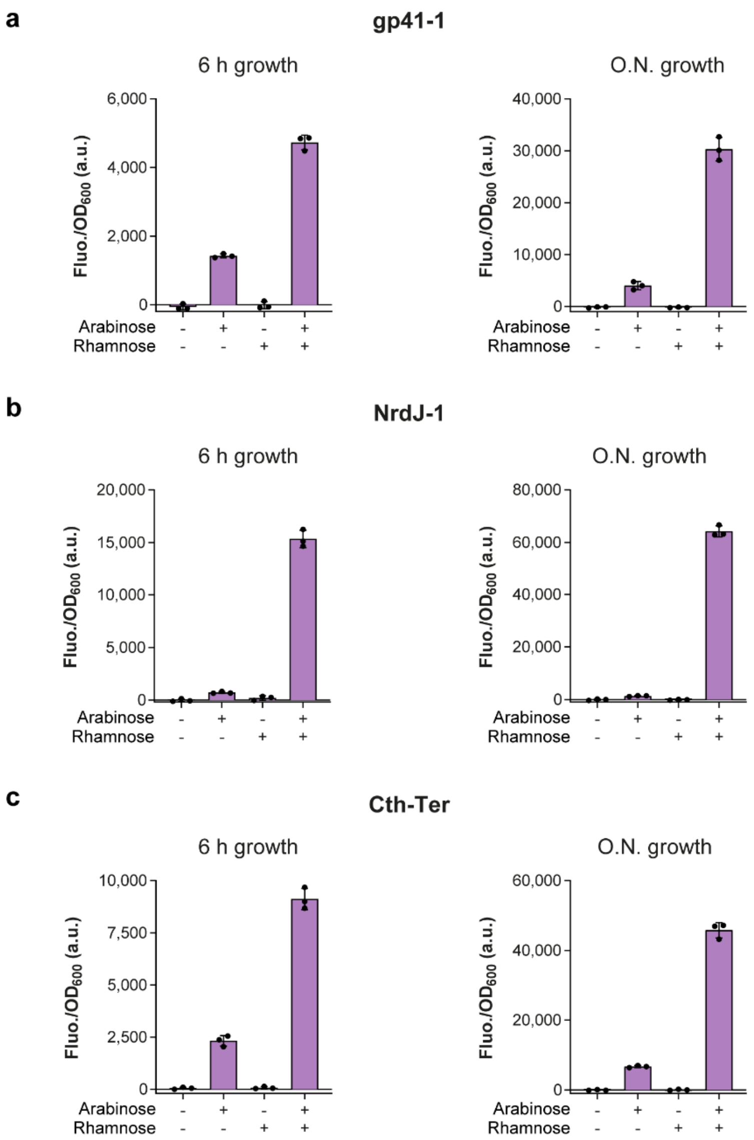

Supplementary Figure 18 | Split mCherry-split intein-based logic AND gates exhibiting C-terminal leakiness. a-c, Background subtracted fluorescence (Fluo.) of *E. coli* cells harboring the genetic circuits for split mCherry-split intein chimeric proteins, in the absence (-) or presence (+) of inducers. All C-terminal halves are controlled by a strong RBS (B0030). Fluorescence was measured 6h after inoculation (left) or after overnight growth (right) and it was normalized to the cell density (OD_{600}). Bars represent the mean of three biological replicates (*n* = 3) and error bars correspond to s.d. Source data are provided as a Source Data file.

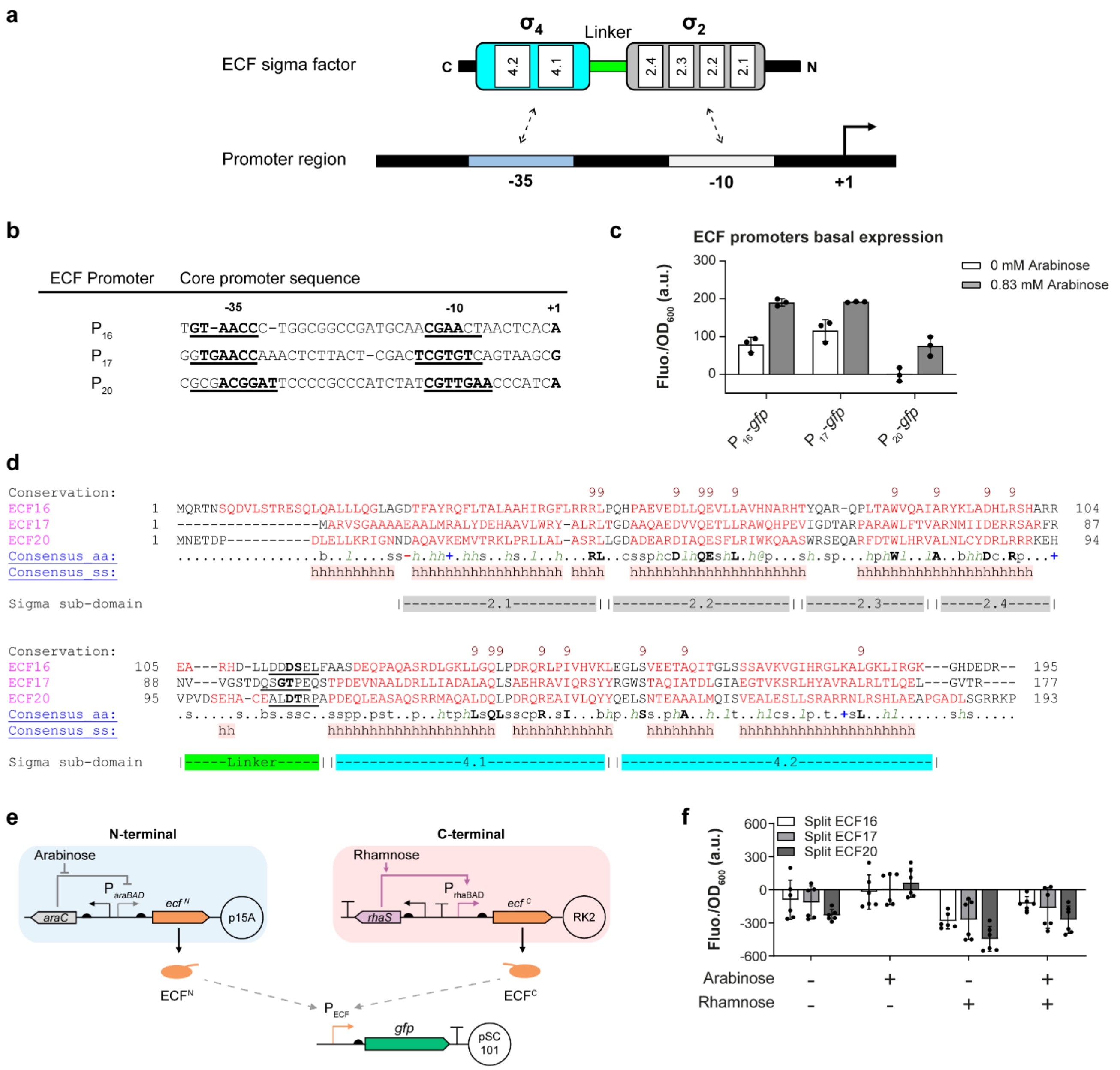

Supplementary Figure 19 | Characterization of split extracytoplasmic function (ECF) sigma factors. a, Schematics of ECFs and cognate promoter structure. ECFs are composed of two domains separated by an unstructured linker. Domain σ_4 interacts with the -35 region of the promoter and the σ_2 interacts with the -10 region. **b**, Alignment of the core promoter sequences of ECF16, ECF17 and ECF20 cognate promoters. The -35 and -10 regions, and the transcription start site (+1) are highlighted. **c**, ECF promoters basal expression. Background subtracted fluorescence (Fluo.) of *E. coli* cells harboring only the ECF cognate promoters upstream of the GFP reporter encoding gene (reporter plasmid) in the absence or presence of 0.83 mM arabinose, normalized to the cell density (OD_{600}). **d**, Structural alignment of ECF16, ECF17 and ECF20 sequences, performed using PROMALS3D (see http://prodata.swmed.edu/promals3d/promals3d.php for output style). The ECF sigma factor sub-domains are indicated. **e**, Genetic circuits for the arabinose- and rhamnose-induced expression of split ECF N- and C-terminal halves, respectively, from two different plasmids. **f**, Background subtracted fluorescence of *E. coli* cells harboring the genetic circuits for the expression of the split ECFs together with the respective reporter plasmids in the absence (-) or presence (+) of inducers. In all the cases, fluorescence was measured 6 h after inoculation and it was normalized to the cell density (OD_{600}). Bars in **c** represent the mean of three biological replicates (n = 3) and in **f** they represent the mean of three biological replicates repeated in two different days (n = 6); error bars correspond to s.d. Source data are provided as a Source Data file.

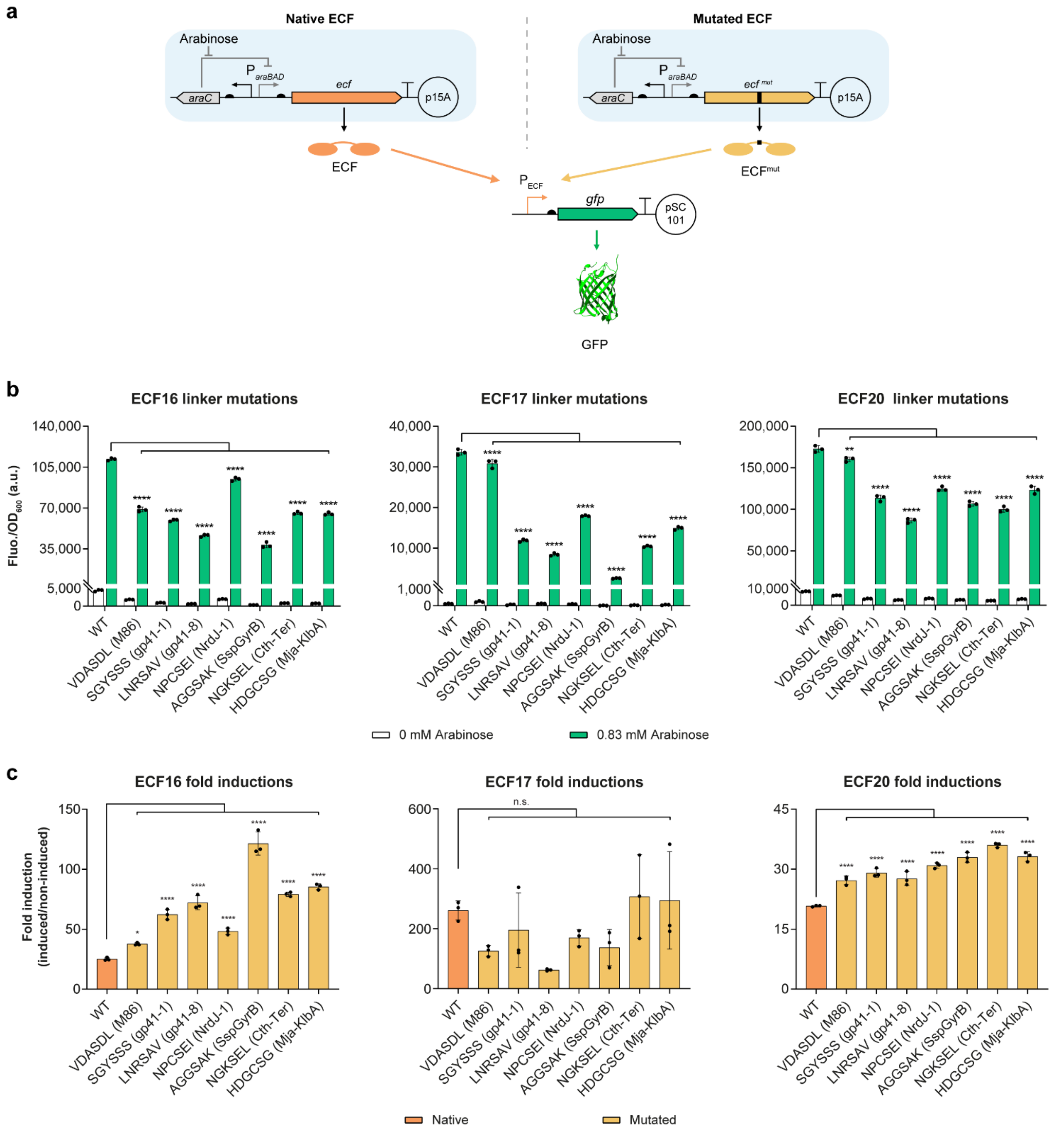

Supplementary Figure 20 | Effect of linker mutation on ECFs activity. a, Genetic circuits for the arabinose-induced expression of native ECFs (WT) or ECFs with the linker region mutated to inteins' preferred junction sequences (ECF^{mut}), in the presence of their respective reporter plasmid (ECF cognate promoters upstream the GFP reporter encoding gene). **b**, Background subtracted fluorescence (Fluo.) of *E. coli* cells harboring the genetic circuits described in **a**, in the absence or presence of 0.83 mM arabinose, normalized to the cell density (OD_{600}). Bars represent the mean of three biological replicates (n = 3) and error bars correspond to s.d. **c**, Fold induction (induced/non-induced) calculated for each native or mutated ECF. Bars represent the mean of the fold induction calculated for the three biological replicates (n = 3) and error bars correspond to s.d. Statistical differences were determined for each ECF (ECF^{mut} vs ECF) by means of two-tailed Student's *t* tests (*P=0.0217; **P= 0.0016; ****P≤0.0001; n.s. non-significant). Non-significant P values are 0.3494 for VDASDL (M86), 0.9104 for SGYSSS (gp41-1), 0.0825 for LNRSAV (gp41-8), 0.7168 for NPCSEI (NrdJ-1), 0.4299 for AGGSAK (SspGyrB), 0.9821 for NGKSEL (Cth-Ter), 0.9971 for HDGCSG (Mja-KlbA). Source data and detailed statistical analyses are provided as a Source Data file.

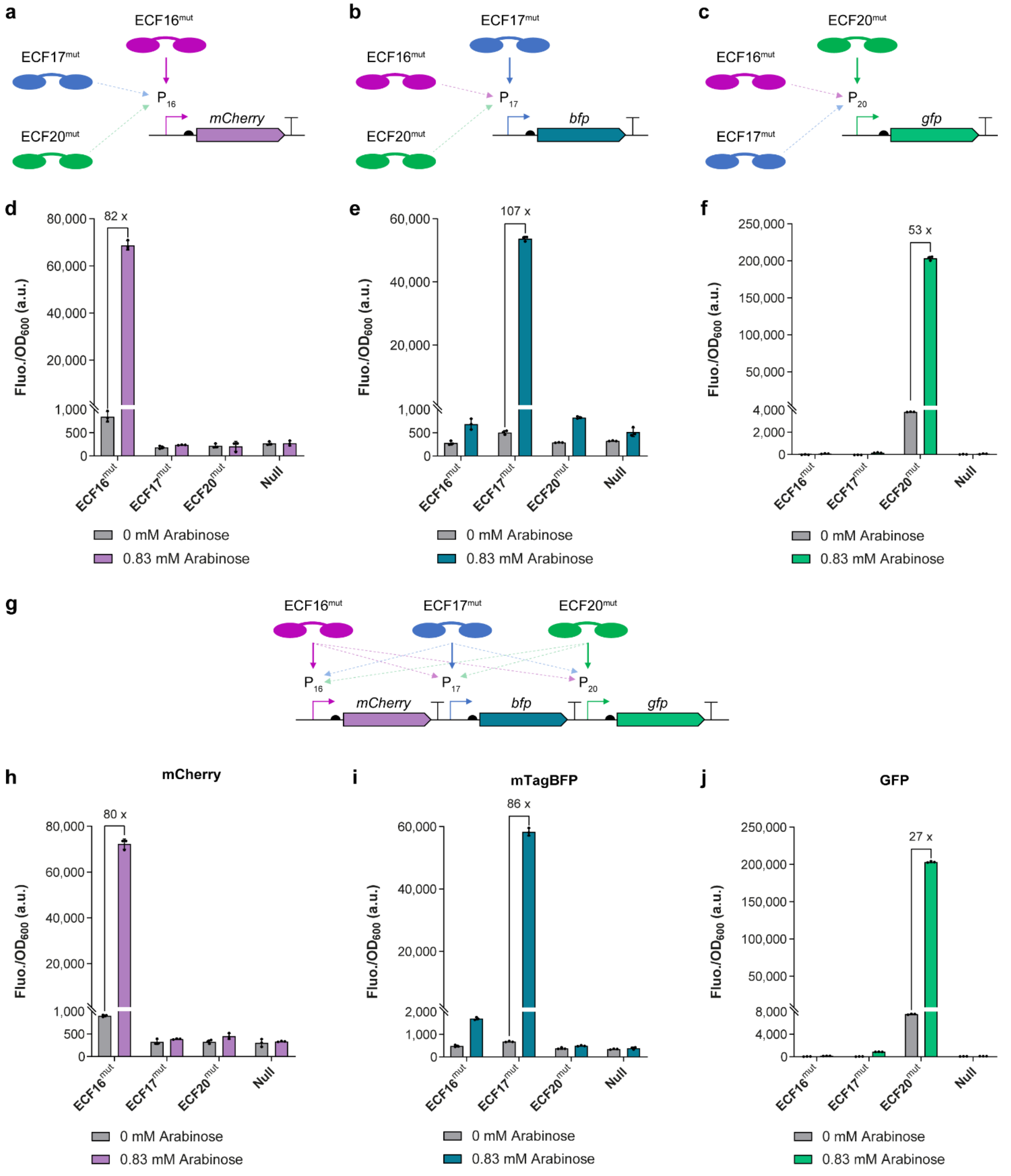

Supplementary Figure 21 | Orthogonality analysis of the mutated ECF sigma factors. a-c, Schematics of ECF^{mut} cognate promoter activation (solid arrows) and cross-reactivity by non-cognate ECFs (dashed arrows) for reporter plasmids containing P_{16}-*mCherry* (**a**), P_{17}-*bfp* (**b**) or P_{20}-*gfp* (**c**). **d-f**, Background subtracted fluorescence (Fluo.) of *E. coli* cells harboring the reporter plasmids containing P_{16}-*mCherry* (**d**), P_{17}-*bfp* (**e**) or P_{20}-*gfp* (**f**) and the plasmids for the arabinose-induced expression of the different mutated ECF sigma factors (ECF^{mut}). **g**, Schematics of ECF^{mut} cognate promoter activation (solid arrows) and cross-reactivity by non-cognate ECFs (dashed arrows) for a reporter plasmids containing the three reporter genes, each under the control of an ECF cognate promoter. **h-j**, Background subtracted red (**h**), blue (**i**) and green (**j**) fluorescence (Fluo.) of *E. coli* cells harboring the 3-reporter plasmid and one of the plasmid for the arabinose-induced expression of an ECF^{mut}. In all the cases, *E. coli* cells containing only the respective reporter plasmids (null) were included as controls. Cells in **d-f** and **h-j** were grown in the absence or presence of 0.83 mM arabinose and fluorescence was measured for each reporter 6 h after inoculation and it was normalized to the cell density (OD_{600}). Bars represent the mean of three biological replicates (n = 3) and error bars correspond to s.d. Source data are provided as a Source Data file.

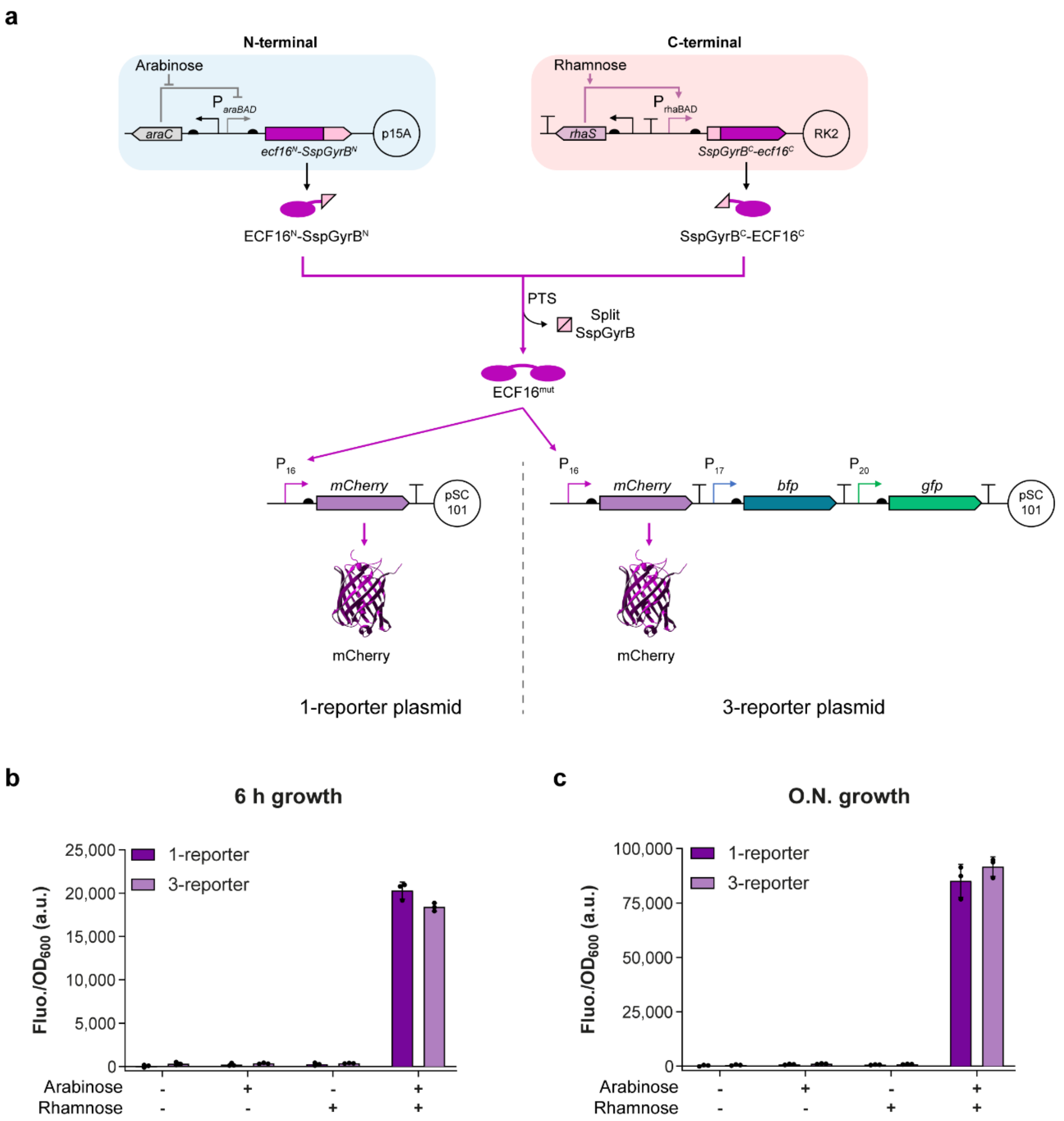

Supplementary Figure 22 | ECF16^{mut} x SspGyrB AND gate characterization. a, Genetic circuits for the arabinose- and rhamnose-induced expression of split ECF16^{mut}-split SspGyrB intein N- and C-terminal halves, respectively, from two different plasmids (top). Schematics of protein trans-splicing with subsequent cognate promoter activation (P_{16}) and mCherry expression after *trans*-splicing, in a 1- or 3-reporter plasmid situation (bottom). Split ECF16^{mut} halves are shown in dark pink and SspGyrB halves in light pink. **b-c**, Background subtracted fluorescence (Fluo.) of *E. coli* cells harboring the three genetic circuits for the expression of chimeric split ECF16^{mut} x SspGyrB halves and expression of the mCherry reporter from P_{16}. mCherry fluorescence was measured 6 h after inoculation (**b**) or after overnight growth (**c**) and it was normalized to the cell density (OD_{600}). Bars represent the mean of three biological replicates (n = 3) and error bars correspond to s.d. Source data are provided as a Source Data file.

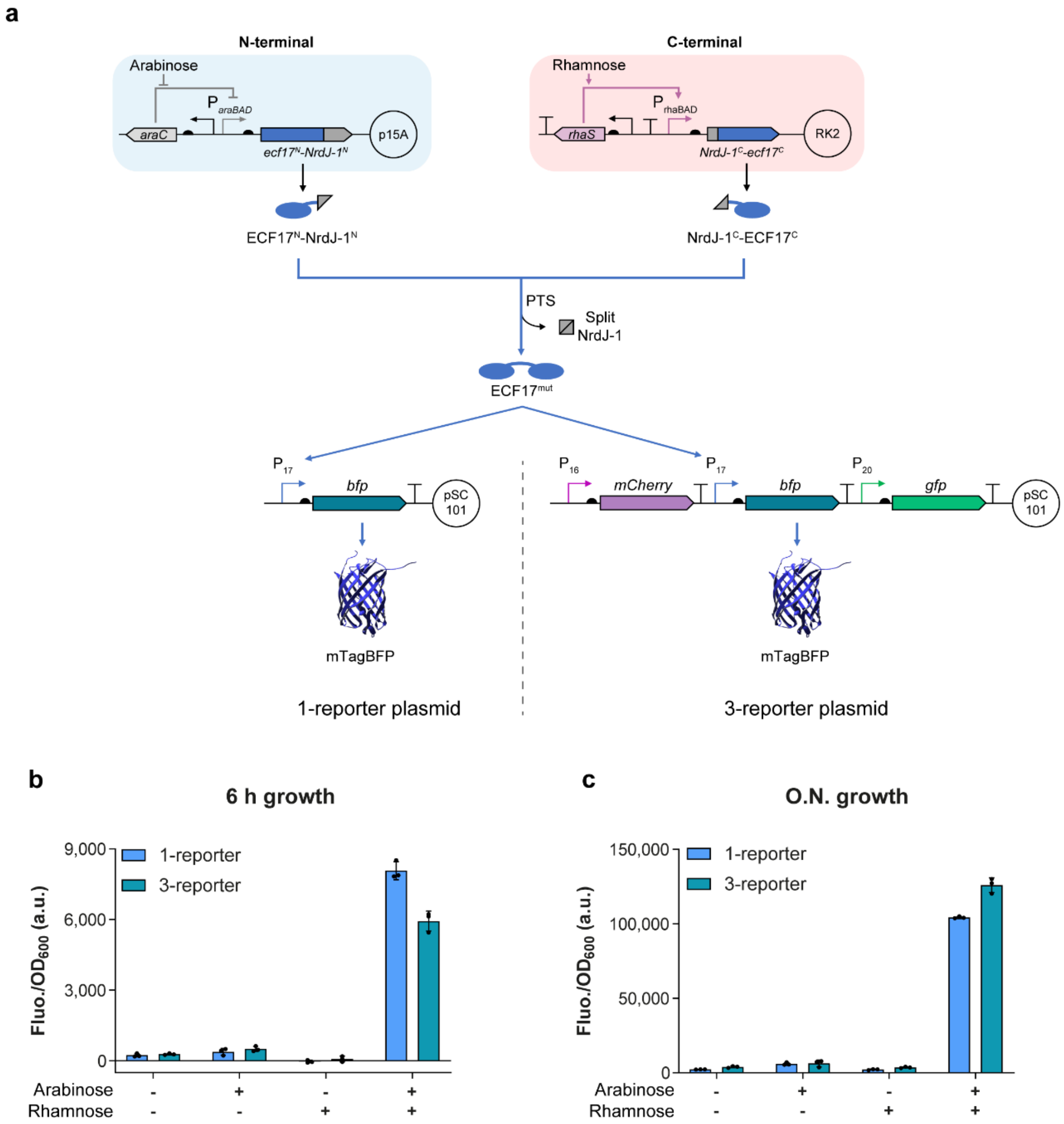

Supplementary Figure 23 | ECF17^{mut} x NrdJ-1 AND gate characterization. a, Genetic circuits for the arabinose- and rhamnose-induced expression of split ECF17^{mut}-split NrdJ-1 intein N- and C-terminal halves, respectively, from two different plasmids (top). Schematics of protein trans-splicing with subsequent cognate promoter activation (P_{17}) and mTagBFP expression after *trans*-splicing, in a 1- or 3-reporter plasmid situation (bottom). Split ECF17^{mut} halves are shown in dark blue and NrdJ-1 halves in gray. **b-c**, Background subtracted fluorescence (Fluo.) of *E. coli* cells harboring the three genetic circuits for the expression of chimeric split ECF17^{mut} x NrdJ-1 halves and expression of the mTagBFP reporter from P_{17}. mTagBFP fluorescence was measured 6 h after inoculation (**b**) or after overnight growth (**c**) and it was normalized to the cell density (OD_{600}). Bars represent the mean of three biological replicates (*n* = 3) and error bars correspond to s.d. Source data are provided as a Source Data file.

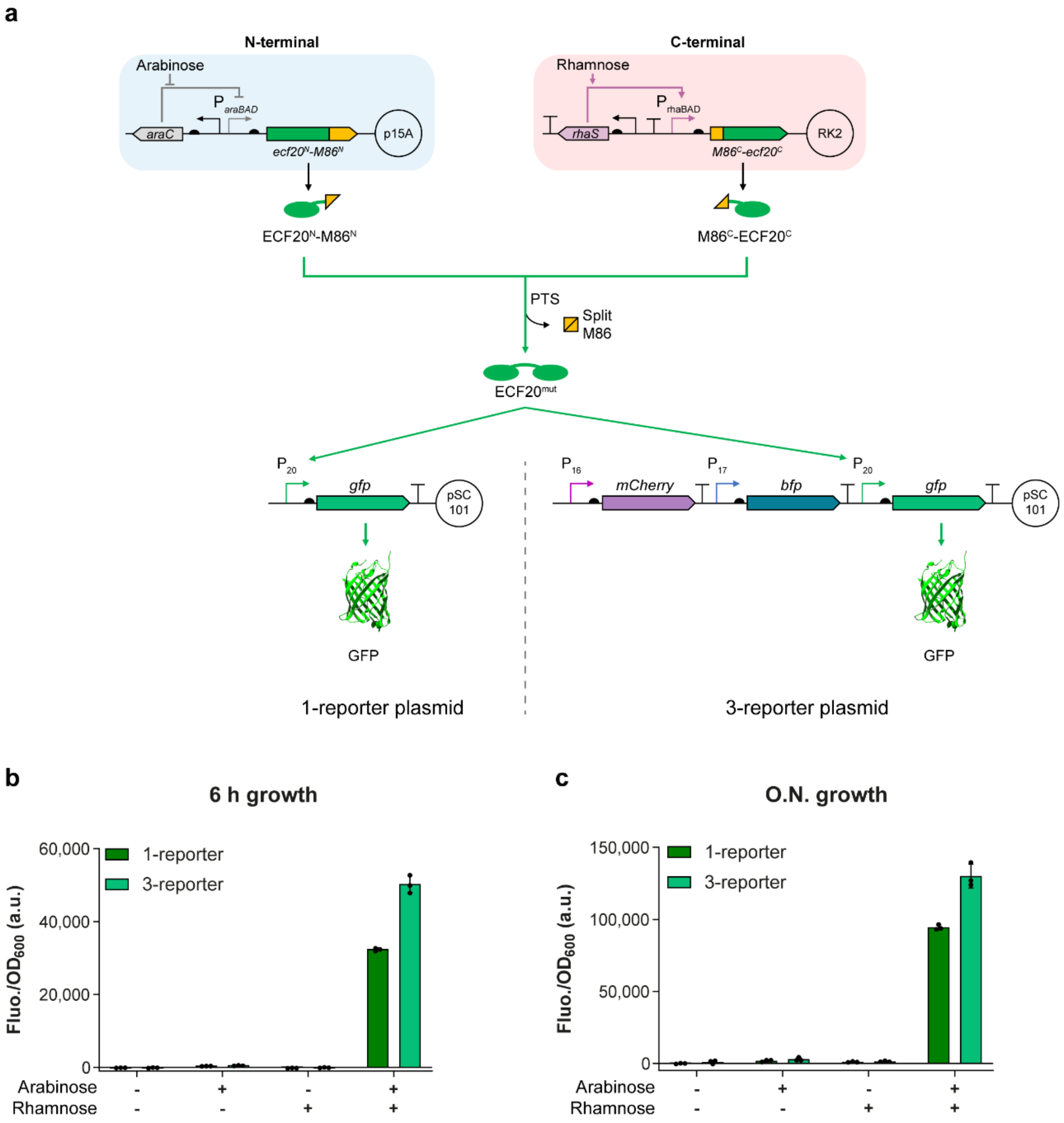

Supplementary Figure 24 | ECF20^{mut} x M86 AND gate characterization. a, Genetic circuits for the arabinose- and rhamnose-induced expression of split ECF20^{mut}-split M86 intein N- and C-terminal halves, respectively, from two different plasmids (top). Schematics of protein trans-splicing with subsequent cognate promoter activation (P_{20}) and GFP expression after *trans*-splicing, in a 1- or 3-reporter plasmid situation (bottom). Split ECF20^{mut} halves are shown in green and M86 halves in yellow. **b-c**, Background subtracted fluorescence (Fluo.) of *E. coli* cells harboring the three genetic circuits for the expression of chimeric split ECF20^{mut} x M86 halves and expression of the GFP reporter from P_{20}. GFP fluorescence was measured 6 h after inoculation (**b**) or after overnight growth (**c**) and it was normalized to the cell density (OD_{600}). Bars represent the mean of three biological replicates (*n* = 3) and error bars correspond to s.d. Source data are provided as a Source Data file.

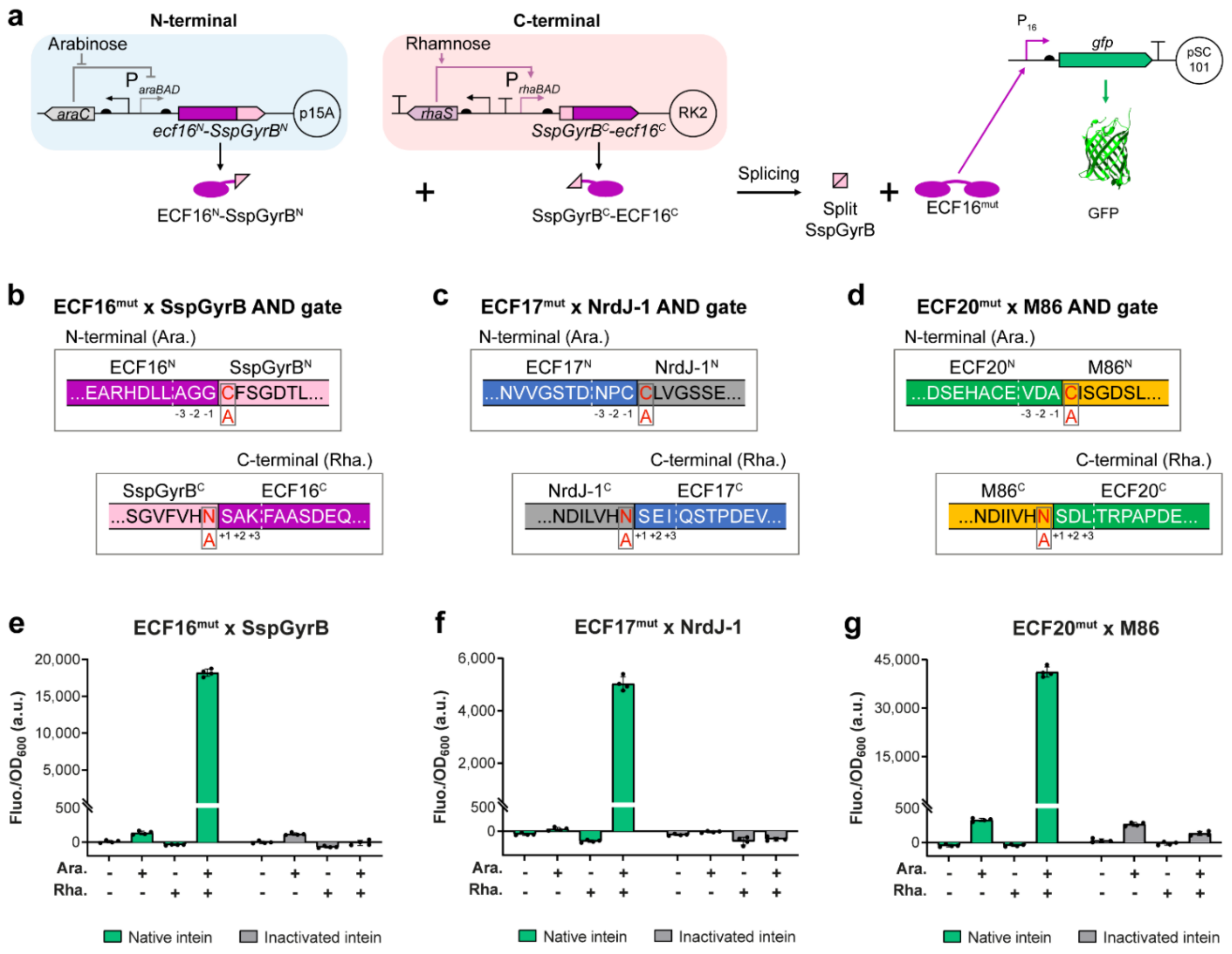

Supplementary Figure 25 | ECF-based AND gates depend on inteins' splicing activity. a, Genetic circuits for the arabinose- and rhamnose-induced expression of split ECF^{mut}-split intein N- and C-terminal chimeric proteins, respectively, from two different plasmids (top, left), and schematics of protein *trans*-splicing (bottom), exemplified using ECF16^{mut} and the SspGyrB intein. Split ECF16^{mut} halves are shown in dark pink and SspGyrB halves in light pink. After splicing, the reconstituted ECF16^{mut} can activate its cognate promoter (P_{16}) and the fluorescent reporter (GFP) is expressed (right). **b-d**, Sequence details of split ECF x split intein chimeric proteins, showing the junction regions for ECF16^{mut} x SspGyrB (**b**), ECF17^{mut} x NrdJ-1 (**c**) and ECF20^{mut} x M86 (**d**) AND gates. The junction sequence amino acid residues (-3, -2, -1 and +1, +2, +3) are separated by a dashed line and the inteins' catalytic residues (at both N- and C- termini) are in red. Inactivated inteins were produced by mutating both catalytic residues to alanine (A). **e-g**, Background subtracted fluorescence (Fluo.) of *E. coli* cells harboring the genetic circuits for the expression of the native (green) or inactivated (gray) intein split ECF-based AND gates: ECF16^{mut} x SspGyrB (**e**), ECF17^{mut} x NrdJ-1 (**f**) and ECF20^{mut} x M86 (**g**). Cells were cultivated in the absence or presence of arabinose (Ara., 0.83 mM) and rhamnose (Rha., 15.3 mM) as annotated under the graphs. GFP fluorescence was measured 6 h after inoculation and it was normalized to the cell density (OD_{600}). Bars represent the mean of four biological replicates (n = 4) and error bars correspond to s.d. Source data are provided as a Source Data file.

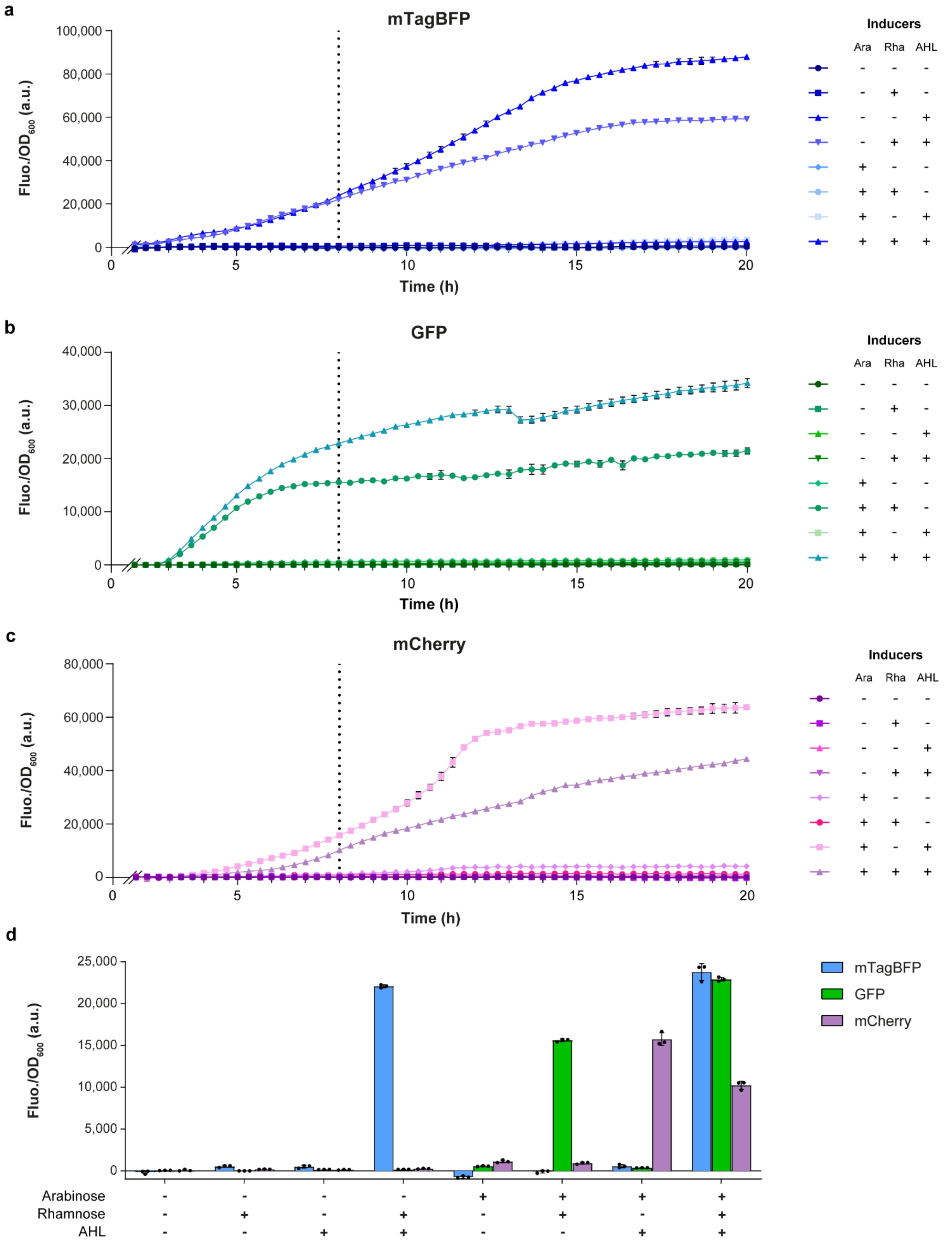

Supplementary Figure 26 | 3-input/3-output integrated circuit characterization. a-c, Time course analysis of mTagBFP (**a**), GFP (**b**) and mCherry (**c**) fluorescence (Fluo.) in *E. coli* cells carrying the integrated logic circuit and exposed to the eight possible input combinations (-, absence of input; +, presence of input). Measurements were taken every 20 min and data represents mean values of background subtracted fluorescence, normalized to the cell density (OD_{600}). Data in **a-c** denotes the mean of three biological replicates (n = 3) and error bars correspond to s.d. The dotted vertical lines represent the time-point analyzed in **d** (8 h after inoculation). **d**, Background subtracted Fluo./OD_{600} of mTagBFP, GFP and mCherry, measured 8 h after inoculation for each combination of inputs. Bars represent the mean of three biological replicates (n = 3) and error bars correspond to s.d. Source data are provided as a Source Data file.

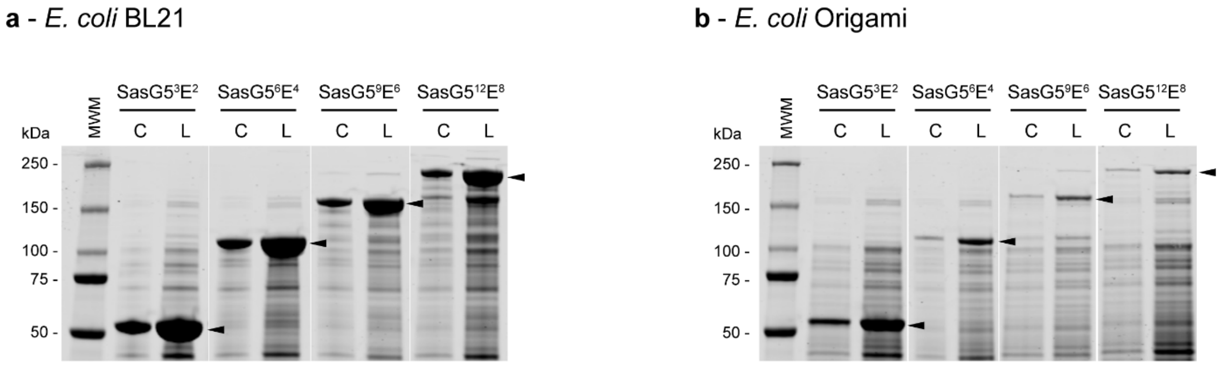

Supplementary Figure 27 | Full-length SasG-based proteins expression analysis. a-b, SDS-PAGE analysis of BL21 (**a**) or Origami (**b**) *E. coli* cells expressing the full-length SasG-based proteins including final junction sequences. Whole cells (C) or clear lysates (L) were analyzed. Arrowheads indicate the recombinant proteins. MWM - Molecular weight marker. The proteins' theoretical molecular weights can be found in **Supplementary Table 9**. Protein gel images are representative of two independent experiments with similar results. Source data are provided as a Source Data file.

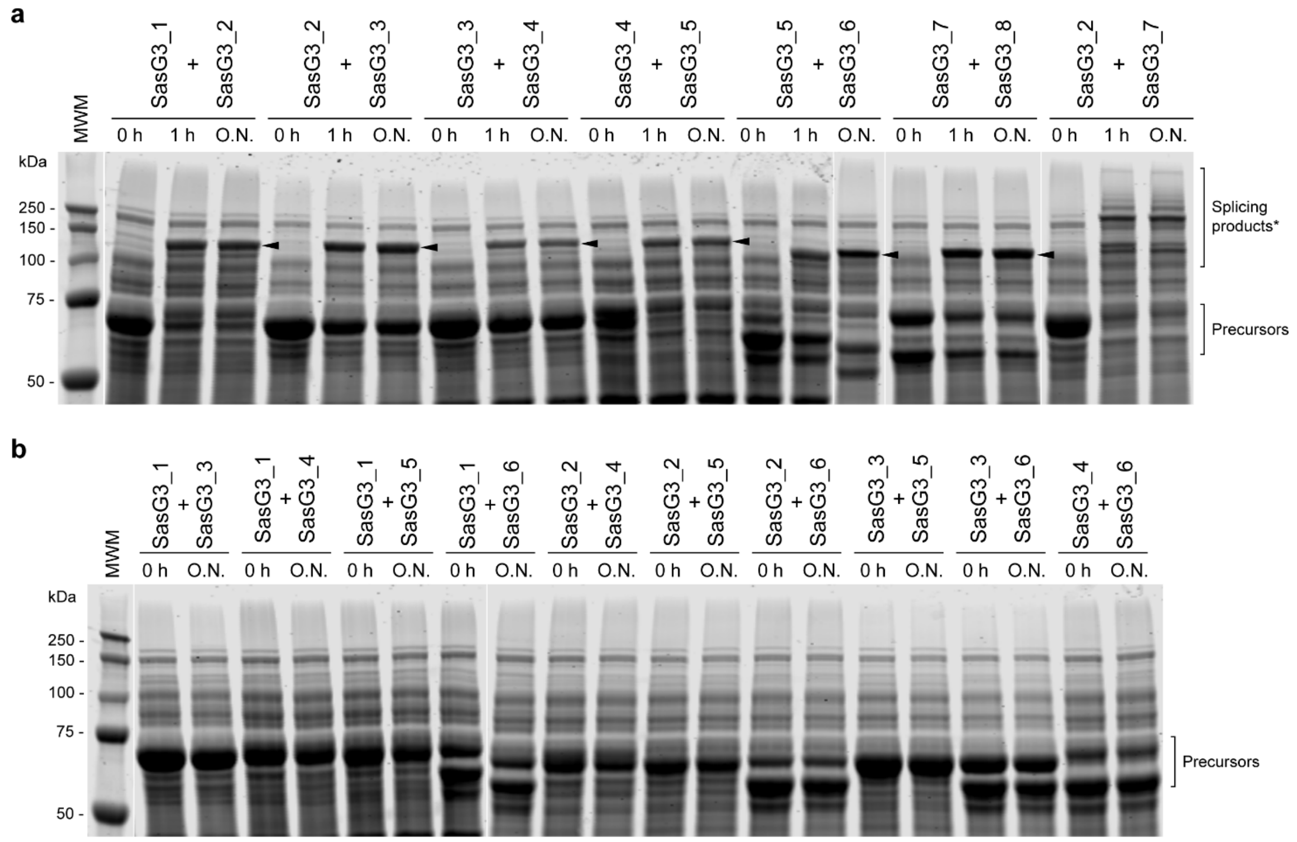

Supplementary Figure 28 | SasG3 assembly units *trans*-splicing and cross-reactivity assessment. a, SDS-PAGE analysis of *trans*-splicing reactions between corresponding SasG assembly units containing split inteins. For each pair, non-reacted (0h), reactions incubated for 1 h and reactions incubated overnight (O.N.) were analyzed. Expected splicing products are indicated by arrowheads. Assembly units SasG3_2 and SasG3_7 react indefinitely originating multiple splicing products (*). **b**, SDS-PAGE analysis of cross-reactivity between SasG assembly units containing unrelated split inteins. For each pair, non-reacted (0h) or reactions incubated overnight (O.N.) were analyzed. MWM - Molecular weight marker. Protein gel images are representative of three independent experiments with similar results. Source data are provided as a Source Data file.

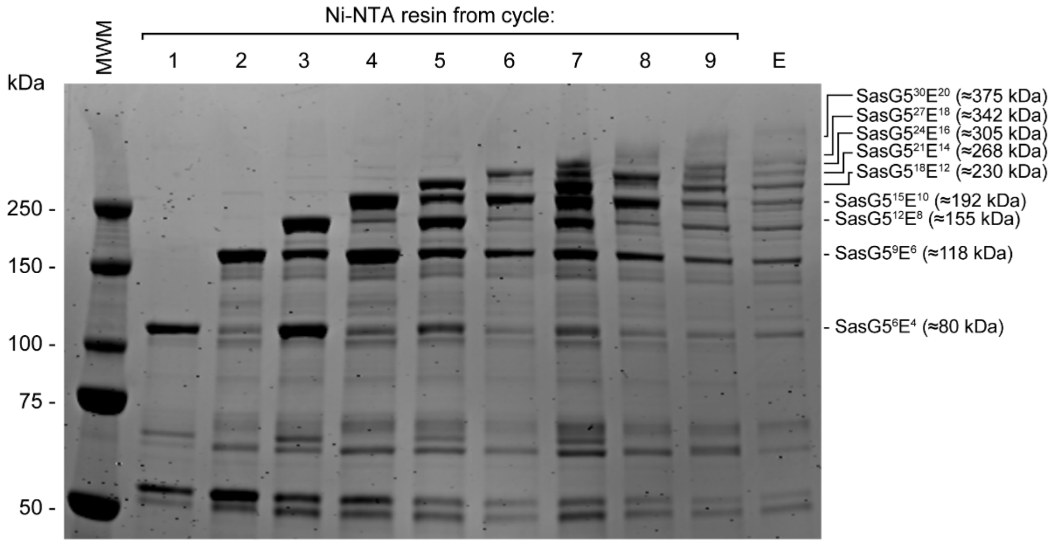

Supplementary Figure 29 | *In vitro* production of up to SasG5^{30}E^{20} by recursive solid-phase assembly. SDS-PAGE analysis of the Ni-NTA resin after each cycle of recursive solid-phase assembly (1-9) and final protein elution (E). The full-length final protein and other side products resulting from incomplete splicing reactions are indicated. MWM - Molecular weight marker. The protein gel image is representative of three independent experiments with similar results. Source data are provided as a Source Data file.

Supplementary Table 1 | List of viral and viral-like inteins retrieved from InBase

Intein	Extein	Organism	Protein sequence
APMV Pol	DNA polymerase B (alpha or family B)	*Acanthomoeba polyphaga* Mimivirus	SVTGDTPIITRHQNGDINITTIEELGSKWKPYEIFKAHEKNSNRKFKQQSQYPTDSEVWTAKGW<br>AKIKRVIRHKTVKKIYRVLTHTGCIDVTEDHSLLDPNQNIIKPINCQIGTELLHGFPESNNVYD<br>NISEQEAYVWGFFMGDGSCGSYQTKNGIKYSWALNNQDLDVLNKCKKYLEETENIQFKILDTMK<br>SSSVYKLVPIRKIKYMVNKYRKIFYDNKKYKLVPKEILNSTKDIKNSFLEGYYAADGSRKETEN<br>MGCRRCDIKGKISAQCLFYLLKSLGYNVSINIRSDKNQIYRLTFSNKKQRKNPIAIKKIQLMNE<br>TSNDHDGDYVYDLETESGSFHAGVGEMIVKN
CV-NY2A RIR1	Ribonucleoside-diphosphate reductase, alpha subunit	*Chlorella* virus NY2A infects *Chlorella* NC64A, which infects *Paramecium bursaria*	CVAGDTKILTSSGYHPIKDMEGKQVRVWNGYEFSETIVHKTGVNQKLIMVSLDDGTELRCTPYH<br>KFYIETGSRPADKSRVMEVRAGDLEKGDRIIRFELPTITVGETTMSDKEAYTKGFFSADGCVIK<br>SKYGEDEYRISVKREDKIEALTKYVDVIKSHTNRFRTHFYVPDYVQNKFEVPINSMVNEKISWL<br>AGFMDGDGCVIRYKDIENMQAVSINKSFLQDIRLMLQTIGIHSTINKFMPNRVMKMPDGRGGTD<br>MYNGAESWRLQIDSEGVRKLFALGFTPRRLKMNGSRKRHHKTNKFTRVVSVTDHGDVEDTYCFN<br>EPKRHMGVFNGVITGQ
CV-NY2A ORF212392	ORF212392	*Chlorella* virus NY2A infects *Chlorella* NC64A, which infects *Paramecium bursaria*	CLHPETDVITFSGTVIKAKDLRPGMQLMGLDSTPRRVLDIGRGREMMYEIVPIKGEPWKCNETH<br>VLSLIYNEHGSIQKTKSNTYFVKFHEYTSDGRGVFKYPTVKTLKEAQDIVARLNPNHIVDIPLN<br>EYLNLPKHVKNSLKLFRSGPITFPNKPEPKFHPYILGAWLGDGKSSTAEITIDVKEKPLLNHVS<br>NLLRPLGMIASKRTGASSLKRTDHYRIQYPDNNSRKGKSNPFMDALKSYDLISNKHIPDDIKCG<br>SLNVRLQILAGLLDTDGYLGGNCFEITQKNKRLSEDIAFVARSLGYAAYLKMVKKSCTYKGEKK<br>CGTYYKVSISGEGLEKIPTILERKCAKPRQQVKDARRTGFTVRKLSVGDYVGPVLDKDHRYLLG<br>DFTVTHN
CroV RIR1	Ribonucleoside-diphosphate reductase, alpha subunit	*Cafeteria roenbergensis virus BV-PW1*	CFTPDTPIFTNDGFVSIENIKPHMKVMTSDGTFRNVNKIFKNNVNKNILKINTTHSLEEIKCTK<br>EHDILIYQNINNESNYEQITHYIETNKYTPNFVKASELKVGDFMVIPKIQINKQTIYSEDDYYL<br>LGLILGKGTIVLNKDYHLMECMLTLDTNSISYQFVKNYLTTKNICFSEINNNSIIEWNLPENFI<br>IQYDDLYINDIKYFSSKFITGETNKLLKLIKGLIDSNGNIDREITVKASNKNMAYSIRYILMIL<br>GIPSSGHFTDNYIIKIPKTNMISNILNIEPDNTFNYIELETCILTKIENIDMCEYSGYVYDLNI<br>EENHNYLTSSGIVHN
CroV Pol	DNA polymerase (alpha or family B)	*Cafeteria roenbergensis* virus BV-PW1	SVIGDTPLLLKNKFTNEILINKIKDLSSNWSNYHNGKESCEIDTYQTWTETGWTDIKRVIRHKL<br>ESNKKLLKIQTHNGEVIVTDEHSLLNKNGKTINAKNVKVGDNILHSFPSYINNIDNTNSINYHN<br>KFYNKKMCNELAYILGCFMKYGLCDSSKKCFTINNKDINLIESLKKMCENIFDEFKWKISSSSH<br>LSDNIYKLVPFQNEIKLIDFIKYFTNKMYNNGEKKVPQCILNSSKEYIKIFLIGLYPEYKLENN<br>QQFIYTCKNNEFSLGIYYLIKKLGYHVKLNSNDSSDSSDSIYTFEISHKLENNNNVITKITEWE<br>HKETYVYDLTTENHHFHAGVGSIIVHN
CroV RPB2	DNA-directed RNA polymerase beta (II) subunit 2	*Cafeteria roenbergensis* virus BV-PW1	CQKYDTLVLTHLGWIKLGEIDITIHKVATLDKHDNIIYVYPTSKFEFDYDGDFYEHKNNSIDIE<br>CTINHKLYCKYNLSSSFTLIPADKVYGNKVIMKNMENEVIWTDPSKEQIHNYKGKVYCIEVPDS<br>HIYYMKTSSITPPVWIGN
CroV Top2	DNA Topoisomerase IIA	*Cafeteria roenbergensis* virus BV-PW1	CISSDTNVLIWNSKISKKAKDIQIGDILVGDDGNKRNVIDVSFGKGQMYKIIQSKGENYSVNNN<br>HTLTLMMPLHKVISNSNNKLKLLWWDNVYKIIKSKPIDVQENKISDDEILLNSVYHDRTKHYRK<br>VRKESQIYTLAEIDQLNLSDEIKKEIIDLCNTIPDENVFDINIQDYMNLDKTTQTYLIGVSGKC<br>INYPYKSVNIDPYLLGLWIGEDCSHNSKIYLNNRVNLNMYKLKSKGIPDDYLYNNKYIRESVLA<br>GIIDKKGSVVRNGTKIIIEQKLSNKELINDIIYLAQSLGFVCSTANINKTITTTGVTHSDNIKT<br>IYITGNLASIPVLREKNKCINPTNFNILDSPGYITVEKDGIGDYVSITVDTTTNQRFLINDFTV<br>THN
CZIV RIR1	Ribonucleoside-diphosphate reductase, alpha subunit, RNR, class I	*Costelytra zealandica* iridescent virus	CVVGETLVLTQNGQFPIKYLVNQYVKVWNGEEWSEVVVKKTGFNQELVRVDFNNGTSIICTPYH<br>NFLSLEDDSTIKNLKRICAQDLPCNFKVLYFYQNLDFVTTVKVTKITHLSQRADTYCFSEPLNN<br>AGVFNGILTGQ
HaV01 Pol	DNA polymerase B (alpha or family B)	*Heterosigma akashiwo* virus 01	SVTKETPLMLRTMETCGNHKHEVISIENVFTDNMRSIDMYSIIGEKEHVMLSRNEEIWTGENWS<br>RIIRVIRHKTQKKIYGVLTENGYVEVTEDHSLISSDYELLKPKNCIVKETQLLQSFPDIVENST<br>IENNMIDIPKGQPCRLTVFGQVSAMIIYTYLKRKNYSITLNVCNVNSNKFYISFMERPRFKNTK<br>KNIIKKIFFIRNTDNEEYVYDVETEDGIFHAGIGEIIVKN
CIV RIR1	Ribonucleoside-diphosphate reductase, alpha subunit	*Chilo iridescent* virus	CVAPETMILTEDGQFPIKDLEGKIIKVWNGNEFSSVTVVKTGTEKELLEVELSNGCTLSCTPEH<br>KFIIVKSYTEAKKQKTDDNAIANAERVDAQDLKPRMKLIKFDLPTLFGNSEHDIKYPYTHGFFC<br>GDGTYTKYGKPQLSLYGDKKELLTYLDVRTMTGLEDASGRLNTWLPLDLAPKFDVPINSSLECR<br>MEWLAGYLDADGCVFRNGTNESIQVSCIHLDFLKRIQLLLIGMGVTSKITKLHDEKITTMPDGK<br>GGQKPYSCKPIWRLFISSSGLYHLSEQGFETRRLKWEPRQPQRNAERFVEVLKVNKTGRVDDTY<br>CFTEPINHAGVFNGILTGQ
IIV6 RIR1	Ribonucleoside-diphosphate reductase, alpha subunit	Invertebrate iridescent virus 6	CVAPETMILTEDGQFPIKDLEGKIIKVWNGNEFSSVTVVKTGTEKELLEVELSNGCTLSCTPEH<br>KFIIVKSYTEAKKQKTDDNAIANAERVDAQDLKPRMKLIKFDLPTLFGNSEHDIKYPYTHGFFC<br>GDGTYTKYGKPQLSLYGDKKELLTYLDVRTMTGLEDASGRLNTWLPLDLAPKFDVPINSSLECR<br>MEWLAGYLDADGCVFRNGTNESIQVSCIHLDFLKRIQLLLIGMGVTSKITKLHDEKITTMPDGK<br>GGQKPYSCKPIWRLFISSSGLYHLSEQGFETRRLKWEPRQPQRNAERFVEVLKVNKTGRVDDTY<br>CFTEPINHAGVFNGILTGQ
WIV RIR1	Ribonucleoside-diphosphate reductase, alpha subunit, RNR, class I	*Wiseana* iridescent virus	CVVGETLILTENGEYPIKSLVDREVSVWNGDEWSDVTVVQTGTDQELLRIDFSNGIFIVCTEYH<br>RFLVLDRSRPIKDLKRKYAKDLPLNFQVMYTHSDLSTTLIKVTKVSKLQRRANTYCFTEQLNNA<br>GVFNGILTSN
CP-P1201 Thy1	Putative thymidylate synthase	*Corynebacterium* phage P1201	CYSSDTEVLTSEGWKRWEEVSMNDSFATLSASGQVEYQYPSEVIQEEYQGDMVRAKAKGVDLLV<br>TPNHKMLACITTTRAGRRKENFSLIPADQLIGVSHAYKKDGDWRASGSGAGEDFAWLVGFFAGD<br>GSIQGKWDNQIEFHMSKARKMSALSKAIQALGWKMSARYEKQREDTGHFSIFLPEDKVEFFRSF<br>YDEDSQKRIPQEFIMSLNKEESLALLQGMIDSDGSVTNESGTGRVFDSTSKSLRDAFSQVALHA<br>GFAANEVEGSTVIAGTEVEINGMKTTLARDCKRVSLSERNLRPEIGKKDSGASKTSYGWEKYSG<br>TVYCATVPNHTLYVRRNGKPVWSGN

AP-APSE1 dpol	DNA polymerase A (Pol I or family A)	*Acyrthosiphon pisum* secondary endosymbiot phage 1	CLAKGTLVLTITGWMPIEIVSQDAYVWDGIEWVRTDGSVFNGNQEVIQAYGVGMTADHQVLTEK<br>GWKSASQSKRYNRSSCRLPDGYKLPRFRRKEINLESTLHLWTRNNHSSNRITKTKKTRYNCLLR<br>MPKGTNNIMQKPKARNVKTPRFCCMEQHVSQMYSPFPQSMVKLWWSGNNGLQTLAKKFQQFLGR<br>HGQDIPTRLIFRSHQQQCRLPPQKLPLGYVASTSSKYSTSTIRANSPRHNEYTGISSPNRDCSK<br>HALLSPGKKGKSSTTSGAPKHIAEVYDLINCGPRNRFVIATPDGPLIVHN
AP-APSE2 dpol	DNA polymerase A (Pol I or family A)	Bacteriophage APSE-2, isolate=T5A	CLAKGTLVLTITGWMPIEIVSQDAYVWDGIEWVRTDGSVFNGNQEVIQAYGVGMTADHQVLTEK<br>GWKSASQSKRYNRSSCRLPDGYELPRFRRKEINLESTLHLWTRNNHSSNRITKTKKTRYSCLLR<br>MPKGTNNIMQKPKARNVKTPRFCCMEQHVSQMYSPFPQSMVKLWWSGNNGLQTLAKKFQQFLGR<br>HGQDIPTRLIFRSHQQQCRLPPQKLPLGYVASTSSKYSTSTIRANSPRHNEYTGISSPNRDCSK<br>HALLSPGKKGKSSTTSGAPKHIAEVYDLINCGPRNRFVIATPDGPLIVHN
AP-APSE4 dpol	DNA polymerase A (Pol I or family A)	Bacteriophage of *Candidatus Hamiltonella* defensa strain 5ATac, endosymbiot of *Acyrthosiphon pisum*	CLAKGTLVLTITGWMPIEIVSQDAYVWDGIEWVRTDGSVFNGNQEVIQAYGVGMTADHQVLTEK<br>GWKSASQSKRYNRSSCRLPDGYELPRFRRKEINLESTLHLWTRNNHSSHRITKTKKTRYSCLLR<br>MPKGTNNIMQKPKARNVKTPRFCCMEQHVSQMYSPFPQSMVKLWWSGNNGLQTLAKKFQQFLGR<br>HGQDIPTRLIFRSHQQQCRLPPQKLPLGYVASTSSKYPTSTIRANSPRHNEYTGISSPNRDCSK<br>HALLSPGKKGKSSTTSGAPKHIAEVYDLINCGPRNRFVIATPDGPLIVHN
AP-APSE5 dpol	DNA polymerase A (Pol I or family A)	Bacteriophage APSE-5	CLAKGTLVLTITGWMPIEIVSQDAYVWDGIEWVRTDGSVFNGNQEVIQAYGVGMTADHQVLTEK<br>GWKSASQSKRYNRSSCRLPDGYELPRFRRKEINLESTLHLWTRNNHSSHRITKTKKTRYSCLLR<br>MPKGTNNIMQKPKARNVKTPRFCCMEQHVSQMYSPFPQSMVKLWWSGNNGLQTLAKKFQQFLGR<br>HGQDIPTRLIFRSHQQQCRLPPQKLPLGYVASTSSKYPTSTIRANSPRHNEYTGISSPNRDCSK<br>HALLSPGKKGKSSTTSGAPKHIAEVYDLINCGPRNRFVIATPDGPLIVHN
AP-Aaphi23 MupF	Putative minor head protein,P32,traN-related protein	Bacteriophage Aaphi23, *Haemophilus* phage Aaphi23	CFPGQSELKGLPRPEKLYRRWYSGKLTELVTDNGTVLLATPNHPILTSNGIKSIDSVNVGDYLA<br>CEIKQTFDTVKLNGKNLIPTIEQVFNSLLLNGVRTSISSSKSGKFHGDFSDSEIEIISIDSFLI<br>DVLNALFIKKLPELGFTNADMVICKALFSTDSHFDLLKCASGSTGSSFMSRFNLLCSLLVAHLT<br>PLELFCLGLGANIGIIGKQIPANNISRDVEMFSNHIFACAALIHGKDFINWQRDRIMSLVAPNF<br>GHRYTDSFETLSKRLLVTTNNSANFGNAQSLGIEFRRVVNKVVTQASCHIYNLQTVSGYYNINS<br>VFVSN
AgP-S1249 MupF	Putative minor head protein	*Aggregatibacter* phage S1249	CFPGQSELKGLPRPEKLYRRWYSGKLTELVTDNGTVLLATPNHPILTSNGIKSIDSVNVGDYLA<br>CEIKQTFDTVKLNGKNLIPTIEQVFNSLLLNGVRTSISSSKSGKFHGDFSDSEIEIISIDSFLI<br>DVLNALFIKKLPELGFTNADMVICKALFSTDSHFDLLKCASGSTGSSFMSRFNLLCSLLVAHLT<br>PLELFCLGLGANIGIIGKQIPANNISRDVEMFSNHIFACAALIHGKDFINWQRDRIMSLVAPNF<br>GHRYTDSFETLSKRLLVTTNNSANFGNAQSLGIEFRRVVNKVVTQASCHIYNLQTVSGYYNINS<br>VFVSN
Ama MADE823	Hypothetical, Phage-related terminase	Phage uncharacterized protein [*Alteromonas macleodii* 'Deep ecotype']	NIAHSTPVLTANRGWVTHGDLVPGDQVFHPSGKPVDVLALSDEAVDDYVVTFTNGEKIRCHANH<br>EWTVYSRADKQEKTVETKWFLENTNRGTPRSLTAGNRFQFQVPKTNALEFDSADLPMHPYVLGA<br>WLGDGTKNSGCITHDKKDQPVIDKITRCGYEVSSWTVHKQTGVYTTRFSGPRPNVAGRMFKELK<br>ELNVLNNKHIPEVFLRASLKDRLELLAGLIDTDGHTDKNSRMRFTTADKPLANGVLDLCTTLGF<br>KPYIEEIQPKLSTSCIQGTKPYFVVGFNPTMVIPVALSRKRITRFPTERRVSIEKVEYLPNGEK<br>GHCIQVDSPDGLYLVGKKLVATHN
Bse-MLS10 TerA	Phage terminase-like protein, large subunit-like protein	*Bacillus selenitireducens* MLS10 (Probably prophage gene)	QLALDTPIPTPDGWSTMVELQVGDQVIDEKGNPCNVVARSEIDDTEQAYRITFRDGQSIVAGER<br>HLWQVQVMNNGQREKLLTTGELYKKQQKVKTKNRAIFRIPVVDAFSLPERSLPVDPYLFGYWIG<br>NGCATKPEITVRRDDVEAVQREVPYPLHNHYPQEGNSDILVYHALKPVLVSHFKDKRIPAAFTR<br>ASKAQRLRLLQGLMDSDGCVSTEKGQSIYVTILPELAKDVQDVLGSLGIKNTLGETPSTRNGVP<br>TGETCYLVRFTAFQDLNVSALDRKLNRSRERNPTTRSHFHYIAAIEKAEPTPMRCIQVDSPSRL<br>YLAGRSMVPTHN
BsuP-M1918 RIR1	Ribonucleoside-diphosphate reductase, alpha subunit	*B. subtilis* M1918 (prophage)	CVTGETLLLTENGYEKAADLYKKQNNLKVVIDNRTKDFAVDSKGTTIVDAIPMQLTKKDAEIFK<br>VKTKQGYEIRATEWHKFYVKRDGEIQKLQLNQLKTGDKLLVQSAEGAYGKIHEPDLAYIMGIIA<br>GDGTITEKTAKIYLYDNKKVLEQKVTDAVHRIIHKHKVDRAYKHNTSLLPTFNMANPEKQDLLY<br>MNSTVLFDILKKFGMNKERKTRVPEFIFQANKETQAAYLSGLFQTDGCVNANHKAKALTIELTS<br>IHYESLQDVQKLLLNMGVYTTIYSNNKRSQELLPDGKGGSKLYNVKPTHKISIQDRNSRELFMS<br>IVELKDYDVYKFNLLTETLQPKSRKPKHDFTAEIISIEEDGVEDVYDTTQEDYHSLIFNGIVTG<br>N
BsuP-SPBc2 RIR1	Ribonucleoside-diphosphate reductase, alpha subunit	*B. subtilis* strain 168 Sp beta c2 prophage	CVTGETLLLTENGYEKAADLYKKQNDLKVVIDNRTKDFAVGSKGTTIVDAIPMQLTKKDAEIFK<br>VKTKQGYEIRATEWHKFYVKRDGEIQKLQLNQLKTGDKLLVQSAEGAYGKIHEPDLAYIMGIIA<br>GDGTITEKTAKIYLYDNKKVLEQKVTDAVHRIIQKHKVDRAYKHNTSLLPTFNMANPEKQDLLY<br>MNSTVLFDILKKFGMNKETKTRVPEFIFQANKETQAAYLSGLFQTDGCVNANHKAKALTIELTS<br>IHYESLQDVQKLLLNMGVYTTIYSNNKRSQELLPDGKGGSKLYNVKPTHKISIQDRNSRELFMS<br>IVEMKEYDVYKFNLLTETLQPKSRKPKHDFTAEIISIEEDGVEDVYDTTQEDYHSLIFNGIVTG<br>N
CbP-C-St RNR	Ribonucleotide reductase, Anaerobic, RNR, class III	*Clostridium botulinum* phage C-St	CFDGQQKTLTKSSHGVNYLSFKDLYDTKYKDKERRNFKVFHNGNWVEGKPIRLLRNDKKMYKIT<br>TVNNKEILVTEDHINVTDKGDKYTTQLTENDYIAFNTRPTNAIPEKDEKLTYEQGVLIGAYLGD<br>GSKQRNEHKIQLSINEEKYNILRPLIEKALKQWNIQAQYRLYTPHNNFYPTAITSEDLLNIIEH<br>WVKGDYSYNKRLNLEILHQSIEFRKGVLDGIYFTDGGNSNRIYTTSDSLKEDLEILIHSLGMVS<br>IINTTDRTDEKVIIRGEEFNRNQKGLYKVINNTMFFKIKTIQQYISNDEYVYCFEMKNIEEPYF<br>TLPNGIITHN
CbP-D1873 RNR	Ribonucleotide reductase, Anaerobic,large subunit, RNR, class III	*Clostridium botulinum* phage D	CFDGQQKTLTKSSHGVNYLSFKDLYDTKYKDKERRNFKVFHNGNWVEGKPIRLLRNDKKMYKIT<br>TVNNKELLVTEDHINVTDKGDKYTTQLTENDYIAFNTRPTNAIPEKDEKLTYEQGVLIGAYLGD<br>GSKQRNEHKIQLSINEEKYNILRPLIEKALKQWNIQAQYKLYTPHNNAYPTAIISEDLLNTIEH<br>WVKGDYSYNKRLNLEILHQSIEFRKGVLDGIYFTDGGNSNRIYTTSDSLKEDLEILINSLGMVS<br>IINTTDGTDEKMIIRSEEFNRNQKGLYKVINNTMFFKIKTIQQYISNDEYVYCFEMKNIEEPYF<br>TLPNGVITHN
Ckl PTerm	Phage terminase, large subunit	*Clostridium kluyveri* DSM 555	ALDLDTPIDTPTGWKTMRDIECGDYVFGVDGKPTKVIGTSDIMVNHECYKVTFEDGEFIIADTE<br>HIWTVTTKSSRKTLKYKPLKGRQLLRPDYRESNGYFDVTTGEMAKNFKHFRKDGKGIEYKYRVP<br>MAGAVEYEEKDLILDPYILGVWIGNGASASARITASVNDTEIIEHVQKIGVSVNIYERKPNVLT<br>VTLREPLLDKYCHRGHLKIKVYDSRGKCKACAREIDYAHRHNLPRPEYKILTIQEKLRKLGVLN<br>NKHIPEIYLRASLEQRYELLKGLMDTDGYCSKAGQCEFVQKNKIIIDGFSNLLSSLGIKHSIRK<br>KQAKCNEKKSTVHSVLFYVDKEHSCFKLKRKHKRLKDKLSDRMKNKSVINIEKYKSVPVKCIAV<br>EDEKKLYLAGKNHTATHN

Cth-ATCC27405 TerA	Phage terminase-like protein, large subunit	*Clostridium thermocellum* ATCC27405 (Probable prophage)	QLALDTPIPTPDGWTTMGEIKAGDKVIDEKGRPCNVVAISEIDDTEQAYKINFRDGTSIVAGER<br>HLWKVQVTNNGRREKLLTTGEMYQKQFKTKSKENRALFRIPIADAFILPENKLPIDPYLFGYWI<br>GNGNAVKPEITVMRDDVDEVIKNIPYKLHNRYKQEGNSDILVYKELKSILVKNFREKRIPIEYL<br>RASAQQRKRLLQGLIDSDGCVSTAKSQAIYVTILFELAKDVQDLLWSLGIKNTLKTAPSARYGI<br>ETGEICYLIKFTAFNDLEVSGLDRKLKRGRERNIKTRSHFHYIKSIEKTGKTKMRCIQVDSPSR<br>LYLAGKSMIPTHN
Cth-DSM2360 TerA	Terminase	*Clostridium thermocellum* DSM 2360 (Probably prophage gene)	QLALDTPIPTPDGWKRMGELKQGDVVFDENGSPCHVLALSEIDDTEQAYRLTFGDGSSIIAGAR<br>HLWKVQIINNGRKERLLQTQQMYEAFSAYRKRHKDAPFRSIYRIPVAGALKLPDAKLPVDPYLY<br>GYWLGNGCATRPEITIRTCDVAGVLKRIPYEVSSLWKNVGDSVVVRIPVLKSVLLKSHHSKHIP<br>SEYLRASENQRWELLQGLMDSDGCIGKLKAQSIYVSTEKQLALDVRELLWSLGIKNSMTESPSQ<br>RCGKPTGKTLYTIRFTSFADLPTSGLARKLCRRKETGSSPTRSNYHYIHSIEPVKERIPMRCIQ<br>VSSPSRQYLAGTSMVPTHN
EP-Min27 Primase	DNA primase, Helicase	Enterobacteria phage Min27	ACPLNEPILLADGTWTTHGDVKIGDQVASVDGNPSTVTGIFPQGVRDVYRVTFEDGRYVDCAGD<br>HLWEVTSRGFTKGEKRRVIDTFELKRLSETKRHKNGVRIPEITGDFGDHSEPLAWVIGSLLGDG<br>SLSNGSVKFSNVEPYMIERMKAELPDYNFSGDGKDWLISTARGQANPLMETLRGYGLMGCTAKN<br>KFIPRVFFSANKSTRIGMLCGLLETDGYVEKDGTLVFSSASEELRNGVVQLVNSLGGSCRTRVK<br>TGVTYTYKDDKRHGMDSYEARIRLTREIREAIRSPRLNGRLTAHRFEGCGVFVRNVEKIGNAEC<br>LCIMVDHPRHLYVTRGYVATHN
LLP-KSY1 PolA	DNA polymerase family A, gp96	*Lactococcus* phage KSY1	CFSGDTEILTPYGWVKFEDYDESMMCAQYDEVTGKISFTYPNETIHLKDQDIWVYEDTNTSIHA<br>TGNHDILIQKPNGDIAKEKFSNLQLLQKGDKHRFINAGYVDSAEEVDTLMQRLVAFTTRYGQTR<br>RDKLHFAIKKPRYVSYLIELLDDLEVIYHLQREYHSGQDFYYSYIEVDDSFLLDHIREFLNDDN<br>SLKYESIPKLGMPFLEAFKDFGHLTSKNGIVVKDIHRSTLDVVQQVGIRHGVKVRINGSYTAAY<br>NLAKGVFSFPGKAFPTGPSYKGDVYCVNVPTHNIVIRHNDKVSIQGN
LP-phiHSIC Helicase	Helicase	*Listonella pelagia* phage phiHSIC	CHAYGHDIMMSDGTKKQVQDIAVGDKVMGPDGNPRKVIRLVKGQDEMFRVTPTKGESFVVNGGH<br>ILSLYQTPRRAGQTPGYTEISVNEYIRSSSTFKHRSKLQRFGFDLPGKNLPIPPYILGLLLGDG<br>CITGVGQMSFTSQDHAAITEIERYLMSIGASNRLHFDSREAVGVCFRDYHRLEDHLTELGLYGR<br>NSGDKFIPSEYLTSSNEDALEMIAGLLDTDGSLCPHGVYDYISKSRELSEGLVYLCRRVGLAAY<br>ISECEKSCQNGFKGTYYRVCVSGDLSKVPCRVSHKKCIFDRKQKKRVDVTGFKVEPIGVGDYYG<br>FTVDGDHLYLDGDFVRHHN
MP-Be DnaB	DnaB helicase, gp10	Mycobacteriophage Bethlehem	PLALNTEVPTPSGWTTVGDLSVGDYVLGSDGQPHRVQRETPVLEGLATYVVRFDDGTEITASAS<br>HGWTTQRLTGHGDSYETVTVTTEELAQTVTNSKGRKRHRIPVVGMELPNQELPLDPWFLGLWLG<br>DGATSDSTVSFDYRLRDEYATLLKPLVQEFQTVVWDNPVPGTNVGTFRIKNNDRTQDDKSIRSL<br>LRKAGVLGNKHIPAAYMQAGTDQRFELLRGLIDSDGGIDSVGRAYFVNANRNLVYQFQELVVGL<br>GFRCTVREHGGDGALRAEFNPGNAVRVSNLAYKFERQRPYSSRNRSQHRWVESVTPVESVPVKC<br>IGIDTEDHLFQVSRSRILTHN
MP-Be gp51	gp51	Mycobacteriophage Bethlehem	CSWTNARAVTRRGFVHVDDLTTDDEVMSVDDQGRTIWQQIDEVVRFPFSGTLYSLGGREINATI<br>TANHRVVGLNREKTKWVEHTPTSLPGNKMWVYTAGEGSNEDYPLTDTEIRLAVWGLTDSHRSPD<br>GRWTFYQSGEKAEQVRKLLADAGIEYRERARNRGITEIDGKVLKAPPKTQYEFSLGKVQELDDL<br>LDRGRSELPTWTLSLSQRQARLFLEEYRFTDGTDTTSAGDSYVLYVCKDRMREQLQMLAAANGL<br>RASTTEYRPGHWRLNISNRALSGLYKNTVEEVAYEGEVWCLRVPNGRFFIEDGGKIHLTGN
MP-KBG gp53	gp53	Mycobacteriophage phage KBG	CSWTNARAVTRRGFVHVDDLTTDDEVMSVDDQGRTIWQQIDEVVRFPFSGTLYSLGGREINATI<br>TANHRVVGLNREKTKWVEHTPTSLPGNKMWVYTAGEGSNEDYPLTDTEIRLAVWGLTDSHRSPD<br>GRWTFYQSGEKAEQVRKLLADAGIEYRERARNRGITEIDGKVLKAPPKTQYEFSLGKVQELDDL<br>LDRGRSELPTWTLSLSQRQARLFLEEYRFTDGTDTTSAGDSYVLYVCKDRMREQLQMLAAANGL<br>RASTTEYRPGHWRLNISNRALSGLYKNTVEEVAYEGEVWCLRVPNGRFFIEDGGKIHLTGN
MP-Catera gp206	gp206	Mycobacteriophage Catera	SLACSTPILTTNGWSTMGALQDGDEVYAPDGQPTKVIKAHPINLNRPCFKVRFRDGQEVVTDAE<br>HLWQVNDRNNGGRDRVMTTQEIADAPWGGRYRFRVPVTEPLQTPEADLPVDPWLLGAWLGDGDA<br>SMVSICSGSQDLDYLISRVEGLGLSHRVNRYGSRAASVYVHGMRAVFSELGLLKNKHIPDRYLT<br>ASVAQRRQLLAGLMDSDGTVSDRQVTISMKNERLMRQVLQLVRSLGYRAGFGSRMARLNGRDCG<br>LVYVVRFHTGWGESPFDMPRKRDGWEQATKTSVQNLRLNAIVAVEPVETVPVRCITVAHESSLY<br>VAGEGFVPTHN
MP-Mcjw1 DnaB	DnaB helicase	Mycobacteriophage CJW1	ALDVETPILTGNGWKKMGDIQVGDYVHAADGTLARVSYVSERHWRDCFSVQFADGAELVASDHH<br>LWAVNDRLKGERVIDTAELYRTQTYGARGDRRYTVTVPEALDRDEAPLPLDPYILGAWLGDGTA<br>TRAEFTSEDPEIFAAIEAAGYPLSYDYASGNARTRGAKGLVAVLRKMGVLGAKHVPQDYLIGSR<br>AQRLALLQGLMDTDGSVITGPNTPRVEFCNTNRDLAEAALFLARSLGWKATLKESRARLNGKDC<br>GPRFRVSWTAYSDMSPFRLQRKSEKLAAAPARATRARTNTITSVTPVPTVETVCIQIDHPSHVF<br>LAGKSLTPTHN
MP-Omega DnaB	DnaB helicase	Mycobacteriophage Omega	ALAIDTPILSERGWTTMGDLVDGDRVYGPDGQLTNVIAHPIRYERPCYRLTFDDGQTIVADEDH<br>LWTVYDAVKREHRTLTVRELVDGGVFTTRRNAGRADSNIYRYRVPVTEPLAGVEADLPVDPYLL<br>GYWLGDGDTNAGRFTVGEEDLEAFKLTLESLGYEYSDSVDPRTGAHTICAYGFIQGLREAGVVG<br>NKHIPETYLTASMEQRRALLAGIMDSDGGVTGHQISVTMKNEALMRQVLMLARSLGYKSFFTSH<br>LSMLNGEHKARVYRVKFANRQELNPFRLPRKAAKVLPPLGRVTRAQYNAIVSIEPVESVPTRCI<br>TVDNDSRLYVVGHGFVPTHN
MP-U2 gp50	gp50	Mycobacteriophage U2	CSWTNARAVTRRGFVHVDDLTTDDEVMSVDDQGRTIWQQIDEVVRFPFSGTLYSLGGREINATI<br>TANHRVVGLNREKTKWVEHTPTSLPGNKMWVYTAGEGSNEDCPLTDTEIRLAVWGLTDSHRSPD<br>GRWTFYQSGEKAEQVRKLLADAGIEYRERARNRGITEIDGKVLKAPPKTQYEFSLGKVQELDDL<br>LDRGRSELPTWTLSLSQRQARLFLEEYRFTDGTDTTSAGDSYVLYVCKDRMREQLQMLAAANGL<br>RASTTEYRPGHWRLNISNRALSGLYKNTVEEVAYEGEVWCLRVPNGRFFIEDGGKIHLTGN
Mca MupF	Prophage MuMc02, F protein	*Methylococcus capsulatus* Bath, prophage MuMc02	CFPAETPVRAAARLGLKTWYAGKVVELQTRLGHRLTLTANHPVLTVRGWIAACQLQKGDQLIGD<br>ASGVNPRLAGVVNDEQPPARAEDLFQTLAAQGFRIVPMAPHDFHGDAGLRKPEIHIAGPDVHLM<br>DEVQAAPGQFVGQQQLRRADACAIMDADRPDGPPPARMILADAVAPQNPADVAEAGAELAADGA<br>FGDQPVAVQGQHPAFEMGVAVAGALPGGGALASNGGGVLFDGSPFDALGFRAPPQGDVAGTEQP<br>AQGVTAASGLVRQLLEANAGLIALDEIVQIRQFDWAGHVYDFETETGLIMAGGVIVHN
PP-PhiEL Helicase	Helicase, PhiEL_ORF166	*Pseudomonas aeruginosa* phage phiEL	AMPLSTRVKVPNGWKALGDLKVKDIVVTPGGDTACVESIYPQGITEVYRFYFEDGRTADSHPYH<br>LWKTTVNGVDEILTTLEVLHKARKEDVYFPLVGEINGCNPHCDTSSEVAARELVNTDVVIGDNV<br>LELPYRDRFNIVTSVIEHVGCLISESVLSAYHENRIGMENFRRLMWSIGGTATEPVLVNGLYKV<br>DFKHRDVGKMMTGLIGDNPRIQGMYNFSQYEDLQLKLAYWEKVENQETCCIALDNDEKLYVVDD<br>YIVTHN

PP-PhiEL ORF11	PhiEL_ORF11, putative ATP-dependent DNA helicase	*Pseudomonas aeruginosa* phage phiEL	QQRNSAKVRIVPKDTLKTITPQDTWKRIEHLRVGDQVLDRSGKPCQVIGIHPQGKRRLYRVITS<br>DGRATDVGTEHLWTLKDYSNCLNGRALWNDYSTVDVINLLKKKVKLQLPLPAPVPGSEQDLPID<br>PYVLGLIYCGQDQDGKVIIPTRTDAVKQYVVDHLPRGVTVIQGVANSCLERTDNQPYLFNREHG<br>LPDQYLEAPLNARRDLLQAFLDVRGKVGRGKVFIALNRVLGGQLAYLARSLGGTGKVTKNGVEI<br>TLPEEVPPFKFREENVVFDNRLLIERVTFVGDDDCTCIEVDNSEQLYLTDDFIVTHN
PP-PhiEL ORF39	PhiEL_ORF39	*Pseudomonas aeruginosa* phage phiEL	AGVLSSKIKIPGGWKTMGNIRVGDEVVTPDGGTAKVLAVHPQGVTKVVRVHFKDGRYTDVSPDH<br>LWKVRRHHWCNDKAMAKLTREEVEERVWRVITTNELKDYIGLSTKVYVQLIEPERNADKPFKIH<br>PYVLGVLLGDGCISQKAVDITKPYQQLFDKVQSLLPEHLECVWRPNRKGDGEPKTFGIRFKDRR<br>SEQHINWHIRDGLKELGLYGMRSWGKVIPEEYLHGSAKQRLELLQGLLDTDGTVDKHKSVSFSS<br>SSKLLSLGVQYLVRSLGGMARLQERTPHYTHNGEKREGRTDYRVYIRYPRPEELFTLDHKRERA<br>VSHQHTETLRLQVTHIEERPDEETQCITIDHPDHLYITDDFIVTHN
PP-PhiEL ORF40	PhiEL_ORF40	*Pseudomonas aeruginosa* phage phiEL	MQPLYAKIKVPGGWKTMREMTVGTEVIAADGTVTQVTGVYPHGKQPIYRLHFEDGRYTDAGLDH<br>LWKVFTEETQAWTVVNTRSVQTLLAKEPDGVFIPLCEPEDGPEKPFVTDQLEGSRQQRLEHLRR<br>LMDEKGYVRDDGSLSFSSEDEVESTTVQYLVRSLGGIARKVPSTGLYRRRQYRVYIKHPRPEEL<br>FTLTNKGGYLTSKSGNQSLKLRVNRIEFIGEHEAQCISVAHPDRLYITDDFIVTHN
SaP-SETP5 dpol	DNA polymerase A (Pol I or family A)	*Salmonella* phage SETP5	CLHRHTQVLTDGGFKDIMAVTSTDKVWSGEKWVNTKGAHLMGWKPVINVDGVLMTEDHKILTHS<br>WKQAKQLVSNKYMMDRALEIGMDAWLSCASYQNDKAKDNYSSNVIVERCLGGSIMTMSGRVKPL<br>NATAAPLKQQKNIVNSISATKTQCRMMRTERDYSTGCPRRSREQQAPGIRVIKTMGQEASRYST<br>SGGLIKGLFLDMFKLWKAGMTRTSKWIESTQTVTTNLETFGLSAVLKTAGTDGKYLSFNESMMQ<br>PLPALLNLNGKLTYCEPVYDLIDVEDGNRFLIASDSGFLVAHN
SaP-SETP12 dpol	DNA polymerase A (Pol I or family A)	*Salmonella* phage SETP12	CLHRHTQVLTDGGFKDIMAVTSTDKVWSGEKWVNTKGAHLMGWKPVINVDGVLMTEDHKILTHS<br>WKQAKQLVSNKYMMDRALEIGMDAWLSCASYQNDKAKDNYSSNVIVERCLGGSIMTMSGRVKPL<br>NATAAPLKQQKNIVNSISATKTQCRMMRTERDYSTGCPRRSREQQAPGIRVIKTMGQEASRYST<br>SGGLIKGLFLDMFKLWKAGMTRTSKWIESTQTVTTNLETFGLSAVLKTAGTDGKYLSFNESMMQ<br>PLPALLNLNGKLTYCEPVYDLIDVEDGNRFLIASDSGFLVAHN
SaP-SETP3 dpol	DNA polymerase A (Pol I or family A)	*Salmonella* phage SETP3	CLHRHTQVLTDGGFKDIMAVTSTDKVWSGEKWVNTKGAHLMGWKPVINVDGVLMTEDHKILTHS<br>WKQAKQLVSNKYMMDRALEIGMDAWLSCASYQNDKAKDNYSSNVIVERCLGGSIMTMSGRVKPL<br>NATAAPLKQQKNIVNSISATKTQCRMMRTERDYSTGCPRRSREQQAPGIRVIKTMGQEASRYST<br>SGGLIKGLFLDMFKLWKAGMTRTSKWIESTQTVTTNLETFGLSAVLKTAGTDGKYLSFNESMMQ<br>PLPALLNLNGKLTYCEPVYDLIDVEDGNRFLIASDSGFLVAHN
SaP-SETP3 Helicase	ORF4, DEAD-like helicases superfamily	*Salmonella* phage SETP3	CLKRGTEVIMFDGTTKKVEDVIVGDVLMGPDSTPRNVLSLGRGREMMYEVKPRKGESYTVNESH<br>ILSLRTTTGIAKGSWPDNTVFDISVRDWLKLPKYVTGPNGYLKGWRVPVDFPRKEQDEALLPPY<br>LMGLWLGDGTSSSGAITSGENEKEIRAYLESYAARNGMQIRKEGLTWSISHGNTGHKKHGFTHA<br>LKSAGVLNNKHIPHNYKCGDRRQRLELLAGLLDSDGYCDLSKAGFDWISVSERLADDFCYLCRS<br>LGFAAYKKKTCKRCANTDVWGDYFRVSVSGDFSEVPFVRGRHQNLPKRNINKNVLNVGIESITP<br>VGVDDYFGFTIDGDSRFLLGDFTVTHN
ShP-Sfv-5 Primase	DNA primase, Helicase	*Shigella flexneri* 5 str. 8401	ACPLNEPILLADGTWTTHGNVKIGDQVASVDGNPSTVTGIFPQGVRDVYRVTFEDGRYVDCAGD<br>HLWEVTSRGFTKGEKRRVIDTFGLKRLSETKRHKNGVRIPEITGDFGDHSEPLAWVIGSLLGDG<br>SLSNGSVKFSNVEPYMIERMKAELPDYNFSGDGKDWLISTARGQVNPLMETLRGYGLMGCTAKN<br>KFIPRVFFSANKSTRIGMLCGLLETDGYVEKDGTLVFSSASEELRNGVVQLVNSLGGSCRTRVK<br>TGVTYTYKDDKQHGMDSYEARIRLTREIREAIRSPRLNGRLTAHRFEGCGVFVRNVEKIGNAEC<br>LCIMVDHPRHLYVTRGYVATHN
SoP-SO1 dpol	DNA polymerase A (Pol I or family A)	*Sodalis* phage SO-1	CLHRHTQVLTNGGFKDIMAVTSTDKVWNGEKWVSTQGAHLMGWKPVIDVDGVLMTEDHKILTHS<br>WKEARQLVSDRCTMARALARGMDAWLSCANYQNDRGTDNCWPNVIAERCQDASGMTTSEGVKHP<br>NATSAQLKRQSGIVNSIFATMTQCQTMRTERGCSTGCQRQYHERQTPAPKGIKTTAVVGLPYVT<br>NGREIKGRFFSTFKRWTAGIIPTSKWTGSIQTATMSLETFGLSAVRKTAVTGAACPSFSESTMK<br>PLPALLNWNGKLTYCEPVYDLIDVEDGNRFLIASKSGFLVAHN
StP-Twort ORF6	TwortORF006	*Staphylococcus* phage Twort	CISMDSMILTTEGYKSLQEIFETQGVKVDNKEKVIELKYPLINRYGDVEYTSHFTKNGEKPTKR<br>IKTNKGIELVNTYNHPLLVREGFNLIWKKSEDIEVGDILVSRVGDHQFGNNNTVENEEEAYALG<br>CMVADSYLGSYSRLSFSNDKKEILDKVSKFWNTFSNKEVYYDTYKESKGITIHLHDTNKTKEFH<br>DKYKIEYGVAKDKKIPKCIMESPENIQLAFVSGYLECESSISEKNLEVTSASKDLLKDLQLILS<br>NIGIVSTNKEKVVKKYKHNKYYRLIVNRKELIKLLPLLRFETQQRKNQKENFLSNNTKIKSSYG<br>NTIEGSRYLLKKYRDSLNIDKKEFSKYLSRDTITIDRLREVISLYPDGDKEIRELFENVVNNNI<br>YYQKVEQVLEGEIIPTFDVCMPKTHSFIANTIVNHN

Supplementary Table 2 | Characteristics of the inteins analyzed in this study

Intein	Name used[a]	Organism	Extein	Junction sequence[b]	Intein type	Evidence[c]	Source
*Ssp*DnaB$^{\Delta 275}$ M86	M86	*Synechocystis* sp. PCC 6803	Replicative DNA helicase (DnaB)	VDA/SDL	Artificially mini	E	[1]
Npu^{N}/*Ssp*^{C}DnaE	NpuSsp	*Nostoc punctiforme* PCC73102 and *Synechocystis* sp. PCC 6803	DNA polymerase III alpha subunit DnaE	GGK/CWN	Chimeric, Naturally split	E	[2,3]
gp41-1	gp41-1	GOS metagenomic dataset	gp41 DNA helicase	SGY/SSS	Naturally split	E	[4-6]
gp41-8	gp41-8	GOS metagenomic dataset	gp41 DNA helicase	LNR/SAV	Naturally split	E	[4-6]
NrdJ-1	NrdJ-1	GOS metagenomic dataset	ribonucleotide reductase (RNR) class II	NPC/SEI	Naturally split	E	[4-6]
IMPDH-1	IMPDH-1	GOS metagenomic dataset	Inosine-5′-monophosphate dehydrogenase (IMPDH)	GGG/SIC	Naturally split	E	[4-6]
*Ssp*DnaX$^{\Delta 297}$	SspDnaX	*Synechocystis* sp. PCC 6803	DNA polymerase III alpha subunit DnaX	IDE/CHT	Artificially mini	E	[7]
*Ssp*GyrB$^{\Delta 279}$	SspGyrB	*Synechocystis* sp. PCC 6803	DNA gyrase subunit B (GyrB)	AGG/SAK	Artificially mini	E	[7]
*Ter*ThyX$^{\Delta 134}$	TerThyX	*Trichodesmium erythraeum* IMS101	Thymidylate synthase X	IGC/SFD	Artificially mini	E	[7]
*Tvo*VMA	TvoVMA	*Thermoplasma volcanium* GSS1	Vacuolar ATPase (H+-transporting ATP synthase), subunit A	GSK/TVI	Naturally mini	E	[8]
*Pho*RadA	PhoRadA	*Pyrococcus horikoshii*	RadA DNA repair protein	SGK/TQL	Naturally mini	E	[8]
CroV RIR1	Cro-RIR1	*Cafeteria roenbergensis* virus BV-PW1	Ribonucleoside-diphosphate reductase, alpha subunit	VNQ/SGR	Bifunctional	T	[9]
CroV RPB2	Cro-RPB2	*Cafeteria roenbergensis* virus BV-PW1	DNA-directed RNA polymerase beta (II) subunit 2	DKF/STK	Naturally mini	T	[9]
CroV Top2	Cro-Top2	*Cafeteria roenbergensis* virus BV-PW1	DNA Topoisomerase IIA	GSK/CSN	Bifunctional	T	[9]
CIV RIR1	CIV-RIR1	*Chilo iridescent* virus	Ribonucleoside-diphosphate reductase, alpha subunit	SNL/CSE	Bifunctional	E	[9,10]
CP-P1201 Thy1	CP-Thy1	*Corynebacterium* phage P1201	Putative thymidylate synthase	KHH/SVL	Bifunctional	T	[9]
Ama MADE823	Ama-Ter	*Alteromonas macleodii* 'Deep ecotype'	Hypothetical, Phage-related terminase	HGK/SEL	Bifunctional	T	[9]
BsuP-M1918 RIR1	BsuP-RIR1	*Bacillus subtilis* M1918 (prophage)	Ribonucleoside-diphosphate reductase, alpha subunit	SNL/CSE	Bifunctional	T	[9]
CbP-C-St RNR	Cbp-RNR	*Clostridium botulinum* phage C-St	Ribonucleotide reductase, Anaerobic, RNR, class III	ASC/CRL	Bifunctional	T	[9]
Ckl PTerm	Ckl-Ter	*Clostridium kluyveri* DSM 555	Phage terminase, large subunit	NGK/TTL	Bifunctional	T	[9]

Cth-ATCC27405 TerA	Cth-Ter	*Clostridium thermocellum* ATCC27405	Phage terminase-like protein, large subunit	NGK/SEL	Bifunctional	E	[9,11]
EP-Min27 Primase	EP-Pri	Enterobacteria phage Min27	DNA primase, Helicase	HGK/TEV	Bifunctional	T	[9]
LLP-KSY1 PolA	LLP-Pol	*Lactococcus* phage KSY1	DNA polymerase family A, gp96	AKA/CNF	Naturally mini	T	[9]
LP-phiHSIC Helicase	LP-Hel	*Listonella pelagia* phage phiHSIC	Helicase	ATG/CGK	Naturally mini	T	[9]
MP-Be DnaB	MP-B-DnaB	Mycobacteriophage Bethlehem	DnaB helicase, gp10	QDQ/TKN	Bifunctional	E	[9,12]
MP-KBG gp53	MP-K-gp53	*Mycobacterium* phage KBG	gp53	NHD/SRA	Naturally mini	T	[9]
MP-Catera gp206	MP-C-gp206	Mycobacteriophage Catera	gp206	ELK/TQN	Bifunctional	E	[9,13]
MP-Mcjw1 DnaB	MP-M-DnaB	Mycobacteriophage CJW1	DnaB helicase	NGK/TEL	Bifunctional	T	[9]
PP-PhiEL ORF39	PP-Phi	*Pseudomonas aeruginosa* phage phiEL	PhiEL_ORF39	AGK/SLT	Bifunctional	T	[9]
SaP-SETP3 dpol	SaP-dpol	*Salmonella* phage SETP3	DNA polymerase A (Pol I or family A)	GKA/CEL	Naturally mini	T	[9]
SaP-SETP3 Helicase	SaP-Hel	*Salmonella* phage SETP3	ORF4, DEAD-like helicases superfamily	SGK/TSS	Bifunctional	T	[9]
NrdA-2	NrdA-2	GOS metagenomic dataset	ribonucleotide reductase (RNR) class I	SNL/CNE	Naturally split	T	[4,5]
*Pfu*RIR1-1	Pfu-RIR1	*Pyrococcus furiosus*	Ribonucleoside-diphosphate reductase, alpha subunit	GGG/TGL	Bifunctional, artificially split	E	[14,15]
*Mja*KlbA	Mja-KlbA	*Methanococcus jannaschii*	KlbA, kilB operon ORF A	HDG/CSG	Naturally mini	E	[16,17]

[a] As used in this study
[b] Extein's amino acid residues flanking the intein insertion site (-3, -2, -1 / +1, +2, +3)
[c] Experimental (E)/Theoretical (T)

Supplementary Table 3 | Mini-intein protein sequences and synthesized DNA sequences

Intein	Protein sequence[a]	Synthesized DNA sequence[b]
M86	CISGDSLISLASTGKRVPIKDLLGEKDFEIWAIN<br>EQTMKLESAKVSRVFCTGKKLVYTLKTRLGRTIK<br>ATANHRFLTIDGWKRLDELSLKEHIALPRKLESS<br>SLQLAPEIEKLPQSDIYWDPIVSITETGVEEVFD<br>LTVPGLRNFVANDIIVHN	TGCATCTCGGGAGATAGTTTGATCAGCTTGGCGAGCACAGGGAAAAGAGTTCCTATTAAGGATT<br>TGTTAGGCGAAAAAGATTTTGAAATATGGGCAATTAATGAACAGACGATGAAGCTGGAATCAGC<br>TAAAGTTAGTCGTGTATTTTGTACCGGCAAAAAGCTAGTCTATACTCTAAAAACTCGACTAGGT<br>AGAACTATCAAGGCAACAGCAAATCATAGATTTTTAACTATTGATGGTTGGAAAAGATTAGATG<br>AGCTATCTTTAAAAGAGCATATTGCTCTACCCCGTAAACTAGAAAGCTCCTCTTTACAATTGGC<br>ACCAGAAATAGAAAAGTTGCCTCAGAGTGATATTTACTGGGACCCCATCGTTTCTATTACGGAG<br>ACTGGAGTCGAAGAGGTTTTTGATTTGACTGTGCCAGGACTACGTAACTTTGTCGCCAATGACA<br>TCATTGTACATAAC
NpuSsp	CLSYETEILTVEYGSLPIGKIVEKRIECTVYSVD<br>NNGNIYTQPVAQWHDRGEQEVFEYCLEDGSLIRA<br>TKDHKFMTVDGQMLPIDEIFERELDLMRVDNLPN<br>MVKVIGRRSLGVQRIFDIGLRQDHNFLLANGAIA<br>AN	TGCCTGAGCTATGAAACCGAAATTCTGACCGTGGAATATGGCAGCCTGCCGATTGGCAAAATTG<br>TGGAAAAACGCATTGAATGCACCGTGTATAGCGTGGATAACAACGGCAACATTTATACCCAGCC<br>GGTGGCGCAGTGGCATGATCGCGGCGAACAGGAAGTGTTTGAATATTGCCTGGAAGATGGCAGC<br>CTGATTCGCGCGACCAAAGATCATAAATTTATGACCGTGGATGGCCAGATGCTGCCGATTGATG<br>AAATTTTTGAACGCGAACTGGATCTGATGCGCGTGGATAACCTGCCGAACATGGTGAAAGTGAT<br>TGGCCGCCGCAGCCTGGGCGTGCAGCGCATTTTTGATATTGGCCTGCGCCAGGATCATAACTTT<br>CTGCTGGCGAACGGCGCGATTGCGGCGAAC
gp41-1	CLDLKTQVQTPQGMKEISNIQVGDLVLSNTGYNE<br>VLNVFPKSKKKSYKITLEDGKEIICSEEHLFPTQ<br>TGEMNISGGLKEGMCLYVKEMMLKKILKIEELDE<br>RELIDIEVSGNHLFYANDILTHN	TGTCTGGATCTGAAAACCCAGGTTCAGACACCGCAGGGTATGAAAGAAATTTCAAATATTCAGG<br>TGGGTGATCTGGTTCTGAGCAATACCGGTTATAATGAAGTGCTGAATGTGTTTCCGAAAAGCAA<br>AAAAAAAGCTATAAAATCACCCTGGAAGATGGCAAAGAAATCATTTGTAGCGAAGAACACCTG<br>TTTCCGACCCAGACCGGTGAAATGAATATTAGCGGTGGTCTGAAAGAAGGTATGTGCCTGTATG<br>TTAAAGAAATGATGCTGAAAAAAATCCTGAAAATCGAGGAACTGGATGAACGCGAACTGATTGA<br>TATTGAAGTTAGCGGTAACCACCTGTTCTATGCCAATGATATTCTGACCCATAAT
gp41-8	CLSLDTMVVTNGKAIEIRDVKVGDWLESECGPVQ<br>VTEVLPIIKQPVFEIVLKSGKKIRVSANHKFPTK<br>DGLKTINSGLKVGDFLRSRAKMCEIFENEIDWDE<br>IASIEYVGVEETIDINVTNDRLFFANGILTHN	TGTCTGAGCCTGGATACCATGGTTGTTACCAATGGTAAAGCCATTGAAATTCGTGATGTGAAAG<br>TTGGTGATTGGCTGGAAAGCGAATGTGGTCCGGTTCAGGTTACCGAAGTTCTGCCGATTATCAA<br>ACAGCCGGTTTTTGAAATTGTGCTGAAAAGCGGCAAAAAAATCCGTGTTAGCGCCAATCATAAA<br>TTCCCGACCAAAGATGGTCTGAAAACCATTAATAGCGGTCTGAAAGTGGGCGATTTTCTGCGTA<br>GCCGTGCAAAAATGTGTGAAATCTTTGAAAACGAGATCGACTGGGATGAAATTGCCAGCATTGA<br>ATATGTTGGTGTGGAAGAAACCATCGATATTAACGTGACCAATGATCGTCTGTTTTTTGCCAAT<br>GGTATTCTGACCCATAAT
NrdJ-1	CLVGSSEIITRNYGKTTIKEVVEIFDNDKNIQVL<br>AFNTHTDNIEWAPIKAAQLTRPNAELVELEIDTL<br>HGVKTIRCTPDHPVYTKNRGYVRADELTDDDELV<br>VAIMEAKTYIGKLKSRKIVSNEDTYDIQTSTHNF<br>FANDILVHN	TGTCTGGTTGGTAGCAGCGAAATCATTACCCGTAATTATGGTAAAACCACCATCAAAGAAGTGG<br>TCGAGATCTTCGATAACGACAAAAACATTCAGGTGCTGGCCTTTAATACCCATACCGATAATAT<br>TGAATGGGCACCGATTAAAGCAGCACAGCTGACCCGTCCGAATGCAGAACTGGTTGAACTGGAA<br>ATTGATACCCTGCATGGTGTTAAAACCATTCGTTGTACACCGGATCATCCTGTGTATACCAAAA<br>ATCGTGGTTATGTTCGTGCAGATGAACTGACCGATGATGATGAACTGGTGGTTGCAATTATGGA<br>AGCCAAAACCTATATCGGCAAACTGAAAAGCCGTAAAATTGTGAGCAACGAGGATACCTATGAT<br>ATTCAGACCAGCACCCATAACTTTTTCGCCAATGATATTCTGGTGCATAAT
IMPDH-1	CFVPGTLVNTENGLKKIEEIKVGDKVFSHTGKLQ<br>EVVDTLIFDRDEEIISINGIDCTKNHEFYVIDKE<br>NANRVNEDNIHLFARWVHAEELDMKKHLLIELEM<br>KFKLKEITSIETKHYKGKVHDLTVNQDHSYNVRG<br>TVVHN	TGTTTTGTTCCGGGTACACTGGTGAATACCGAAAATGGTCTGAAAAAAATCGAAGAAATCAAAG<br>TGGGCGACAAAGTGTTTAGCCATACCGGTAAACTGCAAGAAGTTGTTGATACCCTGATCTTTGA<br>TCGTGATGAAGAGATTATTAGCATCAACGGTATCGACTGCACCAAAAACCATGAGTTTTATGTG<br>ATCGACAAAGAAAATGCCAATCGCGTGAACGAAGATAACATTCACCTGTTTGCACGTTGGGTTC<br>ATGCCGAAGAACTGGATATGAAAAAACATCTGCTGATCGAGCTGGAAATGAAATTCAAACTGAA<br>AGAGATCACCAGCATCGAAACCAAACACTATAAAGGCAAAGTTCATGATCTGACCGTGAATCAG<br>GATCATAGCTATAACGTTCGTGGCACCGTTGTTCATAAT
SspDnaX	CLTGDSQVLTRNGLMSIDNPQIKGREVLSYNETL<br>QQWEYKKVLRWLDRGEKQTLSIKTKNSTVRCTAN<br>HLIRTEQGWTRAENITPGMKILSPAPQWHTNFEE<br>VESVTKGQVEKVYDLEVEDNHNFVANGLLVHN	TGCTTAACGGGGGACTCACAAGTTTTGACCCGCAATGGCTTAATGTCCATTGACAATCCCCAAA<br>TAAAAGGGCGAGAAGTTCTGAGCTACAACGAAACTCTACAGCAATGGGAATATAAAAAAGTTTT<br>AAGATGGCTTGACAGAGGCGAAAAGCAAACATTGTCTATTAAGACAAAAAATTCTACAGTACGG<br>TGTACGGCTAACCATTTAATCAGAACTGAACAAGGATGGACGAGAGCGGAAAACATCACTCCCG<br>GAATGAAGATACTATCCCCTGCGCCGCAATGGCATACAAATTTCGAGGAAGTTGAGTCCGTCAC<br>TAAGGGTCAAGTGGAAAAAGTTTATGACCTGGAGGTGGAAGATAATCACAATTTTGTTGCCAAT<br>GGCTTACTAGTCCATAAC
SspGyrB	CFSGDTLVALTDGRSVSFEQLVEEEKQGKQNFCY<br>TIRHDGSIGVEKIINARKTKTNAKVIKVTLDNGE<br>SIICTPDHKFMLRDGSYKCAMDLTLDDSLMPLHR<br>KISTTEDSGHAMEAVLNYNHRIVNIEAVSETIDV<br>YDIEVPHTHNFALASGVFVHN	TGTTTTTCTGGAGATACATTAGTCGCTTTAACTGATGGTCGTAGCGTTAGCTTTGAGCAATTGG<br>TTGAAGAAGAAAAACAAGGAAAACAAAACTTTTGTTATACCATCCGCCATGATGGTTCTATAGG<br>GGTTGAAAAAATCATCAATGCCCGCAAAACAAAAACTAATGCGAAGGTAATCAAGGTTACGTTG<br>GACAATGGTGAGTCTATTATTTGCACCCCGGATCATAAATTCATGTTGCGGGATGGGAGCTACA<br>AATGTGCGATGGATTTAACTCTCGATGATTCGTTAATGCCGTTACACCGAAAAATTTCGACTAC<br>GGAAGATTCTGGTCATGCGATGGAAGCAGTATTAAATTACAATCACAGAATTGTAAATATTGAA<br>GCTGTGTCAGAAACAATCGATGTTTATGATATTGAGGTTCCCCACACCCACAATTTTGCTTTGG<br>CAAGCGGAGTGTTTGTCCATAAC
TerThyX	CLSGNTKVRFRYSSSSQEAKYYEETIEKLANLWH<br>YGSKNQYTSKDAKCMQENISSRNIFTLDTQTNQI<br>VSSKITNIYINGEKETYTIKTVSGKEIRATLEHQ<br>FWTNQGWKRLKDFNNSTQLCEVQLAGVFVEIESI<br>EKFGKEITYDLEVEHPEHNFIANGLVVHN	TGTTTGTCGGGCAATACAAAAGTTAGATTTAGGTATTCGTCTTCATCCCAAGAAGCGAAATATT<br>ATGAGGAAACAATTGAAAAATTAGCTAACTTATGGCATTACGGGAGCAAAAATCAATATACTTC<br>TAAAGATGCAAAATGTATGCAAGAAAATATATCTAGCCGTAATATTTTTACTCTGGATACCCAA<br>ACTAACCAAATTGTTTCTAGCAAAATTACCAACATATATATTAATGGTGAAAAAGAAACTTACA<br>CAATAAAAACTGTTTCTGGTAAGGAAATAAGAGCTACCCTAGAACACCAGTTTTGGACTAACCA<br>AGGGTGGAAACGATTAAAAGATTTTAATAACAGCACTCAATTATGTGAAGTGCAATTAGCGGGT<br>GTTTTTGTAGAGATAGAATCTATTGAAAAATTTGGCAAAGAAATTACTTATGATCTTGAAGTCG<br>AACACCCAGAACATAATTTTATAGCCAATGGTTTAGTTGTTCATAAT
TvoVMA	CVSGETPVYLADGKTIKIKDLYSSERKKEDNIVE<br>AGSGEEIIHLKDPIQIYSYVDGTIVRSRSRLLYK<br>GKSSYLVRIETIGGRSVSVTPVHKLFVLTEKGIE<br>EVMASNLKVGDMIAAVAESESEARDCGMSEECVM<br>EAEVYTSLEATFDRVKSIAYEKGDFDVYDLSVPE<br>YGRNFIGGEGLLVLHN	TGCGTATCAGGTGAAACACCAGTTTACCTTGCGGATGGCAAGACAATAAAAATAAAGGATCTAT<br>ACAGTTCTGAGAGAAAAAAAGAAGATAACATTGTTGAGGCTGGTTCGGGAGAAGAGATAATACA<br>TCTAAAAGATCCCATTCAAATATATTCTTATGTGGACGGGACCATAGTCAGGAGCAGATCAAGA<br>CTTCTATACAAGGGCAAGAGCTCTTATCTTGTGAGGATAGAAACTATTGGCGGAAGATCGGTAA<br>GCGTTACACCAGTTCACAAACTCTTTGTCCTTACGGAAAAGGGTATCGAAGAGGTTATGGCCTC<br>CAACCTAAAGGTAGGCGACATGATTGCTGCTGTAGCAGAAAGCGAATCTGAAGCAAGAGACTGC<br>GGAATGAGCGAGGAATGCGTGATGGAAGCAGAAGTTTATACGTCACTTGAAGCGACATTCGATA<br>GAGTAAAGTCTATAGCGTACGAGAAGGGTGATTTTGATGTATACGATCTTTCCGTACCAGAATA<br>CGGCAGGAACTTTATAGGCGGAGAAGGACTTCTCGTACTTCACAAC

PhoRadA	CFARDTEVYYENDTVPHMESIEEMYSKYASMNGE<br>LPFDNGYAVPLDNVFVYTLDIASGEIKKTRASYI<br>YREKVEKLIEIKLSSGYSLKVTPSHPVLLFRDGL<br>QWVPAAEVKPGDVVVGVREEVLRRRIISKGELEF<br>HEVSSVRIIDYNNWVYDLVIPETHNFIAPNGLVL<br>HN	TGCTTTGCTAGGGATACCGAAGTTTATTATGAAAACGATACGGTACCACACATGGAATCAATTG<br>AGGAGATGTATAGTAAATACGCCTCGATGAATGGGGAATTACCATTCGATAATGGTTACGCAGT<br>TCCATTAGATAATGTCTTTGTGTACACGTTGGACATCGCTAGTGGTGAAATTAAGAAAACGAGG<br>GCTTCATACATCTATCGCGAGAAGGTTGAGAAGCTCATTGAGATAAAACTATCCAGTGGATACT<br>CCCTTAAGGTTACTCCCTCTCACCCAGTTCTCCTCTTTAGAGATGGCCTGCAATGGGTCCCCGC<br>TGCTGAAGTTAAACCTGGAGATGTTGTTGTTGGTGTTAGGGAAGAGGTACTGAGAAGAAGAATA<br>ATATCCAAAGGAGAACTTGAATTCCATGAGGTTTCCTCGGTAAGGATAATAGATTACAATAACT<br>GGGTCTACGACCTTGTAATTCCGGAAACCCACAACTTCATAGCTCCCAATGGACTTGTTCTCCA<br>TAAT
Cro-RIR1	CFTPDTPIFTNDGFVSIENIKPHMKVMTSDGTFR<br>NVNKIFKNNVNKNILKINTTHSLEEIKCTKEHDI<br>LIYQNINNESNYEQITHYIETNKYTPNFVKASEL<br>KVGDFMVIPKIQI**GSAGSAAGSG**CILTKIENIDM<br>CEYSGYVYDLNIEENHNYLTSSGIVHN	GCCTCCTCCGAGCGGATGTACCCCGAGGACGTGAATCAGTGCTTTACGCCCGACACACCAATTT<br>TTACGAACGATGGCTTTGTATCGATTGAAAACATAAAGCCTCATATGAAGGTTATGACCTCTGA<br>CGGTACGTTCCGCAATGTTAATAAAATTTTTAAAAACAACGTCAATAAGAATATTCTAAAGATC<br>AATACGACTCACTCATTAGAAGAAATCAAATGTACAAAAGAGCATGATATTTTAATCTATCAGA<br>ATATTAATAATGAATCTAACTACGAACAAATTACACATTATATTGAAACGAACAAATATACTCC<br>TAACTTTGTAAAAGCATCCGAGTTAAAGGTAGGTGATTTCATGGTAATCCCGAAAATCCAGATT<br>GGGTCCGCGGGGAGCGCGGCCGGTAGCGGATGCATACTGACGAAGATCGAAAACATTGATATGT<br>GTGAGTACTCCGGCTATGTCTATGATTTGAACATCGAAGAAAACCACAATTATTTAACCTCCTC<br>GGGTATTGTTCATAACTCCGGGAGGGGCGCCCTGAAGGGCGAGATCAAGCAGAGG
Cro-RPB2	CQKYDTLVLTHLGWIKLGEIDITIHKVATLDKHD<br>NIIYVYPTSKFEFDYDGDFYEHKNNSIDIECTIN<br>HKLYCKYNLSSSFTLIPADKVYGNKVIMKNMENE<br>VIWTDP**GSAGSAAGSG**SKEQIHNYKGKVYCIEVP<br>DSHIYYMKTSSITPPVWIGN	GCCTCCTCCGAGCGGATGTACCCCGAGGACGATAAATTCTGCCAGAAATATGATACACTGGTAC<br>TTACTCACCTGGGATGGATCAAACTTGGCGAAATCGATATAACTATTCATAAAGTTGCTACGTT<br>GGACAAGCACGATAACATCATCTACGTTTATCCTACGTCTAAATTTGAATTTGACTATGACGGG<br>GACTTTTACGAGCATAAAAATAATAGCATCGATATTGAATGCACAATTAATCATAAATTATACT<br>GCAAATACAACCTGAGCTCATCATTTACCCTGATTCCAGCAGATAAAGTGTATGGTAACAAAGT<br>GATTATGAAAAATATGGAAAATGAAGTGATCTGGACGGATCCGGGTTCTGCTGGCAGCGCAGCC<br>GGGTCGGGGAGTAAAGAACAAATTCACAACTATAAAGGCAAAGTGTATTGTATAGAAGTGCCAG<br>ATTCCCATATTTATTATATGAAGACCAGCTCTATCACTCCACCAGTTTGGATAGGCAATTCAAC<br>CAAAGGCGCCCTGAAGGGCGAGATCAAGCAGAGG
Cro-Top2	CISSDTNVLIWNSKISKKAKDIQIGDILVGDDGN<br>KRNVIDVSFGKGQMYKIIQSKGENYSVNNNHTLT<br>LMMPLHKVISNSNNKLKLLWWDNVYKIIKSKPID<br>VQENKISDDEILLNSVYHDR**GSAGSAAGSG**LDSP<br>GYITVEKDGIGDYVSITVDTTTNQRFLINDFTVT<br>HN	GCCTCCTCCGAGCGGATGTACCCCGAGGACGGCAGTAAGTGTATATCCTCCGATACCAACGTGC<br>TGATATGGAACAGTAAAATCTCTAAAAAAGCCAAAGACATTCAAATCGGTGATATACTGGTAGG<br>TGACGATGGTAACAAGCGCAATGTTATCGATGTTAGCTTTGGAAAAGGACAGATGTACAAAATC<br>ATTCAGTCCAAAGGTGAAAATTATAGCGTTAATAACAACCATACTCTTACGCTGATGATGCCAC<br>TGCACAAAGTAATTAGCAATAGTAACAATAAGCTGAAATTACTCTGGTGGGATAATGTGTATAA<br>AATTATTAAATCCAAACCGATTGATGTGCAAGAAAACAAAATTAGCGATGATGAAATTCTCCTG<br>AACAGTGTCTATCATGACCGCGGCTCAGCCGGTAGTGCCGCTGGTTCCGGGCTGGATTCGCCAG<br>GGTATATCACGGTTGAGAAAGATGGTATTGGCGATTATGTGAGTATTACAGTGGATACGACGAC<br>GAATCAACGATTTCTGATTAACGATTTTACGGTCACGCATAATTGCTCAAACGGCGCCCTGAAG<br>GGCGAGATCAAGCAGAGG
CIV-RIR1	CVAPETMILTEDGQFPIKDLEGKIIKVWNGNEFS<br>SVTVVKTGTEKELLEVELSNGCTLSCTPEHKFII<br>VKSYTEAKKQKTDDNAIANAERVDAQDLKPRMKL<br>IKFDLPTLFGNS**GSAGSAAGSG**RFVEVLKVNKTG<br>RVDDTYCFTEPINHAGVFNGILTGQ	GCCTCCTCCGAGCGGATGTACCCCGAGGACTCGAATCTCTGCGTTGCACCCGAAACCATGATCT<br>TGACCGAAGACGGTCAGTTTCCGATTAAGGACCTGGAGGGGAAGATAATTAAGGTTTGGAATGG<br>CAACGAATTTAGCTCTGTCACAGTTGTAAAGACTGGCACTGAGAAAGAGCTGCTGGAGGTGGAG<br>TTGAGTAATGGTTGTACATTAAGCTGTACTCCGGAACATAAATTTATCATTGTAAAAAGTTACA<br>CCGAAGCAAAAAAACAAAAAACCGATGATAACGCGATCGCCAATGCCGAACGCGTAGACGCGCA<br>GGATTTGAAACCTAGAATGAAGCTAATCAAATTCGATCTGCCGACGCTGTTTGGTAATAGCGGG<br>AGTGCGGGCAGCGCAGCAGGTTCGGGACGTTTTGTTGAGGTCCTGAAGGTGAATAAAACGGGTC<br>GAGTCGACGATACGTATTGCTTTACGGAACCGATTAATCATGCGGGGGTGTTTAACGGGATCCT<br>GACCGGTCAATGCTCAGAGGGCGCCCTGAAGGGCGAGATCAAGCAGAGG
CP-Thy1	CYSSDTEVLTSEGWKRWEEVSMNDSFATLSASGQ<br>VEYQYPSEVIQEEYQGDMVRAKAKGVDLLVTPNH<br>KMLACITTTRAGRRKENFSLIPADQLIGVSHAYK<br>KDGDWRA**GSAGSAAGSG**GASKTSYGWEKYSGTVY<br>CATVPNHTLYVRRNGKPVWSGN	GCCTCCTCCGAGCGGATGTACCCCGAGGACAAGCATCACTGTTATAGTTCCGACACTGAGGTCC<br>TGACAAGCGAAGGCTGGAAACGTTGGGAAGAAGTAAGTATGAACGATTCGTTCGCTACTTTAAG<br>CGCATCCGGGCAGGTAGAGTACCAGTACCCTAGCGAGGTCATTCAGGAAGAATATCAGGGGGAC<br>ATGGTAAGAGCTAAAGCCAAGGGCGTAGACCTTCTGGTTACTCCAAACCACAAAATGTTGGCCT<br>GTATTACAACCACCCGCGCAGGACGTCGGAAAGAAAATTTTCGCTGATACCGGCGGATCAATT<br>GATTGGCGTGAGCCATGCGTATAAAAAGGACGGGGACTGGCGGGCTGGTTCTGCTGGTTCCGCC<br>GCTGGAAGCGGAGGAGCTTCAAAGACCAGTTACGGTTGGGAGAAATATTCAGGAACCGTGTATT<br>GCGCGACGGTACCGAATCATACCCTGTATGTACGTAGGAATGGTAAGCCAGTATGGTCGGGTAA<br>CTCAGTCCTTGGCGCCCTGAAGGGCGAGATCAAGCAGAGG
Ama-Ter	NIAHSTPVLTANRGWVTHGDLVPGDQVFHPSGKP<br>VDVLALSDEAVDDYVVTFTNGEKIRCHANHEWTV<br>YSRADKQEKTVETKWFLENTNRGTPRSLTAGNRF<br>QFQVPKTNALE**GSAGSAAGSG**ERRVSIEKVEYLP<br>NGEKGHCIQVDSPDGLYLVGKKLVATHN	GCCTCCTCCGAGCGGATGTACCCCGAGGACCATGGTAAAAATATTGCACATAGCACCCCTGTGC<br>TGACTGCGAACCGTGGGTGGGTGACACATGGGGATCTAGTTCCTGGAGATCAGGTTTTCCATCC<br>GAGCGGCAAACCCGTGGACGTGCTGGCGCTTTCGGATGAGGCTGTAGATGACTACGTCGTAACT<br>TTTACGAATGGGGAAAAAATCAGATGCCATGCTAATCATGAATGGACAGTTTATTCCCGCGCGG<br>ATAAACAGGAAAAGACGGTCGAAACAAAATGGTTTCTTGAGAATACGAACCGCGGAACTCCACG<br>CTCCTTAACCGCGGGAAATCGTTTTCAGTTTCAGGTTCCAAAGACAAACGCCCTGGAAGGATCC<br>GCGGGTTCTGCGGCCGGTTCTGGCGAGCGCCGCGTTTCCATAGAGAAAGTAGAATATCTTCCTA<br>ACGGGGAGAAAGGCCATTGCATTCAGGTGGATAGCCCAGACGGTCTGTATTTAGTAGGGAAGAA<br>ACTTGTTGCCACCCACAACTCCGAGCTGGGCGCCCTGAAGGGCGAGATCAAGCAGAGG
BsuP-RIR1	CVTGETLLLTENGYEKAADLYKKQNNLKVVIDNR<br>TKDFAVDSKGTTIVDAIPMQLTKKDAEIFKVKTK<br>QGYEIRATEWHKFYVKRDGEIQKLQLNQLKTGDK<br>LLVQSAEGA**GSAGSAAGSG**DFTAEIISIEEDGVE<br>DVYDTTQEDYHSLIFNGIVTGN	GCCTCCTCCGAGCGGATGTACCCCGAGGACAGCAATCTGTGTGTGACGGGTGAAACCCTCCTTC<br>TGACCGAGAACGGCTATGAAAAAGCTGCTGATCTCTATAAGAAGCAGAATAATTTAAAAGTTGT<br>CATCGATAATAGGACAAAGGACTTCGCGGTAGATAGTAAAGGGACCACTATTGTAGATGCTATT<br>CCAATGCAATTGACGAAAAAAGATGCCGAAATTTTTAAAGTTAAAACTAAGCAGGGGTATGAAA<br>TTCGGGCTACGGAATGGCATAAGTTTTATGTTAAACGCGACGGTGAGATTCAGAAATTACAACT<br>GAATCAGCTGAAGACAGGTGATAAGCTCCTGGTCCAGAGTGCCGAGGGCGCTGGCAGCGCAGGT<br>TCCGCCGCTGGCTCAGGTGATTTTACGGCGGAGATTATCTCTATTGAAGAAGATGGAGTGGAAG<br>ACGTATATGATACAACTCAAGAGGATTATCATTCGCTGATTTTTAACGGCATTGTAACTGGTAA<br>TTGCAGTGAAGGCGCCCTGAAGGGCGAGATCAAGCAGAGG
Cbp-RNR	CFDGQQKTLTKSSHGVNYLSFKDLYDTKYKDKER<br>RNFKVFHNGNWVEGKPIRLLRNDKKMYKITTVNN<br>KEILVTEDHINVTDKGDKYTTQLTENDYIAFNTR<br>PTNAI**GSAGSAAGSG**TMFFKIKTIQQYISNDEYV<br>YCFEMKNIEEPYFTLPNGIITHN	GCCTCCTCCGAGCGGATGTACCCCGAGGACGCTAGCTGTTGCTTCGACGGTCAGCAAAAGACAC<br>TCACAAAATCATCTCATGGGGTTAACTACCTATCCTTCAAGGATCTGTACGATACCAAGTATAA<br>AGATAAAGAACGGCGTAATTTTAAAGTTTTTCACAATGGAAATTGGGTCGAAGGGAAGCCTATA<br>AGACTGCTGAGAAATGATAAAAAAATGTACAAAATTACGACTGTGAATAATAAAGAAATCTTAG<br>TTACGGAAGATCATATCAATGTAACCGATAAAGGAGATAAATACACCACACAATTAACCGAAAA<br>TGATTACATAGCCTTCAATACTAGACCCACAAATGCCATCGGCTCGGCGGGGTCGGCTGCCGGC<br>TCAGGTACCATGTTTTTTAAGATCAAAACAATACAACAGTATATTTCCAATGATGAATACGTTT<br>ATTGTTTTGAAATGAAAAACATCGAGGAACCGTACTTTACCTTACCCAATGGTATAATAACGCA<br>CAATTGTCGTCTCGGCGCCCTGAAGGGCGAGATCAAGCAGAGG

Ckl-Ter	ALDLDTPIDTPTGWKTMRDIECGDYVFGVDGKPT<br>KVIGTSDIMVNHECYKVTFEDGEFIIADTEHIWT<br>VTTKSSRKTLKYKPLKGRQLLRPDYRESNGYFDV<br>TTGEMAKNFKHFRKDGKGIEYKYRVPMAGAVE**GS**<br>**AGSAAGSG**KNKSVINIEKYKSVPVKCIAVEDEKK<br>LYLAGKNHTATHN	GCCTCCTCCGAGCGGATGTACCCCGAGGACAATGGAAAGCGCTTGATCTTGACACGCCCATAG<br>ACACGCCCACGGGGTGGAAAACCATGCGTGACATTGAGTGTGGAGACTATGTTTTTGGAGTTGA<br>TGGTAAACCAACGAAAGTCATTGGAACGTCGGATATAATGGTTAATCATGAATGTTACAAAGTT<br>ACCTTTGAAGATGGAGAGTTTATCATCGCAGATACCGAGCATATTTGGACTGTGACAACAAAAT<br>CGTCACGGAAAACCTTGAAATATAAGCCTCTTAAAGGGCGGCAACTTCTGCGTCCAGATTATCG<br>TGAATCAAATGGATATTTTGATGTAACGACCGGTGAAATGGCCAAGAATTTCAAGCATTTTCGC<br>AAAGACGGAAAGGGTATCGAGTATAAATACAGAGTACCGATGGCCGGCGCAGTTGAGGGTAGCG<br>CAGGTTCAGCAGCAGGATCAGGTAAAAACAAATCAGTAATTAACATAGAAAAATACAAATCCGT<br>TCCTGTAAAGTGTATCGCTGTGGAAGACGAAAAAAAATTGTACCTGGCAGGCAAAAACCACACT<br>GCCACTCACAACACTACCCTCGGCGCCCTGAAGGGCGAGATCAAGCAGAGG
Cth-Ter	QLALDTPIPTPDGWTTMGEIKAGDKVIDEKGRPC<br>NVVAISEIDDTEQAYKINFRDGTSIVAGERHLWK<br>VQVTNNGRREKLLTTGEMYQKQFKTKSKENRALF<br>RIPIADAFI**GSAGSAAGSG**SHFHYIKSIEKTGKT<br>KMRCIQVDSPSRLYLAGKSMIPTHN	GCCTCCTCCGAGCGGATGTACCCCGAGGACAATGGAAGCAGCTTGCGCTTGATACACCTATCC<br>CTACTCCAGATGGTTGGACAACAATGGGAGAGATCAAAGCCGGGGACAAGGTAATCGATGAAAA<br>AGGTCGCCCCTGCAATGTGGTAGCGATCAGCGAAATCGATGATACCGAACAGGCTTACAAGATT<br>AATTTCCGTGATGGTACAAGTATAGTCGCTGGAGAACGCCATCTGTGGAAAGTCCAGGTTACTA<br>ATAATGGCCGGCGAGAAAAGTTGCTGACCACTGGTGAAATGTACCAAAAACAGTTTAAAACTAA<br>GTCTAAAGAAAACCGTGCCCTGTTTCGTATACCGATCGCGGACGCGTTCATTGGAAGTGCGGGA<br>TCCGCGGCCGGAAGCGGATCTCACTTCCACTACATAAAGTCTATTGAAAAAACCGGGAAAACCA<br>AGATGCGGTGTATACAGGTGGATAGTCCTTCCCGTCTGTATCTCGCCGGCAAATCTATGATACC<br>AACGCACAATTCGGAGTTAGGCGCCCTGAAGGGCGAGATCAAGCAGAGG
EP-Pri	ACPLNEPILLADGTWTTHGDVKIGDQVASVDGNP<br>STVTGIFPQGVRDVYRVTFEDGRYVDCAGDHLWE<br>VTSRGFTKGEKRRVIDTFELKRLSETKRHKNGVR<br>IPEITGD**GSAGSAAGSG**GCGVFVRNVEKIGNAEC<br>LCIMVDHPRHLYVTRGYVATHN	GCCTCCTCCGAGCGGATGTACCCCGAGGACCACGGTAAAGCGTGTCCCTTAAATGAACCTATCC<br>TCCTGGCAGATGGCACATGGACTACCCATGGTGATGTCAAAATCGGGGACCAAGTTGCGAGTGT<br>CGATGGCAACCCTTCCACAGTCACCGGCATATTTCCGCAAGGCGTCCGTGATGTTTATCGCGTG<br>ACGTTTGAAGATGGTAGATATGTCGATTGTGCAGGTGATCATCTATGGGAAGTGACCTCGAGAG<br>GGTTTACGAAAGGCGAGAAGCGCCGCGTTATAGACACGTTCGAATTAAAAAGGCTGTCGGAGAC<br>CAAGCGTCATAAGAACGGTGTTCGTATCCCAGAAATCACCGGAGATGGTTCGGCCGGCAGTGCA<br>GCGGGTTCGGGAGGCTGCGGTGTTTTTGTTAGAAATGTCGAAAAAATAGGAAACGCAGAATGTT<br>TATGTATTATGGTTGACCACCCACGGCATCTTTACGTGACGCGCGGATATGTGGCCACCCATAA<br>TACCGAGGTCGGCGCCCTGAAGGGCGAGATCAAGCAGAGG
LLP-Pol	CFSGDTEILTPYGWVKFEDYDESMMCAQYDEVTG<br>KISFTYPNETIHLKDQDIWVYEDTNTSIHATGNH<br>DILIQKPNGDIAKEKFSNLQLLQKGDKHRFINAG<br>YVDSAEEVDTLMQRLV**GSAGSAAGSG**SFPGKAFP<br>TGPSYKGDVYCVNVPTHNIVIRHNDKVSIQGN	GCCTCCTCCGAGCGGATGTACCCCGAGGACGCCAAGGCATGCTTTTCAGGAGATACCGAAATCT<br>TGACGCCATACGGTTGGGTGAAGTTTGAAGATTACGACGAAAGCATGATGTGTGCTCAATACGA<br>TGAGGTTACCGGCAAAATTAGTTTTACCTATCCGAATGAAACAATACACCTGAAGGATCAGGAC<br>ATCTGGGTGTATGAGGATACAAATACCTCCATTCATGCGACCGGTAACCACGATATACTGATTC<br>AGAAACCAAACGGAGATATTGCTAAGGAAAAGTTTTCTAACTTACAACTGTTACAGAAAGGCGA<br>TAAACATAGATTCATTAATGCGGGATACGTGGATTCGGCCGAAGAGGTAGACACTCTCATGCAG<br>CGTCTGGTTGGGAGCGCGGGCAGCGCGGCAGGCTCCGGATCTTTCCCTGGGAAGGCATTCCCTA<br>CAGGCCCGAGTTATAAAGGGGACGTGTATTGCGTCAACGTCCCCACTCATAATATTGTAATTCG<br>TCACAACGATAAGGTGAGCATACAGGGCAACTGCAATTTCGGCGCCCTGAAGGGCGAGATCAAG<br>CAGAGG
LP-Hel	CHAYGHDIMMSDGTKKQVQDIAVGDKVMGPDGNP<br>RKVIRLVKGQDEMFRVTPTKGESFVVNGGHILSL<br>YQTPRRAGQTPGYTEISVNEYIRSSSTFKHRSKL<br>QRFGFD**GSAGSAAGSG**RVDVTGFKVEPIGVGDYY<br>GFTVDGDHLYLDGDFVRHHN	GCCTCCTCCGAGCGGATGTACCCCGAGGACGCCACTGGGTGCCATGCTTACGGACATGATATAA<br>TGATGTCCGATGGTACTAAGAAACAGGTTCAGGATATTGCAGTAGGCGACAAGGTTATGGGTCC<br>TGATGGTAATCCTAGAAAAGTCATTCGGCTGGTGAAAGGTCAAGATGAGATGTTTCGTGTTACT<br>CCGACGAAAGGCGAATCCTTTGTTGTAAACGGTGGTCACATCCTGTCGCTGTATCAAACGCCAC<br>GGCGCGCCGGACAGACCCCGGGATACACCGAGATCTCCGTCAATGAATATATAAGGTCTAGTTC<br>CACTTTTAAGCACCGTTCAAAATTACAGCGCTTTGGCTTCGATGGGTCTGCGGGCAGTGCCGCT<br>GGGAGTGGTCGCGTTGACGTAACGGGGTTTAAAGTTGAGCCCATTGGCGTGGGGGATTACTATG<br>GCTTTACCGTCGACGGTGATCACTTGTACCTTGATGGGGATTTCGTCAGACATCATAATTGCGG<br>GAAGGGCGCCCTGAAGGGCGAGATCAAGCAGAGG
MP-B-DnaB	PLALNTEVPTPSGWTTVGDLSVGDYVLGSDGQPH<br>RVQRETPVLEGLATYVVRFDDGTEITASASHGWT<br>TQRLTGHGDSYETVTVTTEELAQTVTNSKGRKRH<br>RIPVVGME**GSAGSAAGSG**SQHRWVESVTPVESVP<br>VKCIGIDTEDHLFQVSRSRILTHN	GCCTCCTCCGAGCGGATGTACCCCGAGGACCAGGATCAGCCATTGGCACTAAATACTGAGGTTC<br>CCACACCGAGTGGATGGACAACCGTTGGTGATCTGAGCGTCGGGGATTATGTTCTCGGCAGTGA<br>CGGCCAGCCCCATCGGGTTCAGAGGGAGACTCCGGTCCTTGAAGGGCTTGCTACGTATGTGGTA<br>AGGTTTGACGACGGGACAGAAATTACAGCGAGTGCTAGCCACGGCTGGACCACTCAACGTCTGA<br>CGGGCCATGGCGATTCTTACGAAACCGTAACTGTAACCACCGAAGAGCTGGCTCAGACTGTTAC<br>AAATTCCAAGGGACGTAAACGTCATCGCATCCCTGTAGTGGGTATGGAAGGGTCGGCAGGAAGT<br>GCTGCTGGTAGTGGCTCCCAGCATCGGTGGGTTGAATCAGTAACACCCGTTGAGAGCGTTCCGG<br>TTAAATGCATTGGTATTGATACCGAAGATCATCTTTTCCAGGTGAGTCGCAGCCGAATCTTAAC<br>ACACAACACCAAAAATGGCGCCCTGAAGGGCGAGATCAAGCAGAGG
MP-K-gp53	CSWTNARAVTRRGFVHVDDLTTDDEVMSVDDQGR<br>TIWQQIDEVVRFPFSGTLYSLGGREINATITANH<br>RVVGLNREKTKWVEHTPTSLPGNKMWVYTAGEGS<br>**GSAGSAAGSG**LYKNTVEEVAYEGEVWCLRVPNGR<br>FFIEDGGKIHLTGN	GCCTCCTCCGAGCGGATGTACCCCGAGGACAACCATGATTGCTCATGGACAAACGCACGGGCTG<br>TTACCAGAAGGGGGTTTGTACACGTTGACGATCTTACAACGGATGATGAGGTTATGTCTGTTGA<br>TGATCAGGGGCGTACGATTTGGCAGCAAATAGACGAAGTGGTACGCTTCCCATTTAGCGGAACC<br>TTATATTCACTGGGTGGCAGAGAAATTAACGCCACAATAACTGCCAATCACCGCGTGGTCGGAC<br>TTAATCGCGAAAAAACTAAATGGGTGGAACATACACCAACGTCACTGCCGGGCAATAAGATGTG<br>GGTGTACACAGCTGGGGAAGGGAGTGGAAGCGCCGGCTCGGCGGCCGGTTCAGGCTTATACAAG<br>AATACTGTGGAAGAAGTTGCATACGAAGGCGAAGTTTGGTGCCTGAGAGTCCCTAACGGTCGTT<br>TTTTCATTGAAGACGGTGGCAAGATTCACTTAACCGGAAATTCACGGGCGGGCGCCCTGAAGGG<br>CGAGATCAAGCAGAGG
MP-C-gp206	SLACSTPILTTNGWSTMGALQDGDEVYAPDGQPT<br>KVIKAHPINLNRPCFKVRFRDGQEVVTDAEHLWQ<br>VNDRNNGGRDRVMTTQEIADAPWGGRYRFRVPVT<br>EPLQ**GSAGSAAGSG**LRLNAIVAVEPVETVPVRCI<br>TVAHESSLYVAGEGFVPTHN	GCCTCCTCCGAGCGGATGTACCCCGAGGACGAGCTGAAGAGCCTAGCATGTTCCACACCTATTT<br>TGACGACAAACGGCTGGTCCACTATGGGGGCTTTGCAGGATGGTGACGAAGTCTACGCGCCAGA<br>CGGACAGCCAACAAAAGTTATAAAGGCCCACCCGATCAACCTGAATAGACCTTGCTTTAAAGTG<br>CGGTTTCGTGACGGTCAGGAAGTGGTTACCGATGCCGAGCACCTTTGGCAGGTAAACGATCGCA<br>ATAATGGTGGCCGCGATCGTGTTATGACAACTCAGGAGATCGCAGATGCTCCCTGGGGAGGGCG<br>ATATCGATTCCGTGTTCCGGTGACTGAGCCATTACAGGGGTCTGCGGGCTCGGCTGCTGGCTCG<br>GGACTGCGTTTAAACGCCATTGTTGCAGTAGAACCGGTGGAAACCGTCCCGGTGCGGTGCATCA<br>CCGTTGCTCATGAATCATCTTTATATGTTGCAGGTGAAGGATTTGTACCGACTCATAACACACA<br>AAATGGCGCCCTGAAGGGCGAGATCAAGCAGAGG
MP-M-DnaB	ALDVETPILTGNGWKKMGDIQVGDYVHAADGTLA<br>RVSYVSERHWRDCFSVQFADGAELVASDHHLWAV<br>NDRLKGERVIDTAELYRTQTYGARGDRRYTVTVP<br>EALD**GSAGSAAGSG**ARTNTITSVTPVPTVETVCI<br>QIDHPSHVFLAGKSLTPTHN	GCCTCCTCCGAGCGGATGTACCCCGAGGACAACGGAAAGCGTTGGATGTTGAAACACCAATCC<br>TCACTGGCAACGGTTGGAAAAAGATGGGCGACATACAAGTCGGGGACTACGTGCACGCCGCTGA<br>TGGCACACTCGCCCGCGTGAGCTATGTCTCCGAACGTCACTGGCGTGATTGTTTTAGTGTGCAG<br>TTTGCGGATGGGGCAGAATTGGTTGCATCAGACCATCACTTGTGGGCGGTTAACGATCGTCTCA<br>AAGGGGAGCGCGTTATTGACACAGCCGAACTGTACCGCACCCAAACGTACGGGGCCCGCGGCGA<br>TCGTCGTTACACTGTTACGGTGCCAGAAGCTTTGGATGGCAGTGCGGGGTCAGCGGCAGGCAGT<br>GGGGCGCGAACGAATACAATTACCTCTGTGACGCCTGTGCCTACCGTAGAGACCGTATGTATAC<br>AGATTGATCATCCGTCGCATGTGTTTCTGGCTGGTAAAAGTCTGACCCCAACTCACAACACAGA<br>ATTGGGCGCCCTGAAGGGCGAGATCAAGCAGAGG

PP-Phi	AGVLSSKIKIPGGWKTMGNIRVGDEVVTPDGGTA<br>KVLAVHPQGVTKVVRVHFKDGRYTDVSPDHLWKV<br>RRHHWCNDKAMAKLTREEVEERVWRVITTNELKD<br>YIGLSTKVYVQLIEPER**GSAGSAAGSG**TLRLQVT<br>HIEERPDEETQCITIDHPDHLYITDDFIVTHN	GCCTCCTCCGAGCGGATGTACCCCGAGGACGCAGGCAAGGCAGGAGTGCTGTCGTCGAAAATAA<br>AAATCCCTGGGGGGTGGAAAACGATGGGCAATATCCGCGTTGGCGATGAAGTAGTGACACCGGA<br>CGGCGGCACAGCGAAGGTGTTAGCCGTTCATCCGCAAGGCGTAACCAAAGTTGTCCGTGTGCAT<br>TTCAAAGATGGCCGCTATACTGATGTCTCCCCCGATCACCTCTGGAAAGTGAGGCGTCATCACT<br>GGTGTAATGATAAAGCGATGGCGAAACTGACTCGGGAAGAGGTTGAGGAGCGTGTGTGGAGAGT<br>GATAACTACGAATGAGCTGAAAGATTATATTGGACTTTCCACAAAAGTTTATGTTCAACTGATA<br>GAGCCCGAACGTGGCTCGGCGGGCTCAGCCGCGGGCTCAGGTACACTCCGTCTTCAAGTTACAC<br>ATATTGAGGAGCGTCCAGACGAAGAGACACAGTGCATTACCATCGACCATCCGGATCATTTGTA<br>TATTACTGATGATTTCATCGTTACGCACAACTCCCTTACTGGCGCCCTGAAGGGCGAGATCAAG<br>CAGAGG
SaP-dpol	CLHRHTQVLTDGGFKDIMAVTSTDKVWSGEKWVN<br>TKGAHLMGWKPVINVDGVLMTEDHKILTHSWKQA<br>KQLVSNKYMMDRALEIGMDAWLSCASYQNDKAKD<br>NYSSNVIVERCLG**GSAGSAAGSG**NLNGKLTYCEP<br>VYDLIDVEDGNRFLIASDSGFLVAHN	GCCTCCTCCGAGCGGATGTACCCCGAGGACGGAAAGGCCTGCCTGCATCGACACACTCAGGTTC<br>TTACTGATGGCGGATTTAAAGATATCATGGCCGTTACCAGTACTGATAAAGTCTGGAGCGGCGA<br>AAAATGGGTTAATACTAAGGGGGCACATCTGATGGGGTGGAAACCGGTTATTAATGTCGATGGT<br>GTTCTCATGACCGAAGATCACAAAATTCTCACCCACAGCTGGAAACAGGCAAAGCAGCTGGTTT<br>CTAACAAATATATGATGGATCGTGCTCTCGAAATAGGCATGGATGCATGGCTCTCTTGTGCGAG<br>TTATCAAAATGATAAAGCCAAGGACAATTACAGTAGTAACGTTATTGTGGAACGGTGCCTGGGC<br>GGCAGTGCCGGGTCTGCTGCGGGCTCAGGCAATCTGAACGGCAAACTGACGTACTGTGAACCAG<br>TTTATGATCTAATAGATGTGGAGGACGGTAACCGATTCCTAATTGCGTCAGATTCCGGTTTTTT<br>AGTGGCTCATAACTGCGAACTGGGCGCCCTGAAGGGCGAGATCAAGCAGAGG
SaP-Hel	CLKRGTEVIMFDGTTKKVEDVIVGDVLMGPDSTP<br>RNVLSLGRGREMMYEVKPRKGESYTVNESHILSL<br>RTTTGIAKGSWPDNTVFDISVRDWLKLPKYVTGP<br>NGYLKGWRVPVDFPR**GSAGSAAGSG**VLNVGIESI<br>TPVGVDDYFGFTIDGDSRFLLGDFTVTHN	GCCTCCTCCGAGCGGATGTACCCCGAGGACAGCGGCAAGTGCCTGAAACGCGGAACCGAAGTAA<br>TCATGTTTGATGGTACTACTAAGAAAGTAGAAGACGTGATTGTTGGAGATGTGCTTATGGGCCC<br>AGACTCTACCCCGAGAAACGTCCTGTCCCTGGGGCGAGGGCGTGAAATGATGTATGAAGTTAAA<br>CCGCGGAAAGGGGAGAGTTATACAGTTAATGAAAGTCATATCCTGTCCCTGCGAACGACCACTG<br>GAATCGCTAAAGGTTCCTGGCCGGATAATACAGTCTTCGACATCAGTGTGCGTGATTGGCTGAA<br>ACTGCCGAAATATGTAACTGGTCCTAACGGTTATCTGAAGGGTTGGCGCGTGCCTGTAGACTTC<br>CCTCGCGGGTCTGCCGGTTCTGCCGCTGGCTCCGGAGTTTTAAATGTGGGGATTGAATCCATTA<br>CGCCAGTCGGAGTGGATGATTATTTCGGCTTTACCATCGATGGCGATTCTCGGTTCTTATTGGG<br>TGATTTTACAGTTACCCATAACACGAGTTCTGGCGCCCTGAAGGGCGAGATCAAGCAGAGG
NrdA-2	CLTGDAKIDVLIDNIPISQISLEEVVNLFNEGKE<br>IYVLSYNIDTKEVEYKEISDAGLISESAEVLEII<br>DEETGQKIVCTPDHKVYTLNRGYVSAKDLKEDDE<br>LVFS**GSAGSAAGSG**GLKIIKRESKEPVFDITVKD<br>NSNFFANNILVHN	GCCTCCTCCGAGCGGATGTACCCCGAGGACTCAAACCTTTGTCTCACGGGCGATGCTAAAATCG<br>ACGTACTTATCGATAATATTCCTATCTCCCAGATATCCCTGGAGGAAGTTGTTAACCTGTTTAA<br>TGAAGGCAAAGAGATATATGTTTTGTCTTATAATATTGATACCAAGGAGGTTGAATATAAAGAA<br>ATTTCTGACGCAGGTCTCATCAGTGAATCTGCCGAAGTGCTGGAAATCATTGACGAAGAAACTG<br>GGCAGAAAATTGTTTGTACCCCTGATCATAAAGTGTATACTCTGAACCGGGGGTACGTTTCTGC<br>TAAGGATCTCAAAGAAGACGATGAGCTGGTGTTTAGCGGATCAGCCGGCAGCGCAGCTGGCTCG<br>GGCGGTTTAAAAATTATAAAACGTGAATCAAAGGAACCAGTGTTTGACATTACTGTCAAAGATA<br>ATAGCAATTTTTTTGCCAATAATATCTTGGTGCATAACTGCAATGAGGGCGCCCTGAAGGGCGA<br>GATCAAGCAGAGG
Pfu-RIR1	CIDGKAKIIFENEGEEHLTTMEEMYERYKHLGEF<br>YDEEYNRWGIDVSNVPIYVKSFDPESKRVVKGKV<br>NVIWKYELGKDVTKYEIITNKGTKILTSPWHPFF<br>VLTPDFKIVEKRADELKEGDILI**GSAGSAAGSG**L<br>HLIEGLEVVRHITTTNEPRTFYDLTVENYQNYLA<br>GENGMIFVHN	GCCTCCTCCGAGCGGATGTACCCCGAGGACGGGGGCGGCTGCATTGATGGTAAAGCGAAAATCA<br>TCTTCGAAAACGAGGGTGAAGAACACCTGACCACTATGGAAGAAATGTACGAACGTTATAAGCA<br>CCTGGGAGAATTTTACGATGAGGAATATAATAGGTGGGGAATCGACGTATCCAATGTGCCCATT<br>TATGTAAAGAGTTTCGATCCAGAATCAAAGCGAGTTGTCAAAGGTAAGGTTAATGTAATTTGGA<br>AATATGAACTTGGTAAAGACGTTACGAAATACGAGATTATTACTAACAAAGGTACAAAAATTCT<br>TACTTCGCCGTGGCATCCTTTTTTTGTCCTTACCCCGGACTTCAAAATTGTAGAAAAACGCGCG<br>GATGAATTAAAAGAGGGCGATATTCTCATCGGATCAGCGGGATCCGCTGCTGGATCTGGCCTTC<br>ACTTGATCGAAGGCTTGGAGGTGGTTAGGCATATCACCACCACCAACGAGCCGAGAACATTTTA<br>TGATTTGACCGTTGAGAATTATCAGAATTATCTGGCGGGTGAAAATGGGATGATCTTCGTTCAT<br>AATACAGGCTTAGGCGCCCTGAAGGGCGAGATCAAGCAGAGG
Mja-KlbA	ALAYDEPIYLSDGNIINIGEFVDKFFKKYKNSIK<br>KEDNGFGWIDIGNENIYIKSFNKLSLIIEDKRIL<br>RVWRKKYSGKLIKITTKNRREITLTHDHPVYISK<br>TGEVLEINAEMVKVGDYIYIPKNNT**GSAGSAAGS**<br>**G**INLDEVIKVETVDYNGHIYDLTVEDNHTYIAGK<br>NEGFAVSN	GCCTCCTCCGAGCGGATGTACCCCGAGGACCACGATGGAGCGCTGGCCTATGACGAACCCATCT<br>ATCTGTCGGATGGCAACATTATAAATATTGGTGAATTTGTTGATAAATTTTTCAAAAAGTACAA<br>AAACTCTATCAAAAAGGAAGATAACGGTTTTGGATGGATTGACATTGGCAATGAAAACATTTAC<br>ATTAAGTCGTTCAATAAGCTTAGTCTTATCATCGAAGATAAGCGCATTCTGCGGGTTTGGCGAA<br>AAAAATACTCAGGTAAATTAATTAAGATCACCACCAAAAATCGCCGCGAGATTACACTAACACA<br>CGACCACCCTGTTTATATCTCTAAAACCGGTGAGGTGCTGGAAATTAATGCCGAAATGGTTAAA<br>GTCGGTGATTACATTTACATCCCGAAAAATAACACGGGATCAGCCGGTTCAGCGGCCGGTTCGG<br>GAATTAACCTGGATGAAGTAATCAAAGTTGAAACAGTGGACTATAATGGCCACATTTATGATTT<br>AACCGTCGAGGATAATCACACCTACATCGCAGGTAAGAACGAGGGCTTCGCTGTTTCAAATTGC<br>TCGGGTGGCGCCCTGAAGGGCGAGATCAAGCAGAGG

[a] The flexible linker sequence is in bold.

[b] Sequences homologous to mCherry are in pink and sequences encoding the junctions sequence residues are underlined.

Supplementary Table 4 | Sequences for the N- and C-termini of the split inteins

Intein	Split site	Terminal	Sequence[a]
M86	S1	N1	CISGDSLISLAST
		C1	GKRVPIKDLLGEKDFEIWAINEQTMKLESAKVSRVFCTGKKLVYTLKTRLGRTIKATANHRFLTIDGWKRLDELSLKEH<br>IALPRKLESSSLQLAPEIEKLPQSDIYWDPIVSITETGVEEVFDLTVPGLRNFVANDIIVHN
	S2	N2	CISGDSLISLASTGKRVPIKDLLGEKDFEIWAINEQTMKLESAKVSRVFCTGKKLVYTLKTRLGRTIKATANHRFLTID<br>GWKRLDELSLKEHIALPRKLE
		C2	SSSLQLAPEIEKLPQSDIYWDPIVSITETGVEEVFDLTVPGLRNFVANDIIVHN
	S3	N3	CISGDSLISLASTGKRVPIKDLLGEKDFEIWAINEQTMKLESAKVSRVFCTGKKLVYTLKTRLGRTIKATANHRFLTID<br>GWKRLDELSLKEHIALPRKLESSSLQLAPEIEKLPQSDIYWDPIVSITETGVEEVFDLTVPG
		C3	LRNFVANDIIVHN
NpuSsp	S1*	N1	CLSYETEILTVEY
		C1	GSLPIGKIVEKRIECTVYSVDNNGNIYTQPVAQWHDRGEQEVFEYCLEDGSLIRATKDHKFMTVDGQMLPIDEIFEREL<br>DLMRVDNLPN**GSAGSAAGSG**VKVIGRRSLGVQRIFDIGLRQDHNFLLANGAIAAN
	S2	N2	CLSYETEILTVEYGSLPIGKIVEKRIECTVYSVDNNGNIYTQPVAQWHDRGEQEVFEYCLEDGSLIRATKDHKFMTVDG<br>QMLPIDEIFERELDLMRVDNLPN
		C2	MVKVIGRRSLGVQRIFDIGLRQDHNFLLANGAIAAN
	S3*	N3	CLSYETEILTVEYGSLPIGKIVEKRIECTVYSVDNNGNIYTQPVAQWHDRGEQEVFEYCLEDGSLIRATKDHKFMTVDG<br>QMLPIDEIFERELDLMRVDNLPN**GSAGSAAGSG**VKVIGRRSLGVQRIFDIGLRQ
		C3	DHNFLLANGAIAAN
gp41-1	S1	N1	CLDLKTQVQTPQ
		C1	GMKEISNIQVGDLVLSNTGYNEVLNVFPKSKKKSYKITLEDGKEIICSEEHLFPTQTGEMNISGGLKEGMCLYVKEMML<br>KKILKIEELDERELIDIEVSGNHLFYANDILTHN
	S2	N2	CLDLKTQVQTPQGMKEISNIQVGDLVLSNTGYNEVLNVFPKSKKKSYKITLEDGKEIICSEEHLFPTQTGEMNISGGLK<br>EGMCLYVKE
		C2	LKKILKIEELDERELIDIEVSGNHLFYANDILTHN
	S3	N3	CLDLKTQVQTPQGMKEISNIQVGDLVLSNTGYNEVLNVFPKSKKKSYKITLEDGKEIICSEEHLFPTQTGEMNISGGLK<br>EGMCLYVKEMMLKKILKIEELDERELIDIEVSG
		C3	NHLFYANDILTHN
gp41-8	S1	N1	CLSLDTMVVTN
		C1	GKAIEIRDVKVGDWLESECGPVQVTEVLPIIKQPVFEIVLKSGKKIRVSANHKFPTKDGLKTINSGLKVGDFLRSRAKM<br>CEIFENEIDWDEIASIEYVGVEETIDINVTNDRLFFANGILTHN
	S2	N2	CLSLDTMVVTNGKAIEIRDVKVGDWLESECGPVQVTEVLPIIKQPVFEIVLKSGKKIRVSANHKFPTKDGLKTINSGLK<br>VGDFLRSRAK
		C2	MCEIFENEIDWDEIASIEYVGVEETIDINVTNDRLFFANGILTHN
	S3	N3	CLSLDTMVVTNGKAIEIRDVKVGDWLESECGPVQVTEVLPIIKQPVFEIVLKSGKKIRVSANHKFPTKDGLKTINSGLK<br>VGDFLRSRAKMCEIFENEIDWDEIASIEYVGVEETIDINVTN
		C3	DRLFFANGILTHN
NrdJ-1	S1	N1	CLVGSSEIITRNY
		C1	GKTTIKEVVEIFDNDKNIQVLAFNTHTDNIEWAPIKAAQLTRPNAELVELEIDTLHGVKTIRCTPDHPVYTKNRGYVRA<br>DELTDDDELVVAIMEAKTYIGKLKSRKIVSNEDTYDIQTSTHNFFANDILVHN
	S2	N2	CLVGSSEIITRNYGKTTIKEVVEIFDNDKNIQVLAFNTHTDNIEWAPIKAAQLTRPNAELVELEIDTLHGVKTIRCTPD<br>HPVYTKNRGYVRADELTDDDELVVAI
		C2	MEAKTYIGKLKSRKIVSNEDTYDIQTSTHNFFANDILVHN
	S3	N3	CLVGSSEIITRNYGKTTIKEVVEIFDNDKNIQVLAFNTHTDNIEWAPIKAAQLTRPNAELVELEIDTLHGVKTIRCTPD<br>HPVYTKNRGYVRADELTDDDELVVAIMEAKTYIGKLKSRKIVSNEDTYDIQTS
		C3	THNFFANDILVHN
IMPDH-1	S1	N1	CFVPGTLVNTEN
		C1	GLKKIEEIKVGDKVFSHTGKLQEVVDTLIFDRDEEIISINGIDCTKNHEFYVIDKENANRVNEDNIHLFARWVHAEELD<br>MKKHLLIELEMKFKLKEITSIETKHYKGKVHDLTVNQDHSYNVRGTVVHN
	S2	N2	CFVPGTLVNTENGLKKIEEIKVGDKVFSHTGKLQEVVDTLIFDRDEEIISINGIDCTKNHEFYVIDKENANRVNEDNIH<br>LFARWVHAEELDMKKHLLIELE
		C2	MKFKLKEITSIETKHYKGKVHDLTVNQDHSYNVRGTVVHN
	S3	N3	CFVPGTLVNTENGLKKIEEIKVGDKVFSHTGKLQEVVDTLIFDRDEEIISINGIDCTKNHEFYVIDKENANRVNEDNIH<br>LFARWVHAEELDMKKHLLIELEMKFKLKEITSIETKHYKGKVHDLTVNQ
		C3	DHSYNVRGTVVHN
SspDnaX	S1	N1	CLTGDSQVLTRN
		C1	GLMSIDNPQIKGREVLSYNETLQQWEYKKVLRWLDRGEKQTLSIKTKNSTVRCTANHLIRTEQGWTRAENITPGMKILS<br>PAPQWHTNFEEVESVTKGQVEKVYDLEVEDNHNFVANGLLVHN
	S2	N2	CLTGDSQVLTRNGLMSIDNPQIKGREVLSYNETLQQWEYKKVLRWLDRGEKQTLSIKTKNSTVRCTANHLIRTEQGWTR<br>AENITPGMKILSPA
		C2	PQWHTNFEEVESVTKGQVEKVYDLEVEDNHNFVANGLLVHN
	S3	N3	CLTGDSQVLTRNGLMSIDNPQIKGREVLSYNETLQQWEYKKVLRWLDRGEKQTLSIKTKNSTVRCTANHLIRTEQGWTR<br>AENITPGMKILSPAPQWHTNFEEVESVTKGQVEKVYDLEVED
		C3	NHNFVANGLLVHN

SspGyrB	S1	N1	CFSGDTLVALTD
		C1	GRSVSFEQLVEEEKQGKQNFCYTIRHDGSIGVEKIINARKTKTNAKVIKVTLDNGESIICTPDHKFMLRDGSYKCAMDLTLDDSLMPLHRKISTTEDSGHAMEAVLNYNHRIVNIEAVSETIDVYDIEVPHTHNFALASGVFVHN
	S2	N2	CFSGDTLVALTDGRSVSFEQLVEEEKQGKQNFCYTIRHDGSIGVEKIINARKTKTNAKVIKVTLDNGESIICTPDHKFMLRDGSYKCAMDLTLDDSLMPLHRKISTTEDSGHA
		C2	MEAVLNYNHRIVNIEAVSETIDVYDIEVPHTHNFALASGVFVHN
	S3	N3	CFSGDTLVALTDGRSVSFEQLVEEEKQGKQNFCYTIRHDGSIGVEKIINARKTKTNAKVIKVTLDNGESIICTPDHKFMLRDGSYKCAMDLTLDDSLMPLHRKISTTEDSGHAMEAVLNYNHRIVNIEAVSETIDVYDIEVPH
		C3	THNFALASGVFVHN
TerThyX	S1*	N1	CLSGNTKVRFRYS
		C1	SSSQEAKYYEETIEKLANLWHYGSKNQYTSKDAKCMQENISSRNIFTLDTQTNQIVSSKITNIYINGEKETYTIKTVSGKEIRATLEHQFWTNQGWKRLKDFNNSTQLCEVQLA**GSAGSAAGSG**GVFVEIESIEKFGKEITYDLEVEHPEHNFIANGLVVHN
	S2	N2	CLSGNTKVRFRYSSSSQEAKYYEETIEKLANLWHYGSKNQYTSKDAKCMQENISSRNIFTLDTQTNQIVSSKITNIYINGEKETYTIKTVSGKEIRATLEHQFWTNQGWKRLKDFNNSTQLCEVQLA
		C2	GVFVEIESIEKFGKEITYDLEVEHPEHNFIANGLVVHN
	S3*	N3	CLSGNTKVRFRYSSSSQEAKYYEETIEKLANLWHYGSKNQYTSKDAKCMQENISSRNIFTLDTQTNQIVSSKITNIYINGEKETYTIKTVSGKEIRATLEHQFWTNQGWKRLKDFNNSTQLCEVQLA**GSAGSAAGSG**GVFVEIESIEKFGKEITYDLEVEH
		C3	PEHNFIANGLVVHN
TvoVMA	S1	N1	CVSGETPVYLAD
		C1	GKTIKIKDLYSSERKKEDNIVEAGSGEEIIHLKDPIQIYSYVDGTIVRSRSRLLYKGKSSYLVRIETIGGRSVSVTPVHKLFVLTEKGIEEVMASNLKVGDMIAAVAESESEARDCGMSEECVMEAEVYTSLEATFDRVKSIAYEKGDFDVYDLSVPEYGRNFIGGEGLLVLHN
	S2	N2	CVSGETPVYLADGKTIKIKDLYSSERKKEDNIVEAGSGEEIIHLKDPIQIYSYVDGTIVRSRSRLLYKGKSSYLVRIETIGGRSVSVTPVHKLFVLTEKGIEEVMASNLKVGDMIAAVAESESEARDCGMSEECV
		C2	MEAEVYTSLEATFDRVKSIAYEKGDFDVYDLSVPEYGRNFIGGEGLLVLHN
	S3	N3	CVSGETPVYLADGKTIKIKDLYSSERKKEDNIVEAGSGEEIIHLKDPIQIYSYVDGTIVRSRSRLLYKGKSSYLVRIETIGGRSVSVTPVHKLFVLTEKGIEEVMASNLKVGDMIAAVAESESEARDCGMSEECVMEAEVYTSLEATFDRVKSIAYEKGDFDVYDLSVPE
		C3	YGRNFIGGEGLLVLHN
PhoRadA	S1	N1	CFARDTEVYYEND
		C1	TVPHMESIEEMYSKYASMNGELPFDNGYAVPLDNVFVYTLDIASGEIKKTRASYIYREKVEKLIEIKLSSGYSLKVTPSHPVLLFRDGLQWVPAAEVKPGDVVVGVREEVLRRRIISKGELEFHEVSSVRIIDYNNWVYDLVIPETHNFIAPNGLVLHN
	S2	N2	CFARDTEVYYENDTVPHMESIEEMYSKYASMNGELPFDNGYAVPLDNVFVYTLDIASGEIKKTRASYIYREKVEKLIEIKLSSGYSLKVTPSHPVLLFRDGLQWVPAAEVKPGDVVVGVREEVLRR
		C2	RIISKGELEFHEVSSVRIIDYNNWVYDLVIPETHNFIAPNGLVLHN
	S3	N3	CFARDTEVYYENDTVPHMESIEEMYSKYASMNGELPFDNGYAVPLDNVFVYTLDIASGEIKKTRASYIYREKVEKLIEIKLSSGYSLKVTPSHPVLLFRDGLQWVPAAEVKPGDVVVGVREEVLRRRIISKGELEFHEVSSVRIIDYNNWVYDLVIPE
		C3	THNFIAPNGLVLHN
Ama-Ter	S1*	N1	NIAHSTPVLTANR
		C1	GWVTHGDLVPGDQVFHPSGKPVDVLALSDEAVDDYVVTFTNGEKIRCHANHEWTVYSRADKQEKTVETKWFLENTNRGTPRSLTAGNRFQFQVPKTNALE**GSAGSAAGSG**ERRVSIEKVEYLPNGEKGHCIQVDSPDGLYLVGKKLVATHN
	S2	N2	NIAHSTPVLTANRGWVTHGDLVPGDQVFHPSGKPVDVLALSDEAVDDYVVTFTNGEKIRCHANHEWTVYSRADKQEKTVETKWFLENTNRGTPRSLTAGNRFQFQVPKTNALE
		C2	ERRVSIEKVEYLPNGEKGHCIQVDSPDGLYLVGKKLVATHN
	S3*	N3	NIAHSTPVLTANRGWVTHGDLVPGDQVFHPSGKPVDVLALSDEAVDDYVVTFTNGEKIRCHANHEWTVYSRADKQEKTVETKWFLENTNRGTPRSLTAGNRFQFQVPKTNALE**GSAGSAAGSG**ERRVSIEKVEYLPNGEKGHCIQVD
		C3	SPDGLYLVGKKLVATHN
Cth-Ter	S1*	N1	QLALDTPIPTPD
		C1	GWTTMGEIKAGDKVIDEKGRPCNVVAISEIDDTEQAYKINFRDGTSIVAGERHLWKVQVTNNGRREKLLTTGEMYQKQFKTKSKENRALFRIPIADAFI**GSAGSAAGSG**SHFHYIKSIEKTGKTKMRCIQVDSPSRLYLAGKSMIPTHN
	S2	N2	QLALDTPIPTPDGWTTMGEIKAGDKVIDEKGRPCNVVAISEIDDTEQAYKINFRDGTSIVAGERHLWKVQVTNNGRREKLLTTGEMYQKQFKTKSKENRALFRIPIADAFI
		C2	SHFHYIKSIEKTGKTKMRCIQVDSPSRLYLAGKSMIPTHN
	S3*	N3	QLALDTPIPTPDGWTTMGEIKAGDKVIDEKGRPCNVVAISEIDDTEQAYKINFRDGTSIVAGERHLWKVQVTNNGRREKLLTTGEMYQKQFKTKSKENRALFRIPIADAFI**GSAGSAAGSG**SHFHYIKSIEKTGKTKMRCIQVD
		C3	SPSRLYLAGKSMIPTHN
LLP-Pol	S1*	N1	CFSGDTEILTPY
		C1	GWVKFEDYDESMMCAQYDEVTGKISFTYPNETIHLKDQDIWVYEDTNTSIHATGNHDILIQKPNGDIAKEKFSNLQLLQKGDKHRFINAGYVDSAEEVDTLMQRLV**GSAGSAAGSG**SFPGKAFPTGPSYKGDVYCVNVPTHNIVIRHNDKVSIQGN
	S2	N2	CFSGDTEILTPYGWVKFEDYDESMMCAQYDEVTGKISFTYPNETIHLKDQDIWVYEDTNTSIHATGNHDILIQKPNGDIAKEKFSNLQLLQKGDKHRFINAGYVDSAEEVDTLMQRLV
		C2	SFPGKAFPTGPSYKGDVYCVNVPTHNIVIRHNDKVSIQGN
	S3*	N3	CFSGDTEILTPYGWVKFEDYDESMMCAQYDEVTGKISFTYPNETIHLKDQDIWVYEDTNTSIHATGNHDILIQKPNGDIAKEKFSNLQLLQKGDKHRFINAGYVDSAEEVDTLMQRLV**GSAGSAAGSG**SFPGKAFPTGPSYKGDVYCVNVP
		C3	THNIVIRHNDKVSIQGN

	S1*	N1	CHAYGHDIMMSD
		C1	GTKKQVQDIAVGDKVMGPDGNPRKVIRLVKGQDEMFRVTPTKGESFVVNGGHILSLYQTPRRAGQTPGYTEISVNEYIRSSSTFKHRSKLQRFGFD**GSAGSAAGSG**RVDVTGFKVEPIGVGDYYGFTVDGDHLYLDGDFVRHHN
LP-Hel	S2	N2	CHAYGHDIMMSDGTKKQVQDIAVGDKVMGPDGNPRKVIRLVKGQDEMFRVTPTKGESFVVNGGHILSLYQTPRRAGQTPGYTEISVNEYIRSSSTFKHRSKLQRFGFD
		C2	RVDVTGFKVEPIGVGDYYGFTVDGDHLYLDGDFVRHHN
	S3*	N3	CHAYGHDIMMSDGTKKQVQDIAVGDKVMGPDGNPRKVIRLVKGQDEMFRVTPTKGESFVVNGGHILSLYQTPRRAGQTPGYTEISVNEYIRSSSTFKHRSKLQRFGFD**GSAGSAAGSG**RVDVTGFKVEPIGVGDYYGFTVDG
		C3	DHLYLDGDFVRHHN
	S1*	N1	PLALNTEVPTPS
		C1	GWTTVGDLSVGDYVLGSDGQPHRVQRETPVLEGLATYVVRFDDGTEITASASHGWTTQRLTGHGDSYETVTVTTEELAQTVTNSKGRKRHRIPVVGME**GSAGSAAGSG**SQHRWVESVTPVESVPVKCIGIDTEDHLFQVSRSRILTHN
MP-B-DnaB	S2	N2	PLALNTEVPTPSGWTTVGDLSVGDYVLGSDGQPHRVQRETPVLEGLATYVVRFDDGTEITASASHGWTTQRLTGHGDSYETVTVTTEELAQTVTNSKGRKRHRIPVVGME
		C2	SQHRWVESVTPVESVPVKCIGIDTEDHLFQVSRSRILTHN
	S3*	N3	PLALNTEVPTPSGWTTVGDLSVGDYVLGSDGQPHRVQRETPVLEGLATYVVRFDDGTEITASASHGWTTQRLTGHGDSYETVTVTTEELAQTVTNSKGRKRHRIPVVGME**GSAGSAAGSG**SQHRWVESVTPVESVPVKCIGIDTE
		C3	DHLFQVSRSRILTHN
	S1*	N1	ALDVETPILTGN
		C1	GWKKMGDIQVGDYVHAADGTLARVSYVSERHWRDCFSVQFADGAELVASDHHLWAVNDRLKGERVIDTAELYRTQTYGARGDRRYTVTVPEALD**GSAGSAAGSG**ARTNTITSVTPVPTVETVCIQIDHPSHVFLAGKSLTPTHN
MP-M-DnaB	S2	N2	ALDVETPILTGNGWKKMGDIQVGDYVHAADGTLARVSYVSERHWRDCFSVQFADGAELVASDHHLWAVNDRLKGERVIDTAELYRTQTYGARGDRRYTVTVPEALD
		C2	ARTNTITSVTPVPTVETVCIQIDHPSHVFLAGKSLTPTHN
	S3*	N3	ALDVETPILTGNGWKKMGDIQVGDYVHAADGTLARVSYVSERHWRDCFSVQFADGAELVASDHHLWAVNDRLKGERVIDTAELYRTQTYGARGDRRYTVTVPEALD**GSAGSAAGSG**ARTNTITSVTPVPTVETVCIQIDHP
		C3	SHVFLAGKSLTPTHN
	S1*	N1	CLHRHTQVLTDG
		C1	GFKDIMAVTSTDKVWSGEKWVNTKGAHLMGWKPVINVDGVLMTEDHKILTHSWKQAKQLVSNKYMMDRALEIGMDAWLSCASYQNDKAKDNYSSNVIVERCLG**GSAGSAAGSG**NLNGKLTYCEPVYDLIDVEDGNRFLIASDSGFLVAHN
SaP-dpol	S2	N2	CLHRHTQVLTDGGFKDIMAVTSTDKVWSGEKWVNTKGAHLMGWKPVINVDGVLMTEDHKILTHSWKQAKQLVSNKYMMDRALEIGMDAWLSCASYQNDKAKDNYSSNVIVERCLG
		C2	NLNGKLTYCEPVYDLIDVEDGNRFLIASDSGFLVAHN
	S3*	N3	CLHRHTQVLTDGGFKDIMAVTSTDKVWSGEKWVNTKGAHLMGWKPVINVDGVLMTEDHKILTHSWKQAKQLVSNKYMMDRALEIGMDAWLSCASYQNDKAKDNYSSNVIVERCLG**GSAGSAAGSG**NLNGKLTYCEPVYDLIDVED
		C3	GNRFLIASDSGFLVAHN
	S1*	N1	CLTGDAKIDVLI
		C1	DNIPISQISLEEVVNLFNEGKEIYVLSYNIDTKEVEYKEISDAGLISESAEVLEIIDEETGQKIVCTPDHKVYTLNRGYVSAKDLKEDDELVFS**GSAGSAAGSG**GLKIIKRESKEPVFDITVKDNSNFFANNILVHN
NrdA-2	S2	N2	CLTGDAKIDVLIDNIPISQISLEEVVNLFNEGKEIYVLSYNIDTKEVEYKEISDAGLISESAEVLEIIDEETGQKIVCTPDHKVYTLNRGYVSAKDLKEDDELVFS
		C2	GLKIIKRESKEPVFDITVKDNSNFFANNILVHN
	S3*	N3	CLTGDAKIDVLIDNIPISQISLEEVVNLFNEGKEIYVLSYNIDTKEVEYKEISDAGLISESAEVLEIIDEETGQKIVCTPDHKVYTLNRGYVSAKDLKEDDELVFS**GSAGSAAGSG**GLKIIKRESKEPVFDITVKD
		C3	NSNFFANNILVHN
	S1*	N1	ALAYDEPIYLSD
		C1	GNIINIGEFVDKFFKKYKNSIKKEDNGFGWIDIGNENIYIKSFNKLSLIIEDKRILRVWRKKYSGKLIKITTKNRREITLTHDHPVYISKTGEVLEINAEMVKVGDYIYIPKNNT**GSAGSAAGSG**INLDEVIKVETVDYNGHIYDLTVEDNHTYIAGKNEGFAVSN
Mja-KlbA	S2	N2	ALAYDEPIYLSDGNIINIGEFVDKFFKKYKNSIKKEDNGFGWIDIGNENIYIKSFNKLSLIIEDKRILRVWRKKYSGKLIKITTKNRREITLTHDHPVYISKTGEVLEINAEMVKVGDYIYIPKNNT
		C2	INLDEVIKVETVDYNGHIYDLTVEDNHTYIAGKNEGFAVSN
	S3*	N3	ALAYDEPIYLSDGNIINIGEFVDKFFKKYKNSIKKEDNGFGWIDIGNENIYIKSFNKLSLIIEDKRILRVWRKKYSGKLIKITTKNRREITLTHDHPVYISKTGEVLEINAEMVKVGDYIYIPKNNT**GSAGSAAGSG**INLDEVIKVETVDYNGHIYDLTVED
		C3	NHTYIAGKNEGFAVSN

[a] The flexible linker sequence is in bold.

Supplementary Table 5 | List of primers used in this study

Primer name	Sequence
(P001)_pSB3T5_fwd	TAATAATACTAGTAGCGGCCGCTGCAG
(P002)_pSB3T5_rev	CTCTAGAAGCGGCCGCGAATTC
(P003)_P(BAD)-RBS32_fwd	ATTCGCGGCCGCTTCTAGAGTTATGACAACTTGACGGCTAC
(P008)_js-Gp41-1-N_rev	TGCAGCGGCCGCTACTAGTATTATTATTCTTTAACATACAGGCACATAC
(P009)_pSEVA221_fwd	TACTAGTAGCGGCCGCTGCAGCTTGGACTCCTGTTGATAGATCCAGTAATGAC
(P010)_pSEVA221_rev	GGCCTAGGCGGCCTCCTGTG
(P012)_Gp41-1-C-js_fwd	TACTAGAGTCACACAGGAAAGTACTAGATGATGCTGAAAAAAATCCTG
(P089)_mC159(SGY).R	GTATCCTGAGTCCTCGGGGTACATCC
(P090)_gp41-1N.F	ACCCCGAGGACTCAGGATACTGTCTGGATCTGAAAACCCAGG
(P091)_(SSS)mC160.F	TCTAGCAGTGGCGCCCTGAAGGGCGAG
(P092)_gp41-1C.R	CTCGCCCTTCAGGGCGCCACTGCTAGAATTATGGGTCAGAATATCATTGG
(P133)_mC.159.SP1.F	GTGGATGCTAGCGATCTGGGCGCCCTGAAGGGCGAGATCAAGCAG
(P134)_mC.159.SP1.R	CAGATCGCTAGCATCCACGTCCTCGGGGTACATCCGCTCGGAGG
(P135)_mC.159.SP2.F	GGCGGAAAATGCTGGAATGGCGCCCTGAAGGGCGAGATCAAGCAG
(P136)_mC.159.SP2.R	ATTCCAGCATTTTCCGCCGTCCTCGGGGTACATCCGCTCGGAGG
(P137)_mC.159.SP4.F	TTGAACAGGAGTGCGGTTGGCGCCCTGAAGGGCGAGATCAAGCAG
(P138)_mC.159.SP4.R	AACCGCACTCCTGTTCAAGTCCTCGGGGTACATCCGCTCGGAGG
(P139)_mC.159.SP5.F	AATCCGTGTTCAGAGATCGGCGCCCTGAAGGGCGAGATCAAGCAG
(P140)_mC.159.SP5.R	GATCTCTGAACACGGATTGTCCTCGGGGTACATCCGCTCGGAGG
(P141)_mC.159.SP6.F	GGTGGAGGTTCGATATGCGGCGCCCTGAAGGGCGAGATCAAGCAG
(P142)_mC.159.SP6.R	GCATATCGAACCTCCACCGTCCTCGGGGTACATCCGCTCGGAGG
(P143)_mC.159.SP7.F	ATTGACGAATGTCACACTGGCGCCCTGAAGGGCGAGATCAAGCAG
(P144)_mC.159.SP7.R	AGTGTGACATTCGTCAATGTCCTCGGGGTACATCCGCTCGGAGG
(P145)_mC.159.SP8.F	GCAGGTGGTAGTGCAAAAGGCGCCCTGAAGGGCGAGATCAAGCAG
(P146)_mC.159.SP8.R	TTTTGCACTACCACCTGCGTCCTCGGGGTACATCCGCTCGGAGG
(P147)_mC.159.SP9.F	ATTGGTTGTTCGTTCGACGGCGCCCTGAAGGGCGAGATCAAGCAG
(P148)_mC.159.SP9.R	GTCGAACGAACAACCAATGTCCTCGGGGTACATCCGCTCGGAGG
(P149)_mC.159.SP10.F	GGGAGCAAAACTGTCATTGGCGCCCTGAAGGGCGAGATCAAGCAG
(P150)_mC.159.SP10.R	AATGACAGTTTTGCTCCCGTCCTCGGGGTACATCCGCTCGGAGG
(P151)_mC.159.SP11.F	AGTGGTAAAACGCAATTAGGCGCCCTGAAGGGCGAGATCAAGCAG
(P152)_mC.159.SP11.R	TAATTGCGTTTTACCACTGTCCTCGGGGTACATCCGCTCGGAGG
(P179II)_P(BAD).R	CTCTAGTAATGGAGAAACAGTAGAGAGTTG
(P181)_ECF16.R	CCGCTACTAGTATTATTAACGATCTTCATCATGACCTTTACC
(P210)_ECF16.N114.R	CCGCTACTAGTATTATTAATCATCATCCAGCAGATCATGACGTGC
(P211)_ECF17.N97.R	CCGCTACTAGTATTATTAACCGCTCTGATCGGTGCTACCAACAACATTACG
(P212)_ECF20.N107.R	CCGCTACTAGTATTATTAATCCAGTGCTTCACAGGCATGTTCG
(P220)_P(rhaB)-30.R	CTAGTATTTCTCCTCTTTAATCTCTCTAGTATACGACCAGTCTAAAAAGCGCCTCAATTCG
(P226)_ECF16.C115.F	AAAGAGGAGAAATACTAGATGAGCGAACTGTTTGCAGCAAGTGATGAACAGC
(P227)_ECF17.C98.F	AAAGAGGAGAAATACTAGATGACACCGGAACAGAGCACACCGGATGAAGTTAATGC
(P229)_CF20.C108.F	AAAGAGGAGAAATACTAGATGACCCGTCCGGCACCGGATGAACAGCTGGAAGC
(P241)_M86.N.F	CGGATGTACCCCGAGGACGTGGATGCTTGCATCTCGGGAGATAGTTTGATCAGCTTGG
(P241)_M86.N.F	CGGATGTACCCCGAGGACGTGGATGCTTGCATCTCGGGAGATAGTTTGATCAGCTTGG
(P242)_M86.N2.R	CCGCTACTAGTATTATTATTCTAGTTTACGGGGTAGAGCAATATGC
(P242)_M86.N2.R	CCGCTACTAGTATTATTATTCTAGTTTACGGGGTAGAGCAATATGC
(P243)_NpuSsp.N.F	CGGATGTACCCCGAGGACGGCGGAAAATGCCTGAGCTATGAAACCGAAATTCTGACC
(P244)_NpuSsp.N2.R	CCGCTACTAGTATTATTAGTTCGGCAGGTTATCCACGCGCATCAGATCC
(P245)_gp41-8.N.F	CGGATGTACCCCGAGGACTTGAACAGGTGTCTGAGCCTGGATACCATGGTTGTTACC
(P246)_gp41-8.N2.R	CCGCTACTAGTATTATTATTTTGCACGGCTACGCAGAAAATCG
(P247)_NrdJ-1.N.F	CGGATGTACCCCGAGGACAATCCGTGTTGTCTGGTTGGTAGCAGCGAAATCATTACC
(P248)_NrdJ-1.N2.R	CCGCTACTAGTATTATTAAATTGCAACCACCAGTTCATCATCATCG
(P249)_IMPDH-1.N.F	CGGATGTACCCCGAGGACGGTGGAGGTTGTTTTGTTCCGGGTACACTGGTGAATACC
(P250)_IMPDH-1.N2.R	CCGCTACTAGTATTATTATTCCAGCTCGATCAGCAGATGTTTTTTCATATCC
(P251)_SspDnaX.N.F	CGGATGTACCCCGAGGACATTGACGAATGCTTAACGGGGGACTCACAAGTTTTGACC
(P252)_SspDnaX.N2.R	CCGCTACTAGTATTATTACGCAGGGGATAGTATCTTCATTCC
(P253)_SspGyrB.N.F	CGGATGTACCCCGAGGACGCAGGTGGTTGTTTTTCTGGAGATACATTAGTCG
(P254)_SspGyrB.N2.R	CCGCTACTAGTATTATTACGCATGACCAGAATCTTCCGTAGTCG
(P255)_TerThyX.N.F	CGGATGTACCCCGAGGACATTGGTTGTTGTTTGTCGGGCAATACAAAAGTTAGATTTAGG
(P256)_TerThyX.N2.R	CCGCTACTAGTATTATTACGCTAATTGCACTTCACATAATTGAGTGC
(P257)_TvoVMA.N.F	CGGATGTACCCCGAGGACGGGAGCAAATGCGTATCAGGTGAAACACCAGTTTACC
(P259)_PhoRadA.N.F	CGGATGTACCCCGAGGACAGTGGTAAATGCTTTGCTAGGGATACCGAAGTTTATTATGAAAACG
(P260)_PhoRadA.N2.R	CCGCTACTAGTATTATTATCTTCTCAGTACCTCTTCCCTAACACCAACAACAACATCTCC
(P262)_M86.C2.F	AGTCACACAGGAAAGTACTAGATGAGCTCCTCTTTACAATTGGCACCAGAAATAG
(P262)_M86.C2.F	AGTCACACAGGAAAGTACTAGATGAGCTCCTCTTTACAATTGGCACCAGAAATAG
(P263)_M86.C.R	CTCGCCCTTCAGGGCGCCCAGATCGCTGTTATGTACAATGATGTCATTGGCGACAAAGTTACG
(P263)_M86.C.R	CTCGCCCTTCAGGGCGCCCAGATCGCTGTTATGTACAATGATGTCATTGGCGACAAAGTTACG
(P265)_NpuSsp.C.R	CTCGCCCTTCAGGGCGCCATTCCAGCAGTTCGCCGCAATCGCGCCGTTCGCCAGC
(P267)_gp41-8.C.R	CTCGCCCTTCAGGGCGCCAACCGCACTATTATGGGTCAGAATACCATTGGCAAAAAACAGACG
(P268)_NrdJ-1.C2.F	AGTCACACAGGAAAGTACTAGATGGAAGCCAAAACCTATATCGGCAAACTG
(P269)_NrdJ-1.C.R	CTCGCCCTTCAGGGCGCCGATCTCTGAATTATGCACCAGAATATCATTGGCGAAAAAGTTATG
(P271)_IMPDH-1.C.R	CTCGCCCTTCAGGGCGCCGCATATCGAATTATGAACAACGGTGCCACGAACGTTATAGC
(P273)_SspDnaX.C.R	CTCGCCCTTCAGGGCGCCAGTGTGACAGTTATGGACTAGTAAGCCATTGGCAAC
(P274)_SspGyrB.C2.F	AGTCACACAGGAAAGTACTAGATGGAAGCAGTATTAAATTACAATCACAGAATTG
(P275)_SspGyrB.C.R	CTCGCCCTTCAGGGCGCCTTTTGCACTGTTATGGACAAACACTCCGCTTGCCAAAGC
(P277)_TerThyX.C.R	CTCGCCCTTCAGGGCGCCGTCGAACGAATTATGAACAACTAAACCATTGGCTATAAAATTATGTTC
(P279)_TvoVMA.C.R	CTCGCCCTTCAGGGCGCCAATGACAGTGTTGTGAAGTACGAGAAGTCCTTCTCC
(P281)_PhoRadA.C.R	CTCGCCCTTCAGGGCGCCTAATTGCGTATTATGGAGAACAAGTCCATTGGGAGCTATGAAG
(P293)_33-ECF20.F	GTTTCTCCATTACTAGAGTCACACAGGACTACTAGATGAATGAAACCGATCCTG
(P298)_30.mCstar.F	GTTTCTCCATTACTAGAGATTAAAGAGGAGAAATACTAGATGGTGAGCAAGGGCGAAGAAGACAACATGG

(P306)_30-NpuSsp.C2.F	GTTTCTCCATTACTAGAGATTAAAGAGGAGAAATACTAGATGGTGAAAGTGATTGG
(P307)_30-gp41-1.C2.F	GTTTCTCCATTACTAGAGATTAAAGAGGAGAAATACTAGATGCTGAAAAAAATCCTGAAAATCG
(P308)_30-gp41-8.C2.F	GTTTCTCCATTACTAGAGATTAAAGAGGAGAAATACTAGATGTGTGAAATCTTTGAAAACG
(P309)_30-NrdJ-1.C2.F	GTTTCTCCATTACTAGAGATTAAAGAGGAGAAATACTAGATGGAAGCCAAAACCTATATCGGCAAAC
(P315)_30-PhoRadA.C2.F	GTTTCTCCATTACTAGAGATTAAAGAGGAGAAATACTAGATGAGAATAATATCCAAAGGAGAACTTG
(P329)_30-ST-IMPDH-1.C2.F	GAGGAGAAATACTAGATGACAGATGTAACGATTAAAAAATTCAAACTGAAAGAGATCACCAGC
(P330)_30-ST-SspDnaX.C2.F	GAGGAGAAATACTAGATGACAGATGTAACGATTAAACCGCAATGGCATACAAATTTCGAGG
(P332)_30-ST-TerThyX.C2.F	GAGGAGAAATACTAGATGACAGATGTAACGATTAAAGGTGTTTTTGTAGAGATAGAATCTATTG
(P337)_M86.N1.R	CCGCTACTAGTATTATTATGTGCTCGCCAAGCTGATCAAACTATCTCC
(P338)_NpuSsp.N1.R	CCGCTACTAGTATTATTAATATTCCACGGTCAGAATTTCGGTTTCATAGC
(P339)_gp41-1.N1.R	CCGCTACTAGTATTATTACTGCGGTGTCTGAACCTGGGTTTTCAGATC
(P340)_gp41-8.N1.R	CCGCTACTAGTATTATTAATTGGTAACAACCATGGTATCCAGGCTCAGAC
(P341)_NrdJ-1.N1.R	CCGCTACTAGTATTATTAATAATTACGGGTAATGATTTCGCTGCTACC
(P342)_IMPDH-1.N1.R	CCGCTACTAGTATTATTAATTTTCGGTATTCACCAGTGTACCCGGAAC
(P343)_SspDnaX.N1.R	CCGCTACTAGTATTATTAATTGCGGGTCAAAACTTGTGAGTCCCCCGTTAAG
(P344)_SspGyrB.N1.R	CCGCTACTAGTATTATTAATCAGTTAAAGCGACTAATGTATCTCCAG
(P345)_TerThyX.N1.R	CCGCTACTAGTATTATTACGAATACCTAAATCTAACTTTTGTATTGC
(P346)_TvoVMA.N1.R	CCGCTACTAGTATTATTAATCCGCAAGGTAAACTGGTGTTTCACCTG
(P347)_PhoRadA.N1.R	CCGCTACTAGTATTATTAATCGTTTTCATAATAAACTTCGGTATCCCTAG
(P348)_M86.N3.R	CCGCTACTAGTATTATTATCCTGGCACAGTCAAATCAAAAACCTCTTCG
(P349)_NpuSsp.N3.R	CCGCTACTAGTATTATTACTGGCGCAGGCCAATATCAAAAATGCGCTGC
(P350)_gp41-1.N3.R	CCGCTACTAGTATTATTAACCGCTAACTTCAATATCAATCAGTTCG
(P351)_gp41-8.N3.R	CCGCTACTAGTATTATTAATTGGTCACGTTAATATCGATGGTTTCTTCC
(P352)_NrdJ-1.N3.R	CCGCTACTAGTATTATTAGCTGGTCTGAATATCATAGGTATCCTCGTTGC
(P353)_IMPDH-1.N3.R	CCGCTACTAGTATTATTACTGATTCACGGTCAGATCATGAACTTTGC
(P354)_SspDnaX.N3.R	CCGCTACTAGTATTATTAATCTTCCACCTCCAGGTCATAAACTTTTTCC
(P355)_SspGyrB.N3.R	CCGCTACTAGTATTATTAGTGGGGAACCTCAATATCATAAACATCGATTGTTTC
(P356)_TerThyX.N3.R	CCGCTACTAGTATTATTAGTGTTCGACTTCAAGATCATAAGTAATTTCTTTGC
(P357)_TvoVMA.N3.R	CCGCTACTAGTATTATTATTCTGGTACGGAAAGATCGTATACATCAAAATCAC
(P358)_PhoRadA.N3.R	CCGCTACTAGTATTATTATTCCGGAATTACAAGGTCGTAGACC
(P370)_M86.C1.F	GAGGAGAAATACTAGATGGGGAAAAGAGTTCCTATTAAGGATTTGTTAGGCGAAAAAGATTTTGAAATATGG
(P371)_NpuSsp.C1.F	GAGGAGAAATACTAGATGGGCAGCCTGCCGATTGGCAAAATTGTGGAAAAAACGCATTG
(P372)_gp41-1.C1.F	GAGGAGAAATACTAGATGGGTATGAAAGAAATTTCAAATATTCAGGTGGGTGATCTGGTTCTGAGC
(P373)_gp41-8.C1.F	GAGGAGAAATACTAGATGGGTAAAGCCATTGAAATTCGTGATGTGAAAGTTGGTGATTGGCTGG
(P374)_NrdJ-1.C1.F	GAGGAGAAATACTAGATGGGTAAAACCACCATCAAAGAAGTGGTCGAGATCTTCGATAACGAC
(P375)_IMPDH-1.C1.F	GAGGAGAAATACTAGATGGGTCTGAAAAAAATCGAAGAAATCAAAGTGGGCGACAAAGTGTTTAGC
(P376)_SspDnaX.C1.F	GAGGAGAAATACTAGATGGGCTTAATGTCCATTGACAATCCCCAAATAAAAGGGCGAGAAG
(P377)_SspGyrB.C1.F	GAGGAGAAATACTAGATGGGTCGTAGCGTTAGCTTTGAGCAATTGGTTGAAGAAGAAAAACAAGG
(P378)_TerThyX.C1.F	GAGGAGAAATACTAGATGTCTTCATCCCAAGAAGCGAAATATTATGAGGAAACAATTGAAAAATTAGCTAACTTATGG
(P379)_TvoVMA.C1.F	GAGGAGAAATACTAGATGGGCAAGACAATAAAAATAAAGGATCTATACAGTTCTGAGAGAAAAAAAGAAG
(P380)_PhoRadA.C1.F	GAGGAGAAATACTAGATGACGGTACCACACATGGAATCAATTGAGGAGATGTATAGTAAATACG
(P381)_M86.C3.F	GAGGAGAAATACTAGATGCTACGTAACTTTGTCGCCAATGACATCATTGTACATAAC
(P382)_NpuSsp.C3.F	GAGGAGAAATACTAGATGGATCATAACTTTCTGCTGGCGAACGGCGCGATTGC
(P383)_gp41-1.C3.F	GAGGAGAAATACTAGATGAACCACCTGTTCTATGCCAATGATATTCTGACC
(P384)_gp41-8.C3.F	GAGGAGAAATACTAGATGGATCGTCTGTTTTTTGCCAATGGTATTCTGACC
(P385)_NrdJ-1.C3.F	GAGGAGAAATACTAGATGACCCATAACTTTTTCGCCAATGATATTCTGGTGC
(P386)_IMPDH-1.C3.F	GAGGAGAAATACTAGATGGATCATAGCTATAACGTTCGTGGCACCGTTG
(P387)_SspDnaX.C3.F	GAGGAGAAATACTAGATGAATCACAATTTTGTTGCCAATGGCTTACTAGTCC
(P388)_SspGyrB.C3.F	GAGGAGAAATACTAGATGACCCACAATTTTGCTTTGGCAAGCGGAGTG
(P389)_TerThyX.C3.F	GAGGAGAAATACTAGATGCCAGAACATAATTTTATAGCCAATGGTTTAGTTG
(P390)_TvoVMA.C3.F	GAGGAGAAATACTAGATGTACGGCAGGAACTTTATAGGCGGAGAAGG
(P391)_PhoRadA.C3.F	GAGGAGAAATACTAGATGACCCACAACTTCATAGCTCCCAATGGACTTGTTC
(P403)_mC.F	GGCGCCCTGAAGGGCGAGATCAAGCAGAGGCTGAAGC
(P404)_mC.R	GTCCTCGGGGTACATCCGCTCGGAGGAGGCCTCCCAGC
(P405)_Int.F	GCCTCCTCCGAGCGGATGTACCCCGAGG
(P406)_Int.R	CCTCTGCTTGATCTCGCCCTTCAGG
(P407)_mC.159.SP12.F	GTGAATCAGTCCGGGAGGGGCGCCCTGAAGGGCGAGATCAAGCAG
(P408)_mC.159.SP12.R	CCTCCCGGACTGATTCACGTCCTCGGGGTACATCCGCTCGGAGG
(P409)_mC.159.SP13.F	GATAAATTCTCAACCAAAGGCGCCCTGAAGGGCGAGATCAAGCAG
(P410)_mC.159.SP13.R	TTTGGTTGAGAATTTATCGTCCTCGGGGTACATCCGCTCGGAGG
(P411)_mC.159.SP14.F	GGCAGTAAGTGCTCAAACGGCGCCCTGAAGGGCGAGATCAAGCAG
(P412)_mC.159.SP14.R	GTTTGAGCACTTACTGCCGTCCTCGGGGTACATCCGCTCGGAGG
(P413)_mC.159.SP15.F	TCGAATCTCTGCTCAGAGGGCGCCCTGAAGGGCGAGATCAAGCAG
(P414)_mC.159.SP15.R	CTCTGAGCAGAGATTCGAGTCCTCGGGGTACATCCGCTCGGAGG
(P415)_mC.159.SP16.F	AAGCATCACTCAGTCCTTGGCGCCCTGAAGGGCGAGATCAAGCAG
(P416)_mC.159.SP16.R	AAGGACTGAGTGATGCTTGTCCTCGGGGTACATCCGCTCGGAGG
(P417)_mC.159.SP17.F	CATGGTAAATCCGAGCTGGGCGCCCTGAAGGGCGAGATCAAGCAG
(P418)_mC.159.SP17.R	CAGCTCGGATTTACCATGGTCCTCGGGGTACATCCGCTCGGAGG
(P419)_mC.159.SP18.F	GCTAGCTGTTGTCGTCTCGGCGCCCTGAAGGGCGAGATCAAGCAG
(P420)_mC.159.SP18.R	GAGACGACAACAGCTAGCGTCCTCGGGGTACATCCGCTCGGAGG
(P421)_mC.159.SP19.F	AATGGGAAAACTACCCTCGGCGCCCTGAAGGGCGAGATCAAGCAG
(P422)_mC.159.SP19.R	GAGGGTAGTTTTCCCATTGTCCTCGGGGTACATCCGCTCGGAGG
(P423)_mC.159.SP20.F	AATGGGAAGTCGGAGTTAGGCGCCCTGAAGGGCGAGATCAAGCAG
(P424)_mC.159.SP20.R	TAACTCCGACTTCCCATTGTCCTCGGGGTACATCCGCTCGGAGG
(P425)_mC.159.SP21.F	CACGGTAAAACCGAGGTCGGCGCCCTGAAGGGCGAGATCAAGCAG
(P426)_mC.159.SP21.R	GACCTCGGTTTTACCGTGGTCCTCGGGGTACATCCGCTCGGAGG
(P427)_mC.159.SP22.F	GCCAAGGCATGCAATTTCGGCGCCCTGAAGGGCGAGATCAAGCAG
(P428)_mC.159.SP22.R	GAAATTGCATGCCTTGGCGTCCTCGGGGTACATCCGCTCGGAGG
(P429)_mC.159.SP23.F	GCCACTGGGTGCGGGAAGGGCGCCCTGAAGGGCGAGATCAAGCAG
(P430)_mC.159.SP23.R	CTTCCCGCACCCAGTGGCGTCCTCGGGGTACATCCGCTCGGAGG
(P431)_mC.159.SP24.F	CAGGATCAGACCAAAAATGGCGCCCTGAAGGGCGAGATCAAGCAG

(P432)_mC.159.SP24.R	ATTTTTGGTCTGATCCTGGTCCTCGGGGTACATCCGCTCGGAGG
(P433)_mC.159.SP25.F	AACCATGATTCACGGGCGGGCGCCCTGAAGGGCGAGATCAAGCAG
(P434)_mC.159.SP25.R	CGCCCGTGAATCATGGTTGTCCTCGGGGTACATCCGCTCGGAGG
(P435)_mC.159.SP26.F	GAGCTGAAGACACAAAATGGCGCCCTGAAGGGCGAGATCAAGCAG
(P436)_mC.159.SP26.R	ATTTTGTGTCTTCAGCTCGTCCTCGGGGTACATCCGCTCGGAGG
(P437)_mC.159.SP27.F	AACGGGAAAACAGAATTGGGCGCCCTGAAGGGCGAGATCAAGCAG
(P438)_mC.159.SP27.R	CAATTCTGTTTTCCCGTTGTCCTCGGGGTACATCCGCTCGGAGG
(P439)_mC.159.SP28.F	GCAGGCAAGTCCCTTACTGGCGCCCTGAAGGGCGAGATCAAGCAG
(P440)_mC.159.SP28.R	AGTAAGGGACTTGCCTGCGTCCTCGGGGTACATCCGCTCGGAGG
(P441)_mC.159.SP29.F	GGAAAGGCCTGCGAACTGGGCGCCCTGAAGGGCGAGATCAAGCAG
(P442)_mC.159.SP29.R	CAGTTCGCAGGCCTTTCCGTCCTCGGGGTACATCCGCTCGGAGG
(P443)_mC.159.SP30.F	AGCGGCAAGACGAGTTCTGGCGCCCTGAAGGGCGAGATCAAGCAG
(P444)_mC.159.SP30.R	AGAACTCGTCTTGCCGCTGTCCTCGGGGTACATCCGCTCGGAGG
(P445)_mC.159.SP31.F	TCAAACCTTTGCAATGAGGGCGCCCTGAAGGGCGAGATCAAGCAG
(P446)_mC.159.SP31.R	CTCATTGCAAAGGTTTGAGTCCTCGGGGTACATCCGCTCGGAGG
(P447)_mC.159.SP32.F	GGGGGCGGCACAGGCTTAGGCGCCCTGAAGGGCGAGATCAAGCAG
(P448)_mC.159.SP32.R	TAAGCCTGTGCCGCCCCCGTCCTCGGGGTACATCCGCTCGGAGG
(P449)_mC.159.SP33.F	CACGATGGATGCTCGGGTGGCGCCCTGAAGGGCGAGATCAAGCAG
(P450)_mC.159.SP33.R	ACCCGAGCATCCATCGTGGTCCTCGGGGTACATCCGCTCGGAGG
(P451)_ECF20.SP1.F	GTGGATGCTAGCGATCTGGCACCGGATGAACAGCTGGAAGCAAGC
(P452)_ECF20.SP1.R	CAGATCGCTAGCATCCACTTCACAGGCATGTTCGCTATCAACCGGCAC
(P455)_ECF20.SP3.F	TCAGGATACTCTAGCAGTGCACCGGATGAACAGCTGGAAGCAAGC
(P456)_ECF20.SP3.R	ACTGCTAGAGTATCCTGATTCACAGGCATGTTCGCTATCAACCGGCAC
(P457)_ECF20.SP4.F	TTGAACAGGAGTGCGGTTGCACCGGATGAACAGCTGGAAGCAAGC
(P458)_ECF20.SP4.R	AACCGCACTCCTGTTCAATTCACAGGCATGTTCGCTATCAACCGGCAC
(P459)_ECF20.SP5.F	AATCCGTGTTCAGAGATCGCACCGGATGAACAGCTGGAAGCAAGC
(P460)_ECF20.SP5.R	GATCTCTGAACACGGATTTTCACAGGCATGTTCGCTATCAACCGGCAC
(P465)_ECF20.SP8.F	GCAGGTGGTAGTGCAAAAGCACCGGATGAACAGCTGGAAGCAAGC
(P466)_ECF20.SP8.R	TTTTGCACTACCACCTGCTTCACAGGCATGTTCGCTATCAACCGGCAC
(P815)_ECF20-SP20.F	AATGGGAAGTCGGAGTTAGCACCGGATGAACAGCTGGAAGCAAGC
(P816)_ECF20-SP20.R	TAACTCCGACTTCCCATTTTCACAGGCATGTTCGCTATCAACCGGCAC
(P756)_ECF20-SP33.F	CACGATGGATGCTCGGGTGCACCGGATGAACAGCTGGAAGC
(P757)_ECF20-SP33.R	ACCCGAGCATCCATCGTGTTCACAGGCATGTTCGCTATCAACC
(P483)_33-ECF20.N.R	TTCACAGGCATGTTCGCTATCAACCGGCAC
(P484)_E20N.M86.F	AGCGAACATGCCTGTGAAGTGGATGCTTGCATCTCGGGAGATAGTTTGATCAGC
(P493)_E20C.F	ACCCGTCCGGCACCGGATGAACAGCTGGAAGC
(P494)_P(rhaB)-32.R	CTAGTACTTTCCTGTGTGACTCTAGTATACGACCAGTCTAAAAAGCGCCTCAATTCGCGACC
(P495)_E20C.M86.R	ATCCGGTGCCGGACGGGTCAGATCGCTGTTATGTACAATGATGTCATTGGCGACAAAGTTACG
(P937)_ECF17-SP1.F	GTGGATGCTAGCGATTTACAGAGCACACCGGATGAAGTTAATGCAGCACTGG
(P938)_ECF17-SP1.R	TAAATCGCTAGCATCCACATCGGTGCTACCAACAACATTACGAAAACGTGC
(P941)_ECF17-SP3.F	TCAGGATACTCTAGCAGTCAGAGCACACCGGATGAAGTTAATGCAGCACTGG
(P942)_ECF17-SP3.R	ACTGCTAGAGTATCCTGAATCGGTGCTACCAACAACATTACGAAAACGTGC
(P943)_ECF17-SP4.F	TTGAACAGGAGTGCGGTTCAGAGCACACCGGATGAAGTTAATGCAGCACTGG
(P944)_ECF17-SP4.R	AACCGCACTCCTGTTCAAATCGGTGCTACCAACAACATTACGAAAACGTGC
(P514)_ECF17.SP5.F	AATCCGTGTTCAGAGATCCAGAGCACACCGGATGAAGTTAATGCAGCACTGG
(P515)_ECF17.SP5.R	GATCTCTGAACACGGATTATCGGTGCTACCAACAACATTACGAAAACGTGC
(P532)_ECF17.SP8.F	GCAGGTGGTAGTGCAAAACAGAGCACACCGGATGAAGTTAATGCAGCACTGG
(P533)_ECF17.SP8.R	TTTTGCACTACCACCTGCATCGGTGCTACCAACAACATTACGAAAACGTGC
(P955)_ECF17-SP20.F	AATGGGAAGTCGGAGTTACAGAGCACACCGGATGAAGTTAATGCAGCACTGG
(P956)_ECF17-SP20.R	TAACTCCGACTTCCCATTATCGGTGCTACCAACAACATTACGAAAACGTGC
(P794)_ECF17-SP33.F	CACGATGGATGCTCGGGTCAGAGCACACCGGATGAAGTTAATGC
(P795)_ECF17-SP33.R	ACCCGAGCATCCATCGTGATCGGTGCTACCAACAACATTACGAAAACGTGC
(P52)_mC.R	CCGCTACTAGTATTATTAGTGGTGATGGTGATGATGCTTGTACAGCTCGTCCATGCCGCCGGTGG
(P903)_ECF16-SP1.F	GTGGATGCTAGCGATCTGTTTGCAGCAAGTGATGAACAGCCTGCACAGG
(P904)_ECF16-SP1.R	CAGATCGCTAGCATCCACCAGCAGATCATGACGTGCTTCACGACGTGCATGG
(P907)_ECF16-SP3.F	TCAGGATACTCTAGCAGTTTTGCAGCAAGTGATGAACAGCCTGCACAGG
(P908)_ECF16-SP3.R	ACTGCTAGAGTATCCTGACAGCAGATCATGACGTGCTTCACGACGTGCATGG
(P909)_ECF16-SP4.F	TTGAACAGGAGTGCGGTTTTTGCAGCAAGTGATGAACAGCCTGCACAGG
(P910)_ECF16-SP4.R	AACCGCACTCCTGTTCAACAGCAGATCATGACGTGCTTCACGACGTGCATGG
(P512)_ECF16.SP5.F	AATCCGTGTTCAGAGATCTTTGCAGCAAGTGATGAACAGCCTGCACAGG
(P513)_ECF16.SP5.R	GATCTCTGAACACGGATTCAGCAGATCATGACGTGCTTCACGACGTGCATGG
(P530)_ECF16.SP8.F	GCAGGTGGTAGTGCAAAATTTGCAGCAAGTGATGAACAGCCTGCACAGG
(P531)_ECF16.SP8.R	TTTTGCACTACCACCTGCCAGCAGATCATGACGTGCTTCACGACGTGCATGG
(P921)_ECF16-SP20.F	AATGGGAAGTCGGAGTTATTTGCAGCAAGTGATGAACAGCCTGCACAGG
(P922)_ECF16-SP20.R	TAACTCCGACTTCCCATTCAGCAGATCATGACGTGCTTCACGACGTGCATGG
(P754)_ECF16-SP33.F	CACGATGGATGCTCGGGTTTTGCAGCAAGTGATGAACAGC
(P755)_ECF16-SP33.R	ACCCGAGCATCCATCGTGCAGCAGATCATGACGTGCTTCACG
(P544)_NpuSsp.Ln.F	CGGTTCAGCGGCCGGTTCGGGAGTGAAAGTGATTGGCCGCCGCAGCCTGGGCGTGC
(P545)_NpuSsp.Ln.R	GAACCGGCCGCTGAACCGGCTGATCCGTTCGGCAGGTTATCCACGCGCATCAGATCC
(P546)_TerThyX.Ln.F	GGTTCAGCGGCCGGTTCGGGAGGTGTTTTTGTAGAGATAGAATCTATTGAAAAATTTGGCAAAGAAATTAC
(P547)_TerThyX.Ln.R	CGAACCGGCCGCTGAACCGGCTGATCCCGCTAATTGCACTTCACATAATTGAGTGCTGTTATTAAAATC
(P554)_Ama-Ter.N2.R	CCGCTACTAGTATTATTATTCCAGGGCGTTTGTCTTTGGAACCTGAAACTG
(P555)_Cth-Ter.N2.R	CCGCTACTAGTATTATTAAATGAACGCGTCCGCGATCGGTATACGAAACAGG
(P556)_LLP-Pol.N2.R	CCGCTACTAGTATTATTAAACCAGACGCTGCATGAGAGTGTCTACCTCTTCG
(P557)_LP-Hel.N2.R	CCGCTACTAGTATTATTAATCGAAGCCAAAGCGCTGTAATTTTGAACGGTGC
(P558)_MP-B-DnaB.N2.R	CCGCTACTAGTATTATTATTCCATACCCACTACAGGGATGCGATGACG
(P560)_MP-M-DnaB.N2.R	CCGCTACTAGTATTATTAATCCAAAGCTTCTGGCACCGTAACAGTGTAACG
(P561)_SaP-dpol.N2.R	CCGCTACTAGTATTATTAGCCCAGGCACCGTTCCACAATAACGTTACTACTG
(P563)_NrdA-2.N2.R	CCGCTACTAGTATTATTAGCTAAACACCAGCTCATCGTCTTCTTTGAGATCC
(P564)_Mja-KlbA.N2.R	CCGCTACTAGTATTATTACGTGTTATTTTTCGGGATGTAAATGTAATCACCGACTTTAACC
(P567)_Ama-Ter.C2.F	GAGGAGAAATACTAGATGGAGCGCCGCGTTTCCATAGAGAAAGTAGAATATCTTCC

(P568)_Cth-Ter.C2.F	GAGGAGAAATACTAGATGTCTCACTTCCACTACATAAAGTCTATTGAAAAAACCGGGAAAACC
(P569)_LLP-Pol.C2.F	GAGGAGAAATACTAGATGTCTTTCCCTGGGAAGGCATTCCCTACAGGCCCGAGTTATAAAGG
(P57)_mC.159.SP3.R	GCGCCACTGCTAGAGTATCCTGAGTCCTCGGGGTACATCCGCTCGGAGG
(P570)_LP-Hel.C2.F	GAGGAGAAATACTAGATGCGCGTTGACGTAACGGGGTTTAAAGTTGAGCCCATTGG
(P571)_MP-B-DnaB.C2.F	GAGGAGAAATACTAGATGTCCCAGCATCGGTGGGTTGAATCAGTAACACC
(P572)_MP-C-gp206.C2.F	GAGGAGAAATACTAGATGCTGCGTTTAAACGCCATTGTTGCAGTAGAACCGGTGG
(P574)_SaP-dpol.C2.F	GAGGAGAAATACTAGATGAATCTGAACGGCAAACTGACGTACTGTGAACCAG
(P576)_NrdA-2.C2.F	GAGGAGAAATACTAGATGGGTTTAAAAATTATAAAACGTGAATCAAAGGAACCAGTGTTTGAC
(P577)_Mja-KlbA.C2.F	GAGGAGAAATACTAGATGATTAACCTGGATGAAGTAATCAAAGTTGAAACAGTGGAC
(P58)_mC.159.SP3.F	AGGACTCAGGATACTCTAGCAGTGGCGCCCTGAAGGGCGAGATCAAGCAG
(P581)_TvoVMA.N2.v2.R	CCGCTACTAGTATTATTACACGCATTCCTCGCTCATTCCGCAGTCTCTTGC
(P583)_30-TvoVMA.C2.v2.F	GAGGAGAAATACTAGATGGAAGCAGAAGTTTATACGTCACTTGAAGC
(P586)_Ama-Ter.N1.R	CCGCTACTAGTATTATTAACGGTTCGCAGTCAGCACAGGGGTGCTATGTGCAATATTTTTACC
(P587)_Cth-Ter.N1.R	CCGCTACTAGTATTATTAATCTGGAGTAGGGATAGGTGTATCAAGCGCAAGCTGCTTCC
(P588)_LLP-Pol.N1.R	CCGCTACTAGTATTATTAGTATGGCGTCAAGATTTCGGTATCTCCTGAAAAGCATGC
(P589)_LP-Hel.N1.R	CCGCTACTAGTATTATTAATCGGACATCATTATATCATGTCCGTAAGCATGGCACC
(P590)_MP-B-DnaB.N1.R	CCGCTACTAGTATTATTAACTCGGTGTGGGAACCTCAGTATTTAGTGCCAATGG
(P591)_MP-M-DnaB.N1.R	CCGCTACTAGTATTATTAGTTGCCAGTGAGGATTGGTGTTTCAACATCCAACGCTTTCC
(P592)_SaP-dpol.N1.R	CCGCTACTAGTATTATTAGCCATCAGTAAGAACCTGAGTGTGTCGATGCAGGCAGG
(P594)_NrdA-2.N1.R	CCGCTACTAGTATTATTAGATAAGTACGTCGATTTTAGCATCGCCCGTGAGACAAAGG
(P595)_Mja-KlbA.N1.R	CCGCTACTAGTATTATTAATCCGACAGATAGATGGGTTCGTCATAGGCCAGCGCTCC
(P597)_Ama-Ter.N3.R	CCGCTACTAGTATTATTAATCCACCTGAATGCAATGGCCTTTCTCCCCGTTAGGAAG
(P598)_Cth-Ter.N3.R	CCGCTACTAGTATTATTAATCCACCTGTATACACCGCATCTTGGTTTTCCCGGTTTTTTC
(P599)_LLP-Pol.N3.R	CCGCTACTAGTATTATTAGGGGACGTTGACGCAATACACGTCCCCTTTATAACTCG
(P600)_LP-Hel.N3.R	CCGCTACTAGTATTATTAACCGTCGACGGTAAAGCCATAGTAATCCCCCACGCCAATGG
(P601)_MP-B-DnaB.N3.R	CCGCTACTAGTATTATTATTCGGTATCAATACCAATGCATTTAACCGGAACGCTCTCAACG
(P602)_MP-M-DnaB.N3.R	CCGCTACTAGTATTATTACGGATGATCAATCTGTATACATACGGTCTCTACGGTAGG
(P603)_SaP-dpol.N3.R	CCGCTACTAGTATTATTAGTCCTCCACATCTATTAGATCATAAACTGGTTCACAGTACG
(P605)_NrdA-2.N3.R	CCGCTACTAGTATTATTAATCTTTGACAGTAATGTCAAACACTGGTTCCTTTGATTCACG
(P606)_Mja-KlbA.N3.R	CCGCTACTAGTATTATTAATCCTCGACGGTTAAATCATAAATGTGGCCATTATAGTCC
(P608)_Ama-Ter.C1.F	GAGGAGAAATACTAGATGGGGTGGGTGACACATGGGGATCTAGTTCCTGGAGATCAGG
(P609)_Cth-Ter.C1.F	GAGGAGAAATACTAGATGGGTTGGACAACAATGGGAGAGATCAAAGCCGGGGACAAGG
(P610)_LLP-Pol.C1.F	GAGGAGAAATACTAGATGGGTTGGGTGAAGTTTGAAGATTACGACGAAAGCATGATGTGTGC
(P611)_LP-Hel.C1.F	GAGGAGAAATACTAGATGGGTACTAAGAAACAGGTTCAGGATATTGCAGTAGGCGAC
(P612)_MP-B-DnaB.C1.F	GAGGAGAAATACTAGATGGGATGGACAACCGTTGGTGATCTGAGCGTCGGGGATTATG
(P613)_MP-M-DnaB.C1.F	GAGGAGAAATACTAGATGGGTTGGAAAAAGATGGGCGACATACAAGTCGGGGACTACGTG
(P614)_SaP-dpol.C1.F	GAGGAGAAATACTAGATGGGATTTAAAGATATCATGGCCGTTACCAGTACTGATAAAGTCTGG
(P616)_NrdA-2.C1.F	GAGGAGAAATACTAGATGGATAATATTCCTATCTCCCAGATATCCCTGGAGG
(P617)_Mja-KlbA.C1.F	GAGGAGAAATACTAGATGGGCAACATTATAAATATTGGTGAATTTGTTGATAAATTTTTC
(P619)_Ama-Ter.C3.F	GAGGAGAAATACTAGATGAGCCCAGACGGTCTGTATTTAGTAGGGAAGAAACTTGTTGC
(P620)_Cth-Ter.C3.F	GAGGAGAAATACTAGATGAGTCCTTCCCGTCTGTATCTCGCCGGCAAATCTATGATACC
(P621)_LLP-Pol.C3.F	GAGGAGAAATACTAGATGACTCATAATATTGTAATTCGTCACAACGATAAGGTGAGC
(P623)_MP-B-DnaB.C3.F	GAGGAGAAATACTAGATGGATCATCTTTTCCAGGTGAGTCGCAGCCGAATCTTAACACAC
(P624)_MP-M-DnaB.C3.F	GAGGAGAAATACTAGATGTCGCATGTGTTTCTGGCTGGTAAAAGTCTGACCCCAACTC
(P625)_SaP-dpol.C3.F	GAGGAGAAATACTAGATGGGTAACCGATTCCTAATTGCGTCAGATTCCGGTTTTTTAGTGG
(P626)_SaP-Hel.C3.F	GAGGAGAAATACTAGATGGATTCTCGGTTCTTATTGGGTGATTTTACAGTTACCCATAACACG
(P627)_NrdA-2.C3.F	GAGGAGAAATACTAGATGAATAGCAATTTTTTTGCCAATAATATCTTGGTGCATAACTGC
(P628)_Mja-KlbA.C3.F	GAGGAGAAATACTAGATGAATCACACCTACATCGCAGGTAAGAACGAGGGCTTCGC
(P633)_RBS30.F	TACTAGAGATTAAAGAGGAGAAATACTAGATG
(P634)_RBS32.F	TACTAGAGTCACACAGGAAAGTACTAGATG
(P653)_PBAD30.R	CATCTAGTATTTCTCCTCTTTAATCTCTAGTAATGGAGAAACAGTAGAGAGTTGCG
(P712)_32-SspGyrB.C2.F	TACTAGAGTCACACAGGAAAGTACTAGATGGAAGCAG
(P764)_P(rhaB)-32.R	ACTTTCCTGTGTGACTCTAGTATACGACCAGTCTAAAAAGCGCCTCAATTCG
(P765)_SspGyrB.N.FII	GGCAGGTGGTTGTTTTTCTGGAGATACATTAGTCG
(P766)_ECF16.SspGyrB.R	TCCAGAAAAACAACCACCTGCCAGCAGATCATGACG
(P767)_SspGyrB.C.RII	TTTTGCACTGTTATGGACAAACACTCCGCTTGC
(P768)_SspGyrB.ECF16.F	TTTGTCCATAACAGTGCAAAATTTGCAGCAAGTGATGAACAGC
(P769)_NrdJ-1.N.FII	AATCCGTGTTGTCTGGTTGGTAGCAGC
(P770)_ECF17.NrdJ.R	ACCAACCAGACAACACGGATTATCGGTGCTACCAACAACATTACG
(P771)_NrdJ-1.C.RII	GATCTCTGAATTATGCACCAGAATATCATTGG
(P772)_NrdJ-1.ECF17.F	CTGGTGCATAATTCAGAGATCCAGAGCACACCGGATG
(P773)_ECF17.R	CCGCTACTAGTATTATTAACGGGTAACACCCAGTTCTTGC
(P774)_M86.N2-T.R	CCCTTTCGGGAGGCCTCTTTTCTGGAATTTGGTACCGAGCTCTAGTATTATTATTCTAGTTTACGGGGTAGAGCAATATGCTCTTTTAAAGATAGC
(P775)_T-P(BAD).F	AAAGAGGCCTCCCGAAAGGGGGGCCTTTTTTCGTTTTGGTCCTACTAGAGGCTATGCCATAGCATTTTTATCCATAAGATTAGCGGATCCTACC
(P776)_P(BAD)-32.R	ACTTTCCTGTGTGACTCTAGTAATGGAGAAACAGTAGAGAGTTGCGATAAAAAGCGTCAGGTAGGATCCGCTAATCTTATGG
(P777)_NrdJ-1.N2-T.R	ACTGTCAGGTGCGGGCTTTTTTCTGTGTTTCCTCTCTAGTATTATTAAATTGCAACCACCAGTTCATCATCATCG
(P778)_T-P(rha).F	AAAAGCCCGCACCTGACAGTGCGGGCTTTTTTTTTCGACCAAAGGTACTAGAGGACCTTTACGCCGCTGGAGCAGG
(P779)_ECF20.R	CCGCTACTAGTATTATTACGGTTTGCGACGACCGCTCAGATCTGC
(P780)_SspGyrB.N2-T.R	CCCTTTCGGGAGGCCTCTTTTCTGGAATTTGGTACCGAGCTCTAGTATTATTACGCATGACCAGAATCTTCCGTAGTCG
(P781)_T-P(lux2).F	AAAGAGGCCTCCCGAAAGGGGGGCCTTTTTTCGTTTTGGTCCTACTAGAGACCTGTAGGATCGTACAGGTTTACGCAAGAAAATGGTTTGTTACTTTCG
(P782)_P(lux2)-33.R	TAGTCCTGTGTGACTCTAGTATTTATTCGAAAGTAACAAACCATTTTCTTGC
(P783)_33-ECF16.F	TACTAGAGTCACACAGGACTACTAGATGCAGCGTACC
(P783)_33-ECF16.F	TACTAGAGTCACACAGGACTACTAGATGCAGCGTACC
(P784)_3T5-P16.R	TTGCATCGGCCGCCAGGGTTACACCGTCGGTGGGTTTCTTTTCATCCAAGCTCTAGAAGCGGCCGCGAATTCG
(P785)_P16-30-mC.F	AACCCTGGCGGCCGATGCAACGAACTAACTTACTAGAGATTAAAGAGGAGAAATACTAGATGGTGAGCAAGG
(P786)_1A3-P17.R	AGTCGAGTAAGAGTTTGGTTCACCAAACGCATCTAAAAAATTTTGTACTGCTCTAGAAGCGGCCGCGAATTCC
(P787)_P17-30-BFP.F	AACCAAACTCTTACTCGACTCGTGTCAGTATACTAGAGATTAAAGAGGAGAAATACTAGATGAGCGAACTGATC
(P800)_32-Cth-Ter.C.F	TACTAGAGTCACACAGGAAAGTACTAGATGTCTCACTTCCACTACATAAAGTCTATTGAAAAAACC
(P802)_33-NrdJ-1.F	TACTAGAGTCACACAGGACTACTAGATGGAAGCCAAAACCTATATCGGCAAACTGAAAAGC

(P802)_33-NrdJ-1.F	TACTAGAGTCACACAGGACTACTAGATGGAAGCCAAAACCTATATCGGCAAACTGAAAAGC
(P804)_T-P(lux2)-33.R	TAGTCCTGTGTGACTCTAGTATTTATTCGAAAGTAACAAACCATTTTCTTGCGTAAACCTGTACGATCCTACAGGTCTCTAGTAGG ACCAAAACGAAAAAAGG
(P807)_imC.F	GTTTCTTCGAATTCGCGGCCGCTTCTAGAGTTATTAGTGGTGATGGTGATGATGC
(P808)_imC.R	GTTTCTTCCTGCAGCGGCCGCTACTAGTACTTGGATGAAAAGAAACCCACCGACG
(P809)_3R-rep.F	GCTTCTAGAGCCAGGCATCAAATAAAACGAAAGGCTCAGTCG
(P810)_3R-rep.R	TCTAGAAGCGGCCGCGAATTCCAGAAATCATCC
(P1360)_e16-SGyrB.N(C-A).F	GCAGGTGGTGCATTTTCTGGAGATACATTAGTCGCTTTAACTGATGG
(P1361)_e16-SGyrB.N(C-A).R	AGAAAATGCACCACCTGCCAGCAGATCATGACG
(P1362)_SGyrB.C(N-A)-e16.F	TTTGTCCATGCAAGTGCAAAATTTGCAGCAAGTGATGAACAGC
(P1363)_SGyrB.C(N-A)-e16.R	TTTTGCACTTGCATGGACAAACACTCCGCTTGC
(P1364)_e17-NrdJ.N(C-A).F	CCGTGTGCACTGGTTGGTAGCAGCGAAATCATTACC
(P1365)_e17-NrdJ.N(C-A).R	ACCAACCAGTGCACACGGATTATCGGTGCTACCAACAACATTACG
(P1366)_NrdJ.C(N-A)-e17.F	GTGCATGCATCAGAGATCCAGAGCACACC
(P1367)_NrdJ.C(N-A)-e17.R	GATCTCTGATGCATGCACCAGAATATCATTGG
(P1368)_e20-M86.C(C-A).F	GTGGATGCTGCAATCTCGGGAGATAGTTTGATCAGCTTGG
(P1369)_e20-M86.C(C-A).R	CGAGATTGCAGCATCCACTTCACAGGCATGTTCG
(P1370)_M86.C(N-A)-e20.F	GTACATGCAAGCGATCTGACCCGTCC
(P1371)_M86.C(N-A)-e20.R	CAGATCGCTTGCATGTACAATGATGTCATTGGCGAC
ECF17_1691_fwd	CGTCTAGAGATTAAAGAGGAGAAATACTAGATGGCACGTGTTAGCGGTG
P16_3622_fwd	AATTCGCGGCCGCTTCTAGAGCTTGGATGAAAAGAAACCCACCGACGGTGTAACCCTGGCGGCCGATGCAACGAACTAACTTA
P16_3622_rev	CTAGTAAGTTAGTTCGTTGCATCGGCCGCCAGGGTTACACCGTCGGTGGGTTTCTTTTCATCCAAGCTCTAGAAGCGGCCGCG
P17_up1691_fwd	AATTCGCGGCCGCTTCTAGAGCAGTACAAAATTTTTTAGATGCGTTTGGTGAACCAAACTCTTACTCGACTCGTGTCAGTATA
P17_up1691_rev	CTAGTATACTGACACGAGTCGAGTAAGAGTTTGGTTCACCAAACGCATCTAAAAAATTTTGTACTGCTCTAGAAGCGGCCGCG
P20_992_fwd	AATTCGCGGCCGCTTCTAGAGGCGCGGATAAAAATTTCATTTGCCCGCGACGGATTCCCCGCCCATCTATCGTTGAACCCATA
P20_992_rev	CTAGTATGGGTTCAACGATAGATGGGCGGGGAATCCGTCGCGGGCAAATGAAATTTTTATCCGCGCCTCTAGAAGCGGCCGCG
(EP49)_Blunt_SasG3_F	ACCATTACAGAGCTGGAGAAGAAGGTTGAG
(EP50)_Blunt_SasG3_R	CGGACCATACTCGGTGAGCTCGTTAATCGG
(EP51)_Gp41-1_C_F	TAATATTAAAGAGGAGAAATACTAGATGCTGAAAAAAATCCTGAAAATCGAGGAA
(EP52)_Gp41-1_C_R	CCTTCTTCTCCAGCTCTGTAATGGTACTGCTAGAATTATGGGTCAGAATATCATT
(EP53)_Gp41-1_N_F	TAACGAGCTCACCGAGTATGGTCCGTCAGGATACTGTCTGGATCTGAAAACCCAG
(EP54)_Gp41-1_N_R	AGCAGCCGGATCCGGTACCTCCTTATTCTTTAACATACAGGCACATACCTTCTTT
(EP55)_IMPDH_C_F	TAATATTAAAGAGGAGAAATACTAGATGACAGATGTAACGATTAAAAAATTCAAA
(EP56)_IMPDH_C_R	CCTTCTTCTCCAGCTCTGTAATGGTGCATATCGAATTATGAACAACGGTGCCACG
(EP57)_IMPDH_N_F	TAACGAGCTCACCGAGTATGGTCCGGGTGGAGGTTGTTTTGTTCCGGGTACACTG
(EP58)_IMPDH_N_R	AGCAGCCGGATCCGGTACCTCCTTATTCCAGCTCGATCAGCAGATGTTTTTTCAT
(P729)_RBS30_gp41-8.C.F	ATTAAAGAGGAGAAATACTAGATGTGTGAAATCTTTGAAAACGAGATCG
(P730)_gp41-8.C_SasG	CCTTCTTCTCCAGCTCTGTAATGGTAACCGCACTATTATGGGTCAGAATACCATTGG
(P727)_SasG_gp41_8.N.F	TTAACGAGCTCACCGAGTATGGTCCGTTGAACAGGTGTCTGAGCCTGGATACCATGG
(P728)_gp41.1.N_pET11.R	TTAGCAGCCGGATCCGGTACCTCCTTATTTTGCACGGCTACGCAGAAAATCGCCCAC
(EP63)_Nrdj-1_C_F	TAATATTAAAGAGGAGAAATACTAGATGGAAGCCAAAACCTATATCGGCAAACTG
(EP64)_Nrdj-1_C_R	CCTTCTTCTCCAGCTCTGTAATGGTGATCTCTGAATTATGCACCAGAATATCATT
(EP65)_Nrdj-1_N_F	TAACGAGCTCACCGAGTATGGTCCGAATCCGTGTTGTCTGGTTGGTAGCAGCGAA
(EP66)_Nrdj-1_N_R	AGCAGCCGGATCCGGTACCTCCTTAAATTGCAACCACCAGTTCATCATCATCGGT
(EP67)_pET11_F	TAAGGAGGTACCGGATCCGGCTGCTAACAAAGC
(EP68)_PET11_RBS30_R	CTAGTATTTCTCCTCTTTAATATTATTTCTAGAGGGGAATTGTTATCCGCT
(EP69)_SasG3_His_R	AGCAGCCGGATCCGGTACCTCCTTAGTGGTGATGGTGATGATGCGGACCATACTCGGTGAGCTCGTTAATCGG
(EP70)_SasG3_Strep_F	TAATATTAAAGAGGAGAAATACTAGATGTGGTCTCATCCTCAATTTGAAAAAACCATTACAGAGCTGGAGAAGAAGGTTGAG
(EP71)_SspGyrB_C_F	TAATATTAAAGAGGAGAAATACTAGATGGAAGCAGTATTAAATTACAATCACAGA
(EP72)_SspGyrB_C_R	CCTTCTTCTCCAGCTCTGTAATGGTTTTTGCACTGTTATGGACAAACACTCCGCT
(EP73)_SspGyrB_N_F	TAACGAGCTCACCGAGTATGGTCCGGCAGGTGGTTGTTTTTCTGGAGATACATTA
(EP74)_SspGyrB_N_R	AGCAGCCGGATCCGGTACCTCCTTACGCATGACCAGAATCTTCCGTAGTCGAAAT
S3_1_GGSG_linker	CTCCAGCTCTGTAATGGTGCCGCTGCCGCCTTTTTCAAATTGAGGATGAGACCAC
(EP93)_S3_1_F	ATATTAAAGAGGAGAAATACTAGATGCATCATCACCATCACCACACCATTACAGAGCTGGAGAAGAAGG
(EP94)_S3_1_R	ATCTCTGAACACGGATTCGGACCATACTCGGTGAG
(EP95)_S3_2_F	ATCCGTGTTCAGAGATCACCATTACAGAGCTGGAGAAGAAGG
(EP96)_S3_2_R	TACTGCTAGAGTATCCTGACGGACCATACTCGGTGAGCT
(EP97)_S3_3_F	GTCAGGATACTCTAGCAGTACCATTACAGAGCTGGAGAAGAAGG
(EP98)_S3_3_R	CATATCGAACCTCCACCCGGACCATACTCGGTGAGCT
(EP99)_S3_4_F	GTGGAGGTTCGATATGCACCATTACAGAGCTGGAGAAGAAGG
(EP104)_S3_6_R	TCCGGTACCTCCTTACGGACCATACTCGGTGAGCT
(EP108)_S3_J1_SeqF	AATCCGTGTTCAGAGATC
(EP109)_S3_J2_SeqF	TCAGGATACTCTAGCAGT
(EP110)_S3_J3_SeqF	GGTGGAGGTTCGATATGC
(EP111)_S3_J4_SeqF	GCAGGTGGTAGTGCAAAA
(EP112)_S3_J5_SeqF	TCAAACCTTTGCAATGAG

Supplementary Table 6 | Regulatory parts and reporters used in this study

Part name	Part type	Sequence[a]	Source
[*araC*/P$_{araBAD}$]	Regulator/ Inducible promoter	TTATGACAACTTGACGGCTACATCATTCACTTTTTCTTCACAACCGGCACGGAACTCGCTCGGGCTGGCCCCGG<br>TGCATTTTTTAAATACCCGCGAGAAATAGAGTTGATCGTCAAAACCAACATTGCGACCGACGGTGGCGATAGGC<br>ATCCGGGTGGTGCTCAAAAGCAGCTTCGCCTGGCTGATACGTTGGTCCTCGCGCCAGCTTAAGACGCTAATCCC<br>TAACTGCTGGCGGAAAAGATGTGACAGACGCGACGGCGACAAGCAAACATGCTGTGCGACGCTGGCGATATCAA<br>AATTGCTGTCTGCCAGGTGATCGCTGATGTACTGACAAGCCTCGCGTACCCGATTATCCATCGGTGGATGGAGC<br>GACTCGTTAATCGCTTCCATGCGCCGCAGTAACAATTGCTCAAGCAGATTTATCGCCAGCAGCTCCGAATAGCG<br>CCCTTCCCCTTGCCCGGCGTTAATGATTTGCCCAAACAGGTCGCTGAAATGCGGCTGGTGCGCTTCATCCGGGC<br>GAAAGAACCCCGTATTGGCAAATATTGACGGCCAGTTAAGCCATTCATGCCAGTAGGCGCGCGGACGAAAGTAA<br>ACCCACTGGTGATACCATTCGCGAGCCTCCGGATGACGACCGTAGTGATGAATCTCTCCTGGCGGGAACAGCAA<br>AATATCACCCGGTCGGCAAACAAATTCTCGTCCCTGATTTTTCACCACCCCCTGACCGCGAATGGTGAGATTGA<br>GAATATAACCTTTCATTCCCAGCGGTCGGTCGATAAAAAAATCGAGATAACCGTTGGCCTCAATCGGCGTTAAA<br>CCCGCCACCAGATGGGCATTAAACGAGTATCCCGGCAGCAGGGGATCATTTTGCGCTTCAGCCATACTTTTCAT<br>ACTCCCGCCATTCAGAGAAGAAACCAATTGTCCATATTGCATCAGACATTGCCGTCACTGCGTCTTTTACTGGC<br>TCTTCTCGCTAACCAAACCGGTAACCCCGCTTATTAAAAGCATTCTGTAACAAAGCGGGACCAAAGCCATGACA<br>AAAACGCGTAACAAAAGTGTCTATAATCACGGCAGAAAAGTCCACATTGATTATTTGCACGGCGTCACACTTTG<br>CTATGCCATAGCATTTTTATCCATAAGATTAGCGGATCCTACCTGACGCTTTTTATCGCAACTCTCTACTGTTT<br>CTCCAT	18
[*rhaS*/P$_{rhaBAD}$]	Regulator/ Inducible promoter	GGACCAAAACGAAAAAAGACGCTTTTCAGCGTCTTATTGTTCGTCTTTGGTACCGAGCTAGTATTATTGCAGAA<br>AGCCATCCCGTCCCTGGCGAATATCACGCGGTGACCAGTTAAACTCTCGGCGAAAAAGCGTCGAAAAGTGGTTA<br>CTGTCGCTGAATCCACAGCGATAGGCGATGTCAGTAACGCTGGCCTCGCTGTGGCGTAGCAGATGTCGGGCTTT<br>CATCAGTCGCAGGCGGTTCAGGTATCGCTGAGGCGTCAGTCCCGTTTGCTGCTTAAGCTGCCGATGTAGCGTAC<br>GCAGTGAAAGAGAAAATTGATCCGCCACGGCATCCCAATTCACCTCATCGGCAAAATGGTCCTCCAGCCAGGCC<br>AGAAGCAAGTTGAGACGTGATGCGCTGTTTTCCAGGTTCTCCTGCAAACTGCTTTTACGCAGCAAGAGCAGTAA<br>TTGCATAAACAAGATCTCGCGACTGGCGGTCGAGGGTAAATCATTTTCCCCTTCCTGCTGTTCCATCTGTGCAA<br>CCAGCTGTCGCACCTGCTGCAATACGCTGTGGTTAACGCGCCAGTGAGACGGATACTGCCCATCCAGCTCTTGT<br>GGCAGCAACTGATTCAGCCCGGCGAGAAACTGAAATCGATCCGGCGAGCGATACAGCACATTGGTCAGACACAG<br>ATTATCGGTATGTTCATACAGATGCCGATCATGATCGCGTACGAAACAGACCGTGCCACCGGTGATGGTATAGG<br>GCTGCCCATTAAACACATGAATACCCGTGCCATGTTCGACAATCACAATTTCATGAAAATCATGATGATGTTCA<br>GGAAAATCCGCCTGCGGGAGCCGGGGTTCTATCGCCACGGACGCGTTACCGGACGGAAAAAAATCCACACTATG<br>TAATACGGTCATTTGGGCTCCCTCTAGTAGCTAGCATAATACCTAGGACTGAGCTAGCTGTAAACTCTAGTATC<br>ACACTGGCTCACCTTCGGGTGGGCCTTTCTGCGTTTATATACTAGAGAGACCTTTACGCCGCTGGAGCAGGAAT<br>GCGGTGAGCATCACATCACCACAATTCAGCAAATTGTGAACATCATCACGTTCATCTTTCCCTGGTTGCCAATG<br>GCCCATTTTCCTGTCAGTAACGAGAAGGTCGCGAATTGAGGCGCTTTTTAGACTGGTCGTA	19
[*luxR*/P$_{lux2}$]	Regulator/ Inducible promoter	TTTATAGCTAGCTCAGCCCTTGGTACAATGCTAGCTACTAGAGATTAAAGAGGAGAAATACTAGATGAAAAACA<br>TAAATGCCGACGACACATACAGAATAATTAATAAAATTAAAGCTTGTAGAAGCAATAATGATATTAATCAATGC<br>TTATCTGATATGACTAAAATGGTACATTGTGAATATTATTTACTCGCGATCATTTATCCTCATTCTATGGTTAA<br>ATCTGATATTTCAATCCTAGATAATTACCCTAAAAAATGGAGGCAATATTATGATGACGCTAATTTAATAAAAT<br>ATGATCCTATAGTAGATTATTCTAACTCCAATCATTCACCAATTAATTGGAATATATTTGAAAACAATGCTGTA<br>AATAAAAAATCTCCAAATGTAATTAAAGAAGCGAAAACATCAGGTCTTATCACTGGGTTTAGTTTCCCTATTCA<br>TACGGCTAACAATGGCTTCGGAATGCTTAGTTTTGCACATTCAGAAAAAGACAACTATATAGATAGTTTATTTT<br>TACATGCGTGTATGAACATACCATTAATTGTTCCTTCTCTAGTTGATAATTATCGAAAAATAAATATAGCAAAT<br>AATAAATCAAACAACGATTTAACCAAAAGAGAAAAAGAATGTTTAGCGTGGGCATGCGAAGGAAAAAGCTCTTG<br>GGATATTTCAAAAATATTAGGTTGCAGTGAGCGTACTGTCACTTTCCATTTAACCAATGCGCAAATGAAACTCA<br>ATACAACAAACCGCTGCCAAAGTATTTCTAAAGCAATTTTAACAGGAGCAATTGATTGCCCATACTTTAAAAAT<br>TAATAACACTGATAGTGCTAGTGTAGATCACTACTAGAGCCAGGCATCAAATAAAACGAAAGGCTCAGTCGAAA<br>GACTGGGCCTTTCGTTTTATCTGTTGTTTGTCGGTGAACGCTCTCTACTAGAGTCACACTGGCTCACCTTCGGG<br>TGGGCCTTTCTGCGTTTATATACTAGAGACCTGTAGGATCGTACAGGTTTACGCAAGAAAATGGTTTGTTACTT<br>TCGAATAAA	20
P$_{16_3622}$	Inducible promoter	CTTGGATGAAAAGAAACCCACCGACGGTGTAACCCTGGCGGCCGATGCAACGAACTAACT	21
P$_{17_Up1691}$	Inducible promoter	CAGTACAAAATTTTTTAGATGCGTTTGGTGAACCAAACTCTTACTCGACTCGTGTCAGTA	21
P$_{20_992}$	Inducible promoter	GCGCGGATAAAAATTTCATTTGCCCGCGACGGATTCCCCGCCCATCTATCGTTGAACCCA	21
ecf16_3622	Regulator	ATGCAGCGTACCAATAGCCAGGATGTTCTGAGCACCCGTGAAAGCCAGCTGCAGGCATTACTGCTGCAGGGTCT<br>GGCAGGCGATACCTTTGCATATCGTCAGTTTCTGACCGCACTGGCAGCACATATTCGTGGTTTTCTGCGTCGTC<br>GTCTGCCGCAGCATCCGGCAGAAGTTGAAGATCTGCTGCAAGAAGTTCTGCTGGCAGTTCATAATGCACGTCAT<br>ACCTATCAGGCACGTCAGCCGCTGACCGCATGGGTTCAGGCAATTGCACGTTATAAACTGGCAGATCATCTGCG<br>TAGCCATGCACGTCGTGAAGCACGTCATGATCTGCTGGATGATGATAGCGAACTGTTTGCAGCAAGTGATGAAC<br>AGCCTGCACAGGCAAGCCGTGATCTGGGTAAACTGCTGGGTCAGCTGCCGGATCGTCAGCGTCTGCCGATTGTT<br>CATGTTAAACTGGAAGGTCTGAGCGTTGAAGAAACCGCACAGATTACCGGTCTGAGCAGCAGCGCAGTTAAAGT<br>TGGTATTCATCGTGGTCTGAAAGCACTGGGCAAACTGATTCGTGGTAAAGGTCATGATGAAGATCGTTAA	21
ecf17_1691	Regulator	ATGGCACGTGTTAGCGGTGCAGCAGCAGCCGAAGCAGCACTGATGCGTGCACTGTATGATGAACATGCAGCAGT<br>TCTGTGGCGTTATGCACTGCGTCTGACCGGTGATGCAGCACAGGCAGAAGATGTTGTTCAAGAAACCCTGCTGC<br>GTGCATGGCAGCATCCGGAAGTTATTGGTGATACCGCACGTCCGGCACGCGCATGGCTGTTTACCGTTGCACGT<br>AATATGATTATTGATGAACGTCGTAGCGCACGTTTTCGTAATGTTGTTGGTAGCACCGATCAGAGCGGTACACC<br>GGAACAGAGCACACCGGATGAAGTTAATGCAGCACTGGATCGTCTGCTGATTGCAGATGCACTGGCACAGCTGA<br>GCGCAGAACATCGTGCAGTTATTCAGCGTAGCTATTATCGTGGTTGGAGCACCGCACAGATTGCAACCGATCTG<br>GGTATTGCAGAAGGCACCGTTAAAAGCCGTCTGCATTATGCAGTTCGTGCCCTGCGCCTGACCCTGCAAGAACT<br>GGGTGTTACCCGTTAA	21
ecf20_992	Regulator	ATGAATGAAACCGATCCTGATCTGGAACTGCTGAAACGTATTGGTAATAATGATGCACAGGCCGTTAAAGAAAT<br>GGTTACCCGTAAACTGCCTCGTCTGCTGGCACTGGCAAGTCGCCTGCTGGGTGATGCAGATGAAGCACGTGATA<br>TTGCACAAGAAAGTTTTCTGCGCATTTGGAAACAGGCAGCAAGCTGGCGTAGCGAACAGGCACGTTTTGATACC<br>TGGCTGCATCGTGTTGCACTGAATCTGTGTTATGATCGTCTGCGTCGTCGTAAAGAACATGTGCCGGTTGATAG<br>CGAACATGCCTGTGAAGCACTGGATACCCGTCCGGCACCGGATGAACAGCTGGAAGCAAGCGCACAGAGCCGTC<br>GTATGGCACAGGCACTGGATCAGCTGCCGGATCGTCAGCGTGAAGCAATTGTTCTGCAGTATTATCAAGAACTG<br>AGCAATACCGAAGCAGCAGCACTGATGCAAATTAGCGTTGAAGCCCTGGAAAGCCTGCTGAGCCGTGCACGTCG<br>TAATCTGCGTAGCCATCTGGCCGAAGCACCGGGTGCAGATCTGAGCGGTCGTCGCAAACCGTAA	21

B0030	RBS	ATTAAAGAGGAGAAA	18
B0032	RBS	TCACACAGGAAAG	18
B0033	RBS	TCACACAGGAC	18
B0015	Terminator	CCAGGCATCAAATAAAACGAAAGGCTCAGTCGAAAGACTGGGCCTTTCGTTTTATCTGTTGTTTGTCGGTGAAC<br>GCTCTCTACTAGAGTCACACTGGCTCACCTTCGGGTGGGCCTTTCTGCGTTTATA	18
L3S2P21	Terminator	CTCGGTACCAAATTCCAGAAAAGAGGCCTCCCGAAAGGGGGGCCTTTTTTCGTTTTGGTCC	22
ECK120033737	Terminator	GGAAACACAGAAAAAAGCCCGCACCTGACAGTGCGGGCTTTTTTTTTCGACCAAAGG	22
mCherry	Reporter	ATGGTGAGCAAGGGCGAAGAAGACAACATGGCCATCATCAAGGAGTTCATGCGCTTCAAGGTGCACATGGAGGG<br>CTCCGTGAACGGCCACGAGTTCGAGATCGAGGGCGAGGGCGAGGGCCGCCCCTACGAGGGCACCCAGACCGCCA<br>AGCTGAAGGTGACCAAGGGTGGCCCCCTGCCCTTCGCCTGGGACATCCTGTCCCCTCAGTTCATGTACGGCTCC<br>AAGGCCTACGTGAAGCACCCCGCCGACATCCCCGACTACTTGAAGCTGTCCTTCCCCGAGGGCTTCAAGTGGGA<br>GCGCGTGATGAACTTCGAGGACGGCGGCGTGGTGACCGTGACCCAGGACTCCTCCTTGCAGGACGGCGAGTTCA<br>TCTACAAGGTGAAGCTGCGCGGCACCAACTTCCCCTCCGACGGCCCCGTAATGCAGAAGAAGACCATGGGCTGG<br>GAGGCCTCCTCCGAGCGGATGTACCCCGAGGACGGCGCCCTGAAGGGCGAGATCAAGCAGAGGCTGAAGCTGAA<br>GGACGGCGGCCACTACGACGCTGAGGTCAAGACCACCTACAAGGCCAAGAAGCCCGTGCAGCTGCCCGGCGCCT<br>ACAACGTCAACATCAAGTTGGACATCACCTCCCACAACGAGGACTACACCATCGTGGAACAGTACGAACGCGCC<br>GAGGGCCGCCACTCCACCGGCGGCATGGACGAGCTGTACAAGTAA	18
bfp	Reporter	ATGAGCGAACTGATCAAAGAGAACATGCACATGAAGCTGTACATGGAAGGCACCGTTGACAACCACCACTTTAA<br>GTGCACGTCTGAGGGTGAGGGTAAGCCGTACGAAGGCACCCAAACCATGCGTATCAAAGTTGTGGAGGGCGGTC<br>CACTGCCGTTCGCTTTTGACATTCTGGCGACCAGCTTCCTGTACGGTTCCAAAACGTTCATTAACCATACTCAG<br>GGCATTCCGGATTTCTTCAAACAGAGCTTTCCGGAAGGTTTCACCTGGGAGCGTGTCACCACGTATGAAGATGG<br>TGGTGTGTTGACCGCCACCCAAGATACCTCCCTGCAAGATGGCTGTCTGATCTATAACGTGAAAATTCGTGGCG<br>TCAACTTTACGAGCAATGGTCCGGTGATGCAGAAGAAAACCCTGGGTTGGGAGGCGTTTACGGAAACCCTGTAT<br>CCGGCCGATGGTGGCCTGGAGGGCCGTAACGACATGGCACTGAAGCTGGTTGGTGGCAGCCATTTGATCGCAAA<br>TATCAAGACGACGTACCGCAGCAAGAAACCGGCGAAAAATCTGAAGATGCCGGGTGTTTACTATGTCGACTACC<br>GTCTGGAACGCATTAAAGAAGCGAATAATGAGACTTACGTGGAGCAGCACGAGGTTGCAGTCGCGCGCTATTGC<br>GACTTGCCTAGCAAGCTGGGTCATAAACTGAATTAA	18
gfp	Reporter	ATGCGTAAAGGAGAAGAACTTTTCACTGGAGTTGTCCCAATTCTTGTTGAATTAGATGGTGATGTTAATGGGCA<br>CAAATTTTCTGTCAGTGGAGAGGGTGAAGGTGATGCAACATACGGAAAACTTACCCTTAAATTTATTTGCACTA<br>CTGGAAAACTACCTGTTCCATGGCCAACACTTGTCACTACTTTCGGTTATGGTGTTCAATGCTTTGCGAGATAC<br>CCAGATCATATGAAACAGCATGACTTTTTCAAGAGTGCCATGCCCGAAGGTTATGTACAGGAAAGAACTATATT<br>TTTCAAAGATGACGGGAACTACAAGACACGTGCTGAAGTCAAGTTTGAAGGTGATACCCTTGTTAATAGAATCG<br>AGTTAAAAGGTATTGATTTTAAAGAAGATGGAAACATTCTTGGACACAAATTGGAATACAACTATAACTCACAC<br>AATGTATACATCATGGCAGACAAACAAAAGAATGGAATCAAAGTTAACTTCAAAATTAGACACAACATTGAAGA<br>TGGAAGCGTTCAACTAGCAGACCATTATCAACAAAATACTCCAATTGGCGATGGCCCTGTCCTTTTACCAGACA<br>ACCATTACCTGTCCACACAATCTGCCCTTTCGAAAGATCCCAACGAAAAGAGAGACCACATGGTCCTTCTTGAG<br>TTTGTAACAGCTGCTGGGATTACACATGGCATGGATGAACTATACAAATAA	20

[a] DNA sequence colors correspond to promoters (orange), RBSs (green), protein coding sequences (blue), and terminators (purple), and spacers (black).

Supplementary Table 7 | List of the plasmids used in this study

Name	Description	Backbone	Origin	Resistance[a]	Source
pSB3T5	BioBrick vector	pSB3T5	p15A	Tet	18
pSB1A3	BioBrick vector	pSB1A3	pMB1	Amp	18
pSB4A3	BioBrick vector	pSB4A3	pSC101	Amp	18
pSB3K3	BioBrick vector	pSB3K3	p15A	Kan	18
pSEVA221	SEVA vector	pSEVA221	RK2	Kan	23
pET-HT	Expression vector (pET11-derived)	pET-HT	pBR322	Amp	b
pSB1C3-BBa_I0500	Plasmid containing the arabinose-inducible promoter and regulator	pSB1C3	pMB1	Chl	18
pUpR-sfGFP	Plasmid containing the rhamnose-inducible promoter and regulator	pSB1K3	pMB1	Kan	c
pBW313J115-luxR	Plasmid containing the AHL-inducible promoter and regulator and the GFPmut3b encoding gene	pSB3K3	p15A	Kan	20
pSB1C3-BBa_J06504	Plasmid containing mCherry encoding gene	pSB1C3	pMB1	Chl	18
pSB1C3-BBa_K592026	Plasmid containing mTagBFP encoding gene	pSB1C3	pMB1	Chl	18
pVRa-ECF16_3622	Plasmid containing the ECF16_3622 encoding gene	pVRa	pBR322	Amp	21
pVRa-ECF17_1691	Plasmid containing the ECF17_1691 encoding gene	pVRa	pBR322	Amp	21
pVRa-ECF20_992	Plasmid containing the ECF20_992 encoding gene	pVRa	pBR322	Amp	21
pSasG5^1–G5^3	Plasmid containing the SasG5^{3}E^2 encoding gene	pET-YSBLIC	pBR322	Kan	24,d

[a] Tet - Tetracycline; Amp - Ampicillin; Kan - Kanamycin; Chl - Chloramphenicol
[b] Kindly provided by Prof. Lynne Regan
[c] Kindly provided by Yang Liu
[d] Kindly provided by Prof. Jennifer R. Potts

Supplementary Table 8 | List and structure of the plasmids constructed in this study

Name	Structure[a]	Backbone	Origin	Resistance[b]
pFP.R193	[*araC*/P_{araBAD}]-B0030-*mCherry.H*$_6$	pSB3T5	p15A	Tet
pFP.R574	[*araC*/P_{araBAD}]-B0030-*mCherry*^{N}	pSB3T5	p15A	Tet
pFP.R576	[*araC*/P_{araBAD}]-B0030-*mCherry*^{C}*.H*$_6$	pSB1A3	pMB1	Amp
pFP.R575	[*araC*/P_{araBAD}]-B0030-*mCherry*^{N}*(SGY)*	pSB3T5	p15A	Tet
pFP.R577	[*araC*/P_{araBAD}]-B0030-*(SSS)mCherry*^{C}*.H*$_6$	pSB1A3	pMB1	Amp
pFP.R222	[*araC*/P_{araBAD}]-B0030-*mCherry*^{N}*(VDASDL)mCherry*^{C}*.H*$_6$	pSB3T5	p15A	Tet
pFP.R223	[*araC*/P_{araBAD}]-B0030-*mCherry*^{N}*(GGKCWN)mCherry*^{C}*.H*$_6$	pSB3T5	p15A	Tet
pFP.R194	[*araC*/P_{araBAD}]-B0030-*mCherry*^{N}*(SGYSSS)mCherry*^{C}*.H*$_6$	pSB3T5	p15A	Tet
pFP.R224	[*araC*/P_{araBAD}]-B0030-*mCherry*^{N}*(LNRSAV)mCherry*^{C}*.H*$_6$	pSB3T5	p15A	Tet
pFP.R225	[*araC*/P_{araBAD}]-B0030-*mCherry*^{N}*(NPCSEI)mCherry*^{C}*.H*$_6$	pSB3T5	p15A	Tet
pFP.R226	[*araC*/P_{araBAD}]-B0030-*mCherry*^{N}*(GGGSIC)mCherry*^{C}*.H*$_6$	pSB3T5	p15A	Tet
pFP.R227	[*araC*/P_{araBAD}]-B0030-*mCherry*^{N}*(IDECHT)mCherry*^{C}*.H*$_6$	pSB3T5	p15A	Tet
pFP.R228	[*araC*/P_{araBAD}]-B0030-*mCherry*^{N}*(AGGSAK)mCherry*^{C}*.H*$_6$	pSB3T5	p15A	Tet
pFP.R229	[*araC*/P_{araBAD}]-B0030-*mCherry*^{N}*(IGCSFD)mCherry*^{C}*.H*$_6$	pSB3T5	p15A	Tet
pFP.R230	[*araC*/P_{araBAD}]-B0030-*mCherry*^{N}*(GSKTVI)mCherry*^{C}*.H*$_6$	pSB3T5	p15A	Tet
pFP.R231	[*araC*/P_{araBAD}]-B0030-*mCherry*^{N}*(SGKTQL)mCherry*^{C}*.H*$_6$	pSB3T5	p15A	Tet
pFP.R378	[*araC*/P_{araBAD}]-B0030-*mCherry*^{N}*(VNQSGR)mCherry*^{C}*.H*$_6$	pSB3T5	p15A	Tet
pFP.R379	[*araC*/P_{araBAD}]-B0030-*mCherry*^{N}*(DKFSTK)mCherry*^{C}*.H*$_6$	pSB3T5	p15A	Tet
pFP.R380	[*araC*/P_{araBAD}]-B0030-*mCherry*^{N}*(GSKCSN)mCherry*^{C}*.H*$_6$	pSB3T5	p15A	Tet
pFP.R381	[*araC*/P_{araBAD}]-B0030-*mCherry*^{N}*(SNLCSE)mCherry*^{C}*.H*$_6$	pSB3T5	p15A	Tet
pFP.R382	[*araC*/P_{araBAD}]-B0030-*mCherry*^{N}*(KHHSVL)mCherry*^{C}*.H*$_6$	pSB3T5	p15A	Tet
pFP.R383	[*araC*/P_{araBAD}]-B0030-*mCherry*^{N}*(HGKSEL)mCherry*^{C}*.H*$_6$	pSB3T5	p15A	Tet
pFP.R384	[*araC*/P_{araBAD}]-B0030-*mCherry*^{N}*(ASCCRL)mCherry*^{C}*.H*$_6$	pSB3T5	p15A	Tet
pFP.R385	[*araC*/P_{araBAD}]-B0030-*mCherry*^{N}*(NGKTTL)mCherry*^{C}*.H*$_6$	pSB3T5	p15A	Tet
pFP.R386	[*araC*/P_{araBAD}]-B0030-*mCherry*^{N}*(NGKSEL)mCherry*^{C}*.H*$_6$	pSB3T5	p15A	Tet
pFP.R387	[*araC*/P_{araBAD}]-B0030-*mCherry*^{N}*(HGKTEV)mCherry*^{C}*.H*$_6$	pSB3T5	p15A	Tet
pFP.R388	[*araC*/P_{araBAD}]-B0030-*mCherry*^{N}*(AKACNF)mCherry*^{C}*.H*$_6$	pSB3T5	p15A	Tet
pFP.R389	[*araC*/P_{araBAD}]-B0030-*mCherry*^{N}*(ATGCGK)mCherry*^{C}*.H*$_6$	pSB3T5	p15A	Tet
pFP.R390	[*araC*/P_{araBAD}]-B0030-*mCherry*^{N}*(QDQTKN)mCherry*^{C}*.H*$_6$	pSB3T5	p15A	Tet
pFP.R391	[*araC*/P_{araBAD}]-B0030-*mCherry*^{N}*(NHDSRA)mCherry*^{C}*.H*$_6$	pSB3T5	p15A	Tet
pFP.R392	[*araC*/P_{araBAD}]-B0030-*mCherry*^{N}*(ELKTQN)mCherry*^{C}*.H*$_6$	pSB3T5	p15A	Tet
pFP.R393	[*araC*/P_{araBAD}]-B0030-*mCherry*^{N}*(NGKTEL)mCherry*^{C}*.H*$_6$	pSB3T5	p15A	Tet
pFP.R394	[*araC*/P_{araBAD}]-B0030-*mCherry*^{N}*(AGKSLT)mCherry*^{C}*.H*$_6$	pSB3T5	p15A	Tet
pFP.R395	[*araC*/P_{araBAD}]-B0030-*mCherry*^{N}*(GKACEL)mCherry*^{C}*.H*$_6$	pSB3T5	p15A	Tet
pFP.R396	[*araC*/P_{araBAD}]-B0030-*mCherry*^{N}*(SGKTSS)mCherry*^{C}*.H*$_6$	pSB3T5	p15A	Tet
pFP.R397	[*araC*/P_{araBAD}]-B0030-*mCherry*^{N}*(SNLCNE)mCherry*^{C}*.H*$_6$	pSB3T5	p15A	Tet
pFP.R398	[*araC*/P_{araBAD}]-B0030-*mCherry*^{N}*(GGGTGL)mCherry*^{C}*.H*$_6$	pSB3T5	p15A	Tet
pFP.R399	[*araC*/P_{araBAD}]-B0030-*mCherry*^{N}*(HDGCSG)mCherry*^{C}*.H*$_6$	pSB3T5	p15A	Tet
pFP.R265	[*araC*/P_{araBAD}]-B0030-*mCherry*^{N}*(VDA).M86.(SDL)mCherry*^{C}*.H*$_6$	pSB3T5	p15A	Tet
pFP.R266	[*araC*/P_{araBAD}]-B0030-*mCherry*^{N}*(GGK).NpuSsp.(CWN)mCherry*^{C}*.H*$_6$	pSB3T5	p15A	Tet
pFP.R267	[*araC*/P_{araBAD}]-B0030-*mCherry*^{N}*(SGY).gp41-1.(SSS)mCherry*^{C}*.H*$_6$	pSB3T5	p15A	Tet
pFP.R268	[*araC*/P_{araBAD}]-B0030-*mCherry*^{N}*(LNR).gp41-8.(SAV)mCherry*^{C}*.H*$_6$	pSB3T5	p15A	Tet
pFP.R269	[*araC*/P_{araBAD}]-B0030-*mCherry*^{N}*(NPC).NrdJ-1.(SEI)mCherry*^{C}*.H*$_6$	pSB3T5	p15A	Tet
pFP.R270	[*araC*/P_{araBAD}]-B0030-*mCherry*^{N}*(GGG).IMPDH-1.(SIC)mCherry*^{C}*.H*$_6$	pSB3T5	p15A	Tet
pFP.R271	[*araC*/P_{araBAD}]-B0030-*mCherry*^{N}*(IDE).SspDnaX.(CHT)mCherry*^{C}*.H*$_6$	pSB3T5	p15A	Tet
pFP.R272	[*araC*/P_{araBAD}]-B0030-*mCherry*^{N}*(AGG).SspGyrB.(SAK)mCherry*^{C}*.H*$_6$	pSB3T5	p15A	Tet
pFP.R273	[*araC*/P_{araBAD}]-B0030-*mCherry*^{N}*(IGC).TerThyX.(SFD)mCherry*^{C}*.H*$_6$	pSB3T5	p15A	Tet
pFP.R274	[*araC*/P_{araBAD}]-B0030-*mCherry*^{N}*(GSK).TvoVMA.(TVI)mCherry*^{C}*.H*$_6$	pSB3T5	p15A	Tet
pFP.R275	[*araC*/P_{araBAD}]-B0030-*mCherry*^{N}*(SGK).PhoRadA.(TQL)mCherry*^{C}*.H*$_6$	pSB3T5	p15A	Tet
pFP.R355	[*araC*/P_{araBAD}]-B0030-*mCherry*^{N}*(VNQ).Cro-RIR1*.(SGR)mCherry*^{C}*.H*$_6$	pSB3T5	p15A	Tet
pFP.R356	[*araC*/P_{araBAD}]-B0030-*mCherry*^{N}*(DKF).Cro-RPB2*.(STK)mCherry*^{C}*.H*$_6$	pSB3T5	p15A	Tet
pFP.R357	[*araC*/P_{araBAD}]-B0030-*mCherry*^{N}*(GSK).Cro-Top2*.(CSN)mCherry*^{C}*.H*$_6$	pSB3T5	p15A	Tet
pFP.R358	[*araC*/P_{araBAD}]-B0030-*mCherry*^{N}*(SNL).CIV-RIR1*.(CSE)mCherry*^{C}*.H*$_6$	pSB3T5	p15A	Tet
pFP.R359	[*araC*/P_{araBAD}]-B0030-*mCherry*^{N}*(KHH).CP-Thy1*.(SVL)mCherry*^{C}*.H*$_6$	pSB3T5	p15A	Tet
pFP.R360	[*araC*/P_{araBAD}]-B0030-*mCherry*^{N}*(HGK).Ama-Ter*.(SEL)mCherry*^{C}*.H*$_6$	pSB3T5	p15A	Tet

pFP.R361	[araC/P_{araBAD}]-B0030-mCherry^{N}(SNL).BsuP-RIR1*.(CSE)mCherry^{C}.H_6	pSB3T5	p15A	Tet
pFP.R362	[araC/P_{araBAD}]-B0030-mCherry^{N}(ASC).CbP-RNR*.(CRL)mCherry^{C}.H_6	pSB3T5	p15A	Tet
pFP.R363	[araC/P_{araBAD}]-B0030-mCherry^{N}(NGK).Ckl-Ter*.(TTL)mCherry^{C}.H_6	pSB3T5	p15A	Tet
pFP.R364	[araC/P_{araBAD}]-B0030-mCherry^{N}(NGK).Cth-Ter*.(SEL)mCherry^{C}.H_6	pSB3T5	p15A	Tet
pFP.R365	[araC/P_{araBAD}]-B0030-mCherry^{N}(HGK).EP-Pri*.(TEV)mCherry^{C}.H_6	pSB3T5	p15A	Tet
pFP.R366	[araC/P_{araBAD}]-B0030-mCherry^{N}(AKA).LLP-Pol*.(CNF)mCherry^{C}.H_6	pSB3T5	p15A	Tet
pFP.R367	[araC/P_{araBAD}]-B0030-mCherry^{N}(ATG).LP-Hel*.(CGK)mCherry^{C}.H_6	pSB3T5	p15A	Tet
pFP.R368	[araC/P_{araBAD}]-B0030-mCherry^{N}(QDQ).MP-B-DnaB*.(TKN)mCherry^{C}.H_6	pSB3T5	p15A	Tet
pFP.R369	[araC/P_{araBAD}]-B0030-mCherry^{N}(NHD).MP-K-gp53*.(SRA)mCherry^{C}.H_6	pSB3T5	p15A	Tet
pFP.R370	[araC/P_{araBAD}]-B0030-mCherry^{N}(ELK).MP-C-gp206*.(TQN)mCherry^{C}.H_6	pSB3T5	p15A	Tet
pFP.R371	[araC/P_{araBAD}]-B0030-mCherry^{N}(NGK).MP-M-DnaB*.(TEL)mCherry^{C}.H_6	pSB3T5	p15A	Tet
pFP.R372	[araC/P_{araBAD}]-B0030-mCherry^{N}(AGK).PP-Phi*.(SLT)mCherry^{C}.H_6	pSB3T5	p15A	Tet
pFP.R373	[araC/P_{araBAD}]-B0030-mCherry^{N}(GKA).SaP-dpol*.(CEL)mCherry^{C}.H_6	pSB3T5	p15A	Tet
pFP.R374	[araC/P_{araBAD}]-B0030-mCherry^{N}(SGK).SaP-Hel*.(TSS)mCherry^{C}.H_6	pSB3T5	p15A	Tet
pFP.R375	[araC/P_{araBAD}]-B0030-mCherry^{N}(SNL).NrdA-2*.(CNE)mCherry^{C}.H_6	pSB3T5	p15A	Tet
pFP.R376	[araC/P_{araBAD}]-B0030-mCherry^{N}(GGG).Pfu-RIR1-1*.(TGL)mCherry^{C}.H_6	pSB3T5	p15A	Tet
pFP.R377	[araC/P_{araBAD}]-B0030-mCherry^{N}(HDG).Mja-KlbA*.(CSG)mCherry^{C}.H_6	pSB3T5	p15A	Tet
pFP.R400	[araC/P_{araBAD}]-B0030-mCherry^{N}(GGK).NpuSsp*.(CWN)mCherry^{C}.H_6	pSB3T5	p15A	Tet
pFP.R401	[araC/P_{araBAD}]-B0030-mCherry^{N}(IGC).TerThyX*.(SFD)mCherry^{C}.H_6	pSB3T5	p15A	Tet
pFP.R276	[araC/P_{araBAD}]-B0030-mCherry^{N}(VDA).M86^{N1}	pSB3T5	p15A	Tet
pFP.R277	[araC/P_{araBAD}]-B0030-mCherry^{N}(GGK).NpuSsp^{N1}	pSB3T5	p15A	Tet
pFP.R278	[araC/P_{araBAD}]-B0030-mCherry^{N}(SGY).gp41-1^{N1}	pSB3T5	p15A	Tet
pFP.R279	[araC/P_{araBAD}]-B0030-mCherry^{N}(LNR).gp41-8^{N1}	pSB3T5	p15A	Tet
pFP.R280	[araC/P_{araBAD}]-B0030-mCherry^{N}(NPC).NrdJ-1^{N1}	pSB3T5	p15A	Tet
pFP.R281	[araC/P_{araBAD}]-B0030-mCherry^{N}(GGG).IMPDH-1^{N1}	pSB3T5	p15A	Tet
pFP.R282	[araC/P_{araBAD}]-B0030-mCherry^{N}(IDE).SspDnaX^{N1}	pSB3T5	p15A	Tet
pFP.R283	[araC/P_{araBAD}]-B0030-mCherry^{N}(AGG).SspGyrB^{N1}	pSB3T5	p15A	Tet
pFP.R284	[araC/P_{araBAD}]-B0030-mCherry^{N}(IGC).TerThyX^{N1}	pSB3T5	p15A	Tet
pFP.R285	[araC/P_{araBAD}]-B0030-mCherry^{N}(GSK).TvoVMA^{N1}	pSB3T5	p15A	Tet
pFP.R286	[araC/P_{araBAD}]-B0030-mCherry^{N}(SGK).PhoRadA^{N1}	pSB3T5	p15A	Tet
pFP.R448	[araC/P_{araBAD}]-B0030-mCherry^{N}(HGK).Ama-Ter^{N1}	pSB3T5	p15A	Tet
pFP.R449	[araC/P_{araBAD}]-B0030-mCherry^{N}(NGK).Cth-Ter^{N1}	pSB3T5	p15A	Tet
pFP.R450	[araC/P_{araBAD}]-B0030-mCherry^{N}(AKA).LLP-Pol^{N1}	pSB3T5	p15A	Tet
pFP.R451	[araC/P_{araBAD}]-B0030-mCherry^{N}(ATG).LP.Hel^{N1}	pSB3T5	p15A	Tet
pFP.R452	[araC/P_{araBAD}]-B0030-mCherry^{N}(QDQ).MP-B-DnaB^{N1}	pSB3T5	p15A	Tet
pFP.R453	[araC/P_{araBAD}]-B0030-mCherry^{N}(NGK).MP-M-DnaB^{N1}	pSB3T5	p15A	Tet
pFP.R454	[araC/P_{araBAD}]-B0030-mCherry^{N}(GKA).SaP-dpol^{N1}	pSB3T5	p15A	Tet
pFP.R456	[araC/P_{araBAD}]-B0030-mCherry^{N}(SNL).NrdA-2^{N1}	pSB3T5	p15A	Tet
pFP.R457	[araC/P_{araBAD}]-B0030-mCherry^{N}(HDG).Mja-KlbA^{N1}	pSB3T5	p15A	Tet
pFP.R232	[araC/P_{araBAD}]-B0030-mCherry^{N}(VDA).M86^{N2}	pSB3T5	p15A	Tet
pFP.R233	[araC/P_{araBAD}]-B0030-mCherry^{N}(GGK).NpuSsp^{N2}	pSB3T5	p15A	Tet
pFP.R195	[araC/P_{araBAD}]-B0030-mCherry^{N}(SGY).gp41-1^{N2}	pSB3T5	p15A	Tet
pFP.R264	[araC/P_{araBAD}]-B0030-mCherry^{N}(LNR).gp41-8^{N2}	pSB3T5	p15A	Tet
pFP.R235	[araC/P_{araBAD}]-B0030-mCherry^{N}(NPC).NrdJ-1^{N2}	pSB3T5	p15A	Tet
pFP.R236	[araC/P_{araBAD}]-B0030-mCherry^{N}(GGG).IMPDH-1^{N2}	pSB3T5	p15A	Tet
pFP.R237	[araC/P_{araBAD}]-B0030-mCherry^{N}(IDE).SspDnaX^{N2}	pSB3T5	p15A	Tet
pFP.R238	[araC/P_{araBAD}]-B0030-mCherry^{N}(AGG).SspGyrB^{N2}	pSB3T5	p15A	Tet
pFP.R239	[araC/P_{araBAD}]-B0030-mCherry^{N}(IGC).TerThyX^{N2}	pSB3T5	p15A	Tet
pFP.R443	[araC/P_{araBAD}]-B0030-mCherry^{N}(GSK).TvoVMA^{N2}	pSB3T5	p15A	Tet
pFP.R241	[araC/P_{araBAD}]-B0030-mCherry^{N}(SGK).PhoRadA^{N2}	pSB3T5	p15A	Tet
pFP.R406	[araC/P_{araBAD}]-B0030-mCherry^{N}(HGK).Ama-Ter^{N2}	pSB3T5	p15A	Tet
pFP.R407	[araC/P_{araBAD}]-B0030-mCherry^{N}(NGK).Cth-Ter^{N2}	pSB3T5	p15A	Tet
pFP.R408	[araC/P_{araBAD}]-B0030-mCherry^{N}(AKA).LLP-Pol^{N2}	pSB3T5	p15A	Tet
pFP.R409	[araC/P_{araBAD}]-B0030-mCherry^{N}(ATG).LP.Hel^{N2}	pSB3T5	p15A	Tet
pFP.R410	[araC/P_{araBAD}]-B0030-mCherry^{N}(QDQ).MP-B-DnaB^{N2}	pSB3T5	p15A	Tet
pFP.R412	[araC/P_{araBAD}]-B0030-mCherry^{N}(NGK).MP-M-DnaB^{N2}	pSB3T5	p15A	Tet
pFP.R413	[araC/P_{araBAD}]-B0030-mCherry^{N}(GKA).SaP-dpol^{N2}	pSB3T5	p15A	Tet
pFP.R415	[araC/P_{araBAD}]-B0030-mCherry^{N}(SNL).NrdA-2^{N2}	pSB3T5	p15A	Tet

pFP.R416	[araC/P$_{araBAD}$]-B0030-mCherry^{N}(HDG).Mja-KlbA^{N2}	pSB3T5	p15A	Tet
pFP.R287	[araC/P$_{araBAD}$]-B0030-mCherry^{N}(VDA).M86^{N3}	pSB3T5	p15A	Tet
pFP.R288	[araC/P$_{araBAD}$]-B0030-mCherry^{N}(GGK).NpuSsp^{N3}	pSB3T5	p15A	Tet
pFP.R289	[araC/P$_{araBAD}$]-B0030-mCherry^{N}(SGY).gp41-1^{N3}	pSB3T5	p15A	Tet
pFP.R290	[araC/P$_{araBAD}$]-B0030-mCherry^{N}(LNR).gp41-8^{N3}	pSB3T5	p15A	Tet
pFP.R291	[araC/P$_{araBAD}$]-B0030-mCherry^{N}(NPC).NrdJ-1^{N3}	pSB3T5	p15A	Tet
pFP.R292	[araC/P$_{araBAD}$]-B0030-mCherry^{N}(GGG).IMPDH-1^{N3}	pSB3T5	p15A	Tet
pFP.R293	[araC/P$_{araBAD}$]-B0030-mCherry^{N}(IDE).SspDnaX^{N3}	pSB3T5	p15A	Tet
pFP.R294	[araC/P$_{araBAD}$]-B0030-mCherry^{N}(AGG).SspGyrB^{N3}	pSB3T5	p15A	Tet
pFP.R295	[araC/P$_{araBAD}$]-B0030-mCherry^{N}(IGC).TerThyX^{N3}	pSB3T5	p15A	Tet
pFP.R296	[araC/P$_{araBAD}$]-B0030-mCherry^{N}(GSK).TvoVMA^{N3}	pSB3T5	p15A	Tet
pFP.R297	[araC/P$_{araBAD}$]-B0030-mCherry^{N}(SGK).PhoRadA^{N3}	pSB3T5	p15A	Tet
pFP.R459	[araC/P$_{araBAD}$]-B0030-mCherry^{N}(HGK).Ama-Ter^{N3*}	pSB3T5	p15A	Tet
pFP.R460	[araC/P$_{araBAD}$]-B0030-mCherry^{N}(NGK).Cth-Ter^{N3*}	pSB3T5	p15A	Tet
pFP.R461	[araC/P$_{araBAD}$]-B0030-mCherry^{N}(AKA).LLP-Pol^{N3*}	pSB3T5	p15A	Tet
pFP.R462	[araC/P$_{araBAD}$]-B0030-mCherry^{N}(ATG).LP.Hel^{N3*}	pSB3T5	p15A	Tet
pFP.R463	[araC/P$_{araBAD}$]-B0030-mCherry^{N}(QDQ).MP-B-DnaB^{N3*}	pSB3T5	p15A	Tet
pFP.R464	[araC/P$_{araBAD}$]-B0030-mCherry^{N}(NGK).MP-M-DnaB^{N3*}	pSB3T5	p15A	Tet
pFP.R465	[araC/P$_{araBAD}$]-B0030-mCherry^{N}(GKA).SaP-dpol^{N3*}	pSB3T5	p15A	Tet
pFP.R467	[araC/P$_{araBAD}$]-B0030-mCherry^{N}(SNL).NrdA-2^{N3*}	pSB3T5	p15A	Tet
pFP.R468	[araC/P$_{araBAD}$]-B0030-mCherry^{N}(HDG).Mja-KlbA^{N3*}	pSB3T5	p15A	Tet
pFP.R321	[araC/P$_{araBAD}$]-B0030-M86^{C1}.(SDL)mCherry^{C}.H$_{6}$	pSB1A3	pMB1	Amp
pFP.R322	[araC/P$_{araBAD}$]-B0030-NpuSsp^{C1}.(CWN)mCherry^{C}.H$_{6}$	pSB1A3	pMB1	Amp
pFP.R323	[araC/P$_{araBAD}$]-B0030-gp41-1^{C1}.(SSS)mCherry^{C}.H$_{6}$	pSB1A3	pMB1	Amp
pFP.R324	[araC/P$_{araBAD}$]-B0030-gp41-8^{C1}.(SAV)mCherry^{C}.H$_{6}$	pSB1A3	pMB1	Amp
pFP.R325	[araC/P$_{araBAD}$]-B0030-NrdJ-1^{C1}.(SEI)mCherry^{C}.H$_{6}$	pSB1A3	pMB1	Amp
pFP.R326	[araC/P$_{araBAD}$]-B0030-IMPDH-1^{C1}.(SIC)mCherry^{C}.H$_{6}$	pSB1A3	pMB1	Amp
pFP.R327	[araC/P$_{araBAD}$]-B0030-SspDnaX^{C1}.(CHT)mCherry^{C}.H$_{6}$	pSB1A3	pMB1	Amp
pFP.R328	[araC/P$_{araBAD}$]-B0030-SspGyrB^{C1}.(SAK)mCherry^{C}.H$_{6}$	pSB1A3	pMB1	Amp
pFP.R329	[araC/P$_{araBAD}$]-B0030-TerThyX^{C1}.(SFD)mCherry^{C}.H$_{6}$	pSB1A3	pMB1	Amp
pFP.R330	[araC/P$_{araBAD}$]-B0030-TvoVMA^{C1}.(TVI)mCherry^{C}.H$_{6}$	pSB1A3	pMB1	Amp
pFP.R331	[araC/P$_{araBAD}$]-B0030-PhoRadA^{C1}.(TQL)mCherry^{C}.H$_{6}$	pSB1A3	pMB1	Amp
pFP.R481	[araC/P$_{araBAD}$]-B0030-Ama-Ter^{C1}*.(SEL)mCherry^{C}.H$_{6}$	pSB1A3	pMB1	Amp
pFP.R482	[araC/P$_{araBAD}$]-B0030-Cth-Ter^{C1}*.(SEL)mCherry^{C}.H$_{6}$	pSB1A3	pMB1	Amp
pFP.R483	[araC/P$_{araBAD}$]-B0030-LLP-Pol^{C1}*.(CNF)mCherry^{C}.H$_{6}$	pSB1A3	pMB1	Amp
pFP.R484	[araC/P$_{araBAD}$]-B0030-LP.Hel^{C1}*.(CGK)mCherry^{C}.H$_{6}$	pSB1A3	pMB1	Amp
pFP.R485	[araC/P$_{araBAD}$]-B0030-MP-B-DnaB^{C1}*.(TKN)mCherry^{C}.H$_{6}$	pSB1A3	pMB1	Amp
pFP.R486	[araC/P$_{araBAD}$]-B0030-MP-M-DnaB^{C1}*.(TEL)mCherry^{C}.H$_{6}$	pSB1A3	pMB1	Amp
pFP.R487	[araC/P$_{araBAD}$]-B0030-SaP-dpol^{C1}*.(CEL)mCherry^{C}.H$_{6}$	pSB1A3	pMB1	Amp
pFP.R489	[araC/P$_{araBAD}$]-B0030-NrdA-2^{C1}*.(CNE)mCherry^{C}.H$_{6}$	pSB1A3	pMB1	Amp
pFP.R490	[araC/P$_{araBAD}$]-B0030-Mja-KlbA^{C1}*.(CSG)mCherry^{C}.H$_{6}$	pSB1A3	pMB1	Amp
pFP.R182	[araC/P$_{araBAD}$]-B0032-M86^{C2}.(SDL)mCherry^{C}.H$_{6}$	pSB1A3	pMB1	Amp
pFP.R243	[araC/P$_{araBAD}$]-B0030-NpuSsp^{C2}.(CWN)mCherry^{C}.H$_{6}$	pSB1A3	pMB1	Amp
pFP.R244	[araC/P$_{araBAD}$]-B0030-gp41-1^{C2}.(SSS)mCherry^{C}.H$_{6}$	pSB1A3	pMB1	Amp
pFP.R245	[araC/P$_{araBAD}$]-B0030-gp41-8^{C2}.(SAV)mCherry^{C}.H$_{6}$	pSB1A3	pMB1	Amp
pFP.R246	[araC/P$_{araBAD}$]-B0030-NrdJ-1^{C2}.(SEI)mCherry^{C}.H$_{6}$	pSB1A3	pMB1	Amp
pFP.R258	[araC/P$_{araBAD}$]-B0030-ST.IMPDH-1^{C2}.(SIC)mCherry^{C}.H$_{6}$	pSB1A3	pMB1	Amp
pFP.R259	[araC/P$_{araBAD}$]-B0030-ST.SspDnaX^{C2}.(CHT)mCherry^{C}.H$_{6}$	pSB1A3	pMB1	Amp
pFP.R188	[araC/P$_{araBAD}$]-B0032-SspGyrB^{C2}.(SAK)mCherry^{C}.H$_{6}$	pSB1A3	pMB1	Amp
pFP.R261	[araC/P$_{araBAD}$]-B0030-ST.TerThyX^{C2}.(SFD)mCherry^{C}.H$_{6}$	pSB1A3	pMB1	Amp
pFP.R445	[araC/P$_{araBAD}$]-B0030-TvoVMA^{C2}.(TVI)mCherry^{C}.H$_{6}$	pSB1A3	pMB1	Amp
pFP.R263	[araC/P$_{araBAD}$]-B0030-ST.PhoRadA^{C2}.(TQL)mCherry^{C}.H$_{6}$	pSB1A3	pMB1	Amp
pFP.R432	[araC/P$_{araBAD}$]-B0030-Ama-Ter^{C2}.(SEL)mCherry^{C}.H$_{6}$	pSB1A3	pMB1	Amp
pFP.R433	[araC/P$_{araBAD}$]-B0030-Cth-Ter^{C2}.(SEL)mCherry^{C}.H$_{6}$	pSB1A3	pMB1	Amp
pFP.R434	[araC/P$_{araBAD}$]-B0030-LLP-Pol^{C2}.(CNF)mCherry^{C}.H$_{6}$	pSB1A3	pMB1	Amp
pFP.R435	[araC/P$_{araBAD}$]-B0030-LP.Hel^{C2}.(CGK)mCherry^{C}.H$_{6}$	pSB1A3	pMB1	Amp
pFP.R436	[araC/P$_{araBAD}$]-B0030-MP-B-DnaB^{C2}.(TKN)mCherry^{C}.H$_{6}$	pSB1A3	pMB1	Amp
pFP.R438	[araC/P$_{araBAD}$]-B0030-MP-M-DnaB^{C2}.(TEL)mCherry^{C}.H$_{6}$	pSB1A3	pMB1	Amp

pFP.R439	[araC/P$_{araBAD}$]-B0030-SaP-dpol^{C2}.(CEL)mCherry^{C}.H$_{6}$	pSB1A3	pMB1	Amp
pFP.R441	[araC/P$_{araBAD}$]-B0030-NrdA-2^{C2}.(CNE)mCherry^{C}.H$_{6}$	pSB1A3	pMB1	Amp
pFP.R442	[araC/P$_{araBAD}$]-B0030-Mja-KlbA^{C2}.(CSG)mCherry^{C}.H$_{6}$	pSB1A3	pMB1	Amp
pFP.R332	[araC/P$_{araBAD}$]-B0030-M86^{C3}.(SDL)mCherry^{C}.H$_{6}$	pSB1A3	pMB1	Amp
pFP.R333	[araC/P$_{araBAD}$]-B0030-NpuSsp^{C3}.(CWN)mCherry^{C}.H$_{6}$	pSB1A3	pMB1	Amp
pFP.R334	[araC/P$_{araBAD}$]-B0030-gp41-1^{C3}.(SSS)mCherry^{C}.H$_{6}$	pSB1A3	pMB1	Amp
pFP.R335	[araC/P$_{araBAD}$]-B0030-gp41-8^{C3}.(SAV)mCherry^{C}.H$_{6}$	pSB1A3	pMB1	Amp
pFP.R336	[araC/P$_{araBAD}$]-B0030-NrdJ-1^{C3}.(SEI)mCherry^{C}.H$_{6}$	pSB1A3	pMB1	Amp
pFP.R337	[araC/P$_{araBAD}$]-B0030-IMPDH-1^{C3}.(SIC)mCherry^{C}.H$_{6}$	pSB1A3	pMB1	Amp
pFP.R338	[araC/P$_{araBAD}$]-B0030-SspDnaX^{C3}.(CHT)mCherry^{C}.H$_{6}$	pSB1A3	pMB1	Amp
pFP.R339	[araC/P$_{araBAD}$]-B0030-SspGyrB^{C3}.(SAK)mCherry^{C}.H$_{6}$	pSB1A3	pMB1	Amp
pFP.R340	[araC/P$_{araBAD}$]-B0030-TerThyX^{C3}.(SFD)mCherry^{C}.H$_{6}$	pSB1A3	pMB1	Amp
pFP.R341	[araC/P$_{araBAD}$]-B0030-TvoVMA^{C3}.(TVI)mCherry^{C}.H$_{6}$	pSB1A3	pMB1	Amp
pFP.R342	[araC/P$_{araBAD}$]-B0030-PhoRadA^{C3}.(TQL)mCherry^{C}.H$_{6}$	pSB1A3	pMB1	Amp
pFP.R492	[araC/P$_{araBAD}$]-B0030-Ama-Ter^{C3}.(SEL)mCherry^{C}.H$_{6}$	pSB1A3	pMB1	Amp
pFP.R493	[araC/P$_{araBAD}$]-B0030-Cth-Ter^{C3}.(SEL)mCherry^{C}.H$_{6}$	pSB1A3	pMB1	Amp
pFP.R494	[araC/P$_{araBAD}$]-B0030-LLP-Pol^{C3}.(CNF)mCherry^{C}.H$_{6}$	pSB1A3	pMB1	Amp
pFP.R495	[araC/P$_{araBAD}$]-B0030-LP.Hel^{C3}.(CGK)mCherry^{C}.H$_{6}$	pSB1A3	pMB1	Amp
pFP.R496	[araC/P$_{araBAD}$]-B0030-MP-B-DnaB^{C3}.(TKN)mCherry^{C}.H$_{6}$	pSB1A3	pMB1	Amp
pFP.R497	[araC/P$_{araBAD}$]-B0030-MP-M-DnaB^{C3}.(TEL)mCherry^{C}.H$_{6}$	pSB1A3	pMB1	Amp
pFP.R498	[araC/P$_{araBAD}$]-B0030-SaP-dpol^{C3}.(CEL)mCherry^{C}.H$_{6}$	pSB1A3	pMB1	Amp
pFP.R500	[araC/P$_{araBAD}$]-B0030-NrdA-2^{C3}.(CNE)mCherry^{C}.H$_{6}$	pSB1A3	pMB1	Amp
pFP.R501	[araC/P$_{araBAD}$]-B0030-Mja-KlbA^{C3}.(CSG)mCherry^{C}.H$_{6}$	pSB1A3	pMB1	Amp
pFP.R504	[araC/P$_{araBAD}$]-B0030-mCherry^{N}(GGK).NpuSsp^{N3}*	pSB3T5	p15A	Tet
pFP.R506	[araC/P$_{araBAD}$]-B0030-NpuSsp^{C1}*.(CWN)mCherry^{C}.H$_{6}$	pSB1A3	pMB1	Amp
pFP.R505	[araC/P$_{araBAD}$]-B0030-mCherry^{N}(IGC).TerThyX^{N3}*	pSB3T5	p15A	Tet
pFP.R507	[araC/P$_{araBAD}$]-B0030-TerThyX^{C1}*.(SFD)mCherry^{C}.H$_{6}$	pSB1A3	pMB1	Amp
pFP.R541	[lacI/P$_{T7.lacO}$]-B0030-ST.M86^{C2}.(SDL)mCherry^{C}.H$_{6}$	pET11	pBR322	Amp
pFP.R542	[lacI/P$_{T7.lacO}$]-B0030-ST.NpuSsp^{C2}.(CWN)mCherry^{C}.H$_{6}$	pET11	pBR322	Amp
pFP.R517	[rhaS/P$_{rhaBAD}$]-B0032-M86^{C2}.(SDL)mCherry^{C}.H$_{6}$	pSB1A3	pMB1	Amp
pFP.R518	[rhaS/P$_{rhaBAD}$]-B0030-gp41-1^{C2}.(SSS)mCherry^{C}.H$_{6}$	pSB1A3	pMB1	Amp
pFP.R519	[rhaS/P$_{rhaBAD}$]-B0030-gp41-8^{C2}.(SAV)mCherry^{C}.H$_{6}$	pSB1A3	pMB1	Amp
pFP.R684	[rhaS/P$_{rhaBAD}$]-B0030-NrdJ-1^{C2}.(SEI)mCherry^{C}.H$_{6}$	pSB1A3	pMB1	Amp
pFP.R520	[rhaS/P$_{rhaBAD}$]-B0032-SspGyrB^{C2}.(SAK)mCherry^{C}.H$_{6}$	pSB1A3	pMB1	Amp
pFP.R521	[rhaS/P$_{rhaBAD}$]-B0030-Cth-Ter^{C2}.(SEL)mCherry^{C}.H$_{6}$	pSB1A3	pMB1	Amp
pFP.R523	[rhaS/P$_{rhaBAD}$]-B0030-Mja-KlbA^{C2}.(CSG)mCherry^{C}.H$_{6}$	pSB1A3	pMB1	Amp
pFP.R685	[rhaS/P$_{rhaBAD}$]-B0032-gp41-1^{C2}.(SSS)mCherry^{C}.H$_{6}$	pSB1A3	pMB1	Amp
pFP.R688	[rhaS/P$_{rhaBAD}$]-B0032-NrdJ-1^{C2}.(SEI)mCherry^{C}.H$_{6}$	pSB1A3	pMB1	Amp
pFP.R686	[rhaS/P$_{rhaBAD}$]-B0032-Cth-Ter^{C2}.(SEL)mCherry^{C}.H$_{6}$	pSB1A3	pMB1	Amp
pFP.E98	[araC/P$_{araBAD}$]-B0033-ecf16^{N}	pSB3T5	p15A	Tet
pFP.E36	[araC/P$_{araBAD}$]-B0030-ecf17^{N}	pSB3T5	p15A	Tet
pFP.E99	[araC/P$_{araBAD}$]-B0033-ecf20^{N}	pSB3T5	p15A	Tet
pFP.E61	[rhaS/P$_{rhaBAD}$]-B0030-ecf16^{C}	pSEVA221	RK2	Kan
pFP.E62	[rhaS/P$_{rhaBAD}$]-B0030-ecf17^{C}	pSEVA221	RK2	Kan
pFP.E63	[rhaS/P$_{rhaBAD}$]-B0030-ecf20^{C}	pSEVA221	RK2	Kan
pFP.P16	P$_{16}$-B0030-gfp-B0015	pSB4A3	pSC101	Amp
pFP.P17	P$_{17}$-B0030-gfp-B0015	pSB4A3	pSC101	Amp
pFP.P20	P$_{20}$-B0030-gfp-B0015	pSB4A3	pSC101	Amp
pFP.E78	[araC/P$_{araBAD}$]-B0033-ecf16	pSB3T5	p15A	Tet
pFP.E274	[araC/P$_{araBAD}$]-B0033-ecf16^{N}(VDASDL)ecf16^{C}	pSB3T5	p15A	Tet
pFP.E276	[araC/P$_{araBAD}$]-B0033-ecf16^{N}(SGYSSS)ecf16^{C}	pSB3T5	p15A	Tet
pFP.E277	[araC/P$_{araBAD}$]-B0033-ecf16^{N}(LNRSAV)ecf16^{C}	pSB3T5	p15A	Tet
pFP.E155	[araC/P$_{araBAD}$]-B0033-ecf16^{N}(NPCSEI)ecf16^{C}	pSB3T5	p15A	Tet
pFP.E164	[araC/P$_{araBAD}$]-B0033-ecf16^{N}(AGGSAK)ecf16^{C}	pSB3T5	p15A	Tet
pFP.E283	[araC/P$_{araBAD}$]-B0033-ecf16^{N}(NGKSEL)ecf16^{C}	pSB3T5	p15A	Tet
pFP.E181	[araC/P$_{araBAD}$]-B0033-ecf16^{N}(HDGCSG)ecf16^{C}	pSB3T5	p15A	Tet
pFP.ECF17	[araC/P$_{araBAD}$]-B0030-ecf17	pSB3T5	p15A	Tet
pFP.E291	[araC/P$_{araBAD}$]-B0030-ecf17^{N}(VDASDL)ecf17^{C}	pSB3T5	p15A	Tet

pFP.E293	[araC/P$_{araBAD}$]-B0030-ecf17^{N}(SGYSSS)ecf17^{C}	pSB3T5	p15A	Tet
pFP.E294	[araC/P$_{araBAD}$]-B0030-ecf17^{N}(LNRSAV)ecf17^{C}	pSB3T5	p15A	Tet
pFP.E156	[araC/P$_{araBAD}$]-B0030-ecf17^{N}(NPCSEI)ecf17^{C}	pSB3T5	p15A	Tet
pFP.E165	[araC/P$_{araBAD}$]-B0030-ecf17^{N}(AGGSAK)ecf17^{C}	pSB3T5	p15A	Tet
pFP.E300	[araC/P$_{araBAD}$]-B0030-ecf17^{N}(NGKSEL)ecf17^{C}	pSB3T5	p15A	Tet
pFP.E214	[araC/P$_{araBAD}$]-B0030-ecf17^{N}(HDGCSG)ecf17^{C}	pSB3T5	p15A	Tet
pFP.E80	[araC/P$_{araBAD}$]-B0033-ecf20	pSB3T5	p15A	Tet
pFP.E87	araC/P$_{araBAD}$]-B0033-ecf20^{N}(VDASDL)ecf20^{C}	pSB3T5	p15A	Tet
pFP.E89	araC/P$_{araBAD}$]-B0033-ecf20^{N}(SGYSSS)ecf20^{C}	pSB3T5	p15A	Tet
pFP.E90	araC/P$_{araBAD}$]-B0033-ecf20^{N}(LNRSAV)ecf20^{C}	pSB3T5	p15A	Tet
pFP.E91	araC/P$_{araBAD}$]-B0033-ecf20^{N}(NPCSEI)ecf20^{C}	pSB3T5	p15A	Tet
pFP.E94	araC/P$_{araBAD}$]-B0033-ecf20^{N}(AGGSAK)ecf20^{C}	pSB3T5	p15A	Tet
pFP.E230	araC/P$_{araBAD}$]-B0033-ecf20^{N}(NGKSEL)ecf20^{C}	pSB3T5	p15A	Tet
pFP.E182	araC/P$_{araBAD}$]-B0033-ecf20^{N}(HDGCSG)ecf20^{C}	pSB3T5	p15A	Tet
pFP.E199	P$_{16}$-B0030-mCherry-B0015	pSB3T5	p15A	Tet
pFP.E200	P$_{17}$-B0030-bfp-B0015	pSB1A3	pMB1	Amp
pFP.E216	P$_{16}$-B0030-mCherry-B0015-P$_{17}$-B0030-bfp-B0015-P$_{20}$-B0030-gfp-B0015	pSB4A3	pSC101	Amp
pFP.E227	i[P$_{16}$-B0030-mCherry]-B0015-P$_{17}$-B0030-bfp-B0015-P$_{20}$-B0030-gfp-B0015	pSB4A3	pSC101	Amp
pFP.E195	[araC/P$_{araBAD}$]-B0033-ecf16^{N}(AGG).SspGyrB^{N2}	pSB3T5	p15A	Tet
pFP.E196	[rhaS/P$_{rhaBAD}$]-B0032-SspGyrB^{C2}.(SAK)ecf16^{C}	pSEVA221	RK2	Kan
pFP.E197	[araC/P$_{araBAD}$]-B0030-ecf17^{N}(NPC).NrdJ-1^{N2}	pSB3T5	p15A	Tet
pFP.E223	[rhaS/P$_{rhaBAD}$]-B0032-NrdJ-1^{C2}.(SEI)ecf17^{C}	pSEVA221	RK2	Kan
pFP.E124	[araC/P$_{araBAD}$]-B0033-ecf20^{N}(VDA).M86^{N2}	pSB3T5	p15A	Tet
pFP.E144	[rhaS/P$_{rhaBAD}$]-B0032-M86^{C2}.(SDL)ecf20^{C}	pSEVA221	RK2	Kan
pFP.E609	[araC/P$_{araBAD}$]-B0033-ecf16^{N}(AGG).SspGyrB^{N2}(C>A)	pSB3T5	p15A	Tet
pFP.E610	[rhaS/P$_{rhaBAD}$]-B0032-SspGyrB^{C2}(N>A).(SAK)ecf16^{C}	pSEVA221	RK2	Kan
pFP.E611	[araC/P$_{araBAD}$]-B0030-ecf17^{N}(NPC).NrdJ-1^{N2}(C>A)	pSB3T5	p15A	Tet
pFP.E612	[rhaS/P$_{rhaBAD}$]-B0032-NrdJ-1^{C2}(N>A).(SEI)ecf17^{C}	pSEVA221	RK2	Kan
pFP.E613	[araC/P$_{araBAD}$]-B0033-ecf20^{N}(VDA).M86^{N2}(C>A)	pSB3T5	p15A	Tet
pFP.E614	[rhaS/P$_{rhaBAD}$]-B0032-M86^{C2}(N>A).(SDL)ecf20^{C}	pSEVA221	RK2	Kan
pFP.E211	[araC/P$_{araBAD}$]-B0033-ecf20^{N}(VDA).M86^{N2}-L3S2P21-P$_{araBAD}$-B0032-SspGyrB^{C2}.(SAK)ecf16^{C}	pSB3T5	p15A	Tet
pFP.B15-E212	B0015-[rhaS/P$_{rhaBAD}$]-B0030-ecf17^{N}(NPC).NrdJ-1^{N2}-ECK120033737-P$_{rhaBAD}$-B0032-M86^{C2}.(SDL)ecf20^{C}	pSB1A3	pMB1	Amp
pFP.E220	B0015-[luxR/P$_{lux2}$]-B0033-ecf16^{N}(AGG).SspGyrB^{N2}-L3S2P21-P$_{lux2}$-B0033-NrdJ-1^{C2}.(SEI)ecf17^{C}	pSB3K3	p15A	Kan
pFP.E222	[araC/P$_{araBAD}$]-B0033-ecf20^{N}(VDA).M86^{N2}-L3S2P21-P$_{araBAD}$-B0032-SspGyrB^{C2}.(SAK)ecf16^{C}-B0015-[rhaS/P$_{rhaBAD}$]-B0030-ecf17^{N}(NPC).NrdJ-1^{N2}-ECK120033737-P$_{rhaBAD}$-B0032-M86^{C2}.ecf20^{C}-B0015-[luxR/P$_{lux2}$]-B0033-ecf16^{N}(AGG).SspGyrB^{N2}-L3S2P21-P$_{lux2}$-B0033-NrdJ-1^{C2}.(SEI)ecf17^{C}	pSB3K3	p15A	Kan
pET.SasG3	[lacI/P$_{T7.lacO}$]-B0030-H$_{6}$.sasG5^{3}E^{2}	pET11	pBR322	Amp
pET.SasG6_J	[lacI/P$_{T7.lacO}$]-B0030-H$_{6}$.sasG5^{6}E^{4}	pET11	pBR322	Amp
pET.SasG9_J	[lacI/P$_{T7.lacO}$]-B0030-H$_{6}$.sasG5^{9}E^{6}	pET11	pBR322	Amp
pET.SasG12_J	[lacI/P$_{T7.lacO}$]-B0030-H$_{6}$.sasG5^{12}E^{8}	pET11	pBR322	Amp
pET.SasG3_1	[lacI/P$_{T7.lacO}$]-B0030-Strep.sasG5^{3}E^{2}.NrdJ-1^{N2}	pET11	pBR322	Amp
pET.SasG3_2	[lacI/P$_{T7.lacO}$]-B0030-NrdJ-1^{C2}.sasG5^{3}E^{2}.gp41-1^{N2}	pET11	pBR322	Amp
pET.SasG3_3	[lacI/P$_{T7.lacO}$]-B0030-gp41-1^{C2}.sasG5^{3}E^{2}.IMPDH-1^{N2}	pET11	pBR322	Amp
pET.SasG3_4	[lacI/P$_{T7.lacO}$]-B0030-ST.IMPDH-1^{C2}.sasG5^{3}E^{2}.SspGyrB^{N2}	pET11	pBR322	Amp
pET.SasG3_5	[lacI/P$_{T7.lacO}$]-B0030-SspGyrB^{C2}.sasG5^{3}E^{2}.gp41-8^{N2}	pET11	pBR322	Amp
pET.SasG3_6	[lacI/P$_{T7.lacO}$]-B0030-gp41-8^{C2}.sasG5^{3}E^{2}.H$_{6}$	pET11	pBR322	Amp
pET.SasG3_7	[lacI/P$_{T7.lacO}$]-B0030-gp41-1^{C2}.sasG5^{3}E^{2}.NrdJ-1^{N2}	pET11	pBR322	Amp
pET.SasG3_8	[lacI/P$_{T7.lacO}$]-B0030-NrdJ-1^{C2}.sasG5^{3}E^{2}.H$_{6}$	pET11	pBR322	Amp

[a] *- intein segment with flexible linker at the canonical split site; ST - solubility/expressivity tag; H$_{6}$- hexahistidine tag; Strep- strep tag; i - reverse orientation.

[b] Tet - Tetracycline; Amp - Ampicillin; Kan - Kanamycin.

Supplementary Table 9 | Theoretical molecular weights of proteins analyzed by SDS-PAGE and Western blots

Inteins related proteins			
**mCherry controls**		**mCherry with intein insertions *in cis***	
Protein name[a]	Molecular weight (kDa)	Protein name[a]	Molecular weight (kDa)
mCherry-H$_{6}$	27.5		
mCherry^{N}(VDASDL)mCherry^{C}-H$_{6}$	28.1	mCherry^{N}(VDA).M86.(SDL)mCherry^{C}-H$_{6}$	45.5
mCherry^{N}(GGKCWN)mCherry^{C}-H$_{6}$	28.2	mCherry^{N}(GGK).NpuSsp.(CWN)mCherry^{C}-H$_{6}$	43.9
		mCherry^{N}(GGK).NpuSsp*.(CWN)mCherry^{C}-H$_{6}$	44.5
mCherry^{N}(SGYSSS)mCherry^{C}-H$_{6}$	28.1	mCherry^{N}(SGY).gp41-1.(SSS)mCherry^{C}-H$_{6}$	42.3
mCherry^{N}(LNRSAV)mCherry^{C}-H$_{6}$	28.2	mCherry^{N}(LNR).gp41-8.(SAV)mCherry^{C}-H$_{6}$	43.2
mCherry^{N}(NPCSEI)mCherry^{C}-H$_{6}$	28.2	mCherry^{N}(NPC).NrdJ-1.(SEI)mCherry^{C}-H$_{6}$	44.6
mCherry^{N}(GGGSIC)mCherry^{C}-H$_{6}$	28.0	mCherry^{N}(GGG).IMPDH-1.(SIC)mCherry^{C}-H$_{6}$	44.4
mCherry^{N}(IDECHT)mCherry^{C}-H$_{6}$	28.2	mCherry^{N}(IDE).SspDnaX.(CHT)mCherry^{C}-H$_{6}$	43.6
mCherry^{N}(AGGSAK)mCherry^{C}-H$_{6}$	28.0	mCherry^{N}(AGG).SspGyrB.(SAK)mCherry^{C}-H$_{6}$	45.5
mCherry^{N}(IGCSFD)mCherry^{C}-H$_{6}$	28.2	mCherry^{N}(IGC).TerThyX.(SFD)mCherry^{C}-H$_{6}$	47.3
		mCherry^{N}(IGC).TerThyX*.(SFD)mCherry^{C}-H$_{6}$	48.0
mCherry^{N}(GSKTVI)mCherry^{C}-H$_{6}$	28.1	mCherry^{N}(GSK).TvoVMA.(TVI)mCherry^{C}-H$_{6}$	48.7
mCherry^{N}(SGKTQL)mCherry^{C}-H$_{6}$	28.2	mCherry^{N}(SGK).PhoRadA.(TQL)mCherry^{C}-H$_{6}$	47.9
mCherry^{N}(VNQSGR)mCherry^{C}-H$_{6}$	28.2	mCherry^{N}(VNQ).Cro-RIR1*.(SGR)mCherry^{C}-H$_{6}$	46.6
mCherry^{N}(DKFSTK)mCherry^{C}-H$_{6}$	28.2	mCherry^{N}(DKF).Cro-RPB2*.(STK)mCherry^{C}-H$_{6}$	46.0
mCherry^{N}(GSKCSN)mCherry^{C}-H$_{6}$	28.1	mCherry^{N}(GSK).Cro-Top2*.(CSN)mCherry^{C}-H$_{6}$	47.2
mCherry^{N}(SNLCSE)mCherry^{C}-H$_{6}$	28.2	mCherry^{N}(SNL).CIV-RIR1*.(CSE)mCherry^{C}-H$_{6}$	45.7
		mCherry^{N}(SNL).BsuP-RIR1*.(CSE)mCherry^{C}-H$_{6}$	45.6
mCherry^{N}(KHHSVL)mCherry^{C}-H$_{6}$	28.2	mCherry^{N}(KHH).CP-Thy1*.(SVL)mCherry^{C}-H$_{6}$	45.5
mCherry^{N}(HGKSEL)mCherry^{C}-H$_{6}$	28.2	mCherry^{N}(HGK).Ama-Ter*.(SEL)mCherry^{C}-H$_{6}$	46.1
mCherry^{N}(ASCCRL)mCherry^{C}-H$_{6}$	28.2	mCherry^{N}(ASC).CbP-RNR*.(CRL)mCherry^{C}-H$_{6}$	46.6
mCherry^{N}(NGKTTL)mCherry^{C}-H$_{6}$	28.2	mCherry^{N}(NGK).Ckl-Ter*.(TTL)mCherry^{C}-H$_{6}$	48.6
mCherry^{N}(NGKSEL)mCherry^{C}-H$_{6}$	28.2	mCherry^{N}(NGK).Cth-Ter*.(SEL)mCherry^{C}-H$_{6}$	46.0
mCherry^{N}(HGKTEV)mCherry^{C}-H$_{6}$	28.2	mCherry^{N}(HGK).EP-Pri*.(TEV)mCherry^{C}-H$_{6}$	45.4
mCherry^{N}(AKACNF)mCherry^{C}-H$_{6}$	28.2	mCherry^{N}(AKA).LLP-Pol*.(CNF)mCherry^{C}-H$_{6}$	46.8
mCherry^{N}(ATGCGK)mCherry^{C}-H$_{6}$	28.1	mCherry^{N}(ATG).LP-Hel*.(CGK)mCherry^{C}-H$_{6}$	45.1
mCherry^{N}(QDQTKN)mCherry^{C}-H$_{6}$	28.2	mCherry^{N}(QDQ).MP-B-DnaB*.(TKN)mCherry^{C}-H$_{6}$	45.4
mCherry^{N}(NHDSRA)mCherry^{C}-H$_{6}$	28.2	mCherry^{N}(NHD).MP-K-gp53*.(SRA)mCherry^{C}-H$_{6}$	44.7
mCherry^{N}(ELKTQN)mCherry^{C}-H$_{6}$	28.2	mCherry^{N}(ELK).MP-C-gp206*.(TQN)mCherry^{C}-H$_{6}$	45.1
mCherry^{N}(NGKTEL)mCherry^{C}-H$_{6}$	28.2	mCherry^{N}(NGK).MP-M-DnaB*.(TEL)mCherry^{C}-H$_{6}$	45.0
mCherry^{N}(AGKSLT)mCherry^{C}-H$_{6}$	28.1	mCherry^{N}(AGK).PP-Phi*.(SLT)mCherry^{C}-H$_{6}$	47.0
mCherry^{N}(GKACEL)mCherry^{C}-H$_{6}$	28.1	mCherry^{N}(GKA).SaP-dpol*.(CEL)mCherry^{C}-H$_{6}$	46.0
mCherry^{N}(SGKTSS)mCherry^{C}-H$_{6}$	28.1	mCherry^{N}(SGK).SaP-Hel*.(TSS)mCherry^{C}-H$_{6}$	46.1
mCherry^{N}(SNLCNE)mCherry^{C}-H$_{6}$	28.2	mCherry^{N}(SNL).NrdA-2*.(CNE)mCherry^{C}-H$_{6}$	44.6
mCherry^{N}(GGGTGL)mCherry^{C}-H$_{6}$	28.0	mCherry^{N}(GGG).Pfu-RIR1-1*.(TGL)mCherry^{C}-H$_{6}$	48.7
mCherry^{N}(HDGCSG)mCherry^{C}-H$_{6}$	28.1	mCherry^{N}(HDG).Mja-KlbA*.(CSG)mCherry^{C}-H$_{6}$	48.2

Split Inteins related proteins			
**N-terminal**		**C-terminal**	
Protein name[a]	Molecular weight (kDa)	Protein name[a]	Molecular weight (kDa)
mCherry^{N}	18.0	mCherry^{C}-H$_{6}$	9.7
mCherry^{N}(SGY)	18.3	(SSS)mCherry^{C}-H$_{6}$	9.9
mCherry^{N}(VDA).M86^{N1}	19.6	M86^{C1}.(SDL)mCherry^{C}-H$_{6}$	26.0
mCherry^{N}(GGK).NpuSsp^{N1}	19.8	NpuSsp^{C1}.(CWN)mCherry^{C}-H$_{6}$	24.3
		NpuSsp^{C1}*.(CWN)mCherry^{C}-H$_{6}$	24.8
mCherry^{N}(SGY).gp41-1^{N1}	19.7	gp41-1^{C1}.(SSS)mCherry^{C}-H$_{6}$	22.7
mCherry^{N}(LNR).gp41-8^{N1}	19.6	gp41-8^{C1}.(SAV)mCherry^{C}-H$_{6}$	23.7
mCherry^{N}(NPC).NrdJ-1^{N1}	19.8	NrdJ-1^{C1}.(SEI)mCherry^{C}-H$_{6}$	25.0
mCherry^{N}(GGG).IMPDH-1^{N1}	19.5	IMPDH-1^{C1}.(SIC)mCherry^{C}-H$_{6}$	25.0
mCherry^{N}(IDE).SspDnaX^{N1}	19.7	SspDnaX^{C1}.(CHT)mCherry^{C}-H$_{6}$	24.1
mCherry^{N}(AGG).SspGyrB^{N1}	19.4	SspGyrB^{C1}.(SAK)mCherry^{C}-H$_{6}$	26.2
mCherry^{N}(IGC).TerThyX^{N1}	19.8	TerThyX^{C1}.(SFD)mCherry^{C}-H$_{6}$	27.6
		TerThyX^{C1}.(SFD)mCherry^{C}-H$_{6}$*	28.3
mCherry^{N}(GSK).TvoVMA^{N1}	19.5	TvoVMA^{C1}.(TVI)mCherry^{C}-H$_{6}$	29.3
mCherry^{N}(SGK).PhoRadA^{N1}	19.9	PhoRadA^{C1}.(TQL)mCherry^{C}-H$_{6}$	28.1

mCherry^{N}(HGK).Ama-Ter^{N1}	19.7	Ama-Ter^{C1*}.(SEL)mCherry^{C}-H$_6$	26.6
mCherry^{N}(NGK).Cth-Ter^{N1}	19.6	Cth-Ter^{C1*}.(SEL)mCherry^{C}-H$_6$	26.5
mCherry^{N}(AKA).LLP-Pol^{N1}	19.6	LLP-Pol^{C1*}.(CNF)mCherry^{C}-H$_6$	27.3
mCherry^{N}(ATG).LP-Hel^{N1}	19.6	LP-Hel^{C1*}.(CGK)mCherry^{C}-H$_6$	25.7
mCherry^{N}(QDQ).MP-B-DnaB^{N1}	19.6	MP-B-DnaB^{C1*}.(TKN)mCherry^{C}-H$_6$	25.9
mCherry^{N}(NGK).MP-M-DnaB^{N1}	19.6	MP-M-DnaB^{C1*}.(TEL)mCherry^{C}-H$_6$	25.6
mCherry^{N}(GKA).SaP-dpol^{N1}	19.6	SaP-dpol^{C1*}.(CEL)mCherry^{C}-H$_6$	26.4
mCherry^{N}(SNL).NrdA-2^{N1}	19.6	NrdA-2^{C1*}.(CNE)mCherry^{C}-H$_6$	25.1
mCherry^{N}(HDG).Mja-KlbA^{N1}	19.7	Mja-KlbA^{C1*}.(CSG)mCherry^{C}-H$_6$	28.7
mCherry^{N}(VDA).M86^{N2}	29.6	M86^{C2}.(SDL)mCherry^{C}-H$_6$	16.0
mCherry^{N}(GGK).NpuSsp^{N2}	30.1	NpuSsp^{C2}.(CWN)mCherry^{C}-H$_6$	13.9
mCherry^{N}(SGY).gp41-1^{N2}	28.1	gp41-1^{C2}.(SSS)mCherry^{C}-H$_6$	14.0
mCherry^{N}(LNR).gp41-8^{N2}	28.4	gp41-8^{C2}.(SAV)mCherry^{C}-H$_6$	15.0
mCherry^{N}(NPC).NrdJ-1^{N2}	30.2	NrdJ-1^{C2}.(SEI)mCherry^{C}-H$_6$	14.5
mCherry^{N}(GGG).IMPDH-1^{N2}	29.9	ST.IMPDH-1^{C2}.(SIC)mCherry^{C}-H$_6$	15.2
mCherry^{N}(IDE).SspDnaX^{N2}	29.0	ST.SspDnaX^{C2}.(CHT)mCherry^{C}-H$_6$	15.4
mCherry^{N}(AGG).SspGyrB^{N2}	30.8	SspGyrB^{C2}.(SAK)mCherry^{C}-H$_6$	14.8
mCherry^{N}(IGC).TerThyX^{N2}	33.1	ST.TerThyX^{C2}.(SFD)mCherry^{C}-H$_6$	15.0
mCherry^{N}(GSK).TvoVMA^{N2}	33.1	TvoVMA^{C2}.(TVI)mCherry^{C}-H$_6$	15.6
mCherry^{N}(SGK).PhoRadA^{N2}	32.7	ST.PhoRadA^{C2}.(TQL)mCherry^{C}-H$_6$	16.0
mCherry^{N}(HGK).Ama-Ter^{N2}	31.0	Ama-Ter^{C2}.(SEL)mCherry^{C}-H$_6$	14.6
mCherry^{N}(NGK).Cth-Ter^{N2}	30.8	Cth-Ter^{C2}.(SEL)mCherry^{C}-H$_6$	14.6
mCherry^{N}(AKA).LLP-Pol^{N2}	31.9	LLP-Pol^{C2}.(CNF)mCherry^{C}-H$_6$	14.4
mCherry^{N}(ATG).LP-Hel^{N2}	30.3	LP-Hel^{C2}.(CGK)mCherry^{C}-H$_6$	14.2
mCherry^{N}(QDQ).MP-B-DnaB^{N2}	30.3	MP-B-DnaB^{C2}.(TKN)mCherry^{C}-H$_6$	14.6
mCherry^{N}(NGK).MP-M-DnaB^{N2}	30.2	MP-M-DnaB^{C2}.(TEL)mCherry^{C}-H$_6$	14.3
mCherry^{N}(GKA).SaP-dpol^{N2}	31.3	SaP-dpol^{C2}.(CEL)mCherry^{C}-H$_6$	14.1
mCherry^{N}(SNL).NrdA-2^{N2}	30.2	NrdA-2^{C2}.(CNE)mCherry^{C}-H$_6$	13.8
mCherry^{N}(HDG).Mja-KlbA^{N2}	33.2	Mja-KlbA^{C2}.(CSG)mCherry^{C}-H$_6$	14.5
mCherry^{N}(VDA).M86^{N3}	34.1	M86^{C3}.(SDL)mCherry^{C}-H$_6$	11.5
mCherry^{N}(GGK).NpuSsp^{N3}	32.6	NpuSsp^{C3}.(CWN)mCherry^{C}-H$_6$	11.5
mCherry^{N}(GGK).NpuSsp^{N3*}	33.2		
mCherry^{N}(SGY).gp41-1^{N3}	31.0	gp41-1^{C3}.(SSS)mCherry^{C}-H$_6$	11.5
mCherry^{N}(LNR).gp41-8^{N3}	31.9	gp41-8^{C3}.(SAV)mCherry^{C}-H$_6$	11.4
mCherry^{N}(NPC).NrdJ-1^{N3}	33.3	NrdJ-1^{C3}.(SEI)mCherry^{C}-H$_6$	11.5
mCherry^{N}(GGG).IMPDH-1^{N3}	33.1	IMPDH-1^{C3}.(SIC)mCherry^{C}-H$_6$	11.4
mCherry^{N}(IDE).SspDnaX^{N3}	32.3	SspDnaX^{C3}.(CHT)mCherry^{C}-H$_6$	11.4
mCherry^{N}(AGG).SspGyrB^{N3}	34.2	SspGyrB^{C3}.(SAK)mCherry^{C}-H$_6$	11.4
mCherry^{N}(IGC).TerThyX^{N3}	35.9	TerThyX^{C3}.(SFD)mCherry^{C}-H$_6$	11.6
mCherry^{N}(IGC).TerThyX^{N3*}	36.6		
mCherry^{N}(GSK).TvoVMA^{N3}	37.1	TvoVMA^{C3}.(TVI)mCherry^{C}-H$_6$	11.8
mCherry^{N}(SGK).PhoRadA^{N3}	36.5	PhoRadA^{C3}.(TQL)mCherry^{C}-H$_6$	11.5
mCherry^{N}(HGK).Ama-Ter^{N3*}	34.5	Ama-Ter^{C3}.(SEL)mCherry^{C}-H$_6$	11.8
mCherry^{N}(NGK).Cth-Ter^{N3*}	34.3	Cth-Ter^{C3}.(SEL)mCherry^{C}-H$_6$	11.8
mCherry^{N}(AKA).LLP-Pol^{N3*}	35.0	LLP-Pol^{C3}.(CNF)mCherry^{C}-H$_6$	12.0
mCherry^{N}(ATG).LP-Hel^{N3*}	33.6	LP-Hel^{C3}.(CGK)mCherry^{C}-H$_6$	11.7
mCherry^{N}(QDQ).MP-B-DnaB^{N3*}	33.8	MP-B-DnaB^{C3}.(TKN)mCherry^{C}-H$_6$	11.8
mCherry^{N}(NGK).MP-M-DnaB^{N3*}	33.6	MP-M-DnaB^{C3}.(TEL)mCherry^{C}-H$_6$	11.6
mCherry^{N}(GKA).SaP-dpol^{N3*}	34.3	SaP-dpol^{C3}.(CEL)mCherry^{C}-H$_6$	11.8
mCherry^{N}(SNL).NrdA-2^{N3*}	33.2	NrdA-2^{C3}.(CNE)mCherry^{C}-H$_6$	11.5
mCherry^{N}(HDG).Mja-KlbA^{N3*}	36.8	Mja-KlbA^{C3}.(CSG)mCherry^{C}-H$_6$	11.6

SasG related proteins

Full-length controls		Assembly units	
Protein name[a]	Molecular weight (kDa)	Protein name[a]	Molecular weight (kDa)
H$_6$.sasG5^{3}E^{2}	37.8	Strep.sasG5^{3}E^{2}.NrdJ-1^{N2}	50.4
H$_6$.sasG5^{6}E^{4}	75.2	NrdJ-1^{C2}.sasG5^{3}E^{2}.gp41-1^{N2}	51.9
H$_6$.sasG5^{9}E^{6}	112.6	gp41-1^{C2}.sasG5^{3}E^{2}.IMPDH-1^{N2}	53.2
H$_6$.sasG5^{12}E^{8}	149.9	ST.IMPDH-1^{C2}.sasG5^{3}E^{2}.SspGyrB^{N2}	55.2
		SspGyrB^{C2}.sasG5^{3}E^{2}.gp41-8^{N2}	52.2
		gp41-8^{C2}.sasG5^{3}E^{2}.H$_6$	43.1
		gp41-1^{C2}.sasG5^{3}E^{2}.NrdJ-1^{N2}	53.5
		NrdJ-1^{C2}.sasG5^{3}E^{2}.H$_6$	48.6

[a] * - Intein segment with flexible linker at the canonical split site

Supplementary Note 1 | Methods for constructing the plasmids used in this work

PCR amplifications were performed using 1 μL DNA template (10-50 ng), 4 μL of 5X Phusion GC Buffer, 0.4 μL of 10 mM dNTPs (PB10.71, PCR Biosystems), 0.2 μL of Phusion DNA Polymerase (2 U μL^{-1}), 0.25 μL of forward primer (10 μM) and 0.25 μL of reverse primer (10 μM), filled up to a total volume of 20 μL with autoclaved ddH_2O. Annealing temperatures (T_a) were calculated using Thermo Fisher Scientific's Tm Calculator online resource (https://www.thermofisher.com/). For amplification, a 30-40 cycles (step 2-4) PCR program with 3 min at 98 °C, 10 s at 90 °C, 15 s at T_a, 30 s per kb at 72 °C and 10 min at 72 °C was generally used. Successful DNA amplification and specificity was assessed by gel electrophoresis, analyzing 1 μL of the reactions on 0.8-1% agarose gels. All PCR products were treated with FastDigest *Dpn*I (FD1704, Thermo Fisher Scientific), by adding 1 μL of the enzyme to the PCR reaction and incubating for 1-2 h at 37 °C, followed by 5 min at 80 °C to inactivate the enzyme. When required, the entire reaction was separated by gel electrophoresis and DNA was extracted from gels using the Wizard® SV Gel and PCR Clean-Up System (A9282, Promega), following the manufacturer's instructions, and quantified using a DS-11 Spectrophotometer (DS-11, DeNovix). Most DNA constructs were assembled following an enzyme-free cloning approach directly using *Dpn*I-treated unpurified PCR fragments[25] or by using T5 exonuclease as it was shown to improve assembly[26]. For this purpose, 1-2 μL of each corresponding PCR reaction were directly mixed with 10-100 μL of $CaCl_2$ competent *E. coli* TOP10 cells, in 1.5 mL tubes, and incubated on ice for 30 min. When using T5 exonuclease (M0363, NEB), 1 U of the enzyme was added to the DNA mixture and incubated for 5 min at room temperature and 3 min on ice, before adding the competent cells. Heat-shock was performed for 45 s at 42 °C and cells were subsequently incubated on ice for 2 min. LB medium was added up to 1 mL and the tubes were incubated at 37 °C for 1h with shaking (160 r.p.m.). The tubes were centrifuged for 3 min at 8.000 x *g*, 850 μL of the supernatant was discarded and the cell pellets were resuspended in the remaining medium and plated on LB-agar plates containing the appropriate antibiotic. When the DNA assemblies failed by this method, the NEBuilder® HiFi DNA Assembly Master Mix (E2621, NEB) was used following the manufacturer's instructions. Plasmid DNA was purified using the QIAprep Spin Miniprep Kit (27106, Qiagen) and the correct sequence was verified by Sanger sequencing (Source BioScience), prior to their use.

Unless stated differently, all the genes were cloned under the control of the arabinose-inducible promoter P_{araBAD} (BBa_I0500) and RBS30 (BBa_B0030). Initially, an RBS-like sequence upstream of a second ATG codon at the N-terminal sequence of mCherry was changed with synonymous mutations to prevent translation of a truncated protein and a hexahistidine-tag (H_6) was added to the reporter C-terminus to allow Western blot detection (see below); these modifications were introduced by PCR. The modified mCherry reporter was cloned under the control of P_{araBAD} and RBS30, in the pSB3T5 backbone, to be used as a positive control. Subsequently,

a split control of mCherry.H_6 was built to assess the reporter halves self-assembly: (*i*) the N-terminal sequence from amino acids 1 to 159, in pSB3T5, and (*ii*) the C-terminal from amino acids 160 to 242, in pSB1A3. A second control was built containing extra residues corresponding to the junction sequence of the intein gp41-1: (*i*) the three extra residues SGY were added to the C-terminal of $mCherry^N$ and (*ii*) the three extra residues SSS were added to the N-terminal of $mCherry^C$.H_6. To assess the effect of the insertion of the intein junction sequences on the reporter fluorescence, the six amino acid residues were introduced between mCherry.H_6 amino acid residues 159 and 160, by reverse PCR. The *cis*-splicing plasmids were produced by inserting the intein sequences between the respective junction sequences. For this purpose, the DNA sequences of the inteins M86, NpuSsp, gp41-1, gp41-8, NrdJ-1, IMPDH-1, SspDnaX, SspGyrB, TerThyX, TvoVMA and PhoRadA were amplified by PCR using primers containing sequences overlapping the sequences of the primers used to amplify the recipient plasmids. The remaining inteins were synthesized with the overlap regions and were directly assembled with the PCR amplified recipient plasmids. Flexible linkers were added to NpuSsp and TerThyX by reverse PCR. The N-termini plasmids for the *trans*-splicing constructs were produced by reverse PCR using the cis-splicing plasmids as a template. The C-termini plasmids for the *trans*-splicing constructs were produced by PCR amplification of the corresponding regions of the inteins and cloning them into the PCR amplified plasmid containing the $mCherry^C$.H_6 (pFP.R576). When indicated, the RBS was changed to RBS32 (BBa_B0032) or a solubility/expressivity tag[27] was added at the N-terminus of the split inteins' C-terminal. Flexible linkers were added to $NpuSsp^{N3}$, $NpuSsp^{C1}$, $TerThyX^{N3}$ and $TerThyX^{C1}$ by reverse PCR. To improve protein expression of the split intein fragments $M86^{C2}$ and $NpuSsp^{C2}$ fused to $Cherry^C$.H_6 for *in vitro* characterization, RBS30 and the solubility/expressivity tag were introduced by PCR and the fragment was subsequently cloned into the PCR amplified pET11 plasmid. To construct the plasmids for the split mCherry-based AND gates, the arabinose-inducible promoter of the plasmids encoding the selected C-terminal fusion proteins was replaced by the rhamnose-inducible promoter. For this purpose, the *rhaS*/P_{rhaBAD} regulatory element was amplified by PCR and assembled with the corresponding promoterless recipient plasmids, also amplified by PCR. AND gates behavior was improved replacing RBS30 by RBS32, using reverse PCR.

Three orthogonal extracytoplasmic function (ECF) sigma factors were selected to build split intein-based gates: ECF16_3622 (ECF16), ECF17_1691 (ECF17) and ECF20_992 (ECF20). Split ECF plasmids were produced by amplifying the N- and C-termini from the source plasmids and subsequently cloning them under the control of *araC*/P_{araBAD} in pSB3T5 and *rhaS*/P_{rhaBAD} in pSEVA221, respectively. The ECF cognate promoters P_{16_3622} (P_{16}), P_{17_Up1691} (P_{17}) and P_{20_992} (P_{20}) were constructed by annealing oligonucleotides containing restriction sites and subsequently cloned upstream of the composite module containing RBS30-*gfp*-B0015 in pSB4A3, following the BioBrick RFC[10] standard. Full ECFs were PCR amplified and cloned under the control of *araC*/P_{araBAD} in

pSB3T5. Linker mutations to the inteins' junction sequences were performed by reverse PCR. P_{16} and P_{17} were inserted upstream of composite modules containing RBS30-*mCherry*-B0015 in pSB3T5 and RBS30-*bfp*-B0015 in pSB1A3, respectively, by reverse PCR. Subsequently P_{16}-RBS30-*mCherry*-B0015 and P_{17}-RBS30-*bfp*-B0015 were transferred to pSB4A3 or assembled into the 3-reporter plasmid (P_{16}-RBS30-*mCherry*-B0015-P_{17}-RBS30-*bfp*-B0015-P_{20}-RBS30-*gfp*-B0015 in pSB4A3), following the BioBrick RFC[10] standard. Since we observed terminator read-through when testing $ECF16^{mut}$ (**Supplementary Fig. 21**), the orientation of P_{16}-*mCherry* in the 3-reporters plasmid was inverted to test the final circuit. For this purpose, the composite module containing *mCherry* under the control of P_{16} was reversed by PCR amplification using primers with restriction sites and subsequently cloned upstream of B0015 in pSB4A3. Afterwards, this module was cloned upstream of P_{17}-RBS30-*bfp*-B0015-P_{20}-RBS30-*gfp*-B0015 in pSB4A3. Split ECF x split intein AND gates were produced by amplification of the selected inteins fragments and subsequent cloning into the plasmids carrying the respective split ECF halves. Mutation to inactivate the inteins were performed by reverse PCR using the respective split ECF x split intein AND gates plasmids as template.

To build the three-input circuit, each arabinose-, rhamnose- or AHL-induced modules were produced separately by PCR amplifying each individual part and assembling them using T5 exonuclease, as described above. The terminator B0015 was cloned upstream of the rhamnose- and the AHL-induced modules and the three modules were assembled together following the BioBrick RFC[10].

The plasmids for the expression of full-length SasG proteins and SasG assembly units were built using the NEBuilder® HiFi DNA Assembly Master Mix, as described above. For plasmids containing the $SasG5^9E^6$ and $SasG5^{12}E^8$ encoding genes, the DNA insert for the final Gibson assembly was produced by Overlap Extension PCR, following the same parameters of PCR outlined above. However, for Overlap Extension PCR reactions, where the template for the reaction was two PCR products rather than plasmid DNA, for the first 10 cycles the annealing temperature was 50 °C, followed by 35 cycles with an annealing temperature of 72 °C. Full-length SasG proteins contain inteins junction sequences between each assembled $SasG5^3E^2$ to resemble the final assembly product and to facilitate cloning.

Supplementary Data (Provided as individual supplementary files)

Supplementary Data 1 | *In vivo* orthogonality data.

Supplementary Data 2 | *In vitro* orthogonality data.

Supplementary Data 3 | *In vitro* splicing conditions screening data.

Supplementary Data 4 | Sequences of the plasmids constructed in this work

Source Data | Source data underlying all reported averages in graphs and charts, and uncropped versions of any gels or blots presented in the figures.

References

1. Appleby-Tagoe JH, Thiel IV, Wang Y, Wang Y, Mootz HD & Liu X-Q. Highly efficient and more general *cis*- and *trans*-splicing inteins through sequential directed evolution. *J. Biol. Chem.* **286**, 34440-47 (2011).
2. Lockless SW & Muir TW. Traceless protein splicing utilizing evolved split inteins. *Proc. Natl. Acad. Sci. USA* **106**, 10999-1004 (2009).
3. Iwai H, Züger S, Jin J & Tam P-H. Highly efficient protein trans-splicing by a naturally split DnaE intein from *Nostoc punctiforme*. *FEBS Lett.* **580**, 1853-58 (2006).
4. Rusch DB, Halpern AL, Sutton G, Heidelberg KB, Williamson S, Yooseph S, Wu D, Eisen JA, Hoffman JM, Remington K *et al.* The Sorcerer II Global Ocean Sampling expedition: northwest Atlantic through eastern tropical Pacific. *PLoS Biol.* **5**, e77 (2007).
5. Dassa B, London N, Stoddard BL, Schueler-Furman O & Pietrokovski S. Fractured genes: a novel genomic arrangement involving new split inteins and a new homing endonuclease family. *Nucleic Acids Res.* **37**, 2560-73 (2009).
6. Carvajal-Vallejos P, Pallissé R, Mootz HD & Schmidt SR. Unprecedented rates and efficiencies revealed for new natural split inteins from metagenomic sources. *J. Biol. Chem.* **287**, 28686-96 (2012).
7. Lin Y, Li M, Song H, Xu L, Meng Q & Liu X-Q. Protein *trans*-splicing of multiple atypical split inteins engineered from natural inteins. *PLoS ONE* **8**, e59516 (2013).
8. Aranko AS, Oeemig JS, Zhou D, Kajander T, Wlodawer A & Iwaï H. Structure-based engineering and comparison of novel split inteins for protein ligation. *Mol. Biosyst.* **10**, 1023-34 (2014).
9. Perler FB. InBase: the Intein Database. *Nucleic Acids Res.* **30**, 383-84 (2002).
10. Amitai G, Dassa B & Pietrokovski S. Protein splicing of inteins with atypical glutamine and aspartate C-terminal residues. *J. Biol. Chem.* **279**, 3121-31 (2004).
11. Powers TL, Drago MJ, Dorval DM, Connor KR & Mills KV. Protein splicing of a non-canonical *Clostridium thermocellum* intein with N-terminal Gln. *FASEB J.* **20**, A964-A64 (2006).
12. Tori K, Dassa B, Johnson MA, Southworth MW, Brace LE, Ishino Y, Pietrokovski S & Perler FB. Splicing of the mycobacteriophage Bethlehem DnaB intein: identification of a new mechanistic class of inteins that contain an obligate block F nucleophile. *J. Biol. Chem.* **285**, 2515-26 (2010).
13. Tori K & Perler FB. Expanding the definition of Class 3 inteins and their proposed phage origin. *J. Bacteriol.* **193**, 2035-41 (2011).
14. Iwaï H, Lingel A & Plückthun A. Cyclic green fluorescent protein produced *in vivo* using an artificially split PI-*Pfu*I intein from *Pyrococcus furiosus*. *J. Biol. Chem.* **276**, 16548-54 (2001).
15. Komori K, Fujita N, Ichiyanagi K, Shinagawa H, Morikawa K & Ishino Y. PI-*Pfu*I and PI-*Pfu*II, intein-coded homing endonucleases from *Pyrococcus furiosus*. I. Purification and identification of the homing-type endonuclease activities. *Nucleic Acids Res.* **27**, 4167-74 (1999).
16. Southworth MW, Benner J & Perler FB. An alternative protein splicing mechanism for inteins lacking an N-terminal nucleophile. *EMBO J.* **19**, 5019-26 (2000).
17. Ellilä S, Jurvansuu JM & Iwaï H. Evaluation and comparison of protein splicing by exogenous inteins with foreign exteins in *Escherichia coli*. *FEBS Lett.* **585**, 3471-77 (2011).
18. Shetty RP, Endy D & Knight TF. Engineering BioBrick vectors from BioBrick parts. *J. Biol. Eng.* **2**, 5 (2008).
19. Liu Y, Wan X & Wang B. Engineered CRISPRa enables programmable eukaryote-like gene activation in bacteria. *Nat. Commun.* **10**, 3693 (2019).
20. Wang B, Barahona M & Buck M. Amplification of small molecule-inducible gene expression via tuning of intracellular receptor densities. *Nucleic Acids Res.* **43**, 1955-64 (2015).
21. Rhodius VA, Segall-Shapiro TH, Sharon BD, Ghodasara A, Orlova E, Tabakh H, Burkhardt DH, Clancy K, Peterson TC, Gross CA *et al.* Design of orthogonal genetic switches based on a crosstalk map of σs, anti-σs, and promoters. *Mol. Syst. Biol.* **9**, 702 (2013).
22. Chen Y-J, Liu P, Nielsen AAK, Brophy JAN, Clancy K, Peterson T & Voigt CA. Characterization of 582 natural and synthetic terminators and quantification of their design constraints. *Nat. Methods* **10**, 659 (2013).
23. Silva-Rocha R, Martínez-García E, Calles B, Chavarría M, Arce-Rodríguez A, de Las Heras A, Páez-Espino AD, Durante-Rodríguez G, Kim J, Nikel PI *et al.* The Standard European Vector Architecture (SEVA): a coherent platform for the analysis and deployment of complex prokaryotic phenotypes. *Nucleic Acids Res.* **41**, D666-D75 (2013).
24. Gruszka DT, Whelan F, Farrance OE, Fung HKH, Paci E, Jeffries CM, Svergun DI, Baldock C, Baumann CG, Brockwell DJ *et al.* Cooperative folding of intrinsically disordered domains drives assembly of a strong elongated protein. *Nat. Commun.* **6**, 7271 (2015).
25. Li C, Wen A, Shen B, Lu J, Huang Y & Chang Y. FastCloning: a highly simplified, purification-free, sequence- and ligation-independent PCR cloning method. *BMC Biotechnol.* **11**, 92-92 (2011).
26. Xia Y, Li K, Li J, Wang T, Gu L & Xun L. T5 exonuclease-dependent assembly offers a low-cost method for efficient cloning and site-directed mutagenesis. *Nucleic Acids Res.* **47**, e15-e15 (2018).
27. Hansted JG, Pietikäinen L, Hög F, Sperling-Petersen HU & Mortensen KK. Expressivity tag: A novel tool for increased expression in *Escherichia coli*. *J. Biotechnol.* **155**, 275-83 (2011).
