# Supplementary material for: An expanded library of orthogonal split inteins enables modular multi-peptide assemblies
Source: Nat Commun. 2020 Mar 23;11:1529. doi: 10.1038/s41467-020-15272-2 (PMC7090010; doi:10.1038/s41467-020-15272-2)
Supplement: Supplementary file 2 — Description of Additional Supplementary Files [file 41467_2020_15272_MOESM2_ESM.docx]

**Description of Additional Supplementary Files**

**Title:** Supplementary Data 1

**Description:** *In vivo* orthogonality data. Background subtracted Fluorescence/OD_600_ (a.u.) measured in the *in vivo* orthogonality assessment in Figure 2.

**Title:** Supplementary Data 2

**Description:** *In vitro* orthogonality data. Normalized fluorescence (a.u.) measured in the *in vitro* orthogonality assessment in Figure 3.

**Title:** Supplementary Data 3

**Description:** *In vitro* splicing conditions screening data. Normalized fluorescence (a.u.) measured in the screening performed in Supplementary Figure 12.

**Title:** Supplementary Data 4

**Description:** Sequences of the plasmids constructed in this work.
