# Supplementary material for: An expanded library of orthogonal split inteins enables modular multi-peptide assemblies
Source: Nat Commun. 2020 Mar 23;11:1529. doi: 10.1038/s41467-020-15272-2 (PMC7090010; doi:10.1038/s41467-020-15272-2)
Supplement: Supplementary file 7 — Reporting Summary [file 41467_2020_15272_MOESM7_ESM.pdf]

nature research

Corresponding author(s): Baojun Wang
Last updated by author(s): Feb 21, 2020

Reporting Summary

Nature Research wishes to improve the reproducibility of the work that we publish. This form provides structure for consistency and transparency in reporting. For further information on Nature Research policies, see Authors & Referees and the Editorial Policy Checklist .

Statistics

For all statistical analyses, confirm that the following items are present in the figure legend, table legend, main text, or Methods section.

n/a	Confirmed	
☐	☒	The exact sample size (*n*) for each experimental group/condition, given as a discrete number and unit of measurement
☐	☒	A statement on whether measurements were taken from distinct samples or whether the same sample was measured repeatedly
☐	☒	The statistical test(s) used AND whether they are one- or two-sided<br>*Only common tests should be described solely by name; describe more complex techniques in the Methods section.*
☐	☒	A description of all covariates tested
☐	☒	A description of any assumptions or corrections, such as tests of normality and adjustment for multiple comparisons
☐	☒	A full description of the statistical parameters including central tendency (e.g. means) or other basic estimates (e.g. regression coefficient) AND variation (e.g. standard deviation) or associated estimates of uncertainty (e.g. confidence intervals)
☐	☒	For null hypothesis testing, the test statistic (e.g. *F*, *t*, *r*) with confidence intervals, effect sizes, degrees of freedom and *P* value noted<br>*Give P values as exact values whenever suitable.*
☒	☐	For Bayesian analysis, information on the choice of priors and Markov chain Monte Carlo settings
☒	☐	For hierarchical and complex designs, identification of the appropriate level for tests and full reporting of outcomes
☒	☐	Estimates of effect sizes (e.g. Cohen's *d*, Pearson's *r*), indicating how they were calculated

Our web collection on statistics for biologists contains articles on many of the points above.

Software and code

Policy information about availability of computer code

Data collection	Image Lab v5.2; Image Studio v5.2; Omega Control v5.11 R4.
Data analysis	Image Lab v5.2; Image Studio Lite v5.2; Omega MARS Data Analysis Software v3.32; Microsoft Excel 2013; GraphPad Prism v8.1; MOTIF (https://www.genome.jp/tools/motif/); PROMALS3D (http://prodata.swmed.edu/promals3d/promals3d.php); BioNJ (http://www.phylogeny.fr/one_task.cgi?task_type=bionj); TreeDyn 198.3 (http://www.phylogeny.fr/one_task.cgi?task_type=treedyn); GeneOptimizer™ algorithm (https://www.thermofisher.com/), Gene Designer v2.0; SWISS-MODEL (http://swissmodel.expasy.org/); Swiss-PdbViewer v4.1; POV-Ray v3.7; Tm Calculator (https://www.thermofisher.com/).

For manuscripts utilizing custom algorithms or software that are central to the research but not yet described in published literature, software must be made available to editors/reviewers. We strongly encourage code deposition in a community repository (e.g. GitHub). See the Nature Research guidelines for submitting code & software for further information.

Data

Policy information about availability of data

All manuscripts must include a data availability statement. This statement should provide the following information, where applicable:

- Accession codes, unique identifiers, or web links for publicly available datasets
- A list of figures that have associated raw data
- A description of any restrictions on data availability

All data and plasmids supporting the findings are available from the corresponding author upon reasonable request. Representative plasmids of the 34 selected inteins, as well as the plasmid for the 3-input/3-output logic circuit and the corresponding circuit output reporter plasmid are available from Addgene (ID in brackets): pFP.R265 (138167), pFP.R400 (138175), pFP.R267 (138179), pFP.R268 (138180), pFP.R269 (138181), pFP.R270 (138219), pFP.R271 (138220), pFP.R272 (138221), pFP.R401 (138222), pFP.R274 (138223), pFP.R275 (138224), pFP.R355 (138225), pFP.R356 (138226), pFP.R357 (138227), pFP.R358 (138228), pFP.R359 (138229), pFP.R360 (138230), pFP.R361 (138231), pFP.R362 (138232), pFP.R363 (138233), pFP.R364 (138234), pFP.R365 (138235), pFP.R366 (138236), pFP.R367 (138237), pFP.R368 (138238), pFP.R369 (138239), pFP.R370 (138240), pFP.R371 (138241), pFP.R372 (138242), pFP.R373 (138243), pFP.R374 (138244), pFP.R375

(138245), pFP.R376 (138246), pFP.R377 (138247), pFP.E222 (138248), pFP.E227 (138249). The source data and full scan figures for the protein gels and Western blots underlying Figs. 1d-e, 2b, 3a-b and d, 4b-h and j-l, 5d and 6b and d-e and Supplementary Figs. 2d, 3b, 4a-c, 5a-b, 7, 8a-b, 9a-b, 10a-b, 11a-b, 13a-x, 14a-b, 15a-l, 16a-b, 17a-g, 18a-c, 19c and f, 20b-c, 21d-f and h-j, 22b-c, 23b-c, 24b-c, 25e-g, 26a-d, 27a-b and, 28a-b and 29 are provided as a Source Data file.

Field-specific reporting

Please select the one below that is the best fit for your research. If you are not sure, read the appropriate sections before making your selection.

- [x] Life sciences
- [ ] Behavioural & social sciences
- [ ] Ecological, evolutionary & environmental sciences

For a reference copy of the document with all sections, see nature.com/documents/nr-reporting-summary-flat.pdf

Life sciences study design

All studies must disclose on these points even when the disclosure is negative.

Sample size	Sample sizes (= number of replicate experiments) were chosen according to common practices in the field and were such that standard deviations were small enough to allow determination of significant effects and trends. In most cases, at least 3 independent replicates were carried out for each experiment. Only 2 replicates were performed when the experiment only aimed at screening reaction conditions.
Data exclusions	No data were excluded from the analyses.
Replication	All in vivo experiments were performed with three or more biological replicates. All experiments in vitro were performed with two or more independent replicates. All attempts at replication were successful.
Randomization	Each biological replicate of a bacterial culture used for in vivo experiments was obtained by liquid selection from transformation in 96-well plates. The fact that in some cases a random transformation replicate didn't grow in medium supplemented with antibiotics shows that the bacterial cells transferred from the transformation mix are not homogeneous and that randomization is attained by this selection method. For in vitro experiments bacterial cultures were inoculated from a single colony, which was randomly chosen from an agar plate.
Blinding	No blinding was involved as it was not relevant to this study because our data is not based on qualitative scoring metrics. No animal or human research participants were utilized and all samples were processed in parallel. Blinding during group allocation is also irrelevant in our study because samples of bacterial cultures that were split into different conditions were random samplings and there is no control over which cells will be selected and thus there is no bias during group allocation.

Reporting for specific materials, systems and methods

We require information from authors about some types of materials, experimental systems and methods used in many studies. Here, indicate whether each material, system or method listed is relevant to your study. If you are not sure if a list item applies to your research, read the appropriate section before selecting a response.

Materials & experimental systems

n/a	Involved in the study	
[ ]	[x]	Antibodies
[x]	[ ]	Eukaryotic cell lines
[x]	[ ]	Palaeontology
[x]	[ ]	Animals and other organisms
[x]	[ ]	Human research participants
[x]	[ ]	Clinical data

Methods

n/a	Involved in the study	
[x]	[ ]	ChIP-seq
[x]	[ ]	Flow cytometry
[x]	[ ]	MRI-based neuroimaging

Antibodies

Antibodies used	Primary: Anti RFP-tag, pAb, Rabbit (A00682, GenScript) and THE His Tag Antibody, mAb, Mouse (A00186, GenScript); Secondary: IRDye 680RD Goat anti-Rabbit (925-68071, Li-cor) and IRDye 800CW Goat anti-Mouse (925-32210, Li-cor).

Validation

Validation for each primary antibody is provided on the manufacturers' websites:

GenScript Anti RFP-tag, pAb, Rabbit #A00682: https://www.genscript.com/antibody/A00682-RFP_tag_Antibody_pAb_Rabbit.html
GenScript THE His Tag Antibody, mAb, Mouse #A00186: https://www.genscript.com/antibody/A00186-THE_sup_TM_sup_His_Tag_Antibody_mAb_Mouse.html

In our work, we assessed the cross-reactivity of the primary antibodies against native E. coli proteins by using cells transformed with an empty plasmid, thus not carrying any proteins with the specific epitopes recognized by the antibodies used (see Supplementary Figure 4a, last lane named 'Empty plasmids').
